# Fake IDs? Widespread misannotation of DNA transposons as a general transcription factor

**DOI:** 10.1186/s13059-023-03102-9

**Published:** 2023-11-13

**Authors:** Nozhat T. Hassan, David L. Adelson

**Affiliations:** School of Biological Sciences, University of Adelaide, North Terrace, Adelaide, South Australia 5005 Australia

**Keywords:** Transposable element, Genome, Annotation, Transcription factor, GTF2, DNA transposon

## Abstract

Accurate annotation of genes and transposable elements (TEs) is vital for understanding genomes, but current annotation pipelines often misannotate TEs as genes. This study reveals how the general transcription factor II-I repeat domain-containing protein 2 (GTF2IRD2) erroneously annotated DNA transposons in non-mammalian species, as it contains a 3′ fused hAT transposase domain. We also demonstrate the generality of this problem by identifying misannotated TEs as genes in other vertebrate genomes. Such misannotations can lead to errors in phylogenetic analyses and wasted time for investigators. The study proposes adding a final TE-check to gene annotation pipelines to mitigate this problem.

**Supplementary Information:**

The online version contains supplementary material available at 10.1186/s13059-023-03102-9.

## Background

Annotation of transposable elements (TEs) is essential for understanding genome structure and function; however, misannotation may result in erroneous classification of TEs as *trans-*regulatory elements such as transcription factors. TEs can introduce genetic novelty to the host genome, and an example of this is the exaptation of TEs into the human general transcription factor II-I repeat domain-containing protein 2 (GTF2IRD2). GTF2IRD2 contains a Charlie8-like element positioned at the 3′ end (C terminus) of the gene model/full protein that has retained transposable element features such as catalytically active DDE/RW amino acids (D; aspartic acid, E; glutamic acid, R; arginine, and W; tryptophan) required for transposition [1]. The Charlie transposon (DNA transposon; hAT superfamily) is an ancient autonomous group of transposons abundant in mammalian genomes, including humans [2]. Charlie transposons are defined by their target site duplication (TSDs) and terminally inverted repeats (TIRs). At the same time, the protein-coding sequence of the transposase may vary between Charlie elements from different species [3]. GTF2IRD2 is found in mammals and is predicted to be in many reptiles, amphibians, and bony fishes [4]. However, upon closer inspection, some non-mammalian GTF2IRD2 sequences appear to be hAT transposons, not transcription factors. Here, we have used structural and phylogenetic analysis to resolve the widespread misannotation of non-mammalian DNA transposons as GTF2IRD2 transcription factors. In addition, we demonstrated that the issue of misannotation is indeed widespread by finding several instances of TEs of different classes incorrectly predicted as genes in a selection of genome assemblies. We believe that this paper addresses an important issue; it has implications not only for the study of TEs but is also relevant as similar misannotations could also cause the misinterpretation of other results that depend on reliable gene annotation.

## Results and discussion

**Fig. 1:**
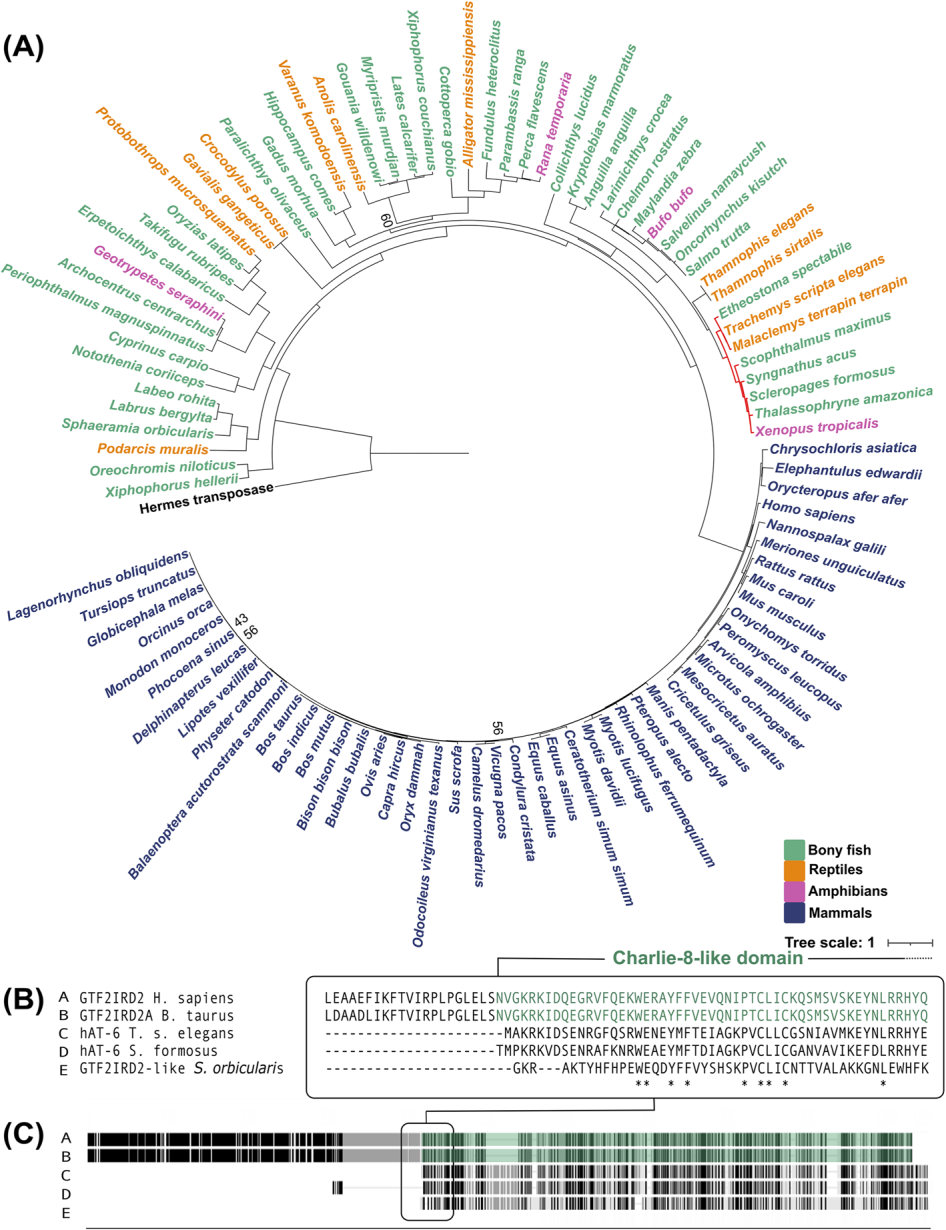
GTF2IRD2/2A is used to erroneously misannotate DNA TEs. **A** The phylogenetic relationship of sequences annotated as GTF2IRD2/2A from bony fishes, reptilian, amphibian, and mammalian genomes from NCBI. Bony fish are coloured green, reptiles are orange, amphibians are pink, and mammals are blue. Branches coloured in red are hAT-6 transposons. The Hermes transposase was used as an outgroup. Support values under 60 are shown at nodes. For the full tree and support values, see Additional file 4. **B** Boundary of non-transposon and Charlie8-like domain of a multiple sequence alignment (MSA) of mammalian GTF2IRD2/2A to hAT-6 transposons and a TE sequence misannotated as GTF2IRD2-like. **C** Full schematic MSA of the selected sequences. The length of sequences in amino acids is shown on the *x*-axis from the 5′ to 3′ direction. The Charlie8-like domain of mammalian GTF2IRD2/2A is highlighted in red text and red shading. ‘*’ denotes a single, fully conserved amino acid within the alignment

While annotating hAT-6 transposons in Testudines genomes, we noticed that GTF2IRD2/2A, a human general transcription factor, was the top BLASTN result when using hAT-6 transposons as a query to search non-mammalian genomes. This was unexpected as hAT-6 has hallmarks of a functional transposase, such as TIRs and TSDs, and has the functional motifs required for transposition, such as the DDE/RW residues in the translated open reading frame (ORF) [4, 5]. NCBI’s eukaryotic gene annotation pipeline prefers to use experimental evidence when annotating genes but uses an ab initio model to predict optimal coding sequence (CDS) alignments when there is no experimental data (https://www.ncbi.nlm.nih.gov/genome/annotation_euk/process/). Specifically, Gnomon is meant to exclude gene predictions with high homology to transposable or retro-transposable elements from the final gene models; however, the eukaryotic annotation pipeline appears to lack a final TE filtering step after integrating RefSeq annotations. This may explain how genes such as GTF2IRD2/2A, which contains an integrated Charlie8-like element (a hAT-like transposase), can lead to hAT-6 being predicted to be a transcription factor in non-mammalian genomes [3]. When searching Interpro for GTF2IRD2/2A sequences, we saw that 485 proteins were annotated as GTF2IRD2/2A proteins, but only 4 of these have been reviewed in human, cow and mouse genomes (https://www.ebi.ac.uk/interpro/entry/InterPro/IPR042224/protein/reviewed/#table, Accessed 11th November 2022). To determine if additional hAT transposons were incorrectly annotated as GTF2IRD2/2A, we examined the phylogeny of a set of protein sequences annotated as GTF2IRD2/2A or GTF2IRD2/2A-like from mammals, reptiles, bony fishes, and amphibians (Fig. 1A). Birds were excluded from analysis as the sequences annotated as GTF2IRD2/2A had no significant similarity to either mammalian GTF2IRD2/2A or to any DNA TE structures. Any sequence homology in birds was limited to the N terminus of the mammalian GTF2IRD2/2A, an indication of potential similarity to the ancestral GTF2IRD2/2A protein prior to the exaptation of Charlie8 (Additional file 1: Table S1).

There was a clear distinction between mammalian and non-mammalian GTF2IRD2/2A gene models. Mammalian GTF2IRD2/2A are correctly annotated as transcription factors as they have an N terminus ~ 400 aa long containing a GTF2I-Like repeat domain, a zinc finger binding domain, and an integrated Charlie8-like element at the C terminus [1]. However, this is not the case with non-mammalian sequences. Multiple alignment of GTF2IRD2/2A from mammals and non-mammals with hAT-6 transposons exclusively shows high similarity alignment at the 3′ end (Fig. 1B, C). This is consistent with the position of the Charlie-like element in GTF2IRD2/2A, demonstrating that non-mammalian GTF2IRD2/2A are not likely to be transcription factors (TFs), but TEs.

Non-mammalian GTF2IRD2/2A from fish, reptiles, and frogs were manually curated to identify signature motifs of DNA transposons as they appear more closely related to hAT-6 transposons than mammalian GTF2IRD2/2A. Twenty-eight sequences were classified as autonomous hAT transposons from several different species, particularly from the genome of the Atlantic salmon (*Salmo salar*) (Additional file 1: Table S2). TSDs and TIRs are characteristic of hAT transposons and were found in most sequences, while sequences without them were classified as partial, non-autonomous transposons (Additional file 1: Table S3). Furthermore, DDE/RW residues required for transposition were identified for most of the newly curated hAT transposons (Additional file 2). Finally, the 5′ and 3′ TIRs were mostly conserved across the misannotated hAT transposons, which further demonstrates the degree of misannotation (Additional file 1: Table S4).

While we have demonstrated the widespread effects of misannotation in a single case of hAT-6 transposons, we wanted to determine if this was an isolated case or whether the problem extends to other TE classes and predicted gene models. To examine the potential magnitude of TE misannotation, we assessed a dataset of mammalian and non-mammalian vertebrate genome assemblies for instances of TE annotations intersecting with gene annotations from the UCSC Genome Browser [6]. We focused specifically on instances where 100% of a TE sequence overlapped with the protein-coding region (CDS) of gene and gene predictions.

**Fig. 2:**
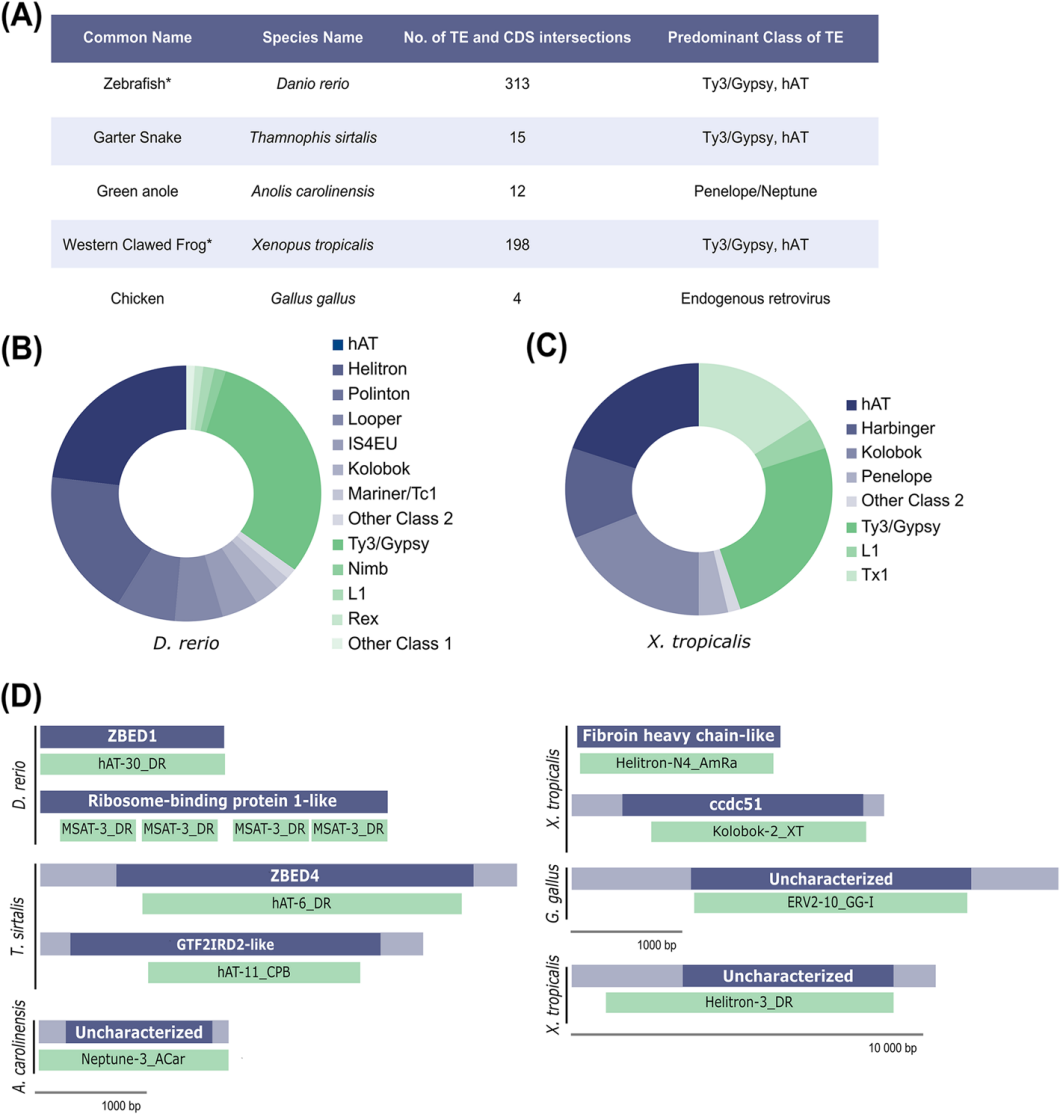
Frequency and types of TEs found in overlapping events with predicted protein-coding regions (CDS) of genome assemblies. **A** Number of intersections of TEs with predicted protein-coding regions (CDS) of genome assemblies of various vertebrates. Only TEs with 100% sequence overlap with protein-coding regions are shown. The predominant type of TE found for each species is shown. Species with (*) show unusually high levels of overlapping events. **B**, **C** Class 1 TEs are shown in green, and class 2 are shown in navy. **B** Types of TEs found in *Danio rerio* (zebrafish) and **C** types of TEs found in *Xenopus tropicalis* (western clawed frog). Other class 1 and 2 elements for **B** include Crypton, Harbinger, I, Ngaro, and Proto1. Other class 2 for **C** include piggyBac and Polinton. **D** Illustrating a sample of TEs overlapping with predicted protein-coding regions (CDS) from various vertebrates. The approximate length and relative location of the protein-coding regions are shown in dark navy, untranslated regions in light navy and TEs are in green

As shown in Fig. 2A, we found hundreds of instances of TEs overlapping completely with the CDS annotations of non-mammalian vertebrate genomes. Notably, overlapping events were very abundant in zebrafish and the western clawed frog genomes, both of which are used widely in a variety of biochemical and evolutionary analyses. In both the zebrafish and western clawed frog, a diverse range of TE classes were found overlapping with genes (Fig. 2B, C). The predominant class of TE were hAT DNA transposons and Ty3/Gypsy LTR retrotransposons. This observation is consistent with both the distribution and high abundance of hAT and Ty3/Gypsy elements across vertebrates [2, 7].

In contrast, in mammalian genomes, all overlapping events were correctly annotated as ‘transposase-like’ in the corresponding gene records in NCBI. This was expected as there have been significantly more concerted efforts to curate mammalian genomes with respect to both genes and repeats. The same was true for most avian genomes investigated for misannotation as these have benefited from developing interest in high-quality annotation [8]. This level of annotation detail was notably absent in the other vertebrate genomes studied as instances where TEs overlapped with genes were not noted in the gene records.

While the number of overlapping events in vertebrates highlights how widespread misannotation can be, we further confirmed this by manually curating a sample of genes for all vertebrate genomes investigated for TE(s). Some genes predicted to be proteins or left uncharacterised are actually TEs (Additional file 1: Table S5). DNA transposable elements were often misannotated as zinc finger binding proteins. This is not surprising, as autonomous DNA TE ORFs encode DNA binding domains which can explain why some TEs were mistakenly predicted as non-TE genes. Uncharacterised loci were the predominant type of misannotation for a variety of both class 1 and 2 TEs. This finding shows a deficiency in genome annotation pipelines in combining RepeatMasker results to properly characterise unknown genes, which with minimal manual curation can be correctly annotated as TE sequences. Figure 2D illustrates a selection of TEs overlapping with gene annotations. In most cases, the TE spans the majority of the CDS, further showing that the misannotation of TEs is not limited to a particular class of TE or type of gene prediction.

We focused specifically on the CDS of gene predictions and instances where 100% of the TE overlaps with those predictions. Even with stringent parameters and a limited number of organisms, we have demonstrated that misannotation occurs frequently and we strongly encourage better curation of repeats as it affects gene annotation for a more comprehensive picture of genomes, especially of emerging fish and reptile genomes. A simple additional screening for sequence homology of gene models with TEs as the last step of gene model annotation would mitigate/eliminate this problem.

## Conclusions

In this study, we present a case of widespread incorrect annotation of hAT DNA transposons as GTF2IRD2/2A. This has led to at least 28 new instances of hATs from reptiles, bony fishes, and amphibians that had been overlooked and could have been incorrectly used as transcription factors in other analyses. We have also demonstrated that misannotation occurs frequently for different types of both class 1 and class 2 TEs, especially across vertebrate genomes. Correct annotation is a vital step in furthering our understanding of genome evolution, and misannotation of TEs as *trans-*regulatory genes such as TFs affects downstream research and can confound phylogenetic analysis.

## Methods

### Manual curation of hAT transposons from GTF2IRD2/2A sequences

A set of GTF2IRD2/2A and GTF2IRD2/2A-like protein sequences were downloaded from NCBI. The search was limited to species belonging to the *Actinopterygii*, *Reptilia*, and *Amphibia* classes. To determine whether these GTF2IRD2/2A proteins were actually DNA transposons, extensive manual curation was performed to locate characteristic sequence features such as TIRs, TSDs, and ORFs. To identify hAT transposons, hAT-6_TSE (manuscript in prep) was used as a query in a BLASTP 2.7.1 + search against a set of mammal, reptile, amphibian, bony fish, and bird genomes containing GTF2IRD2/2A gene annotations [9]. The corresponding nucleotide sequence of each top hit was extended 1000 bp in flanking regions where possible and used for manual annotation of TIRs and TSDs characteristic of hAT transposons. ORFs were searched using GENSCAN (http://hollywood.mit.edu/GENSCAN.html) and searched for DD/E and RW residues. Sequences that contained 5′ and 3′ TSDs, TIRs, and an intact ORF were classified as autonomous hAT transposons [9, 10] (Additional file 1: Table S6). The best match for each new autonomous hAT was found using Repbase (https://www.girinst.org/repbase/; accessed August 2020), and both 5′ and 3′ TIRs were aligned using MAFFT v7.310 to view conserved nucleotides [11, 12]. hATs misannotated as GTF2IRD2-like were aligned using MAFFT to mammalian GTF2IRD2/2A to confirm they had homology to GTF2IRD2/2A’s Charlie8-like domain (Additional file 3).

### Tree-building

GTF2IRD2/2A and GTF2IRD2/2A-like protein sequences from mammals, reptiles, amphibians, and bony fishes were aligned to the Hermes and hAT-6 transposons using MAFFT v7.319 [13]. The alignment was trimmed using CLipKit v1.3.0, and IQTree v1.6.12 was used for tree reconstruction with JTT + F + I + G4 as the best-fit model with 20 maximum likelihood trees and 1000 bootstraps [14–17].

### Determining whether the misannotation of TEs is a recurring event or limited to hAT-6 s

To examine the breadth of TE misannotation in other organisms, gene and gene prediction files were downloaded from the UCSC Genome Browser (https://genome.ucsc.edu/index.html) and the corresponding RepeatMasker files for mammals and vertebrates. Using BEDTools v2.30.0, the RepeatMasker output was overlaid with the gene and gene prediction files to extract regions of overlap between TEs and genes [18]. Stringent parameters were used to exclude only simple repeats and include TEs that overlapped 100% with protein-coding regions (CDS), which were selected for analysis (bedtools intersect -a repeats.bed -b cds.bed -wo -f 1.00 > 100_overlap_cds.bed). A sample of genes that overlapped with TEs greater than 1000 base pairs were selected to check whether any true misannotation event occurred. The best TE match for the sample of genes was found through Repbase [11]. Assemblies used for analysis, frequency (TE:Protein-coding gene count), and coordinates of misannotation events are in Additional file 1: Table S7.

### Supplementary Information

**Additional file 1: Table S1.** Pairwise Identity Matrix of predicted GTF2IRD2/2A sequences in birds vs GTF2IRD2/2A in mammals. **Table S2.** Species from the Actinopterygii, Reptilia and Amphibia class containing hAT transposons that were incorrectly annotated as GTF2IRD2/2A. **Table S3.** 3′ and 5′ TIRs of TEs misannotated as GTF2IRD2/2A. **Table S4.** Multiple alignment of the 5′ and 3′ TIRs of Actinopterygii, Amphibia and Reptilia hAT transposons derived from sequences misannotated as GTF2IRD2/2A. **Table S5.** A sample of TEs from vertebrates which were incorrectly annotated as various other proteins and uncharacterised loci. **Table S6.** Nucleotide sequences TEs misannotated as GTF2IRD2/2A. **Table S7.** Sample of misannotation events. **Table S8.** Conserved domains of GTF2IRD2/2A compared to TEs

**Additional file 2.** DDE/RW positions in new hAT TEs.

**Additional file 3.** Text file of multiple alignment of mammalian GTF2IRD2/2A to predicted GTF2IRD2/2A sequences in vertebrates

**Additional file 4.** Full support values for Fig. 1 phylogenetic tree.

**Additional file 5. **Peer review history.

## Data Availability

The dataset(s) supporting the conclusions of this article are included within the article (and its additional file(s)).

## Abbreviations

GTF2IRD2: General transcription factor II-I repeat domain-containing protein 2
GTF2IRD2A: General transcription factor II-I repeat domain-containing protein 2A
