# Supplementary material for: Fake IDs? Widespread misannotation of DNA transposons as a general transcription factor
Source: Genome Biol. 2023 Nov 13;24:260. doi: 10.1186/s13059-023-03102-9 (PMC10641963; doi:10.1186/s13059-023-03102-9)
Supplement: Supplementary file 1 — Additional file 1: Table S1. Pairwise Identity Matrix of predicted GTF2IRD2/2A sequences in birds vs GTF2IRD2/2A in mammals. Table S2. Species from the Actinopterygii, Reptilia and Amphibia class containing hAT transposons that were incorrectly annotated as GTF2IRD2/2A. Table S3. 3′ and 5′ TIRs of TEs misannotated as GTF2IRD2/2A. Table S4. Multiple alignment of the 5′ and 3′ TIRs of Actinopterygii, Amphibia and Reptilia hAT transposons derived from sequences misannotated as GTF2IRD2/2A. Table S5. A sample of TEs from vertebrates which were incorrectly annotated as various other proteins and uncharacterised loci. Table S6. Nucleotide sequences TEs misannotated as GTF2IRD2/2A. Table S7. Sample of misannotation events. Table S8. Conserved domains of GTF2IRD2/2A compared to TEs [file 13059_2023_3102_MOESM1_ESM.pdf]

Table S1: Pairwise Identity Matrix of predicted GTF2IRD2/2A sequences in birds vs GTF2IRD2/2A in mammals

	**Antrostomus_caroli**	**Phasianus_colchicu**	**Camarhynchus_par**	**Pterocles_gutturalis**	**Calypte_anna**	**Haliaeetus_leucoce**	**Aptenodytes_forste**	Condylura_cristata	Vulpes_lagopus	Rhinolophus_ferrume	Bos_taurus	Monodon_monocero	Mus_caroli	Mesocricetus_auratus
**Antrostomus_carolinensis**	100	86.5	86.08	86.96	88.41	89.81	89.71	24.65	25.41	25.54	24.84	24.9	22.88	23.78
**Phasianus_colchicus**	86.5	100	90.52	91.07	91.04	93.37	92.9	23.64	24.22	24.45	23.93	24.1	22.3	22.81
**Camarhynchus_parvulus**	86.08	90.52	100	91.41	92.34	93.73	94.11	24.16	24.4	24.76	24.58	25	22.92	23.44
**Pterocles_gutturalis**	86.96	91.07	91.41	100	92.02	95.03	94.67	23.25	23.84	23.84	23.31	23.49	21.6	22.4
**Calypte_anna**	88.41	91.04	92.34	92.02	100	94.69	94.98	23.85	24.32	24.32	24.15	24.44	22.3	23.37
**Haliaeetus_leucocephalus**	89.81	93.37	93.73	95.03	94.69	100	97.03	23.89	24.36	24.48	24.19	24.36	22.44	23.19
**Aptenodytes_forsteri**	89.71	92.9	94.11	94.67	94.98	97.03	100	24.06	24.65	24.65	24.36	24.65	22.84	23.35
Condylura_cristata	24.65	23.64	24.16	23.25	23.85	23.89	24.06	100	87.34	87.66	88.19	89.24	75.79	77.29
Vulpes_lagopus	25.41	24.22	24.4	23.84	24.32	24.36	24.65	87.34	100	90.08	89.14	91.03	76.77	77.07
Rhinolophus_ferrumequinum	25.54	24.45	24.76	23.84	24.32	24.48	24.65	87.66	90.08	100	90.51	91.46	77.75	78.15
Bos_taurus	24.84	23.93	24.58	23.31	24.15	24.19	24.36	88.19	89.14	90.51	100	94.09	76.34	77.61
Monodon_monoceros	24.9	24.1	25	23.49	24.44	24.36	24.65	89.24	91.03	91.46	94.09	100	77.86	78.9
Mus_caroli	22.88	22.3	22.92	21.6	22.3	22.44	22.84	75.79	76.77	77.75	76.34	77.86	100	85.03
Mesocricetus_auratus	23.78	22.81	23.44	22.4	23.37	23.19	23.35	77.29	77.07	78.15	77.61	78.9	85.03	100

Greater than 70% indentity

Less than 30% identity

Bird species are in bold text

Class	Species Name	Length (bp)	Repbase Best Match	Pairwise Identity (%)
Actinopterygii	*Sphaeramia orbicularis*	2921	hAT-1_SSa (*Salmo salar)*	81
	Xiphophorus hellerii	3231	hAT-1_XT (*Xenopus tropicalis)*	79
	Archocentrus centrarchus	2522	hAT-2_PM *(Petromyzon marinus)*	83
	Takifugu rubripes	2612	hAT-7_PM *(Petromyzon marinus)*	82
	Erpetoichthys calabaricus	2591	hAT-4_SSa (*Salmo salar)*	88
	Oryzias latipes	2614	hAT-1_SSa (*Salmo salar)*	67
	Xiphophorus couchianus	2756	hAT-1_SSa (*Salmo salar)*	75
	Gouania willdenowi	2789	CHAPLIN4_FR *(Takifugu rubripes)*	91
	Cottoperca gobio	2656	hAT-1_SSa (*Salmo salar)*	74
	Kryptolebias marmoratus	2673	hAT-1_SSa (*Salmo salar)*	74
	Larimichthys crocea	2787	hAT-6_XT *(Xenopus tropicalis)*	87
	Chelmon rostratus	2530	hAT-1_SSa (*Salmo salar)*	80
	Maylandia zebra	2551	hAT-1_SSa (*Salmo salar)*	87
	Salvelinus namaycush	2488	hAT-1_SSa (*Salmo salar)*	99
	Oncorhynchus kisutch	2508	hAT-1_SSa (*Salmo salar)*	98
	Salmo trutta	2893	hAT-1_SSa (*Salmo salar)*	99
	Labrus bergylta	2191	hAT-2B_PM *(Petromyzon marinus)*	96
	Labeo rohita	2528	hAT-8_PM *(Petromyzon marinus)*	72
	Paralichthys olivaceus	2255	hAT-17_SSa (*Salmo salar)*	79
	Gadus morhua	2803	hAT-22_DR *(Danio rerio)*	70
	Lates calcarifer	2788	hAT-9_DR *(Danio rerio)*	83
	Collichthys lucidus	3127	CHAPLIN8_FR *(Takifugu rubripes)*	91
	Anguilla anguilla	3562	hAT-1_SSa (*Salmo salar)*	95
	Perca flavescens	2442	hAT-1_SSa (*Salmo salar)*	80
	Parambassis ranga	2630	hAT-4_SSa (*Salmo salar)*	70
Amphibia	*Bufo bufo*	2392	hAT-1_SSa (*Salmo salar)*	93
Reptilia	*Anolis carolinensis*	2250	hAT-2_AC *(Anolis carolinensis)*	100
	Alligator mississippiensis	4120	hAT-7_AMi *(Alligator mississippiensis)*	87

Table S2: Species from the *Actinopterygii, Reptilia* and *Amphibia* class containing hAT transposons that were incorrectly annotated as GTF2IRD2/2A. The best match of each hAT transposon to the Repbase database and the corresponding pairwise identity is shown.

Table S3: 3' and 5' TIRs of TEs misannotated as GTF2IRD2/2A		
**3_TIR**	**5_TIR**	
>Sphaeramia_orbicularis	>Sphaeramia_orbicularis	
AGTTTGACACCCCTG	CACATATGTCAAACT	
>Xiphophorus_hellerii	>Xiphophorus_hellerii	
AGTTTGACACCCCTG	CCAGTGTATTGATGT	
>Archocentrus_centrarchus	>Archocentrus_centrarchus	
AGTTTGGACACCCCTG	CAGGGGTGTCCAAAC	
>Takifugu_rubripes	>Takifugu_rubripes	
AGTTTGCCCATGCCT	CAGGGGTGGGCAAAC	
>Erpetoichthys_calabaricus	>Erpetoichthys_calabaricus	
TATAAGCCTTGCTTG	CACAGGTGTCAAACT	
>Oryzias_latipes	>Oryzias_latipes	
AATTGCCCACCCCTG	CAGACCTGGGCAAAC	
>Xiphophorus_couchianus	>Xiphophorus_couchianus	
ACTTTGACACCCCTG	CACATATGTCAGAGT	
>Gouania_willdenowi	>Gouania_willdenowi	
AGTTTGACACCCCTG	CAGGGGTGTCAAACG	
>Cottoperca_gobio	>Cottoperca_gobio	
ACTTTGACACCCCTG	CACATATGTCAGAGT	
>Kryptolebias_marmoratus	>Kryptolebias_marmoratus	
ACTTTGACACCCCTG	CACATATGTCAGAGT	
>Larimichthys_crocea	>Larimichthys_crocea	
TAAAGAACCCAGCTA	CATATGTGTCAAACT	
>Chelmon_rostratus	>Chelmon_rostratus	
AGTTTGACACCCCTG	CATATGAGTCAAACT	
>Maylandia_zebra	>Maylandia_zebra	
AGTTTGACACCTATG	CAGGGGTGTCAAACT	
>Salvelinus_namaycush	>Salvelinus_namaycush	
AGTTTGACACCCCTG	CATGGGTGTCAAACT	
>Oncorhynchus_kisutch	>Oncorhynchus_kisutch	
AGTTTGACACCCCTG	CATGGGTGTCAAACT	
>Salmo_trutta	>Salmo_trutta	
AGTTTGACACCCCTG	CATGGGTGTCAAACT	
>Labrus_bergylta	>Labrus_bergylta	
AGTTTGGACACCCCT	CAGGGGTGTCCAAAG	
>Labeo_rohita	>Labeo_rohita	
AGTTTGAGACCCCTG	CAGGGGTCTCAAACT	
>Acipenser_ruthenus	>Acipenser_ruthenus	
TATTGACTACCCCTG	CAGGGATAGTCAATT	
>Paralichthys_olivaceus	>Paralichthys_olivaceus	
AGGTTCCCCACCCCT	CAGGGGTGGGGAACG	
>Gadus_morhua	>Gadus_morhua	
AATGGGGCTGCCCTG	CAGGGGTGCCCAAAT	
>Lates_calcarifer	>Lates_calcarifer	
AAGTTTGGGGACCCC	AGTGGTCCCCAAACT	

>Collichthys_lucidus	>Collichthys_lucidus	
AGTTTGAGACCCCTG	CAGGGGTCTCAAACT	
>Anguilla_anguilla	>Anguilla_anguilla	
AGATTGTAGCCCCTA	CAGGGGTGTCAAACT	
>Perca_flavescens	>Perca_flavescens	
AGTTTGACACCCTTG	CACATGTGTCAAACT	
>Parambassis_ranga	>Parambassis_ranga	
CAATGCCCAGGTCTG	CAGGGGTGGGCAATT	
>Bufo_bufo	>Bufo_bufo	
AGTTTGACACCTATG	CAGGGGTGTCAAACT	
>Anolis_carolinensis	>Anolis_carolinensis	
AGTTTGGGGACCCCTG	TCAGGGGTGCTTTGAT	
>Alligator_mississippiensis	>Alligator_mississippiensis	
AATTGCCCACCCCTG	AGCAGGGTGGGCAAAA	

Species name	5' TIR	[hAT-6]	3'TIR
Sphaeramia orbicularis	-**CA**CATA**T**GTC**AAA**C-T	----------------------	**AGTTT**GACA**CCCCT**G
Xiphophorus hellerii	-**C**CAGTG**T**ATTGATG-T	----------------------	**AGTTT**GACA**CCCCT**G
Archocentrus centrarchus	-**CA**GGGG**T**GTCC**AAA**-C	---------------------	**AGTTT**GGACA**CCCCT**G
Takifugu rubripes	-**CA**GGGG**T**GGGC**AAA**-C	---------------------	**AGTTT**GCCCATG**CCT**-
Erpetoichthys calabaricus	-**CA**CAGG**T**GTC**AAA**C-T	----------------------	TATAAGCCTTGCT**T**G
Oryzias latipes	-**CA**GACC**T**GGGC**AAA**-C	----------------------	AA**TT**GCCCA**CCCCT**G
Xiphophorus couchianus	-**CAC**ATA**T**GTCAGAG-T	----------------------	AC**TTT**GACA**CCCCT**G
Gouania willdenowi	-**CA**GGGG**T**GTC**AAA**C-G	----------------------	**AGTTT**GACA**CCCCT**G
Cottoperca gobio	-**CA**CATA**T**GTCAGAG-T	----------------------	AC**TTT**GACA**CCCCT**G
Kryptolebias marmoratus	-**CA**CATA**T**GTCAGAG-T	----------------------	AC**TTT**GACA**CCCCT**G
Larimichthys crocea	-**CA**TATG**T**GTC**AAA**C-T	----------------------	TAAAGAACCCAGC**T**A
Chelmon rostratus	-**CA**TATGAGTC**AAA**C-T	----------------------	**AGTTT**GACA**CCCCT**G
Maylandia zebra	-**CA**GGGG**T**GTC**AAA**C-T	----------------------	**AGTTT**GACACCTA**T**G
Salvelinus namaycush	-**CA**TGGG**T**GTC**AAA**C-T	----------------------	GA**TTT**GACA**CCCCT**G
Oncorhynchus kisutch	-**CA**TGGG**T**GTC**AAA**C-T	----------------------	**AGTTT**GACA**CCCCT**G
Salmo trutta	-**CA**TGGG**T**GTC**AAA**C-T	----------------------	**AGTTT**GACA**CCCCT**G
Labrus bergylta	-**CA**GGGG**T**GTCC**AAA**-G	---------------------	**AGTTT**GGACA**CCCC**T-
Labeo rohita	-**CA**GGGG**T**CTC**AAA**C-T	----------------------	**AGTTT**GAGA**CCCCT**G
Acipenser ruthenus	-**CA**GGGA**T**AGTCAAT-T	----------------------	TA**TT**GACTA**CCCCT**G
Paralichthys olivaceus	-**CA**GGGG**T**GGGGAAC-G	---------------------	AG**GTT**CCCCA**CCCCT**-
Gadus morhua	-**CA**GGGG**T**GCCC**AAA**-T	----------------------	AATGGGGCTGCCC**T**G
Lates calcarifer	--**A**GTGG**T**CCCC**AAA**CT	--------------------	AAG**TTT**GGGGA**CCCC**--
Collichthys lucidus	-**CA**GGGG**T**CTC**AAA**C-T	----------------------	**AGTTT**GAGA**CCCCT**G
Anguilla anguilla	-**CA**GGGG**T**GTC**AAA**C-T	----------------------	AGA**TT**GTAG**CCCCT**A
Perca flavescens	-**CA**CATG**T**GTC**AAA**C-T	----------------------	**AGTTT**GACACCCT**T**G
Parambassis ranga	-**CA**GGGG**T**GGGCAAT-T	----------------------	CAATGCCCAGGTC**T**G
Bufo bufo	-**CA**GGGG**T**GTC**AAA**C-T	----------------------	**AGTTT**GACACCTA**T**G
Anolis carolinensis	T**CA**GGGG**T**GCTTTGA-T	---------------------	**AGTTT**GGGGA**CCCCT**G
Alligator mississippiensis	AG**CA**GGG**T**GGGC**AAA**-A	----------------------	AA**TT**GCCCA**CCCCT**G

Table S4: Multiple alignment of the 5' and 3' TIRs of *Actinopterygii*, *Amphibia* and *Reptilia* hAT transposons derived from sequences misannotated as GTF2IRD2/2A. Nucleotides in bold text are conserved in most of the species.

Species Name	Predicted Sequence	Repbase Best Match	Frequency
Danio rerio	Zinc finger BED domain-containing protein 1-like (ZBED1)	hAT-30_DR	1
	Ribosome-binding protein 1-like	MSAT-3_DR	1
	Uncharacterized	Helitron-3_DR	13
		ISL2EU-1_DR	
Thamnophis sirtalis	General transcription factor II-I repeat domain-containing protein 2-like	hAT-11_CPB	1
	Zinc finger, BED-type containing 4 (ZBED4)	hAT-6_DR	3
Anolis carolinensis	Uncharacterized	Neptune-3_ACar	4
Xenopus tropicalis	Fibroin heavy chain-like	Helitron-N4_AmRa	1
	Coiled-coil domain containing 51 (ccdc51)	Kolobok-2_XT	4
	Uncharacterized	BEL-10_XT-I	
		Polinton-2_XT	13
		Kolobok-1_XT	
Gallus gallus	Uncharacterized	ERV2-10_GG-I	3

Table S5: A sample of TEs from vertebrates which were incorrectly annotated as various other proteins and uncharacterised loci. The best TE match in Repbase was found for each predicted sequence. Frequency refers to the number of times the predicted sequence overlapped with a TE from the top 25 hits for each species.

Table S6: Nucleotide sequences TEs misannotated as GTF2IRD2/2A

>R_NC_043968.1:53464292-53467595_Sphaeramia_orbicularis_chromosome_12,_fSphaOr1.1,_whole_genome_shotgun_sequence

ATGTTGATCACATATGTCAAACTCGGGCCCACGGGCCAAATTTGGCCCGCAGTGTAATTATAATTGGCCCGCGAGATCATATCAAATGTGCATTAGAACTGGCCCGCGGTATATACTGTACCGCTAATACTACAAATCCCACAATGCTTTGCTAGTATGTT
GGCGCGCAGTCGAAACCGGCAAGCGCTCCTTTTTTCTGTTGACAGTCATTGACAACCATGCTCCAGTCACATCAGACAAATTAATTTCACCCTCCACAAAAATGGCCAAACGAAAGATGGACAACAGGAGCTTTCAAGACAGGTGGGAGGCAGATTAT
CTGTTCACGAATATAAAGGACAGACCTGTTTGTCTTGTGTGCGGAGCTAACGTGGCTGTAACGAAAGAATATAACATAAGAAGACACTATGAAACAAAACATTATGAAAAGTACAAGGACCTGGACGTAAAGCAGAAGCTGCAGAAGGTGGAGGAGATG
AAAAGAAGTCTGGTTTGCCGGCAGACTATGTTCATGAAAGCAAAATCAAAAAGTGAAGCTGCTGTAAAGGCCAGCTTTATTGTGGCAGCAGAGATAGCAAAATCAGCCCGGCCCTTTAATGAGGGACAGTTTGTCAAAAAGTGCATGGTCAAAGTTTG
CGACATCGTGTACCCAGATAAAAAGCAAGAATTTTTAAATGTGAGCCTGAGCAGAAACACTGTGGCTGAGCGCGTGTGTGAGCTTTCCACTAATCTACATGAACAACTAATAAAAAAGGGAAAAGATTTTATTGCATACTCCCTTGGTGTGGATGAGAGC
AGCGACACGTCTGATACTGCCCAGCTGTCAATCTTCATCCGTGGAGTAGACTCGAGCCTGTGCGTCACAGAGGAACTTTTGGGATTAAAATCAATACATGGCACCACCACAGGAAAAGATATCTTTGAAGAGGTATCCAAATGTATAACTGAAATGAAC
CTGCCTTGGGATAAACTTGTGGGACTGACGACAGATGGTGTGCCCGTGATGTGCGGTCAAAAGAGTGGACTGGTCGGCAGGATTCAGGAGAAGATGCGGGAGGAAGATGCAGGTGAACTTACAGTTTATCACTGTATCATACATCAGGAAGCGCTC
TGTGGCAAAGCTCTGCAAATGGAACATGTTATGAGCTCTACAAAACGAGTAGTCAACTTCATAAGAGCCAAAGGTTTGAATCACCGGCAGTTCAAGTCTTTTCTGGAGGAGTTGGATTCGGAATACAGAGATATGCCCTATCACACAGAGGTGAGATGG
CTAAGCAGAGGAAAAGTACTGAACAGATGTTTTGAGCTACATGAGGAAATCTGTCAGTTTATGGAAAACAAAGGGAAGGACACAACAGAGCTGAGAGATAAAAAGTTTCTGTGTGAGCTTGCATTTCTGTCTGACATTGTGAGCCATCTCGATGTGCTT
AACCTGCAGCTTCAGGGACGGGGCCACATCATCACAGACATGTACGCAGCAGTGAGGGCCTTTAAAACTAAGTTGTGTCTGTGGAAGACTCAGATGCTGCAACGAAACTTGGGTCATTTTCCGTGCTGCCAAACCATGGCAGAGCAAATTTCTCCTG
CCGTGCTGCCTAGCACACAGTTTGCTGAAAAAATCAACGCGCTCAGCGCCGAGTTTTCCCGGCGATTTGCCAACTTTGAGGCTCAGAAAGGGAGATTTGAACTGCTCAGTAACCCGTTTGCGATCGACATGGAAAGCGCACCAACCAGCCTCCAAA
TGGAGCTAATTGAACTCCAATGTAGTGACATGCTCAAATCAAAATATGACTCTGTTGGTGCTGCCCGGTTTCCATGTTTCCTCCCCGACACAATGCCTCAACTAGAAGCACTCGGAGAGCGCAGACCTCCGCCAAGGCTGATCAGTGGCCCCCCCCG
TGGGCCCCCCCGCGCCAAGGAGGTTATGTTTTTGCCAGGGTTTGTTTGTCTGTCTGTCTGTTTGTCTGCCCGTTAGTGTGCAACATAACTCAAAAAGTTATGGACAGATTTTGATGAAATTTTCTGGTCTGCGCCCCCCCCCCCCGTGGGCCCCCCCAC
CCCCGATCACCACCAAAATTTAATCATTTCTTCCTTATCCCATTTCCAACAAACCCTGAAAATTTCATCAAAATCTGTCCATAAGTTTTTGAGTTAAGTTGCACACTAACGGACAGACAAACAGACAAACAAACCTTGGCAAAAACAACCTCCTTGGCGGA
GGTAATTACGCACCCAAGCTGCTCAGATGCTCTCAATGTTCGGCAGCACATATCTATGTGAACAACTGTTCTCTATGATGAAGGTGAACAAAACACCTCACAGAAGTCGGCTCACTGATGAACACCTTCATGCCGTCCTGAGGTTTTCCTCAGCTCAGA
GCCTGACCCCAGACATCGATGAACTTGCATCTAAGAAAAGATGTCAGGTATCTGGCTTAGACCAATGTGCAGAGTAAATCAGAGTGTAGCAAACTGAGCTTGAATATATCTGTTTCTTATGCACTTTTTCTACTGCAAGGCATGGTCTGTTAATAGTTGAA
ATGTTGGATTTTTATTGAATGACATTATTATTTTTGGTTCTTTTGTTGTTGGCTTTGAAAAAGATTTACTTTAAAAAGGAACTTGAAAGGATACGGGTAGAGACATACTTTATTTATTTATTTATTTATTTAAATAAGAAATGAATACCACTGATGTGTCTTTTATTT
CAAATTTAATTTCTCATGTGTTTGTAATATCAAGCTTTGGTTGTTCCAGATTCTGTGTTTAAGCAAAACTAAAGTTTGTTTCCATATGAAAAGGTTAAACATTACATATCAGTTGCAGTTAATTTTTCAATAAATATTGAGTTTGGCCCGTGACTTTACTTCACT
GCTTAATTTTGGCCCACTGTGAATTTGAGTTTGACACCCCTGATGTTGAT

>NC_045692.1:1086575-1090260_Xiphophorus_hellerii_strain_12219_chromosome_21,_Xiphophorus_hellerii-4.1,_whole_genome_shotgun_sequence

TTCTAAGTCCAGTGTATTGATGTGAGCTGTGGTTATCCAGACAGATGGGGCATCTTGAAGCACAGTCACTCCCTGAATTGAAGCACTAATCACATCCTGAGTCACTTCTTCTGTACATTGCTGCAATAAGTCATTTATGTCCAGTGGCATGCGAGAGAGG
CCATCTGCGTCTGTATTGTGTTTGCCTGGTCGATATTTAATGGTGAAATTGAAATCTGCAAGTTGGGCAACCCACCTGTGACCTGTGGCATTCAACTTGGCTGTGGTGAGAATGTAGGTCAGGGGGTTATTATCTGTATATACAGTGAAATGAGGTGCAT
GGTACAAATAATCTCTAAACCTTTCACATATTGCCCACTTGAGTGCTAGAAATTCCAATTTTCCTGAATGCATGTGATAATTTTTCTCAGGTGGGGTCAGTGTTCTAGATCACATGTGTCAAACTCAAGGCCCGCAGGCCAGATGTGGCCCGCCACGTCA
TTTTATGTGGCCCGCGAGAGTGTCCGTGTTGAGTATCTAAAAATAAATACAGAAAAATAAATTAAGAGCGTGCTTTGACCAAAAACTACATTTCCCACAATGCCTTTCTACCTCGGTAGCCGCGGCAGTGTTTCCACATCCATGCCAACAAGTGGAATTG
AGATATCAATAATGATCCCAAAATGTCCAGAAAGAGAAAAGTTGATGCAGACGGAAGACTGTTTCAAGAAAGATGGAAAGGCGAATACTTGTTTGTGCTTCAAGGAGAAAGACCAGTATGTCTTTTGTGTTACGAGGCGGTGTCGGTTGTCAAAGAGTA
CAATCTGCGTCGACATTTTGACACCAAACACGGAGCTAAGTATGCTAAAGCTAGCCTTCAAGAAAAGCAACAAATTGCCCAAGAATTAAAAGGCAAACTGCGGTTGCAGCAGAGTTTGTTCACAAAATCCACAGCCAAAAACGAGGCTGCAGTGAAAG
CTAGCTTCATTGTGGCTGAAGAAATCGCCCACGCTTCCAAGTCTTTTTCAGAGGGAGCGTTTTTGAAGCAGTGCATGCTGAAGGTCTGTGAGCAGGTGTGTCCCGACCAGTTACAGACTTTTAGAAATGTCAGTTTATCAAGAAACACCATCGCGAAC
CGAGTGAAGGAACTAGCAGAAAATCTTACAACACAGCTGACCGAGGAGACACGCAGCTACACAGCTTTTTCATTAGCTGTTGATGAAAGTACAGATAACACAGACACAGCGCAGTTATCAATTTTTATTAGAGGCGTAAAGTCGGACCTCTCTATCACT
GAGGAGCTGTTGGATGTGGCTGCAATGCATGGGACCACGACGGGACAGGACATTTTTGATGCGGTGGAGAAGTCCGTAAGTAAAAATGCATTGCAGTGGGAAAACTTGGTGGGATTAACTACAGACGGTGCACCGGCAATGTGTGGAGGAAAAGTG
GGCTTGGTTGGACTAATGAAGAGGAAAATGCAAGAAATGAACTGTCACACGCCTCTGATAACATATCATTGCATTATCCATCAAGAAGCTTTGTGTGGGAAGGTGCTGGGGATGGAGGACATTGTGACCACAGTCATGAAAACAGTGAACTTCATTCGG
GCGCGGGGCCTGAACCACCGTCAGTTCCAGCAGTTTTTGCTGGAGATGGGCTCGGAACACGGGGATGTGCCGTACCATACAGAAGTGCGGTGGCTGAGTAGGAGCAAAGTCCTCAAGCGATTTTTTGAGCTCAGGGAAGACATTGCTATTTTTATG
CAGAGTAAAGGAAAACTCATGTCTGAACTATCAGATCCAAAGTGGCTGTGTGACTTTGCTGTGCTGTGTGACATAACCGACCATCTCGCCCAGCTGAATCAGAAGCTGCAGGGACGCAAACAAGTCATCACGCAGATGTCCGACACGATCACGGCTT
TCCAGCGTAAATTGGACTTATGGATGTGGCAAGTGAAACAGGACAATCTTGTCCACTTTCCTGTTTGTCAGAGCATGTCAACCTCGTTTCCCGAGACTTTTTCATGTGCTCAGTTAGCTACCAAACTGAGCTGGTTAAAGACTGAGTTTGATCGGCGCT
TCTCTGACTTCAGAGCACAACAGTCAGGCTTTAACATCTTTGCCAATCCTTTCACCACCGATGTCTGCACTGCACCCCAACACCTTCAGATGGAGCTAATTGAGCTTCAAAGCGACAGTGGCCTGAGAGCAAAGTTCCAGGATGCTGCAATCCAGGAC
TTTTACCGTCTGATGCCCCCTGGTTTGATGCCACAGCTTCGACTTCATGATGCCCGTGTTCTGTCCATGTTTGGAAGCACCTATTTGTGCGAACAGTTTTTTTCCATAATGAATCTGAACAAAAACAAGCACAGATCACGCCTCACGGATGACAACCTCC
ATGCTGTTCTACGCATTGCCTCAGCACAGGACCTAAAGCCAGACATTGACACACTGGCAACGGAAAAACGGTGTCAGACATCTGGTCAGAAAAACACAGGTTAGCATTTTCTTTTTGAAAACTAAAATATAAGAAAATGTAATTCAACCAAGAAGAAGAA
CAGTTCAACTGTGTGCAATTTAATGATTGTGTTTGCATTTTGTTTTGTGTTTATCATTTTCAGAACATGATCAACGATAGTAGAGAGAGAATCCACTTGGTGTGTTCAAGGACAGGCAGCATCTCCTCATCAAAGACTAACCAAAAAACTTGAGTAATATAG
TTGTGAACTGAATCAACCTTTATTTTGGTATTTTTAGTTGATTATATATTATTTATGTATAGCTGCTGTTGTAATTATTTAGTGTTTGCAGGTCTTATGTGTGTATGCAGGCAAATTGTTGTCATCAAACCATCAGTTGGATGCAAGGCTGTGTGTAGCCTTTTT
TTGTAAAATGCTACATGTGTCATACATGTTCTTTGCAGCTCAGTTTTAGGATGTCTTCAAAGCTATTTGACTACTTGTTATTTTTCTTTATTCACTTGGACAGTTTGATCCTTCATTAATGGAGTTATGTGGGTTTTCATAAGCTGACAAAATTTAAAAGGATTT
ATGTTAGAGCAACAAGCAAACATATGTTTTCTATGCAACTGTAATTGTCACTTGAATAAGTTATAATAAATAATGAGTTATTTAACATTAAGGAGTAGTTACATTTAGTTACATTTATAAGTTACATCTGGCCCTTTAAGGACAGCCATTATGCTGATGTGGCCC
TCGGTGAAAATGAGTTTGACACCCCTGTTCTAGAT

>NC_044366.1:27698981-27702214_Archocentrus_centrarchus_isolate_MPI-CPG_fArcCen1_chromosome_21,_fArcCen1,_whole_genome_shotgun_sequence

ATCTAAGGCAGGGGTGTCCAAACTTTTTGGACAGAGGGCCACACATGGAAAAATATACAAAGGCCGGGCCACTTAGTTCAGGACAAATTTTATATATCACAAGTTATTAAACCAAACACATGTGAATAATCATTGACATTGCCTGGATCACAGTTTTTTAT
TAATGATTTTTCAATTTTTCATTTATTCTGCTGCTTTACAGGTCTTTTCACTGTTGGCAGCCTCACATTGCTTCTTACTCGGGATCATGATTCCCTCTTCACACCCCTGTATAAAAATGGGGAAATATAAGGGGGAAAGAGGAAGGGTATATGTGAGCTTGT
TATGCAAATATGTATGTGGGCTTGTTAACAGATTTATGTTTGTTGCAAAATGTTATCAACTTAAATTGACTGAATTTATAAATTAATTACTTATTTACACATGATTATTTAATAATGTGTATTTTTACCTCACCTAAAATGGGAACAGTGCTGCACTCAGTGGGAG
CAGTGATGCTGTGCTTTGGCCTGAAGGATGGCTGGCAGATCAGGAGTAAGTTTGGTGGTTGAGATGCGGAGGACATCACAGAGATGGCTGTCAGTCATTTTGGTTCTCAGTCTGCTTTTGTTCAGAGTTAATAATGAAAATGTTTGCTCACATATGTAT
GTGGAGCCAAACAGACTCATCATTCTTTTTGCATGGCGTCTCATCAGAGGAAACTTGTCCTTTTCCACCCTCCGATAGAAGTCAGGTAGGGACAAAAGCTGGTGTTGATTTCTCAGAGAATCATCACACTGCATATCAATAAGTTTTAATTGCAGACTCT
CTTCCACTCCCTCTGCATCCACCAGGAAGGGAGTCGAGAACAGTTTGATTTGTTTTTCAATATTAGAGAAATCTTGGAAGCGCTGACTGAATTCTGTCATCAGAGATGTGATAACAGACAAATATTTCCCTTTCTTAAGAGAAAGATTGGCCTTTGGAAAA
GCACTCTTGATTTCTGACAGCGTGGGGAAGTGCGCAAAATTAAAGTTGCGTAACTGTGCCTCAAAGAGACGGAGTTTCACGCAGAATGCCTTCATCTGCGTATAAAGTTGTGGTACAAGCTGGTCTTTGCCTTGTAGGCTCTTGTTAAGTGAGTTAAGA
TGATCGGTAAGATCAACTAAAAATGCTAGGTCGGCCAACCATAAAGGATCACTCAGCTCATGAAGCGGGCGGTCCTTTTCTTTCAAAAACTGGTCAATTTCCGACCTCAGGGCATAAAACCGCTGCAGCACGGAGCCGCGACTTAGCCAGCGCACAT
CACAATGGTACAGCAGGTCTCCGTATTCAGCATCGATGTCTGATAGGAACGCTTGAAATTCTCTGTGGTACAGCCCTCGTGCTCGAATTATGTTTACCGTTTTTACAACAGTGTTCATCACATCGCCGAGCTGGACTGTCTTGGAGCAGAGGGCTTCTT
GGTGAATGATACAATGCATTTTCACAGCCTCACCTCCACTCTCTTTAACCTTGGCACACACCATAGAGGCCATTCCTTTGCGCTCTCCGGTCATAGCTGGGGCTCCATCCGTTGTAACTCCACACAACTTGTCCCATTTAAGTCCAATGTTGTCAACTGC
ATCTGATACCGCTTCAAAAATGTCCTTACCCGTCGTTGTGCCCTTTAGACTTCTAAGATCAAGCAGCTCCTCCGTGATGCAAAAATTATCATCAACTCCCCGCAAAAAAATTAACAGCTGTGCGGTGTCTGTGGCATCTGTGCTCTCATCACATGCTATC
GAGTAAAAATCAAAAGCGCAAGCTTTATCTGACATTTGATGTTTTAAGTCAGCAGACAAGTCTTCTATTCGCCGCACAACTGTGTTTCTGGACATGCTCACGCTGCTGAATTCCTGCAGTTTTTCCGGGCACATTATTCCTGCAACTTTAGTGAGGCACT
GTTTTATGAAGTCTCCATCTGAGAAAGGTTTCCCGTGTTGTGCAATGAGTTGAGCTATCTCGTAGCTAGCCACTGTGGCATTTTCTTGTGCCTTATTAGCACGAAAAAAATACTGTTGCTGAGCTAACAGGCTGGCTGCCATTTGCTTAACTTTATGTTCT
CGTTCGGCACCAGTGTAGGATGCATACGTCTGATGTTTGGTTTCATAGTGTCTTCGTAAATTATACTCTTTCATCACTGCCACAGTCTCAGTACAAATCAAGCAGACACACTTCCCTTTCACTTCTATAAAAAAGTATTCATTTGCCCACCGTGTCTGAAAT
CTTCTGCATTCACGCGCTACCTTACGTTTCTTCATAGACGCCATTTTTTGTCTGCCCTGACATTTTTCTTCACTTATCTTGTTTTGTTGAGAAACTGCCTTGTTCAAGATAGTAGCTCCACCTAGTGTTAAAAAAGTGCAGTGCAGTCAAGCAGAAATTTGA
CGCTTGGGCCATATTGTATTATACTTATTTTAATTTGCTGCGGGCCAATTAAAAATGGACGGCGGGCCGCAGTTGGCCCGCGGGCCGTAGTTTGGACACCCCTGATCTAAGG

>NC_042287.1:1903615-1907853_Takifugu_rubripes_chromosome_3,_fTakRub1.2,_whole_genome_shotgun_sequence

GGGTTAGAACAGGGGTGGGCAAACATTTTGATGCGCGGGCCACAATGGGTTCTAACTTTTGACAAAGGGGCCGGACCAGGAGCAGATGGATGGATGGAGTGCTTTGGTAACCTCACCTCATTGGAGAAAAAATACACATCTTGGGATATGGAGAAAA
CATGTGCTTTAATTTAAAATGAAAATGAAGAGAAGCATTATAACAAAATATCTGAATTTGGAATGAGTCTTAAAACAATTCAAATAAAATGAATAAAATGTGCCTTTTTTTTAAAAAAAATAAAAAAATTCTATTTTAAAGCACTGAAAGTTCTGTTATCCTACCA
TGGTGCCAAAAGTCAACAATGAACAGTGCAAGGAGGAAACAGTGGCAACAAGTAAGTCTGTGAAGCGGCATTATCTGCGTGCGCATCAGCGAGACACTTTCCATTTAATGGGAACAGTGTTGTTGCTCTCCTTTTTTAGCCAGCACATCAAAGTCCG
GAGTTAGTTTTGTTGTGGCGATTCTCAGAATGGATCTCAGGTGTTGGTCTGTTAACCTGGATCTGTGGCTGGCTTTGTTGATGTTCATGATGCTGAAGGTCTGTTCACACACATAGGTCGAGCCGAACAATGCCAGGATCTTCTGCGCTGTCTGCCGG
AGCCTTGGAAACGCTGCTTTGCTGAGTGACGCATAGAAGTCATTTAGTTTCAGACAGTTGAACTTCCCTTTTAAGACTGGGTCGGACTGAAGGTCGATCAGTTCGAGCTGCAGCTCCTCTGGGGCTGTTTCGGGGTCTTGTGACAGAGGATAGGCCA
CCAACTGAAGTGACTTGTCAATTTTGTCGAAATCCGAGAACCGACGGCAGAATTCTCCGTGCAGTGCATCCAGTGACTCACTGTATTTCTTCACTTTTGCTCCGGCCTCTTTCAGCGTGGCTAGTGTAGGAAAATGAGTGAATGACTTGTTCAAAATTT
GCCTGGAGAATATAGCCAGCTTGGATTTGAAGGCTTTCACGTTTGCGTACATGTCGTGCACAAACTGATCCTTCCCCTGTAGTTTCGTGTTGAGCTCATTTAAGTGTGAAAATACGTCCGTAGCGAACGCAAAGTCACAAATCCATCTCTTGTTGCTCA
GCTCAGGAAATTCATCAGCCTTCCCCAGTTGCTCCAAAAACAAGACAATCTCCTCTTTGAGCTCCCACACGCGTTGAAACACTTTGCCCAAACTCAACCAGCGAACACGGGAGTGATAAAGCAGGTCCTGGTGATCCGCATCAGTTTCCTCCAGGAA
CGCAATAAACTGACGGTGTTGAAGTCCCCTTGCCCGTATGAAGTTGACGAGTTTCACGACAGGATTCACAACATGGTTCAGCTGTAATACCGATTTACATAGAGATTCCTGGTGGATGATGCAGTGGAGGAAAATGACATCCTGGTCAGGGTTTTCATC
TTTCACTTTATTCTGGATTCTCCTCAGCAGGCCAACGTTTTTCCCTGTTAAATTTGGAGATCCATCTGTGGTGACGTTGACAAGCTTACTCCAAGGAAGCTTCGCATTTTCGATGACAGCAGACACCTGGTCAAACAAGTCCTCTCCCCGTGTTTGTCC
TTTCAGAGACTCCATTGTTAAAAACTCCTCCGTTATTTCAAAAGTGTCGTTGATACCTCGGATAAAAATGAGAAGCTGAGCAGTGTCTTTGACATCGTTGCTTTCATCCAGTGCTAAAGAATACAACGTGAATGACTCCGTCTTTTTATTTAAGCTGCTGAT
GATATCTTCATCTATTAGTTCCACGCGGCGAGTCACTGTCCGCCGTGATAAGCTGATTTTCTCAAATTTGTCTTTGCTCTCCGGGCACAAAATACCTGCTGTCTCCACCATGCATTCTTTTAAAAACTCGCCCTCAGACAGAGCTTTGCTGGCCTTCGCT
AGTCTGAATGAAAGCATAAAACTGGCCTTGGTAATTGACTCTTGAATGGCAGTTTGACGGTGAAAGATACTTTGTTGAGCCTTTAAATTAGCTGCCAACTTCTGAGCAGCAGATTCCCGTTCTTTTTTTGACAGCTTGCTAGCATAGTTAGCATGCTTTGT
GGCAAAGTGTCGGCTAATATTATACTCTTTAAAAACCGCCACAGTTTCTTGGCATATGAGGCAAACAGCAGATGATCGGTGTTCAGTGAAAAAATATTTAGTTGTCCACTCCTTATTGAACACTCGACATTCTGTGTCCACTTTCCGTTTCTTAGGTCCGC
TCATCTTTATAGAAGGGCTGATGGGTCAGAGGATAAAGTGCAAACGTGAAGTAGAAAAGTGTTTATGTAATGTATCGATTGTTTGTATAAAGTTTTATACAGCGATCGTAATGACAGAATACCGTAAAATGCGCTGAGGTTGAAATACGAGGAGAGGGAGT
GACGTAAATTCACACATGCGCAGTACAAATCGTGTTTCCGGGTACAGATCGAGTAGTGTACAGTGCGCCACCTAGTGGGAAAACACCAGAATTGCAGTGAAAATATAACATTAACAAGGTTTATTCATTTAATTTTTCAAGTTTGGCGGGCCGGATTAAAA
CGTTTAACGGGCCGGGTTAGGCCCGCGGGCCGTAGTTTGCCCATGCCTGGGTTAGAA

>NC_041409.1:27368371-27371176_Erpetoichthys_calabaricus_chromosome_16,_fErpCal1.1,_whole_genome_shotgun_sequence

TCTTAAGTCACAGGTGTCAAACTCTGGCCCGCGGGCCAAATTTGGCCCGCCGTGTAATTTCATTTGGCCCGTGGGGCAATATCAAATTAACATTAGAGCTGGCCCACCGGTATTATACAGCGGCGGTGTTGCTGTAACACCGCATTCACTGCGAATACT
ACAAATCCCATAATGCTCTGCGGTTGTTTTGGCGCGCCAGCTTTGTGTCAGTAGATTTCTAGCAAGCAGCGTTCTTTCCAGACGTGCTGCGGCCCAATACTACAACTCTAGGTGTCACTGTTTTACCCCTACCAAAAATGACGAAAAGGAAGGGAGAA
AACAGAACTTTTCGGGACAGGTGGGAGACAGAATATATGTTTACGTATCTGAAAGACAGACCTGTTTGTCTCGTTTGTGGAGCCAACGTGTCGGTACCTAAGGAGTACAACCTTAGGCGACACTATGAAACGAAACATCAGGACATGTACAAGGACCT
GGACACGACTCAAAGGAGCCAGAAAGTAGAGGAGATGAAGAGAGGGTTGGTTTCACAACAAAATATGTTCAAAAAAGCCACAGCACAAAACGAGGCTGCTGTAAAGGCGAGTTATATCGTGGCAGAAGAAATCGCCAAATCAACCCGATCCTTTAATG
AGGGAGAATTTATTAAAAAGATAATGCTGAAAGTTTGTGACCAAGTGTGCCCAGAGAAAAAACAGGCCTTTTCAAACGTAAGCCTGAGCAGGAACACAATAGCGGAGCGCATATGTGATCTTGCCACCAATCTGCATGACCAGCTGATGGAAAAGGGG
AAAGATTTTGTTTCTTTTTCCCTCGCTGTGGACGAGAGCACTGACGCGTCTGATACTGCGCAGCTGTCAGTCTTCATCCGCGGTGTCGACTCAAAAATGTGTGTTACGGAGGAGCTTTTGGGATTTAAATCCATGCATGGCACAACCACAGGGACGGA
CATTTTTGAGGAGGTTTGCAAATGTGTAACTGAAATAAACCTGCCGTGGGATAAACTCGTGGGATTAACCACTGATGGTGCGCCAGCGATGAGTGGTAAAAAGAATGGTCTGGTGGGCAGGATGCGTGAAAAGATGCGGGAAGAGAACTGTGCAGGT
GAGCTATCGGTTTATCACTGCATTATACATCAAGAGTCACTGTGTGCTAAAGCCCTAAAGATGGAACATGTTATGACCACAGTAACACAAGTTGTTAACTTTATAAGAGCCAAAGGTCTGAATCACCGCGAGTTTAAGTCTTTTCTGGAAGAGTTTGGTTC
GGAACACACAGACGTGCCGTATCACACAGAGGTGAGATGGTTAAGCCGCGGAAAAGTACTGAACAGATTTTTTGAGCTGCGTGAAGAAATATGTCCGTTTCTGCAAAGCAAAGGGAAGGACACAGCAGAACTCCAGGAGCAAAAGTTTATGTGTGAG
CTGGCCTTTCTGTCTGACATCGCAAGCCATCTTGATGCGCTGAACCTGCAGCTACAGGGGCGGGGCCACATCATCACCGAGATGTACTCGTCAGTGAAAGCTTTTAAAGCTAAACTCTGCCTGTGGGAGAATCAGCTGCTGCAAGGAAACCTTGGCC
ATTTCCCCTGTTGCCAAACCATAAATACGCAGATCTCTACCGCCGTGTGCGCACAGTTTGCTGAAAAACTCAGTGTACTCGGCGCTGAGTTTAACCGACGATTTGGCGACTTTGATGGTCAGAAATGGAGATTTGAACTGCTTAGTAATCCCTTCGCAG
TCGATGTGGAAAAAGCACCAACTAACATTCAAATGGAGCTGATTGAACTCCAGTGTGATGACACGCTGAAGTCAAAGTATGATGCTGTTGGCGCCGCACAGTTTCCACAACACATCCCTGACACAATGCCTCAGCTCCGCACCCAAGCTGCTCAATTG
CTCTCCATGTTCGGCAGCACTTACCTATGTGAGCAACTTTTCTCCTCAATGAAGATGACGAAAACATCTCACAGGGCACGTCTCACTGATGAACACCTTCGTTCGATAATGAAGGTTGCCTCAGCTCAAAGCCTGAGCCCCGACACTGATGAACTAGC
ATCCAAGAAAAGATGCCAGGTGTCTGGCTTGAACACACCATGTTAGATCAGTGTGTCGCAAACTGAACAGTAAAAAGGCCTGAATGATTGATTTTTGTATTAGCCAGTGGAAAAAGTTAATGTTGATATTTAGCAAATTGAAAAGAGGCATTCATTTTTTTAT
TTAAATGTTATTTAATATGCCATTGATGTTTCTTCGTTTGTTCTTTGACAGTTGATTTTGCACTATTAAGTTAAGTTAAGCTATAAGCATGGCTTGTTCCATATTCAGTGTTAAAGCAAATCAGTGTAGCAAACTGAGCAAAAACATTTTATTCATGCACTCTTC
TCTTGCTACTTCAAGGCTTGAATGTTTGATTCATTCATTATTGTTATTTTGTTTTCAAATGTATTTATTAGCCTGTGGAAAAAAGTTTATTTTGATATTTACCTCAGAAGGCTGCAAATAGAAAAGAGGCATTCAATTTTTATATTTAAACAAGAAACTCGGGTTT
GCATGTCATTATAAAGTTATATAAGCCTTGCTTGTTCAATTT

>NC_019865.2:1536826-1540972_Oryzias_latipes_chromosome_7,_ASM223467v1

CTCTACATCAGACCTGGGCAAACTACGGCCCGCGGGCAACATCCGGCCCTTCGAACGCCTCTGTCCGGCCTGCAAGAGGCCCGTCATAAAGAACATAAGTCAAACAAACATCTGCCGTCCTTTTGCTTTTATTTTGAAATGTGGACTTTTGTTTTGAA
AAGCATTGCTTTACGACAGTTGCGCATACGTGAGTGAATGTCAACAGCAACAAATGATGCGAGCCAGCTCACCCTCAAGATGTCAGCACACTCTAAGAAAAGAAAAATTGATAAGGAATGTCGTGTTTTCAAAAAGACAAATATTTCTTCACAGAAGTTA
AAGGTGAAGCAGTGTGCTTAATCTGTGGTGCGAAGGTCGCTGTGTTTAAAGATTATAATTTGAATCGACACTACACGACTAAGCACGAAGAGAAATACAGAAATCTGTCTGATGAAGAACGTGCAAAGGAGTCTGAAGCTTTGCTCCAAAAACTCCAAA
CACAACAGGGACTTTTGACAAAACTTCACGCACCCAGAAATGCAGCCGTCAGGACAAGTTTTGTAATTTCTCACAAAATTGCGCGGAACAGTAAGCCGTTTTCTGATGGAGAGTTTGTAAAGGAGTGTTTACTGGACTCTGTTGCGCTGATATGCCCG
GAGACAAAGAGCGCGTTTGAGAACATTTCACTGTCCCGACGCACGGTGGCGAGGCGGATTGAGGACATCGCGGGAAACTTGGAGCTTCAGCTGAAGAGCAGAGCGGCTGACTTTGACTGTTTCTCTCTGGCGTTGGATGAGAGCTGCGATATACG
TGACACCGCCCAGCTACTCATCTTCTTACGTGGGATAACTGCAGAGTTTCAAATCACGGAGGAGCTGGCAGCCATGCGATCAATGAAAGGGACAACCACTGGACAGGACTTGTTCAGAGAGGTAAATGCATGTTTGGACATGTTGGAACTGAAGTGG
GACAAACTTGTAGGTGTGACAACAGATGGCTGTCCAAATCTGACTGGAAAAAATGTTGGACTTTTAAAGAGAATGCAGGATAAAATGACAGAAATTGTGCAGAAGTTGACATTTTTGCATTGTATTATACATCAAGAGGTGCTGTGTAAGACAGTGTTAAA
AATGAAACACGTTGTTGATGTTGTCACCAAAACAGTTAACTTCATCAGAGCGAGAGCATTAAATCACAGACAATTTGTTGCATTTTTGGAGGAAAATGAGATTGAACATGGTGACATAAGATATCACTGCACTGTTAGGTGGCTCAGCCTTGGCAAGGTG
CTGAAAAGTGTCTGGGACCTGAGAGGCCAGATTCAGGATTTCTGTGTTGACAAAGGTCATGACGTCCCAGAACTTTCAGATGAAGACTGGGTGGCAGACCTTGGATTCGCTGTGGATGTGACTGCACTAATGAACCAACTAAATGTCAAACTGCAGT
CCAAGGGGCTTTTTGTGCATGAGATGTACAGTGCAGTGAAGGCTTTTATGAGAAAGTTGCAGTTTCTCTCAAGCCAAGTGAAGGACAATAATCTGACACACTTGCCAACACTAAAGGAAGCCAAAAGACCAGAGGGTCACCTTCAAAAGTACTCAACC
ATGCTAGAAGCACTGCATGCAGAGTTTTCAAGGCGATTTCAAGATTTCAAAACTCTTGAGAGTGAAATGCATGTGGTTTCTTCTCCCTTCAACTGCATTGTGGATGATGCACCAAGTGAAGTTCAGATGGAACTCATTGAGCTGCAGTCTGATATGCTCC
TGGCAGAGCGTTTCAGGTCAGTGTCACTGCTGGACTTCTACTCCTCCCTTAAAAAGGAGAACTTTCCACACCTGAGGAGGCATGCTCAGAAGATCCTGGTCCTCTTTGGCTCTACCTACCATTGTGAACAGGCATTTTCAGTTATGAAGTTCAACAAAT
CCAAACACAGATCCTCTGTGACTGATGACCACCTCTCAGCTGTTCTTCGCATTGCCACTTCAGACATTCAGCCAGATTTTTGTGCCCTTGTTCAAGCCCAGAACAGACTGGATTATTCTCACTGAAGAGGTAACATGGATGGAAAACATTAAAAGTTATC
GTTTGTTCCACAATACATATGAATACTGATGTGTTGCTCTATTTCTATAAATTTGTTAAGTTTGTTGTTGTTCATTTTTTGGGGAGATTAACAAGTTAAAAACTGATAACTGTGATGGTATGTTCCCCCTCTCAGGTTGCTATGTTTTCATTTTCAAGTCAAGC
CAAGTCACATCTAGTCAAGTCAGTTCTTAGCTTTGTCAAGTCTTAGTTCATGTTTCTGCTTTGGTTTTGCTTGTCCTGCCCTGCAGTGCCTTTTGTTTATTTAAATAAACCTTTAGTTTTTTGGAACCACCTGCCTCCTGTGGCATTTGCACAGATTCAATA
ATTGTTTTTGTTTTCCTTTTTGACCTATAAACCACAATTTTACATACCCTGATATAAACATTTACACTTTACAGAATGCATTATTCTTCTGAATATCAGTAACAGCTATTAAAAATATTTACTTTAAAAGCATTTTACAACGGAAGAAAGTTAGTGGCCCTCTTGC
ACAATTCAGGTTTCTGATGTGGCCCCCGGCAAAAATTAATTGCCCACCCCTGCTCTACAT

>NC_040228.1:12311419-12316516_Xiphophorus_couchianus_chromosome_1,_X_couchianus-1.0,_whole_genome_shotgun_sequence

CCTTAAAGCACATATGTCAGAGTCAAGGCCCGCGGGCCACATCCGGCCCGCGAGAAGGTTTTTTTACGGCCCCTGGAATGATCTTGATTTATTATTAGAACCGGCCCGCAGACCGCAGTAAGCCGGCACCCCGTCTGAAAAAAAAATACCTTCCTTAT
TTGAACTGTAAGTAGTTCTTTAAATGTTCTGTGAGATGATGTACTTACCCATGTATCCATAACAACCGGAACTTTCAATATCCACCAAGTTATTGCCGAAATATACCATCTATTAAAGGAGTGCCCCCCGGATTAATTACACTCTCTGCAGCTGCAAAGTTAC
AGTATTAACCCGATACTTGCTGGAAAGTGCAACGTCTCCCCTTTCAGAAACCGTTGGAATTTTTCCACTTAGCGCTACGTGTGGCGGAATTACGGTACTGCAAATTTAGATGTGTCGCCGGCGACACGTGTAGAAGGGATTTATTAGCACAGCAGTCGT
CAGCATAACTGTAAAATTAACTTTCAGATACCCATCAAAAATGGCAAAAAGGAAGGTGGACACTGAGAACCGGGGGTTTCAAACAAGGTGGGAGTCGGAGTATATGTTCACGGAGGTAGCTGGAAAACCTGTGTGTCTTCTGTGTGGAGAAAGTGTG
GCGGTACTGAAAGAGTATAATCTGAGACGACATTATGAAACGAAACACGCGGACAAAAACAAGAATATGGACATGGAACAAAGGCTACAGAAGGCCGAGGAATTAAAACGAGGCCTCAAATCTCGACAGGCTCTGTTCAAAAAAGCCAAATCACAAGG
CCAGGCTGCTGTCAAGGCCAGTTTTATTTTGGCAGAAGAGATCGCTAAATCAGCCCGGCCATTTACGGAGGGGGATTTCATCAAAAACTGTATGATTAAAGTTTGTGACGAAGTTTGCCCAGAAAAAAGGCAACTCTTTTTAAATGTGAGTCTGAGCAG
AAACACCATTGCCGAGCGAGTAGTCCAGTTGTCCATCAATCTAAAAGAGCAGCTTGTGAAAAAGGGAAAAGATTTCATCGCATATTCCTTGGCTGTGGATGAGAGCGCCGACATTTCTGACATTGCCCAGTTGTCAATTTTCATCCGCGGAGTGGACTC
CAGCCTAAGCGTGACAGAGGAGTTGTTGGCTTTACGTCCTATGCATGGCACAACTACGGGGCATGATTTGTATGAAGAGGTGTCAAGATGTGTAAATGAGATGGAGCTGCCTTGGGAAAAACTCGTGGGTTTGACAACCGACGGAGCACCTGCGATG
TGTGGACACAGGAGCGGACTGGTGGCGAAGATACGGGAAAAGATGCAAGAGGAAAACGCGACAGGTGAGCTGACAGCTTATCATTGTATCATACACCAGGAAGCGTTGTGTGGTAAAGCCTTAAAAATGGAACATGTAATGAGCATCATCACACGCG
CAGTTAACTTTATCAGAGCCAAAGGTTTAAATCACCGCCAGTTCAAGGTATTTCTGACGGAGTTAGAAACGGAGCATGGTGATTTGCCTTATCACACAGAGGTGCGATGGTTAAGCCAGGGAAAGGTGCTCCAAAGATGTTTCGAGCTGCGTGAGGAG
ATTTGTCTGTTCTTGGACAGCAAAGGGAAAGACACAACACAACTCCGAGACGAGATGTTTCTGTGTGAAATGGCTTTTCTGTGTGACATTACAAGTCATCTGAATACAATGAACTTGCAGCTGCAGGGTCGGGATCGTGTCATCTCTGATATGTACAGT
ACAGTGAAGGCATTTAAAACCAAACTGACTCTGTGGGAGACGCAGATGCGGAAAGAAAATTTGAGCCACTTTCCCAGCTGCCAGACCATGAAAGAGAAGCTCTCTACCAGTGCGTTCCCGAGCGCACAGTTTGCTGATAAAATAGGTATGCTTTCCG
CTGACTTTCGACGCCGATTTGCTGACTTTGAAGCACAAAAAAACAGGTTTGAACTGCTCGGTAATCCATTTGGTGTTGACGTGGAAAGCTCACCACCAAACCTCCAAATGGAGTTGATTGACCTCCAATGCAATGATGCACTGAGGGCAAAATATGCG
GCAGTGGGTGCTGCGGAGTTTGCCCGTTTCCTCCCCGACACAATGCCCCAGCTGCGCATCCAGGCTGCTCAAACGTTGTCTATGTTTGGCAGCACATACCTGTGTGAACAACTGTTTTCTTTGATGAACCTGAACAAAACATCACACAGAAGTCGAC
TTACTGCTGAACACCTTCACTCAATTCTGAGGATTTCCTCAGCTCAGAGCCTTACCCCGAACATTGATGAACTTGTGGAAAAGATGGGACACCACCAAGTATCACCCTCAACCTCAAACAAGTGAACATTACTGTGCAATCACATATTTAGAGTTTTTACT
CAGTTCAAGTTTAAAAGTTAAAGTTTAATATTTGTTTTCACTGCATGTTACTTCTCCTTGATGAAAGTGTTGTTTTTGATTAATAGATTTTTGCACTTTGTTTTATTGTATTTCAATCCAATTATATTTTAAAAATATTTCAGTTGTGTCTGTTGAAAATTAAAGATT
ACTGACAGAGCCATAAGAAAATATTGCTTTATTTATCTGATCATATTGGAATATATTTGTTAGGTTTTCAGTAGGTTCAATTAGGTTCACTTGACTATATGCGTCATTTAAAAAAATTTCAATGAACATTCGATCAGTCCGGCCCTCGGCTTGTAGCTAAATTTT
TTATTTGGCCCTCCGTCCATTTGACTTTGACACCCCTGCCTTAAAG

>NW_021144926.1:125362-130272_Gouania_willdenowi_unplaced_genomic_scaffold,_fGouWil2.1_scaffold_175_arrow_ctg1,_whole_genome_shotgun_sequence

GTCTAAACCAGGGGTGTCAAACGTACGGCCCGTGGGCCGGAACCGGCCCACCAGTGGGTTCAGTCCGGCCCGCGGGATAAATCTGCATTATTAAAGAAGAATAAACTTCATTTTCTTTAAAATTTGCAGTTCCTATATTGTCCGCTAGGGGCGCTATGT
TTTAGTCAGTTCTATCGCTTTACCCACTTTTTCTGTTGACCCTGGCACCCTCTTCAGTGAAATGTCTTTGTCAAAAAAAAGAAAAGTGGACACAGAGTGTCGGATTTTTCAAGAAAAATGGACCTCTTCCTATATGTTCACGGAGGTGAATGGGAAACCC
GTGTGCCTGGTGTGCATGCAGCAAGTTTCGGTGCTTAAGGAATACAATATTCGGCGCCACTATGAAACTCAGCATGGTGAAAAATACAACACCTTGCACGGAGAACTGAGAAAACAGAAGGTAAATGAAATGCTGGTGGGTCTGAGGAAACAGCAATC
TGTTTTCACCCGGAGCCGAGAGGTCAGTGATGCTGCGGTGAAAGCCAGCTACGTAATTGCTAGCCAAATAGCATTAGCGTCAAAGCCGTACTGTGACGGGGAGTTCCTCAAAAACTGTATGCTGAAGGCTGCCAAAATTGTGTGTCCTGAGAAGCGA
CAAGCTTTTGCCAATATTAGCTTGACGAGAAATACCGTGGCAGACAGGATTTCGGAACTATCGGCAGATTTGGACAGCCAACTGAAACGCAAAGGGGAGTCATTTCTGGCATTTTCTATTGCAATTGATGAGAGTACTGATATTACGGACGTCGCTCAA
CTGGCCATATTCATTCGTGGAGTTGACGAGACTTTGACTGTCACAGAAGAGTTTCTCGAGTTGGTGCCTATGACCGACACCACAACAGCTGAGGACATTTTCCGCTCCGTTGTTGGAGCGCTGGACAGAGTCGGGGTGGACTGGTCCCGCGCCGTA
AGCCTGGCTACTGATGGTGCACCGTCAATGATCGGGAAAAAGGCAGGTGTTGTGACAAAGTTCAGAGAGAAAGTACAAGCCGCAAACGGAGGAGGTAATTTTTGGACATTTCATTGCATTTTGCATCAGGAGGCATTATGTTGCAAGTCGTTAAAAAT
GGATCACGTTATGGAGGTGGTTGTCCGAACTGTTAATTTCATCCGAGCCAGAGGTCTCAATCATCGTCAGTTTGACAGCTTTCTCAGTGACTGTAACATTACCCATGGTCTGCCATACCACACTGAAGTAAGATGGTTAAGCAGAGGTGCTGTGCTGAA
GCGCTTCTTTGATCTACGTGGGGAAATCGGACAATTCATGTAGAAAAAAGGAAAACCTGTTAAGGAATTACAGTGCCCACTTTGGCTGCAGGACCTTGCATTTATGGTTGATATTACAGAGCACTTGAATAATCTGAACAAAATGTTGCAAGGACGCAAA
AAAATTGTAACACAGTATTATGACAGCATACGTGCATTCAAGTTAAAGCTCGCGTTATGGGAGACGCAGATGGCAAGCGGTGACGCTGCTCATTTTCCCTGTCTCAGAGATGTGTGCGCAGCAAGCGTTAACCTCACGTGACACAGTACAAAGACAAG
ATTTCAGGGTTGCTGAGGGAGTTTGAGAAGCGATTTCAGGTCTTTAGCGAACTTGAGACAGAATTTGCAGTTTTTCGCTCACCATTCACAGTCAGAGCTTCTGATGTGCCCATTGACATGCAACTTGAAATCATTGATTTGCAGTGTGATGTAGAATTGA
AGGACACATTTGCTTCTGTGGGCTTGGACACATTTTACAAGTATCTCCTACCAGGCTATCCTAAATTGACAGCACTGGCTGCAAAAATGTTGTCCATGTTTGGGACTACCTACCTTTGTGAGCAGGTCTTCTCAGTAATGAACATTAATAAAACAAAGCTT
CGCTCAAAGCTCACACATAAGCACTTAAATGAGATTCTGAAACTGGCTGCTTCTCAGGACATGAAGCCTGATATTGATGCACTTGTGCAGGCTAAAAGATGCCAAGTTTCAGGGGCAAATACTGAGCAACACTAAATCCTATGGAATGCTGCTTTCAACA
TTGCACTATAAAAATAAACAGCTTTGAATATTTGCTGTTGTAATTGCTGTGAAAGTCAGTGCTAGTCAGTAACAGATTTACAACAGGAGAGTTTTGTTGAGTAAAATATTATTTCTTATGCATGCCATATATGTTATGTATGTGAGTTTTTTTTTTTTACATGTA
TACATTTTATTTTATATTAACAAGGAAGGGGAAAAATTTACTTTATCAAATGTCTGTTTAAAACAGCTGCCACTTAAGATTTCAAGTGTGTGGAGTCAGTTCATGTGTTCAGAATTGCTTCCCAGATGTGGAAAATGGATTTTAAATCATTTCTAAATTTGTATG
TGTTTGATGTATTTTAACATGCAGGACAGTGTGTTTAAGTTCCACAAAACTGTGAATAAATGTTTTGCAACATTGTACAATCACTGATCAGTTTTTATGCATAATGCACTTGAATAAATGTTAAAGTGAGTAAAAGTGTTGTTGAAATTGCACATACTTTTCTT
AAAAACTCTTAGGTTGTTCATAATATATTTAGTAAAAGGGACAATTCAATTCATATAAAGATTTTTATAATTTACTTCTTCTTCTTTGCACTAATACAAAAAAAAAAAAGTGGACTTATTGCTAGTTCTATGTAATTTTGGAATGAGTTTACTAGTCTGGCCCCCTT
GAGATCCGATTAGGTTGTATGTGGCCCCAAGACCAAAGTGAGTTTGACACCCCTGGTCTAAAC

>NC_041362.1:20598387-20604013_Cottoperca_gobio_chromosome_8,_fCotGob3.1,_whole_genome_shotgun_sequence

CTCTTGAGCACATATGTCAGAGTCAAGGCCCGCGGGCCACATCCGGCCCGCGAGAAGGTTTTTTACGGCCCCTGGGATGATCTTGATTTATTATTAGAACCGGCCCGCAGCCCGCAGATCTTTTACACGCACCAATACTACATTTCCCACAATGCAAC
GGTGACGCACCGAGCAGTAGGCTGCTTCATTTCAATATTTATTGGCACAGCAGTCGTCAGCATCACAGTAAAATTAACTTTCAGATACCCATCAAAAATGGCAAAACGGAAGGTGGACACTGAGAACCGGGGGTTTCAAACAAGGTGGGAGTCGGAGT
ATATGTTCACGGAGGTAGCTGGAAAACCTGTGTGTCTTCTGTGTGGAGAAAGTGTGGCGGTACTGAAAGAGTATAATCTGAGACGACATTATGAAACGAAACACGCGGACAAAAACAAGAATATGGACATGGAACAAAGGCTACAAAAGGCAGAGGAA
TTAAAACGAGGCCTCAAATCTCGACAGGCTCTGTTCAAAAAAGCCAAATCACAAGGCCAGGCTGCTGTCAAGGCCAGTTTTATTTTGGCAGAAGAGATCGCTAAATCAGCCCGGCCATTTACGGAGGGGGATTTCATCAAAAACTGCATGATTAAAGTT
TGTGACGAAGTTTGCCCAGAAAAAAGGCAACTCTTTTTAAATGTGAGTCTGAGCAGAAACACCATTGCCGAGAGAGTAGACCAGTTGTCCATCAATCTAAAAGAGCAGCTTGTGAAAAAGGGAAAATATTTCATTGCATATTCCTTGGCTGTGGATGAG
AGCACCGACATTTCTGACATTGCCCAGTTGTCAATTTTCATCCGCGGAGTGGACTCCAGCCTAAGCGTGACAGAGGAGTTTTTGGCTTTACGTCCTATGCATGGCACAACTACGGGGCATGATTTATATGAAGAGGTGTCAAGATGTGTAAATGAGATG
GAGCTGCCTTGGGAAAAACTCGTGGGTTTGACAACCGACGGAGCACCTGCGATGTGTGGACACAGGAGCGGACTGGTGGCGAAGATACGGGAAAAGATGCAAGAGGAAAACGCGACAGGTGAGCTGACAGCTTATCATTGTATCATACACCAGGA
AGCGTTGTGCGGTAAAGCCTTGAAAATGGAGCATGTAATGAGCATCATCACGCGCACAGTTAACTTTATCAGAGCCAAAGGTTTGAATCACCGCCAGTTCAAGGCATTTCTGACGGAGTTAGAAACGGAGCATGGTGATTTGCCTTATCACACAGAGG
TGCGATGGCTAAGCCAGGGAAAGGTGCTTCAAAGATGTTTCGAGCTTCGTGAGGAGATTTGTCTGTTCTTGGACAGCAAAGGGAAAGACACAACACAACTCCGAGACGAAATGTTTCTGTGTGAAATGGCTTTTCTGTGTGACATTACGAGTCATCTG
AATGCAATGAACTTGCAGCTGCAGGGTCGGGATCATGTCATCTCTGATATGTACAGTACAGTGAAGGCATTTAAAACCAAACTGACTCTGTGGGAGACGCAGATGCGGAAAGAAAATTTGAGCCACTTTCCCAGCTGCCAGACCATGAAAGAGAAGCT
CTCTACCAGTGCGTTCCCGAGCGCACAGTTGGCTGATAAAATAGGTATGCTTGCCGCTGACTTTCGACGCCGATTTGCTGACTTTGAAGCACAAAAAAGCAGGTTGGAACTGCTCGGTAACCCATTTGCTGTTGACGTGGAAAGCTCACCACCAAAC
CTCCAAATGGAGTTGATTGACCTCCAATGCAATGATGCACTGAGGGCAAAATATGCGGCAGTGGGTGCTGCGGAGTTCGCCCGTTTCCTCCCCGACACAATGCCCCAGCTGCGCATCCAGGCTGCTCAAACGTTGTCTATGTTTGGCAGCACATACC
TGTGTGAACAACTGTTTTCTTTGATGAACCTGAACAAAACATCACACAGAAGTCGACTTACTGCTGAACACCTCCACTCAATTCTGAGGATTTCCTCAGCTCAGAGCCTTACCCCGAACATTGATGAACTTGTGGAAAAGATGGGACACCACCAAGTAT
CACCCTCAACCTCAAACAAGTGAACATTACTGTGCAATCACATATTTAGAGTTTTTACTCAGTTCAAGTTTAAAAGTTAAAGTTTAATATTTGTTTTCACTGCATGTTACTTCTCCTTAAACAAAGTGTTGTTTTTGATTAATAGATTTTTGCACTTTATTTTATT
GTATTTCAATCCAATTATATTTTAAAAATATTTCAGTTGAGTGGATGATAGAAAATTGCTATTATTGTTTTTTTTCTTTGAAGTAAATTTAGCCCACTTTTGCTAAAATAGAAAATATAGGCTACTGATGGTGCCTTGAATACCGGTTTCTTTCATTTAATGTTCATG
TTATGGGGATTTTTATATAAAGGAAATTTGTCTTTTGTGTCTGTTGAAAATTAAAGATTACTGACAGAGCCATAAGAAAATATTGCTTTATTTATCTGATCATATTGGAATATATTTGTTAGGTTTTCAGTAGGTTCAATTAGGTTCACTAGACTATATGCGTCATT
TAAAAATTTTTCAATGAACATTCGAACAGTCCGGCCCTCGGCTTGTAGCTAAATTTTTTATTTGGCCCTCCGTCCATTTGACTTTGACACCCCTGCTCTTGAG

>NC_051431.1:29899831-29905418_Kryptolebias_marmoratus_isolate_JLee-2015_linkage_group_LG2,_ASM164957v2,_whole_genome_shotgun_sequence

CTTTAAACCACATATGTCAGAGTAAGGCCTGCGGGCCACATCCGGCCCGCGAGAAGGTTTTTTACGGCCCCTGGGATGATCTTGATTTATTATTAGAACCGGCCCGCAGACCGCAGCAAGCCGACAGCCCGCAGATCTTTTACACGCACCAATACTAC
ATTTCCCACAATGCAACGGTGACGCACCGAGCAATAGGCTGCTTCATTTCAGTGTTTATTAGCCCAGCAGTCGCCAGCATAACTGTAAAATTAACTTTCAGATACCCATCAAAAATGGCAAAAAGGAAGGTGGACACTGAGAACCGGGGGTTTCAAACA
AGGTGGGAGTCGGAGTATATGTTCACGGAGGTAGCTGGAAAACCTGTGTGTCTTCTGTGTGGAGAAAGTGTGGCGGTACTGAAAGAGTATAATCTGAGACGACATTATGAAACGAAACACGCGGACAAAAACAAGAATATGGACATGGAACAAAGGC
TACAGAAGGCCGAGGAATTAAAACGAGGCCTCAAATCTCGACAGGCTCTGTTCAAAAAAGCCAAATCACAAGGCCAGGCTGCTGTCAAGGCCAGTTTTATTTTGGCAGAAGAGATCGCTAAATCAGCCCGGCCATTTACGGAGGGGGATTTCATCAA
AAACTGTATGATTAAAGTTTGTGACGAAGTTTGCCCAGAAAAAAGGCAACTCTTTTTAAATGTGAGTCTGAGCAGAAACACCATTGCCGAGCGAGTAGACCAGTTGTCCATCAATCTAAAAGAGCAGCTTGTGAAAAAGGGAAAAGATTTCATCGCATAT
TCCTTGGCTGTGGATGAGAGCACCGACATTTCTGACATTGCCCAGTTGTCAATTTTCATCCGCGGAGTGGACTCCAGCCTAAGCGTGACAGAGGAGTTTTTGGCTTTACGTCCTATGCATGGCACAACTACGGGGCATGATTTGTATGAAGAGGTGTC
AAGATGTGTAAATGAGATGGAGCTGCCTTGGGAAAAACTCGTGGGTTTGACAACCGACGGAGCACCTGCGATGTGTGGACACAGGAGCGGACTGGTGGCGAAGATACGGGAAAAGATGCAAGAGGAAAACGCGACAGGTGAGCTGGCAGCTTATC
ATTGTATCATACACCAGGAAGCGTTGTGCGGTAAAGCCTTGAAAATGGAACATGTAATGAGCATCATCACACGCGCAGTTAACTTTATCAGAGCCAAAGGTTTAAATCACCGCCAGTTCAAGGCATTTCTGACGGAGTTAGAAACGGAGCATGGTGATTT
GTCTTATCACACAGAGGTGCGATGGCTAAGCCAGGGAAAGGTGCTCCAAAGATGTTTCGAGCTTCGTGAGGAGATTTGTCTGTTCTTGGACAGCAAAGGGAAAGACACAACACAACTCCGAGACGAGATGTTTCTGTGTGAAATGGCTTTTCTGTGC
GACATTACGAGTCATCTGAATGCAATGAACTTGCAGCTGCAGGGTCGGGATCGTGTCATCTCTGATATGTACAGTACAGTGAAGGCATTTAAAACCAAACTGACTCTGTGGGAGACGCAGATGCGGAAAGAAAATTTGAGCCACTTTCCCAGCTGCCA
GACCATGAAAGAGAAGCTCTCTACCAGTGCGTTCCCGAGCGCACAGTTTGCTGATAAAATAGGTATGCTTTCCGCTGACTTTCGACGCCGATTTGCTGACTTTGAAGCACAAAAAAACAGGTTTGAACTGCTCGGTAATCCATTTGCTGTTGACGTGG
AAAGCTCACCACCAAACCTCCAAATGGAGTTGATTGACCTCCAATGCAATGATGCACTGAGGGCAAAATATGCGGCAGTGGGTGCTGCGGAGTTTGCCCGTTTCCTCCCCGACACAATGCCCCAGCTGCGCATCCAGGCTGCTCAAACGTTGTCTAT
GTTTGGCAGCACATACCTGTGTGAACAACTGTTTTCTTTGATGAACCTGAACAAAACATCACACAGAAGTCGACTTACTGCTGAACACCTTCACTCAATTCTGAGGATTTCCTCAGCTCAGAGCCTTACCCCGAACATTGATGAACTTGTGGAAAAGAT
GGGACACCACCAAGTATCACCCTCAACCTCAAACAAGTGAACATTACTGTGCAATCACATATTTAGAGTTTTTACTCAGTTCAAGTTTAAAAGTTAAAGTTTAATATTTGTTTTCACTGCATGTTACTTCTCCTTGAGGAAAGTGTTGTTTTTGATTAATAGAT
TTTTGCACTTTATTTTATTGTATTTCAATCCAATTATATTTTAAAAATATTTCAGTTGAGTGGATGATAGAAAATTGCTATTATTTTTTTTCTTTGAAGTAAATTTAGCCCACTTTTGCTAAAATAGAAAATATAGGCTACTGATGGTGCCTTGAATAATACAGGTTTC
TTTCATTTAATGTTCATGTTATGGGGATTTTTATATAAAGGAAATTTGTCTTTTGTGTCTGTTGAAAATTAAAGATTACTGACAGAGCCATAAGAAAATATTGCTTTATTTATCTGATCATATTGGAATATATTTGTTAGGTTTTCAGTAGGTTCAATTAGGTTCAC
TAGACTATATGCGTCATTTAAAAAAATTTCAATGAACATTCGATCAGTCCGGCCCTCGGCTTGTAGCTAAATGTTTTATTTGGCCCTCCGTCCATTTGACTTTGACACCCCTGCTTTAAAC

>Larimichthys_crocea_isolate_SSNF_chromosome_XVI,_L_crocea_2.0,_whole_genome_shotgun_sequence

CCCTAGAACATATGTGTCAAACTCACGGCCCGCGGGCCACATCCGGCCCGGCGTACAATTATATCCGGCCCGTGAAATCATTTTATTAATCTACTATAATGGCCCGGCTATATGAAACGCTGATAACTCTCAAACTACAGATCCCATAATGCAGCGCTTCA
GCTGCCTTGCCGCCGCAAGACTTACTGGAAACGTTCCCGCGTCAACAGTTGACTTCGCGTTGGTTTCGCGGATGCTAATCCCTCACAATGGCCAAGAGAAAAGTTGACTCTGAAAACAGAGCCTTTAAAACCGATGGGAGGCTGAGTATATGTTTAC
TGACATTGCCGGTAAACCCGTGTGTCTTCTTTGTGGAGCTAATGTGGCCGTAATTAAGGAATATAATGTAAAACGGCACTACGAGACAAAACATCAGGATAAGCTAAAAAACCTGAATGCAGAGCAGAAGATGCAGAAAGTCGAAGAGTTAAAGAAGAA
TATGAAGATCCAGCAGATGTTTTTCACCAGAGCAAAATCACAAAGTGAAGCTGCTGTGAAAGCAAGTTTTATAGTGGCAGAGGAGATCGCCAAATCAGGCCGACCATTTTCCGAGGGAGAGTTTCTGAAGAGCTGCATGATGAAGGTGTGCGACGTCT
TGTGTCCAGACAAAAAGCAGATGTTCGCAAATGTAAGCCTGAGTAGAAATACGGTTGCTGATCGGGTTTGTGAGATTGCCACTGATTTGAGAACACAGTTGAGTGAAAAAAGCAGAGACTTTGTTGCATACTCTCTTGCTGTGGATGAAAGCACAGAC
ATAACGGACACTGCACAGCTGGCGATCTTCCTCCGTGGAGTGGACTCTAATTTGCACGTTACAGAAGAAATATTGGACATTAAATCGATGCATGGAACAGCAACGGGAAAAGACATTTTTGAAAACGTGTGTCAAAGTGTAACCGACATGAAATTGCCC
TGGGACAAACTTGTTGGACTTACTACGGATGGAGCACCGGCAATGTGTGGTGAAAAAAGTGGACTAGTGGGAAGGATGCGAGTAAAGATGCAGGAGGAGAACTGTGTTGGTGAGTTAACCGCATATCACTGCATTATTCACCAAGAATCGCTGTGTG
GTAAAGTCCTAAAAATGGAGCATGTGATGAACACTGTAACGCAGACCATAAACTTTATCCGAGCCAAGGGATTAAATCACCGGCAATTTCAATCTTTTCTGCGCGAAATAGATTCGGATTTTGCCGACATTCCTTATCATACAGAGGTACGTTGGCTGAGT
CGGGGAAAAGTTCTCAACAGAGTTTTCGAGCTCATTAAAGAAATCTGTCAGTTCATGGACAGTAAAGGAAAAGACTCCACAGTTTTGCGGGACGAAAAATGGAAATGTGAATTGGCGTTTCTGGCTGACATAACAGCTCATCTCAACGTCTTAAACCTG
CAGCTCCAGGGACGTGACCGCATGATCACTGACATGTATGATGCGGTGAAGGCATTTCAAGTGAAGCTGCTCTTGTGGGAGACACAAATGCACCAGTGCAACTTGCCTCACTTTCCCCGTTGCCAAGTAATGATGAACCAAGTCGGCACAGCAGCGT
TCTCAAATGCGCACTTTGCTGACAAACTGAGCGCACTGCGCACTGAGTTCGCACGGCGCTTTGGTGACTTTGAAGCACAAAAAAATAACTTCGAGCTGCTTCGCAACCCATTTGCCGTCGATGTGGAAACTGCACCTGTACAGATGCAGATGGAGCT
GATTGAACTGCAGTGTAATGGGACACTAAAGGCAAAGTATGACACTGCAGGGCCCGCACAGTTTTTTCGCTCCATTCCTGAAGCAATGCCCCAGCTCCGTCTACATGCGGCTCGAACCTTGTGCATGTTTGGTAGCACATATCTATGTGAGAAGCTGT
TCTCAGCGATGAAGCTTAACAAAACAGCACACAGAAGTCGTCTCACTGATGAACACCTGCAGTCCATCCTGAGAATCTCTGCAATACAGGACCTCACACCAAACCTGGATGAAATTGTTGCCAAGAAAAGATGCCTAGCATCCAGCTCTGACAAAATG
GCATAAGCTGAAAAAAAAAAAAAAAAAATGAATGAAAACTTAAAAAAAATGAACTGAATGTTTTAATTTGTATTTTAACTTTTGGTGAAAAGCACAAATTTTAATTATATTTGCATTTTTTCATTTTCATGAATGCATTTTGAATGTATATAGTTTTGACAGGAGC
AAAGTATTTTAAGTTATTAATGTTTAAGTTTGTTTATTCTGGAATAATATTCCTGTCTGATTTTAATTCATATTTGTGTTAAAAAAAAACGTCAAGTTTTAGTGTATTCAATAAATGTTTATCCTGTTCGGCCCGCAACCAAAGGTGTGGTTTGAGTTTTGGCCC
TCTGTACAATTGAGTTTGACACCCCTGCCCTAGAACAACTTCAGCGGCTTATAGAGCCTTCTAAAAAAAAAAACTCTAGAACATTGTCAGTGCCTACTAAAACCTCCTATCAGACACCTAAAATCTCCTGAATGCACATCAGAACCTTATGAAAGAATACAA
GGATCTCTTTAAGGATCCATAGAACCTCTTTGTTGGCCAATAGGACATCTTAAAGAAATAGAACATCATAAGTGGCTACTAAAACCTCCTACTGGACCTCTAGAACCCTTTCAATGGCCTGAACCTCATGAAGAACCACTAGAACATCCTAAATGACAATA
GTACTGCCTTCATAGATCACTAGAACATTATCAAATGCTATTAAAACCTCCTAAAGAACCCAGCTACCCTAGAA

>NC_055658.1:14862861-14867684_Chelmon_rostratus_isolate_fCheRos1_chromosome_1,_fCheRos1.pri,_whole_genome_shotgun_sequence

GTGTAGGGCATATGAGTCAAACTCAGGCCCGCGGGGCAAATTTGGCCCGCAGTGTGATTATAATTGGCCCGCGAGATCATATCAAATGTGCATTAGAGCTGGCCCGCGGTATATACTGTGCCGCTAATACTACAAAGCCCACAATGCTTTGCTAGTATTT
TGGCGCGCAGTAAAACCCGGCAAGCGCTCCTTACTTCCGTTCACAGTCATTCATCCATGCCCCGGTCGCATCGGACAAATTAATAATTTCACCCTCCACAAAAATGGCCAAACGAAAGATAGACAACAGGACCTTCCAAGACAGGTGGGAGGCAAATT
ATTTGTTCACGACTATAAAGGACAAACCTGTTTGTCTTGTGTGCGGAGCTGGCGTGGCTGTAATAAAAGAGTATAACATAAGAAGACACTATGAAACGAAACATTATGAAAAGTACAAGGACCTGGACCTAAAGCAGAAGCTGCAAAAGGTGGAGGAGA
TGAAAAGAAGTCTGGTTTGCAGGCAGACCTTGTTCACGAAAGCAAAATCAAAAAGTGAAGCTGCTGTAAAGGCCAGCTTTATTGTGGCAGCAGAGATCGCAAAATCAGCCCGGCCCTTCAATGAGGGAGAGTTTGTCAAAAAGTGCATGGTCAAAGT
TTGCGACATCGTGTGCCCAGATAAGAAGCAAGAATTTTTAAACGTGAGCCTGAGCAGAAATACCATGGCTGAGCGCGCGTGTGAGCTTTCCACTAATCTACATGAACAACTAATAAAAAAGGGAAAAGATTTTATTGCATACTCCCTTGCTGTGGATGAG
AGCAGCGACACGTCTGATACTGCACAGCTGTCAATCTTCATCCGTGGAGTAGACTCGAGCCTGTGCGTCACAGAGGAACTTTTAGGATTAAAATCAATACATGGAACGACCACAGGAAAAGATATCTTTGAAGAAGTATCCAAATGTATAACTGAAATGA
GCCTGCCTTGGGATAAACTTGTGGGACTGACGACAGATGGTGCGCCCGCAATGTGCGGTCAAAAGAGTGGATTGGTCGGCAGGATTCAGGAGAAGATGCGGGAGGAAGATGCAGGTAAGATTACAGTTTATCACTGTATCATTCATCAGGAAGCGCT
CTGTGGCCAAGCTCTGCAAATGGAACATGTTATGAGCTCTATAAAAGGAGTAGTCAACTTCATAAGAGCCAAGGGTTTGAATCACCGGCAGTTCAAGTCTTTTCTGGAGGAGTTGGATTCAGAATACAGAGATGTGCCTTATCACACAGAGGTTAGATG
GCTAAGCAGTGGGAAAGCACTGAACAGATGTTTTGAGCTGCGCGAGGAAATCTGTCAGTTTATGGAGAACAAAGGGAAGGACACAACAGAGCTGAGAGATGAAAAGTTTCTGTGTGAGCTGGCATTTCTCTCTGACATTGTGAGCCATCTCGATGTG
CTTAACCTGCAGCTTCAGGGGCGGGGCCACGTCATCACAGACATGTACGCAGCTGTGAGGGCCTTCAAAACTAAGTTGCGTCTGTGGAAGACTCAGATGCTGCAAGGAAACTTTGGTCATTTTCCGTGCTGCCAAACCATGGCAGCGCATATTTCTC
CTGCCATGCTGCCTAGCGCACAGTTCGCTGAAAAAATCAACGCGCTCAGCGCCGAGTTTTCCCGGCGATTTGCCGACTTCGAGGCTCAGAAAGGGAGATTTGAACTGCTCAGTAACCCGTTTGCGATCGATGTGGAAAGCGCACCAACCAGCTTCC
AAATGGAGCTAATTGAACTCCAATGTAATGACATGCTCAAATCAAAATATGACTCTGTTGGTGCTGCGCAGTTTCCATGTTTCCTCCCCGACACAATGCCTCAATTACGCACCCAAGCTGCTCAGATGCTCTCAATGTTCGGCAGCACATATCTTTGTGA
GCAACTGTTCTCTATGATGAAGGTGAACAAAACACCTCACAGAAGTCGACTCACTGATGAGCACCTTCACTCCGTCCTGAGGCTTTCCTCAGCTCAGAGCCTGACTCCAGACTTTGACGAACTTGCATCTAAGAAAAGATGCCAGGTATCTGGCTTAG
ACCAATGTGCAGAGTACATCAGAGCGTAGCAAACTGAGCTTGAATATATCCGTTTCTTATGCACTTTTTCTACTGCAAGGCATGGTCTGTCAATAGTTGAAATGTTGGATTTTTATCGAATGACATTATTATTTTTGGTTCTGTTGTTGTTGGCTTTGAAAAA
GATTTACTTGAAAAAGGAACTTGAAAGGATAAGGGGAGAGACATACTACTTTACTTATTTAAATAAGAAATGAATACCACTGTGTCTTTTATTTCAAATTTAATTTCTCATGTTTTTGTAATATCAAGCTTTGGTTGTTCCATATTCTGTGTTTAAGCAAAACTA
AAGTTTGTTTCCATATGAAAAGGTTAAACATTACATATCAGTTGCAGTTAATTTTTCAATAAATATTGACTTTGGCCCGTGACTTTACTTCTCTGCTTAATTTTGGCCCACTGTGAATTTGAGTTTGACACCCCTGGTGTAGGG

>NW_020193272.1:23008-27041_Maylandia_zebra_isolate_#2_Kocher_and_#3_Kocher_ecotype_Mazinzi_reef_unplaced_genomic_scaffold,_M_zebra_UMD2a_001670F_pilon_quiver,_whole_genome_shotgun_sequence

TGCTAGACCAGGGGTGTCAAACTCAAATACACAGTGGGCCAAAATTCAAAACTGGAACAAAGTCGCGGGCCAACGTTAATATTTATTGAAAAAAATCTTCCTCCAAATATAACAAGGAACCTTTTCTTACAGACTCAAATAAGTTTTGCTGAAAAACTGAA
TATGGAATAAGAAACGCTTAATACTGAACGATATATAACTTATATAAACATATATAACTTAATATTGCACACATGCAAAATTGAAATTCCAATTAAATAACATATCACTGGAATGCATTTCTTAAGTAAAAATTGTATTTGCAGCCTTCTGATTTAAATTTTAGCAGT
AAACTTTTTCCCCAGGCTAATAATAAATGTAAAAATAAAATAACAATAATAAACCACTTAACTAATAAAAATATCATTAAACGTGAAACCATTCAAGCCCATGTCTGGGAGTAGCAAGACAAATTGCACAAAGAAAATGGTAATTATAGCTCAGTTTGCTACAC
ACTGATCTACTCTGATGCGTCCAAGCCAGACACCTGGCATCTCTTCTTGGATGCTAGTTCCTCAATGTCTGGGCTCAGGCTCTGAGCTGAGGAAATCCTCAGGATCGAGTGAAGGTGTTCATCGGTCAGACGTCTCCTGTGAGATGTTTTGGCCATCT
TCATTGAGGAGAATAGTTGCTCACATAGGTAAGTGCTGCCGAACATGGAGAGCATCTGAGCAGCGTGGGTCCGGAGCCCAGGCATTGTGTCAGGGATGAAATGTGGAAATTGTGAAGCGCCCACAGCATCATACTTTGATTTCAGCGTGTCACTACA
CTGGAGTTCAATCAGCTCCATTTGGAGGTTTGTTGGTGCTTTTGCCACGTCAACTGCAAAAGGATTACTGAGCAGTTCAAATCGACATTTCTGGGCGTCAAAGTCGGCAAACCGCCAGGTAAACTCAGTACTGAGTACACAGAGTTTTTCAGCAAACT
GTGCACATGGGAACACAGCAGTAGAGATTTGCATGTTCATCGTTTGGCAGCAGGGAAAATGGCCCAGGTTTCCTTGTAGCATCTGATTCTGCCACAAGCACAATTTCGTTTTAAAAGCCCTCACTGCAGTGTACATATCTGTGATGATACGCCCCCGTC
CCTGAAGCTGCAGGTTCAGCGCATCGAGATGGCTCGTGATATCACACATAAAGGCCAGCTCACACAGAAACTCTTGCTCCCTGAGCTCAGCTGTGTCTTGCCCTTTGCTTTCCAGAAACTGAAATATTTCCTCGCGTAGCTCGAAACATCTGTTCAGT
ACTTTCCCTCGGCTTAACCACCTCACCTCCGTGTGATACGGCACGTCTCCGTATTCCGAACCACACTCCTCAAGAAAAAATTTGAATTGGCGGTGATTCAGACCTTTGGCTCTTATAAAGTTAACTACTCGTGTTACTGCGGTCATAACATGTTCCATCTT
TAGGGCTTTGCCACAAAGTGATTCTTGATGTATGATGCAGTGATAAACAGTCAGCTCACCTGCACAGTTCTCCGCTCGCATCTTCTCCCGTACCCTGCCCACCAGTCCGCTCCTTTGACCACACATCGCTGGTGCACCATCTGTCGTTAACCCCACAA
GTTTTTCCCATGGCAACTTCATTTCAGTTAGACATTTGGAAACCTCTTCAAATATTTCCTTCCCTGTGGTTGTTCCATGCATTGATTTTAATCCTAAAAGTTCTTCCGTAACACAGAGAGTTGAGTCCACACCGCGGATGAAGACTGAAAGCTGGGCAGTA
TCAGAGGCGTCGCTGCTCTCATCCATAGCGAGGGAGAATGCAACGAAACTGTTTCCCTTCTCCATCAGCTGGTTATTTAGATTGTTGGCAAGTTCACGTGTGCGATCAGCTACTGTGTTTCTGCTCAGGCTAACATTTGAAAATACTTGCTTTTTCTCTG
GGCACACTTTCATCATACAACTTTTCACAAACTCTCCCTCACTGAAGGGCCGGGCTGATTTTGCGATCTCTGCTGCCACAACAAAACTAGCCTTTACAGCAGCCTCACTTTGTGATATGGCTTTTTTGAACATAGTCTGTTGTGAAACAAAACTTCTTTT
CATCTCTTCTGCTTTCCGGCGCCTTTGAGTCATGTCCAGGTCCTTGTACTTCTCTTGGTGTTTCGTTTCATAGTGTCGTCTAATGTTGTACTCCTTGGTTAAAGCCACATGAGCTCCACATACAAGACAAACAGGTCTGTCTTTTACATAGGTAAACAGAT
ATTCTGTCTCCCACCTGTCCAGGAAGCTCCTGTTTTCGTTCTTTGTTTTCGCCATTTTGGGAGAGGTAGCGCGGCAGTATTCATGCGGTTGCTATGACTACAGTAAACAGAGGAGAGGGCGTTAATGGGTCCTGTCCTGACTGACGCGCCAAAACGC
CAACAGAGCATTCTGGGAGTTGTAGTATTAGCAGTACATGCGCTGCATAAGACCGGCGGGCCAGCTCTAATAGTAATTTGGTATGGCCTCGCGGGCCAAATGTAATTAGGCTGCGGGCCAAATTTTGAGTTTGACACCTATGTGCTAGAC

>NC_052307.1:41485384-41490357_Salvelinus_namaycush_isolate_Seneca_chromosome_1,_SaNama_1.0,_whole_genome_shotgun_sequence

GTCTAATGCATGGGTGTCAAACTCTGGCCCGCGGGGTAATTATATTTGGCCCGCGAGACAATACCAAATTACTACTAGAGCTGGCCCGCCGGTATTATACAGCGCATTCACCGCTAATACTACGAATCCCATAATGCTCTGCTGTTGTTTTCGCGCGCCA
ATCAGGACAGGACCCAGAAATGCCCTCTCCTCTGTGACAGTAGTCATAGCAACATAGACGCTACAACTGTCAGCGCGCTATCCCTTCCCAAAAATGGCGAAAAGAAGGCAGAAAACAGGAGCTTTCTGGACAAGTGGGAGGCAGAATATCTGTTTAC
ATATGTAAAAGACAAACCTGTTTGTCTTGTTTGTGGAGTCAACGTGGCTGTAAGTAAGGAGTACAACATTAGACGACACTATGAAACGAAACACCATGACAAATACAAGGACCTGGACATGACTCAAAGGAGCCAGAAAGTAGAGGAGATGAAAAGAAG
TTTGGTTTCACAACAGAATATGTTCAAAAAAGCCACATCACAAAGTGAGGCTGCTGTAAAGGCTAGTTATATAGTGGCAGCAGAGATCGCAAAATCAGCCCGGCCCTTTAATGAGGGAGAGTTCGTGAAAAAGTGCATGATGAAAGTTTGTGACCTCGT
ATGCCCAGAGAAAAAGCAAGCATTTTCAAACGTGAGCCTGAGCAGGAACACAGTAGCTGATCGCACATGTGATCTTGCCACCAATCTGTATGACCAGCTGATGGAAAAGGGAAAAGATTTCGTTGCGTTCTCCCTCGCTGTGGATGAGAGCTGCGAC
GCATCTGATACTGCTCAGCTGTCAGTCTTCATCCGTGGAGTGGACTCAAATCTGTGTGTTACGGAGGAGCTATTGGGATTCAAATCAATGCATGGCACAACCACAGGAAAGGAAATCTTTGAGGAGGTTTCCAAATGTGTAACTGAAATAAAGCTGCCG
TGGGATAAACTCGTTGGATTAACGACAGATGGTGCGCCAGCGATGTGCGGTAAAAAGAGTGGACTGGTGGGCATGGTTCGGGAGAAGATGCGGGAAGAGAACTGTGCAGGTGAGCTAACTGTTTACCACTGCATCATACATCAGGAATCACTGTGT
GCCAAAGCCCTAAAGATGGAACATGTTATGACCACAGTAACACAGGTAGTTAACTTTATAAGAGCCAAAGGTCTGAATCACCGCCAGTTTAAATTTTTTCTAGAGGAGTGTGGTTCGGAATACGCAGACGTGCCGTATCACACAGAGGTGAGATGGCTA
AGCAGAGGAAAAGTACTGAACAGATGTTTCGAGCTGCGTGAGGAAATATGTCAATTCCTGGAAACCAAAGGGAAGGATACAGCAGAGCTCCGGGAGCAAAAGTTTCTGTGTGAGCTGGCCTTTCTCTGTGACATCTCGAGCCATCTCGATGCGCTGA
ACCTGCAGCTTCAGGGGCGGGGGCGCATCATCACAGACATGTACGCTGCAGTGAGGGCCTTTAAAACTAAACTGTGCCTGTGGGAGAATCAGATGCTGCAAGGAAACCCTTGCCATTTTCCCTGCTGCCAAACCATAAAAGCGCAGATCTCTACCGC
CGTGTTCCCATGCGCACAGTTTGCTGAAAAACTCAGTGTTCTCGCCGCTGAGTTTAGCCGGCGATTTGCCGACTTCGATGTCCAGAAATGTAGGTTTGAACTGCTTAGTAATCCCTTCGCAGTTGATGTGGAAAATGCACCAACCAACATCCAAATGG
AGCTGATTGAACTCCAGTGCAACGACACGCTGAAGTCAAAGTATGATGCTGTGGGCGCCGCACAGTTTCCACGGTTCATCCCTGACACAATGCCTCAGCTCCGCACCCAAGCTGCTCAGATGCTCTCCATGTTCGGCAGCACTTATCTATGCGAACA
ACTTTTCTCCTCGATGAAGATGACCAAAACAACTCACAGGAGACGTCTGACTGATGAACACCTTCGCTCGATACTGAGGATTTTTTCAGCTCAGAGCCTGAGCCCAGACATTGATGAACTAGCATCCAAGAAGAGATGCCAGGTATCTGGCTTGGGCA
CATCAGATCAGTGTGCAATAATTAACGTTTTCTTTGTGCACTTTTTCTTGCTACAAGGCATGGGCTTGAATGGTTGATTGATTTATTATCATTTTATTTGTAAAATTATTAGCCAGTGGAAAAAGTTTATTTTGGTATTTAAATCAGAAGGCTGCAAATAGAAAA
GAGGCATACGATTTTTATTTAAATTTTATTTATTTAATAAATGAATGCCATTGATGTGTTTTTTCATTTGAAATTCGATTTTGCATGTCTCCACTATTAAATTATATATTGTATGGTAATAAGCGATGCTTGTTCCATATTCAATGTTAAAGCAAAACTTGTTTGGGT
CCATATTAAAAGGTTCATTTGTTCAATGTTGGCCCGCGACTTTGTTCAGGTTTTACATTTTGGCCCACTGGTTATTTGAGTTTGACACCCCTGGTCTAATG

>NC_034201.2:29887417-29892572_Oncorhynchus_kisutch_isolate_150728-3_linkage_group_LG28,_Okis_V2,_whole_genome_shotgun_sequence

GTCTAGAGCATGGGTGTCAAACTCTGGCCCGTGGGCCAAATTTGGCCCGCGGGGTAATTATATTTGGCCCGCGAGACAATGCCAAATTACTACTAGAGCTGGCCCGCCGGTATTATACAGCGCATTCACCACTAATACTACGAATCCCATAATGCTCTG
CTGTTTTCGCGCGCCAATCAGGACAGGACCCAGAAACGCTCTCTCCTCTGTGACAGTAGTCATAGCAACATAGACGCTACAACTGTCAGCGAGCTAACCCTTCCCAAAAATGGCGAAAAGAAGGCAGAAAACAGGAGCTTTCTGGACAAGTGGGA
GGCAGAATATCTGTTTACATATGTAAAAGACAAACCTGTTTGTCTTGTTTGTGGAGTCAACGTGGCTGTAAGTAAGGAGTACAACATTAGACGACACTATGAAACGAAACACCATGACAAATACAAGGACCTGGACATGACTCAAAGGAGCCAGAAAGTA
GAGGAGATGAAAAGAAGTTTGGTTTCACAACAGAATATGTTTAAAAAAGCCACATCACAAAGCGAGGCTGCTGTAAAGGCTAGTTATATAGTGGCAGCAGAGATCGCAAAATCAGCCCGGCCCTTTAATGAGGGAGAGTTCATGAAAAAGTGCATGATG
AAGGTTTGTGACCTCGTATGCCCAGAGAAAAAACAAGCATTTTCAAACGTGAACCTGAGCAGGAACACAGTAGCTGATCGCACATGTGATCTTGCCACCAATCTGTATGACCAGCTGATGGAAAAGGGAAAAGATTTTGTTGCGTTCTCCCTCGCTGT
GGATGAGAGCTGCGACGCATCTGATACTGCTCAGCTGTCAGTCTTCATCCGTGGAGTGGACTCAAATCTGTGTGTTACGGAGGAGCTATTAGGATTCAAATCAATGCATGGCACAACCACAGGAAAGGAAATCTTTGAGGAGGTTTCCAAATGTGTAA
CTGAAATAAAGCTGCCGTGGGATAAACTCGTTGGATTAACGACAGATGGTGCGCCAGCGATGTGCGGTAAAAAGAGTGGACTGGTGGGCATGGTTCGGGAGAAGATGCGGGAAGAGAACTGTGGAGGTGAGCTAACTGTTTACCACTGCATCATAC
ATCAGGAAGCACTGTGTGCCAAAGCCCTAAAGATGGAACATGTTATGACCACAGTAACACAGGTAGTTAACTTTATAAGAGCCAAAGGTCTAAATCACCGCCAGTTTAAATCTTTTCTGGAGGAGTGTGGTTCGGAATACGCAGACGTGCCGTATCACA
CAGAGGTGAGATGGCTAAGCAGAGGAAAAGTACTGAACAGATGTTTCGAGCTGCGTGAGGAAATATGTCAATTCCTGGAAACCAAAGGGAAGGATACAGCAGAGCTCCGGGAGCAAAAGTTCCTGTGTGAGCTGGCCTTTCTCTGTGACATCTCGA
GCCATCTCGATGCGCTGAACCTGCAGCTTCAGGGGCGGGGGCGCATCATCACAGACATGTACGCTGCAGTGAGGGCCTTCAAAACTAAACTGTGCCTGTGGGAGAATCAGATGCTGCAAGGAAACCCTTGCCATTTTCCCTGCTGCCAATCCATAA
AAGCGCAGATCTCTACCGCCGTGTTCCCATGCGCACAGTTTGCTGAAAAACTCAGTGTTCTCGCCACTGAGTTTAGCCGGCGATTTGCCGACTTCGATGCCCAGAAATGCAAGTTTGAACTGCTTAGTAATCCCTTCGCAGTTGATGTGGAAAATGCA
CCAACCAAGATCCAAATGGAGCTGATTGAACTCCAGTGCAACGACACGCTGAAGTCAAAGTATGATGCTGTGGGCGCCGCACAGTTTCCACGGTTCATCCCTGACACAATGCCTCAGCTCCGCACCCAAGCTGCTCAGATGCTCTCCATGTTCGGCA
GCACTTATCTATGCGAGCAACTTTTCTCCTCGATGAAGATGACCAAAACAACTCACAGGAGACGTCTGACTGATGAACATCTTCGCTCGATACTGAGGATTTCTTCAGCTCAGAGCTTGAGCCCAGACATTGATGAACTAGCATCCAAGAAGAGATGCA
AGGTATCTGGCTTGGGCACATCAGATTAGACCAGTGTGCAATAATTTACGTTTTCTTTATGCACTTTTTCTTGCTACAAGGCATGGGCTTGAATGGTTGATTGATTTATTATCATTTTATTTGTAAAATTATTAGCCAGTGGAAAAAGTTTATTTTGGTATTTAA
ATCAGAAGGCTGCAAATAGAAAAGAGGCATACAATTTTTATTTAAATTTTATTTATTTAATAAATGAATGCCATTGATGTGTTTTTTCATTTGAAATTCGATTTTGCATGTCTCCACTATTAAATTATATATTGTATGGTAATAAGCGATGCTTGTTCCATATTCAAT
GTTAAAGCAAAACTTGTTTGGGTCCATATTAAAAGGTTAATTTGTTCAATGTTGGCCCGTGACTTTGTTCAGGTTTTACATTTTGGCCCACTGGGTATTTGAGTTTGACACCCCTGGTCTAGAG

>NC_042977.1:30684131-30698870_Salmo_trutta_chromosome_21,_fSalTru1.1,_whole_genome_shotgun_sequence

TGCTAAGGCATGGGTGTCAAACTCTGGCCCGCGGGCCAAATTTGGCCCGCTGGGTAATTATATTTGGCCCTTGAGACAATACCAAATTACTACTAGAGCTGGCCCGCCGGTATTATACAGCGCATTCACCGCTAATACTACGAATCCCATAATGCTCTGC
TGTTGTTTTCGCGCGCCAATCAGGACAGGACCCGGAAACGCCCTTTCCTCTGTGACAGTAGTCATAGCAACATAGACGCTACAACTGTCAGCGCGCTATCCCTTCCCAAAAATGGCGAAAAGAAGGCAGAAAACAGGAGCTTTCTGGACAAGTGGG
AGGCAGAATATCTGTTTACATATGTAAAAGACAAACCTGTTTGTCTTGTTTGTGGAGTCAACGTGGCTGTAAGTAAGGAGTACAACATTAGACGACACTATGAAACGAAACACCATGACAAATACAAGGACCTGGACATGACTCAAAGGAGCCAGAAAGT
AGAGGAGATGAAAAGAAGTTTGGTTTCACAACAGAATATGTTCAAAAAAGCCACATCACAAAGTGAGGCTGCTGTAAAGGCTAGTTATATAGTGGCAGCAGAGATCGCAAAATCAGCCCGGCCCTTTAATGAGGGAGAGTTCGTGAAAAAGTGCATGAT
GAAAGTTTGTGACCTCGTATGCCCAGAGAAAAAGCAAGCATTTTCAAACGTGAGCCTGAGCAGGAACACAGTAGCTGATCGCACATGTGATCTTGCCACCAATCTGTATGACCAGCTGATGGAAAAGGGAAAAGATTTCGTTGCGTTCTCCCTCGCTG
TGGATGAGAGCTGCAATGCATCTGATACTGCTCAGCTGTCATTCTTCATCCGTGGAGTGGACTCAAATCTGTGTGTTACGGAGGAGCTATTGGGATTCAAATCAATGCATGGCACAACCACAGGAAAGGAAATCTTTGAGGAGGTTTCCAAATGTGTAA
CTGAAATAAAGCTGCCATGGGATAAACTCGTTGGATTAACGACAGATCAGGCCTCTGATCAGGCCCTACACCCAAACAAGGGCACTGCGTTCATCCACCTCTGGCCTGCTCGCCTCCCTACCTCTGAGGAAGCACAGTTCCCGCTCAGCCCAGTCAA
AACTGTTCGCCGCTCTGGCACCCCAATGGTGGAACAAGCTCCCTCACGATGCCAGGACAGCGGAGTCAATCACCACCTTCCGGAGACACCTGAAACCCCACCTCTTTAAGGAATACCTGGGATAGGATAAAGTAATCCTTCTAACCCCCCCCCCCTT
TAAAAGATTTAGATGCACTATTGTAAAGTGGTTGTTCTACTGGATATTATAGGTGAATGCACCAATTTGTAAGTCGCTCTGGATAAGAGCGTCTGCTAAATGACTTAAATGTAAATGTAAATGAGCGATGTGCGGTAAAAGAGTGGACTGGTGGGCATGG
TTCGGGAGAAGATGCGGGAAGAGAACTGTGCATGTGAGCTAACTGTTTACCACTGCATCATACATCAGGAATCACTGTGTGCCAAAGCCCTAAAGATGGAACGTGTTATGACCACAGTAACACAGGTAGTTAACTTTATAAGAGCCAAAGGTCTGAATC
ACTGCCAGTTTACATTTTTTCTAGAGGAGTGTGGTTCAGAATACGCAAACATGCCGTATCACACAGAGGTGAGATGGCTAAGCAGAGGAAAAGTACTGAACAGATGTTTCGAGCTGCGTGAGGAAATATGTCAATTCCTGGAAACCAAAGGGAAGGATA
CAGCAGAGCTCCGGGAGCAAAAGTTTCTGTGTGAGCTGGCCTTTCTCTGTGACATCTCGAGCCATCTCGATGCGCTGAACCTGCAGCTTCAGGGGCGGGGGCGCATCATCACAGACATGTACGCTGCAGTGAGGGCCTTTAAAACTAAACTGTGCC
TGTGGGAGAATCAGATGCTGCAAGGAAACCCTTGCCATTTTCCCTGCTGCCAAACCATAAAAGCGCAGATCTCTACCGCCGTGTTCCCATGCGCACAGTTTGCTGAAAAACTCAGTGTTCTCGCCGTTGAGTTTAGCCGGCAATTTGCCGACTTCGAT
GTCCAGAAATGTAGGTTTGAACTGCTTAGTAATCCCTTCGCAGTTGATGTGGAAAATGCACCAACCAATATCCAAATGGAGCTGATTGAACTCCAGTGCAACGACACGCTGAAGTCAAAGTATGATGCTGTGGGCGCCGCACAGTTTCCACGGTTCAT
CCCAGACACAATGCCTCAGCTCCGCACCCAAGCTGCTCAGATGCTCTCCATGTTCGGCAGCACTTATCTATGCGAGCAACTTTTCTCCTCGTTGAAGATGACCAAAACAACTCACAGGAGACGTCTGACTGATGAACACCTTCGCTCGATACTGAGGA
TTTCTTCAGCTCAGAGCCTGAGCCCAGACATTGATGAACTAGCATCCAAGAAGAGATGCCAGGTATCTGGCTTGGGCACATCAGATTAGATCAGTGTGCAATAATTAACGTTTTCTTTGTGCACTTTTTCTTGCTACAAGGCATGGGCTTGAATGGTTGA
TTGATTTATTATCATTTTATTTGTAAAATTATTAGCCAGTGGAAAAAGTTTATTTTGGTATTTAAATCAGAAGGCTGCAAATAGAAAAGAGGCATACGATTTTTATTAAAATTTTATTTATTTAATAAATGAATGCCATTGATGTGTTTTTTCATTTGAAATTCGATT
TTGCATGTCTCCACTATTAAATTATATATTGTATGGTAATAAGCGATGCTTGTTCCATATTCAATGTTAAAGCAAAACTTGTTTGGGTCCATATTAAAAGGTTCATTTGTTCAATGTTGGCCCGCGACTTTGTTCAGGTTTTACATTTTGGCCCACTGGGTATT
TGAGTTTGACACCCCTGTGCTAAGG

>NW_018114555.1:1004248-1015587_Labrus_bergylta_unplaced_genomic_scaffold,_BallGen_V1_LaB_20160104_scaffold_140,_whole_genome_shotgun_sequence

GTAGTAGACCAGGGGTGTCCAAAGTTTTTTTTAGTGAGGGCCAAATACAAAAAAAAATAATTTGGGGCCGGGCCACACACTAGAGGTGACATATTACCACATGAATACTGTGTGTATTTCTAACTCACTTATAATCTGAAGAACATTGCACTCAGTGGGAG
CAGTGATACTGTCCTTTGGATTGAAGGATGGCTGACATGTCTGGAGTAAGTTTGGTGGTTGAGACACGAAGGACTTCACAGAGATGGCTATCAGTCATTCTGGTTCTCAGTCTGCTTTTGTTATGACTTAACAGAGAAAATGTTTGTTCACATATGTATG
TTGAGCCGAACAGGCTCATCATTCTTTTTGCGTGGCGTCTCATCAGAGGAAACTTGGCATTATCCAAACTCTGATAGAAGTCAGGGAGGGAGAAAAGCTGATGTTGACTCTTCAGAGAATCATCACATTGCATTTCAATCAGTTCTAACTGCAGACTCT
CTTCCACTTCTTCTGCATCCACCAGAAAGGGGGTCGAGAACAGCTTGATTTCTTTTTCAATGACAGAAAAATCTTGGAAACGCTGGTTGAATTCTGTCAGGAGAGATGTAATCACAGACACATATTTCTCCTTTTTAGCAGAAAGGTCGGCCTTTGGAAT
AGCAGACATGATTTCAGACAGCGCAGGGAAGTGTGCAGCATTAAAGCTTCGTAGTTGTGTCTCAAACAGACGGAGCTTTACACAGAAGGCTTTCATGTGCGCATACAGGTGAGGCACCAGCTGTTCTTTGCCTTGTAGGTTCTTGTTCAGTGTATTCA
GATGATGAGTAAGATCAACTAAAAAAGCCAGGTCTGACAGCCACAGAGGGTCACTCAGTTCATGAAGAGGTCGGCCCTTTTCTTTCAAAAACTGGTCGATTTCTGATCTCAGCGAGTAAAACCGCTGCAGCACAGAGCCGCGGCTGAGCCAGCGCA
CATCAGAGTGGTAGAGTACATCCCCGTATTCCGCATCCACGTCAGATAGGAAAGCTTGAAATTCTCTGTGGTACAGTCCTCTAGCGCGAATTATGTTGACAGTTTTCACAACAGTGTTCATCACATCGCCCAGCTGGACAGTCTTGGCACACAGTGCTT
CTTGGTGGATTATACAGTGCATCCTAACAGCCTCACCTCCGCTCTCTTTTACCTTGGCGCACACCATCGATGCCATTCCTTTGCGCTCGCCGGTCATGGCCGGAGCTCCATCTGTTGTTACTCCACAGAGCTTTTCCCATTTAAGCCCCATCTTTTCAA
CGGCCTCCGACACAGCTTCAAAAATGTCTTTACCCGTCGTTGTGCCTTTTAGGCTCATCATATCAAGCAGCTCCTCCGTACAGCAAAAATTATCATCCACTCCCCGCAAAAAAATTAACAGCTGCGCGATGTCTGTGGCATCTGTGCTCTCATCGCATG
CTATCGAGTAAAAATCAAAAGCACAAGCTTTGTCTTTCAGCTGAAGTTTCAAGTTAGCTGACAAGTCCTCGATTCTCCGCACCACTGTATTTCTGGACAAGCTCACGTTTTTGAAATCCTGCACTTTTTCCGGGCACATTATTTCTGTGACTTTGATGAGG
CACTGCTTTATGAAATCGCCGTCTGAGAACGGTTTGCCGTGCTGGGCAATAAGTTTAGCCACTTCATAGCTTGCTGTTGTGGCGTTTTCCTGTATTTTGTTTGCACGAAAAAAAAGCTGTTGTTGAGCTTGCAGGCTAGCTGCCATTTGCTTGACTTTCT
GTGTTCGTTCGGCACCGGTGTACGATGTGTAGCTCTGATGTTTGGTCTCATAGTGCCGACGGACATTATACTCTTTCATCACAGCAACATCTTCCTTGCAAATCAAACAGACACACTTTCCGTTGACTTCTTTGAAGAAATATTCATTTTCCCACCGTGTT
TGAAATCTCCTACACTCACTAGCTATTTTGCCCTTCTTCGGTGCTGCCATTTCTGGTGATTCGCGGTATAAGTTTTTCTTTGCTTAATTTTGTTTAGTGGAGAAGCACTGTTTCCGTGGCTGGCTCCATCTAGTGTTGAAACTGAGAAGTGCAACAGATGT
GAGCTCAAGCTTCTTGTATGGGCCATATTCAATTATATTTTCAGAATTTGCTGCGGGCCAATAAAAACTGGACCGCGGGCCGCAGTTGGCCGTAGTTTGGACACCCCTGTAGTAGAC

>QBIY01012736.1:1167577-1171409_Labeo_rohita_breed_JAYANTI_isolate_DASCIFA01_scaffold_12861,_whole_genome_shotgun_sequence

GTCTAGATCAGGGGTCTCAAACTGCCGGCCCGCGGGCCATTTACGGCCCTCCCTCCCTCCCTCCCCCTCCCTCCGGCCCGCAACTGACGTTAAAAATATAACGCAATCCAGCCCGCAAAATAATAATTAACAATATTTAATATTAATATTACTAATCATTT
TTGTAGTTATTTCATAGCAATTTAAACTACTCATTTAATATACAAACACAATACAAACAAAAACACGGTTAAAAAAATGAATACGTTGATAGCGTAAAAAACCTGCGTGTGGCAGCAGACATGCTAAGAAGAGGGCTAGTTTCAGGGCTTTAGCAGTTTATTT
TTTGTTTGGCTGTGAATGAAAATGTCTTATTCCAAACCGAAAAGGAAAGTGGATGATGAACACAGACAGTTTCAGGAAAGATGGACACTGCAGTATTTTTTTGTGGAGTTTAATGGAAATGCCACCTGTCTGATATGCAAAGAAAAAGTTGCCGTTCTAA
AGGAATACAATCTCAAGCGTCATTACTCTACTAAACACGGAGACCAGTATGAGAAGTACCAGGAAGACGAAAGAAAAAAACGAGCCACCCAGTTGCAGAGAGAGCTAACGTCCCAGCAAACTCTTTTCTACAAAGCCAAAAAGGATGCTGATGCTGCA
GTTGAAGCGAGTTATGTGGTGAGTGAAATGATCACCAAAGCAGGAAAGCCATTCACTGAAGGGCAGTTTATGAAGGACTGTATGTTGAAGGTCGCTGATATTTTATGTCCAGAAAAGAAGAACATGTTCAACAATCTCTCTTTATCGGCAAATACAGTGG
CAGAGCGGATTACTGAATTATCGAGTGACATTTATGATCAGCTACGCGGGAAAGCAGGGGTTTTCACTGCATACTCTGTGGCGCTTGACGAAAGCACGGACAAAACTGACAATGCTCAGCTAGCTATTTTCGTCAGGGGTATTAATGAAAAGTTTGAGG
TGACAGAAGAATTGCTGAGTTTGTGTCCAATGCACGGCCGGACAACAGCCAAAGATATATTTCAGCAGTTGGGCGACGCGATTGAACGCGCTGGTTTACCTTGGAACAGACTAGTAGGCATTACTACCGATGGCGCCCCGTCTATGACCGGTAAAAAA
AACGGACTGGTGGCACTGGTGCAAAGAAAGTTAGAAGAGGAACATGCAGATCCAGCAGTTGTTCTGCACTGCATTATACACCAACAGGCACTGTGCAGCAAATGCTTAAAATATGAACATGTCATGTCTGTTGTCCTAAAATGCATCAATTACATCAGGT
CAAGGAGTTTACAGCACCGCCAGTTTCGTGCCTTTTTAGAAGAAATTGAGGCAACATATGGTGACGTACTTTACTTCACTGAGGTGCGCTGGCTCAGCAGGGGAAATGTCTTGAAGAGATTCTTTGAGCTAAGAACAGAGGTGAAGAGATTCATGGAA
GATGGCAGAATGGATGTTCCTGAATTTGATGATCCTAAATGGGTCATGGACCTTGCATTCCTAGTGGACATTACACAGGAACTGAACATCCTTAACCTGAAGCTCCAGGGCCCTGGTCAGCTTATCACAGCAGCGTATGAAAGTGTGAAAGCTTTCTCC
ACAAAATTAAGATTGTGGAAAACTCAGCTTTCTGCAAAAAACCTCAGTCATTTCACAACATGCAGATCTCTTGTGGAGGAGGGCATAGCATTTAATGGTGATGAATATGCATCTGCTGTTGAAAACCTACTTCAGGAATTTGATAAGCGTTTTGCGGACTT
CAAGGCACACCGTGACACTTTCGAGCTGTTTGCAGACCCCTTCTCAGCTGATGTGGAGAGTGTCCCAAATTTTTTGCAAATGGAACTTATTGACTTGCAGTGCAACAGTGAGCTAAAAACTAAATTCAGAGAGGCACAGGGAAATGCAGACAAGACTG
CACAGTTTCTGAGAGAATTACCTCCATGCTTCCCAGAGCTGTCCAAAGTGTTCAGTCGGGTAATGTGCCTTTTTGGAAGCACATATCTGTGTGAGAAGCTTTTCTCAGCTATGAACTTTAATAAATGCAAGTTCAGGTCCAGGCTTAGTGATGCTCATCT
TGAAGCTGTACTAAGGGTTTCAACTGTTAACACCATCAGGGCCAATGTGGCACGGTTGTGTGAGCAAAAGCGCTGCCAGGTGTCTGGCAAGAAATAGCATAGTTTGCAAATGTATTGCATTTGTTCAGTGTAAGGTTGTTTGTTCAGATTAGTTTTGATA
AGTAACATAAGTTAAAATTAAGTAGGTAACTGTTAATTAAAAAGTAAAAAATATATAACATAATACACAGACACTGTTGTTTATATTTTTGTTAAGGATAACAGAAATATGTTAGCAATGTAATGTAAAAGAAAGTTCAGCATGTAATGTAATGTAAAAGAAAGTTC
AACAACTCAGCAATTAAAGTAAATTTCTACAAATGTTGTGCTTGAATGATAAAATGGCCCTCCTATGAAATATCAGTCACAGCAATGGCCCCCAGCCAATTTGAGTTTGAGACCCCTGGTCTAGAT

>SCEB01001531.1:18286-22142_Acipenser_ruthenus_isolate_WHYD16114868_AA_scaffold2694,_whole_genome_shotgun_sequence

GTGTACAGCAGGGATAGTCAATTATTTTTTGTCAAGGTCCACATTTCTTGGTCAAGGTATAGTCAAGGTCCAGACTCATCTGAAAGTCAAGGTCCGAAACATTTTTCTTGCTGCAAGTGCCCCTGTCCAACAAACACATACAATGCTGTGAATTAGTAATA
TACCCCATTGCTTTTAGTCATATTTTTAGTCCATTAAAAAAAATGTAATAAATTAAATCTGAGACTTTGAGAAAGTACATATATCAAATGTCAAACAGATGTAAATATTAGCATTAAAACACTTTCAGATAACTGTTTTAAGTTTTGAATTAGAGTTTACAAAGAGT
ATTAACTTTGGTTTTGTTAAATAAAATTATAGTAGGCATAGATTAATATAGATAATCTACAAAGCCTTGTTGCTTGTTTAATATTAAAGTTTGAAAGAAAATACTCTCTATCTTAGCTTTTATGATTCAAGTCTTTCCAATAACAAATACACACCCACACACACATG
TTTTAAGCAAAAGAATCATGAATAATTACTGTGAAATACCTGTTAACTATAGTACGGTATCACTACTGAAAATGTAATTTTATACAGCAATACAATTTGAAATAAAATATATGTGAATAATGTAGAATTATAATACTCACCATCAGTGGGAAAAATGACAGGTCCT
CTGGTGGACAAGGGCCTTGAAGTTTGGAGTGAGTTCAGTCAAGGCAAGACGGAGGCAGTTGCACAGATGATTATCAGTCAAAACGGAGCGATTACGCGATTTAATGTTGTTCATTGTTGAGAACGCAGACTCACACACGTATGTCGAGCCAAACATA
GTCAAGATGTTAAATGCCACCTTCCTCAAGTGTGGATACTTGGAGTCCAGCACAAGGGTTGTCCAGAAAGTTTCACATGTAGTTTCAGTGTGCCGCATCTTAAGCACTTCATCAGATTGTAAATCAATAAGCTCCAGCTGTAGTTTTGCTTTTTCAACATC
CGGAAAGGATGCTTTTGCTTGATCACACCAAGTGCCGTTTGCTTGCACCACGAATGGGTTCTTTACAAACAGGAAGACATCTTTAACACTTTCAAATTCTTGGAACCTGTGCTGGAAGTTTTGAAGAAGCCTGTCCAGAGATGACGTCATCTGATGCAC
GTCTGCAGTGTCCCGGCAGCTTATCAGCGTTGGGAAATGCAGCATCCCCCCAGTTTCCAAATCACTTTTGAATAGACTCAGTTTGTTTTTAAATGAAGCGACACTGCTATGGAGATCTACTACATTGTGATTTCGCCCCTGTAGCTTTAAGTTGAAATCAT
TTAAGTGGCCAGTCAAATCAACAAGGAATGCCAGGATGCTCATGCTTGCGGCATCTCTCAAAAATGTGTATAGTTCTTCTGCCCTTTTCCCTGGAATTGTATGCAGATATTCTTCCACATGAACTCGAATTTCCCACAGCCTCCTTAGTACTTGCCCTCTG
CTCAGCCAGCGGATGTCATTGTGGACCAAGAGGTCATCGTAATCGGCAGACACTTCTGAAAGAAAGCTTTTGAATTGCCTGTGTTGCAGTGCTGACTTTGACCGAAGGAAGTTGACAAGTTTTATCACCATTGACATTACATTAGAATACTCATCGCTG
AGCTTTGCACAAAGTGCGGTTTGGTGGATTGCACAGTGATAGGAAATCAAATTGGGGTTCAGGTCTTGCAGACGTTTCACAAGCCCTCGGTGAGTACCGATCATGGCAGGAGCGCCATCTGTTGTAATTGACACAATCTGATTCACATCAAAACCTTC
ATTAGACAAAAAAGTTTGAACAGAATTAAACAAATCCTCACCTGTCGTGCGATTGTTATGCGGTATAAGCGACAACAAATCCTCCACAAATTTCTCTCCATCAAAAAACCGGACATACATTGCTAGCTGAGCTGTGTCCCTGATATCACAGGATTCGTCCA
CTGCCATGGAAAATTTTCCAGTTTTCAGTTTCTCACTTAACTGCTTAAAAACGTCTTGTGCCATGATCTCAGCGCGCCTAATAGCTGTTGTATCTGAAAGTGGTAGTTGTCTGAAGGCTTCAACAACGTCTTTCTTCTCCGCGAAAAGGGTTTCAGCTGT
AGTAACCATACATTGCTTAACCATGTCCGCATCTGTAAAAGGTTTCCTGTGTTTTGCCATAACCCACACACACCTCAGTGAAGCTTCAGTTGCCTTTTCCTGAGCACAAGTAGCCTTTGAAATAATATTTGTGCTATGAGAATATCCATCTTTTAATGAAGT
GATCTTGTCTGATCTTAAACGGGAGCCCAGTGGATATTTGTGGTCAAAATCCATGTGCTTCATTTCATAGTGCCGTTTGATGTTCCCACTTTTAATAACAGCAACAGCCTCTGTACATATTAAACATACCGGTCGAGCATTTGGTCGCTGTGGCAAAACAA
AACAAAAAGCATCGGTCCACTCTTCTTTAAATGAACGATTTTCGGTATCAACTTTCCTTTTTTTTGCTGCCATGTTTAAACACACTGCTGTTAACTTGACATCGAAAGCACTGGAGGCGGGACTACAGCGTTCAACAGTGCGCACGAGCGTACAGTGGA
AGGTGTATAAAAAAGGAACTCTCGTTCGTTTTTTTTATTCGTGGAAACTGACGATAGTTCAAATTAACAGTACACTAGATCAGAGATGGCATTGTGAGGGAATGAGAGGGTAAAAATAATAATTAAAACAATTACAATAATGATAATATTAAGTAAATAAAATG
ATTCCGCGGTCCGTTCAAAAGCGTCTGGCGGTCCGGATTTGGCCCGCGGGCCGCCTATTGACTACCCCTGGTGTACAG

>NW_017860098.1:42537-46909_Paralichthys_olivaceus_breed_gynogenesis_unplaced_genomic_scaffold,_Flounder_ref_guided_V1.0_scaffold602,_whole_genome_shotgun_sequence

GGGCTAGACCAGGGGTGGGGAACGTCCGGCCTCCGGGCCGTATACGGCCCGCGAGGCCATTTCAACTGGCCCGCAATGCAATACAATTTTTATATTTTATATCTTCATATATGCATACATGAATTAAAAAAAAAATCAGGAAAAAAAATATATCGCCCGTC
ATTAGTTTTCCGAGAGAGAGTCCTATGTCCGAGTCAACGTCAGGGTCAGTGAACACAGCATGTGATGTACGTACCACTGCATTGGGTCGGAGTGACTGACACATGACAAGTACCGACACAGACAAGTGATGCATCATGGCGACTGTCAAGAAAAGGA
AGGTGGACGGAGAATGCCGCGTATTTCAGGAAAAATGGACAAATGACTTTTTCTTTGTTGAAGTGAAAGGCAAGCCAGTGTGCCTAGTTTGTGGCGAGGCGCTTGCGGTGATGAAAAAGGCCAATGTCGAGCGCCATTACAGCTCGAARCATGCTAA
ACTCGACGAGTTGAAAGGACAAATGYGTTTGGATAAAATTAACGCTCTTCGTCGGAGTTTGGGTGCTCAACAGGCAGCTWTCACACGGCCACAGACTGACAGAGAGAATATTACGCGTGCAAGTTTTGTGGTGAGTGAACTGATAGCCACGAAGCTG
AAACCTCACGCTGAAGGAGAGTTCGTGAAGGAGTGCCTCGTAGCCGCCGCGGAGCTGCTAGCCCCCGACAAAGTGAAATCATTTCAAAGCGTCAGTTTGTCCCGGAGAACCGTTTCAGAAAGGATTACTGATATGGCGCAAGATATTGAAAAAACAC
TCAAGAACACTGCAAGAGACTTYGAGTATTTTTCTCTGGCCTGTGACGAGACAACAGACATCACACACACAGCGCAGCTTGCCATTTTTCTGCGTGGGATTACGTCTGAGTTTGAAACAAAGGAGGAGTTGCTGTCTTTGCAAGCCATGCATGGCACT
ACTAAAGGTGAGGATTTGTTCAATCAAGTTATTGTGGCCATGAACAATTTTGAACTGCCATTTGAGAAGTTGAGCGGCATCGCCACTGACGGAGCTCCCGCAATGGTTGGCGYGCAAAAAGGATTGACTGCTTTAGTCAAAAAGAAATGAGCCGACT
GAGTCTGGATCCAAGTGATTTAGTTGTATGCCACTGTATCATACATCAAGAGAGTTTGTGTGCACATTCCCTGAAGCTTAACAATGTGATGACAACAGTTGTGTCCACCATAAACTTCATTAAAGGCAGAGGGCTGAACAACCGCCAGTTCAAGGAGTTA
CTCAGCGAGATTGAGTCAGAGTATGGTGATCTGGTTTATCATTGTGAAGTGCGATGGCTGAGTCGTGCAAATATGCTCGCCGGTTTATACACTGCGGGAAGAAGTCAGACGCTTCATGGAGATGAAGGGTAAACCTGTCATGGAGCTCAGTGATGGC
AAGTTCCTCCGTGATCTGGCCTTCATGGTGGACATCAGCAAGCACCTGTCAGAGCTAAACATCAAGCTGCAAGGTCCCAACCAGCTCATCAGCTCTCTGCTTTCAAATGTGAAATCATTTGAAGTGAAGTTAAGGCTGTGGCAAGGACAGCTGRAAAG
GGGAAACACTGTGMACTTTCCCACCCTACAAGAACAAAAGCCTGATGTTACGACTGAATATGCTGGTGAGTGTGCAAAACTCATCCAGGCAYTTGATGAGAGGTTTCATGATGTGAAAAATATACAAAAGGAACTCGACGTGTTTGCGACACCATTTAA
TGTGCAACCGTCTGATGTGCCAGACAACCTCCAAATGGAAATAATTGATCTGCAAAACAACAACGAGCTCAAAGCCAAGTACCACAACCTTTCTCTGCTGGACTTTTACAAACTGTATGTTCGTGCTGAGGATTTTCCCATCCTGAGGAGACATGCCCT
GAAGTTTGCATCTCTGTTTGGGACAACGTATCGCTGTGAACAGTTCTTTTCAAAACTGACTCTTGCAAAGACTYGCTTCCGCTCAAGACTGACTGACTCAAACTTGGAAAACCAGCTTCGAGTAGCATCATCATCACTGCCAGCTRATATCAGACGCCT
TGCTAGAGAAAAACAGTTCCAGCCATCACATTAGGTGAGTCTAGACATGCAGCAAACATGAGGCTTAGTAACRCAGCAGTTATAGACTTTTTTTGTCTTTTTTTTCATCTTTAGTTATGTGTTCATAATGGTATGGTCCTCTGAGGACTTTGGAAAAATTGA
AATGGCCCTTGATGTGAAAAAGGTTCCCCACCCCT

>NC_044049.1:18472549-18478074_Gadus_morhua_chromosome_2,_gadMor3.0,_whole_genome_shotgun_sequence

CTCTATAGCAGGGGTGCCCAAATTCTTCCCTCTGGAGGGCCAAAATTTAATCGGGGGGAGGCTCGTGGGCCAAAATTGTAATTTTTCAGTATATAAGGTTAAATATGGGTATAAATAGCATGCGTGTGCAAAATCAACACTATCACAAGGTCATTTTGAAA
AATGAATGACAATGTTTATTTGTGCTTCACATTAATGTTCAAAAAATAATAAATAAATCATAAAATCTATCTTCTTTAAATAAGAAAAATAATTGAAGGACTATGAAATATTTGAAGACAGACATGGAACATTACTAGGCCTTGGGCCTGTATCTTCAAATACAAAG
ATTTTTTTTAGTGCAATAAAAGTAACACAGAACAATAACACAGGACAGTAACAAAGTAACACAGGACAGAGCAGAACATACTCCGACACATTGGAGGTCCAGGCCTATACATTATGATTTGAAGTAGGCCTATCAGTGGGAGACATGAAGCCTGTCCTTG
GTTTTTACAAGTCTATTAATATCAGGCTCCAGCTGACTAACTGACAGAGTGAGAATGTCATGAAGGTGAGAGTCAGATAAAGCATTTCTCAGTTTGGACTTATTAAGATTCATCAGGCTGAATGCCTGCTCACAGACATAGGTACTTCCAAAGAGGGAGA
ACATCACCTGTGCATGTTTGCGGATCTTGCCATATCTGTGTGCAGGAACAGATTTGTAAAAGTCCTGTAAAGGAAGCTCTCTAAACCGGCAACAGAGATCTGAATCAGACTGAAGCTCCAAAAGTTCCATTTGAAGCTCCTCACTGACTGCATCCACAT
TTACAGCAAAGGGTTGGGCAAACAGCTCAAATGCTGCTTCGTTTCCATGAAAATCCTCAAAGCGATGGTCAAACTCCACCACAAGATCGCTGAGAATGTTGCAGTATTTTGGTGTGTCTTCCTCCATCAACCCAACTTCTCTGAGGCTGGGAAAATGT
GCCAAATTTCCTAAATGTACAGACACAAAGACAAAAGCAGCAATGTAAGATGCAAGGTTGTTCATTTGATTTTCAATTAAATACCAAACATCAATCAAATGAGAAAAAAACTTGTTTTTTTTGTGTTTATGGCTGCAGTACAAAAAACAAATGTGTTATTGGAT
TTAATGCCTATATTTAAATAGAAAATGTATTCTCCACACACAATGTATGTAACAAATATAATAACATGCTGGTTATAGTGATCAATAACCAGCCTACTACATTGTTTTGTGTTCAATAACATATTCAAGTTAGATCTCACCTGTTAAGAGATGTCTGTTAAGCAAC
TGGAGCTTTTGCTTGAAGGCTCTGACATGGTCGTACAGCTGAGTGATTATCTGGTCTCTCCCTTGAAGCTGAAGATTGAGGATATTCAGCAGCTCTGTGATGTCCACAAGAAAGGCCAGATCTGCAATCCAGTGCTTGTCCGAGGGCACTTGGATGTT
GTTCTCCTTTGTGGCTTGAAACACCCTGATTTCCTCCACGCAGCTCAAAGAAACGTCGCAAAACTTTCCCGCGGCTCAGCCACCGCACCTCCTGGTGGTACAATACATCCCCATACTGGGCATCCATCTCATCAACAACAGCTTTGAACTGACGGTGTG
AGAGCGCCTTTGAGCGAATATTATTGACAATGCCAACGACATCCTGCATCACGTTCTTTAGGCCGATATTCTTAGCGCACAGTTGCTCCTGATGCAAAATGCAATGATATTTAAAAATCGAGCCACCACACTCACGGACTTTGTCCGACACAAGTGCAGT
TAGGCCTGCTCTCTTACCGACCATGGCCGGGGCTCCATCAGTCGTGATGCCAGATAGGTTTCCCACTTCAAACCATTCTTGTCATAACTTGCTCCACTGCCTTAAAAATATCGATACCTCTTGTCGTCCCTTTCAGTGTTTCAAGGCCCGCTAGTTCT
TGGCACACTTCAAAGTCCTCATTGACGCCTCGCAAAAACACCAGCAACTGTGCTGAGTCCGATATGTCGGTGCTTTCATCGCATGCGATGGAGAAGGCAGAAAACCGACCTGCTCTTTGGGCTATCTGGCCCCGAACGTCTTCGGCCATGTCCTCAA
CGCGGCGGGTCACCGTGTTCCGTGACAGGCTTATTTGAGAAAAAGCCCGCTTCTGAGTTGGACACACAGCTTCAGCAGCAACTGTCAGACACTCTTTTACAAAATCACCCGCAGCAAAAGCGTGTCCACTTCTGACAATGATGTTGCTAATGCGATA
ACTTGCAAGGGTGGCATTTTCTTGTGCGGTGGATGGCTGAAGTAAAGTTTGCTGCTGTGTTTCTAGCTTGATTAGCAAGTTTGCCGCCTTTACCTTTCGGTCCTCGCCTGTGTATTTGCGAACTGGTGAGAATGCATAGTTTCATAGTGACGACGAAT
GTTGTACTCCTTTAAAACAGCGACCTTCTGCTTACATACCAGGCATATTGCGTTGGAATCGACCTCGGTAAATAAATAATCATGTTCCCAACATTTTTTGAATTTGCGTTTGTCTGTGTGCCACTGCCGCTCCATCCTTGCGTCTCACTTACTCGCTGTCA
CGTGACTTCGGATTGGAGCGCAAGAGGAATGAGCTCAACAGCGCCACCCTACCATCAATGTTCACAATGTTATTCTATGGTCAGGAATGGAAGTGCACCAATCAATATATTCAATGAGTGCGAGCGCTGATGGTGGAAAACAATAATATATACAGTG
GGTAAGGCGCGGGCCAAATAAAATCAGTCGCGGGCCAAGTTTGGCCCGCGGGCCCCAAATGGGGCTGCCCTGCTCTATAG

>NW_017367106.1:8777-13530_Lates_calcarifer_isolate_ASB-BC8_unplaced_genomic_scaffold,_ASM164080v1_unitig_5775_quiver,_whole_genome_shotgun_sequence

TGCCTTAACACAGTGGTCCCAAACTATGGCCTGCGGGCCGCCTCCACATTGATGCGGCCCCCTGAACAATACCAGAGAGCATTTTGATTTTTTTTCATATATGTGTATTTGTATTTGATAATGAAGTTGGACTTTTGCAATAAAATGTTCCTTAGTTATC
AACTATTTTCATTATTAACTCTCAGTTAGTAACTATTTCATACACGCATTTAACGTGACATTCTGTTCCACCCTTCTGCAGTCTGTGCCTCTATCTGGCAACCCCCAGAAAAAAATCCTAAACCCGCCCTTCTCAAAGAGAGAAGGGAATAATGGCGAAAA
GATTAGATAAGAGAAAAATTGATTCAGAATGCAGGGCATTTAACCTGCAGTGGACAAACGATTATTTTTTTGTTCAATGCAAAGAAAAGGCTGTCTGTCTCATCTGTCACAGGCAGTGGTGGCGGTATTCGAAGAATACAATCTGCGGCGACACTATGAATCC
CGTCACAAAGACAAGTATGATAGCTTGCAAGGTCAAATGCGAGCAGCAGACAAACTCTCAAAGCTAAAAAGTGGACTGTTAGCTCAGCAGAATACATTTGTACGCCAAGCTCAGCTGAACCAGTCATCTGTTCGGGCCAGCTTTCGGGTTGCTCAACTGAT
AGCAAGCAGCGGTAAACCTTTCACTGATGGAGAGTTTGTCAAGAAATGCATGAACGCTGTCACGGAGGTGTGTCCCGAGAAGAAAAATGTCTTTAATGCCGTGAGTCTATCAGTGAGTACAATCACCAGACACATTGAAGAAATCGGGGGTAATGTATA
TGCCCAGCTGCAGCAGAGATGAAAGAATTTGAATTTTTTTCATTAGCACTGGATGAGAGCACGGACGTGCAGGACACCGTGCAGCGGCTCATTTTTATTCGTGGAGTTAGTGCAAACTTTGAGATGTGCAAGGAGCTGGCAGCCCTCCAAAGTCTC
AAAGGGACTACGACGGGGGAGGATATTTTCGGGAAAGTGTACCGAACCATGGAAGAGTTGGACCTGGACTGGTCAAAGCTGGCCAGCATCACAACTGACGGGGCTCCTAGTATGGTGGGCATGTTTCGGGGTCTAATAGGACGCATGAACCGGGA
GATGGAAGAACGGGGTCTCATCGCCCCGCTACAAGTCCACTGCCTAATTCACCAGCAAGCACTGTGCTGCAAAGTGTTGACATGGGATTCTGTCATGAAGGTTGTGGTGTCATGCATAAACTTCATCAGAGCAAAGGGACTTAAACACAGGCAGTTC
CAAGAATTCCTGTCTGAACTGGAACCTGCGCACGGAGATGTGCTGTACTACACAGAGGTCCGATGGTTGAGCCGGGGCAGAGTTTTGAGGCGTTTTTACGAGCTGCTACCTGAAATTAACGCATTTCTTCATTCACAAAACAAAACGGTGCCAGAGC
TGATCGAACCAGAATGGAAATGGCACCTCGCATTTTTAACAGACATGACAGAAATGCTGAACAGCTTTAACTTGCAGCTACAAGGCCAGGGGAAACTCATTTGTGAAATGTATTCCCACATAAAAGCATTTGAGGTGAAACCAGAGCTGCTTTTGGGAC
AAGTGAAAAAGCACAGCTTCATCATCTCCCTGCTACACAAAACCTCTCTGCAGAGAACCCAGAGGTCCCGTTTCCAGCTGAAAAGTGTGTGGAAGCACTGGAAATGCTGAAGGCGGAGTTCGGTGTGCGATTCCGTGAACTACGTGTTTATGCAAA
AGAAATCCGTCTTTTCCAGAACCCCTTTGTTGCCGACATTGATTAAGCCCAGCCTTCTTATCAGTTTGAGTTGGCCGAGTTACAGAACTGTGATGTTCTGAAAGACGCGTTCAAGCCCAACCAGTCTCATTGACTTCTATGCCGCCCTCCCAAACGACAC
GTACCCTAACATCAGAAAACACGCAATGAAGATGTCACACTTTTTGGCAGCACGTATATCTGCGAGCAAACCTTTTCACGCATGAACCTGCTGAAAACTCCAATGAGATCAAGATTGACAGATGAACATCTGCATCAGTGTTGAGACTGGCAGTGAC
TAGAATGGAACCTGACATTCAACTTCTCACCAGCCAGATGCAGGCCCACAGTTCACACTGATGAACATACAAAGGTAAGTTCACTTTTCTGACTTGAATGAGTCATTCTGAGTATAAACAAATATAATCATAATAAAATCTACTGGGATAAAATCCATCTCC
ACAACCATACTGGTAGTGGTACTGGTTGTGCTGTGTAGGTGTTGTATCACTTAGTTCAGTTTCTCAGCCTGCTTGCTTTGTGGTTTTCACAGGGAAGTTGATGAGAGAGAGAGCTGCAAACAGCGATGTCTACAAGCCTACTGGGACTCACAAAAGGAT
TATAAGGAAGGAACACAAGAACTTTGAGAGACTGCTCATATGAGCCATTTAAGTAAGAGATTCTGCTCTGACAACTAAGCTGAACTTGTACCTGTTAAGATTGTGCACGGCACAACAGAAAGTTATGTTCCATGGACTTTTTTTCTGTGAAGAACCCAG
AGAGGGTTATTTGGTTTATTATTATTTCATTAATAGTGTTATTATTTATTTCCTGACTTTTGTTCTGTGAAGAATCCAAAGGGTTATTTGATTGTGCTTTCTGGAAAACAATACATTTTTACGTTAGGCACTCTTGCAATCGTCACACTTTTCTGTTACAAA
CTGACCCCGGCCCCCCATCAGAGAAGGGAAAAATTATGTGGCCCTCACAGGAAAAAAGTTTGGGGACCCCTGCCTTAACAC

>ML142788.1:2414833-2418596_Collichthys_lucidus_isolate_JT15FE1705JMU_unplaced_genomic_scaffold_scaffold815,_whole_genome_shotgun_sequence

GTATACACCAGGGGTCTCAAACTCGCGGCCCGCGGGCCAATTGCGGCCCACAGGACGATAGCTTGTGGCCCCCGCCTTAATATTAAAGTTTAATGTCAGTGCGGCCCGCGAGTTTGATATGTATGGCAGTTTACAGTGTTGTGTGCGGAGCTGAACG
AACCTACCAATCACGGTGGGGTATATGGCTCGCGGGGGCGGGACATCGGTCGGGCTTGACGTAAGCAGAGAAACATTTGTCAATGAAAGATACAGCAGCGTTGCCATGGAGTCCTTTCCTTCCATATATATAAACACACACACGTCAATGGCACTTAG
AAAGTTAATATGAGGTCTCTCTCTCTGACAAAATAAATCAAAGTGATGCGTACGCGGCGGGATAAAGGAAAAGGTAAGGAGCTCAATGCAGCCTAATGTAGTCTGCAGTCACATAGCGACACCTGTCCAGCGGCAATAACAGTCCCCGGTAACCCGGA
CCAATTATAAGGTCGCACCGTTATTTATGGGTTATTATGTTTTTATTTCCATCGCGCTATTTGCATTGTATTCGCATTCCCTCACACAATGAACATTACATATATTTCTATATGATGTAAAAGTAGTCAAAACAAATAAGATCGACAACAGCACGATGTCATTTC
CGGGCTTTTATCCGCCGACCGCCGTGCTGCGGTTACAATAAAAAGAAGCGGGAATGACGTTCTCCATGTAGTGAGACGTTCACTGTCCGTCGGCTAAAGGCTTGATTGAAACGTCAATGCGACAGTGACAACAAGTTGAAATCTATATATTACACAGC
CCCAAACATGTCTTTTTCAAAGCCTGCAGTGAAGAGAAAGGTTGGTGATGAGCACAGACAATTTCAGGAAAAGTGGAAAATGCAATATTTCTTTGTTGAGCATAGGGGCACCCCGACATGTCTGATTTGCACAGAGAAAGTTGCTGTGCACAAGGAAT
ACAATTTGAAACGTCATTACACAACTAGACATGCTGAGGAGTATGAGAAATACCAGGGAGATGAGAGAGCCAACCGGATTGCCAATCTTAAAACATGTCTACTGAGGCAACAAGATTTCTTCAAGAAAGCAACCAAAGAGAGTAATGCAGCAGTCAAAG
CTAGCTACATGGTTAGTGAGATGATTGCTACAGCAGGAAAGCCATTCACAGAAGGTGAATTTGTCAAAAGTGCATATTACAGGCTGCAAGTATAATCTGTCCAGAAAAGAAAGGTCAGTTTAGCAACATCAGCCTTCTGCCAACACCGTGGCAGAGC
GCATTTCTGACCTGTCAAATGACATTTATGACCAACTGTGTGAGAAAGCCAAATGTTTCAGTGCATACTCAGTCGCTCTTGATGAGACCACAGACATCACCGACACTGCCCAGCTCGCAATGTATGTCCGTGGTGTTAATGACAATTTTGAAGTGATGGA
GGAGTTGCTCAGAGTAATTCCAATGCATGGCCAGACCACTGCTCAGGAGATATTTTGCCAGTTGTGTGATGCCATCAAGAATGCCGGTTTGCCATGGAAGAGGTTTGTTGGGATAACAACCAATGGAGCGCTCTCAATGACACGGAGGAAAAATGGAC
TGGTGGCACTTGTTAAAAAAAAACTGGAAGAGGAGGGTGTGGAGGAGGCCATTGCGCTGCACTGCATTATCATCAGCAGGCCCTTTGCAGCAAATGCCTGAAGTTTGACAATGATGTCTGTCGTTGTGAAATGCATTAACCAAATCAAATCCAGG
GGCTTAAAGCACCGAAGGTTCCGTGCTTTTTAGAGGAAATGGAGTCAGAATATGGGGATGTGCTCTACTTCACCGAGGTACGTTGGCTCAGCAGGGGAAACGTATTGAAGAGATTTTTTGAGTTGAGAGCAGAGTGAAAGCCTTCATGGAGAAGG
ATGGAGTGGCTGTTCCTGTGCTAAGTGATCCCAAATGGTTAATGGACTAGCTTTCTTCTTGTTGATATCACACATGAGCTTAATGTACTCAACAAGAAGTTACAAGGCCAGGGGCAACTTGTCAGTGCTGCCTATGACAACGTGAGAGCATTCTCCACAAA
ACTAGTGTTATGGAAAGCCCAGCTCTCTCAGACAAACCTTTGCCATTCCCAGCATGCAAGGCACTGGCGGATGCAGGCACCACCATTCAGTGGTGAGAGAAGTATGTTGACGTCATTTTGAAGCTACAGGAGGAATTTGATCACAGATTTGCAGACTTCA
AGACGCACAGAGCCACATTTCAAATTTTTGCTGACCCCTTCTCCTTGATGTGCAAGATGCCCCTCCTGTGCTTCAAAATGGAGCTCATTGACCTGCAGTGCAACTCTGAACTCAAAGCGAAGTTCAGGGAGGTGAGTGGAAAAGTAGACAAGCTTGG
GCAATTTTTGAGAGAATTGACCCCAGCTTCCCTGAGCTTTCCAGGATGTTCAAGCGGACCATGTGCCTTTTTGGGAGCACATACTTGTGAAAAGCTCTTCTCCACCTTGAACTTCAATAAGTCAAGTATAGGTCCAGACTTACTGATGATCATCTT
CAAGCCATACTAAGAGTCTCAACTGCTTCCTCCCTCCACCCAAATGTGGCTCGGCTATGCGAGAGGAGGCGCTGCCAGGTCTCTGGCAGCAAGAAGTAGGCAAGAGGAGCCATGTTCAGAAGAACAATTCATGTTTTTCAATGTTCCATTCATGTTCA
GGACAATTCATGTTCAGAAGATCAGTTCATGTTCTTCAATGTTCCATTTATGTTCAGGACAATTTATGTTCAGAAGATCAGGATGTTCATTCAATGTTCCATTCATGTTCAGGACAATTTATGTTCAGAAGAAGGTATATAGTCACTAACTAGAGAAATCTAA
TTATTATTATTATTATTTTCATGATTATTATTATTATTTATTTATTTATTTATTAGTGATTGATTTATTTTCTTATTCATTCTTAATTTATTTATTTATTTTTTCATAGATTCTTAATTTATTTATTTTTTCAACTTATTTTGTGTTAAAAATAAAAGATTTGACAAGAATTTTTC
TTGTGGAAAACCTGATGCGGCCCAGCCTCACCCAGACTCTGCCTCCAGCGGCCCCCAGGTAAATTGAGTTTGAGACCCCTGGTATACAC

>NC_049218.1:31353156-31358019_Anguilla_anguilla_isolate_fAngAng1_chromosome_18,_fAngAng1.pri,_whole_genome_shotgun_sequence

CTCTAGGGCAGGGGTGTCAAACTCAAATACACAGTGGGCCAAAATTTAAAACTTGAACAAAGTCGCGGGCCAACATTGAACAAATGAACCTTTTAATATGGACCCAGACAAGTTTTGCTTTAACATTGAATATGGAACAAGCAACGTTTATTACTATACAA
TATATAACAATAGTGCAGACATGCAAAATCGAATTTCAAATAAAAAAACACATCAATGGCATTAATTATTAAATGAAATTTAAATAAAAATCGTATGCCTCTTTTCTATTTGCAGCCTTCTGATTTAAATATCAAAATAAACTTTTTCCACAGGCTAATAATTTAAA
AAATAAAACAACAATAATAAATCAATCAACATTCAAGCCCATGCCTTAAAAAAAAGTGCATAAAGAAAAGGTTAATTATTGCTCAGTTTGCTACACACTGATCTAATCTGATGTGCCCAAGCCAGATACCTGGCATCTCTTCTTGGATGCTAGTAGTTCATC
AATGTCTGGGCTCAGGCTCTGAGCTGAGGAAATCCTCAGTATCGAGCGAAGGTGTTCATCAGTCAGACGTCTCCTGTGAGATGTTTTAGTCATCTTCATCGAGGAGAAAAGTTGCTCACATAGTAAGTGCTGCCGAACATGGAGAGCATCTGAGCAG
CTTGGGTGCGCAGCTGAGGCATTGTGTCATGGATGAACTGTGGAAACTGTGCGGCGCCCACAGCATCATACTTCGACTTCAGTGTGTCATTACACTGGAGTTCAATCAGCTCCATTTGGAGGTTGGTTGGTGCGTTTTCCACATCAACTGCGAAGGG
ATTACTGAGCAGTTCAAACCTACATTTCTGGACATCAAAGTCGGCAAATCGCCGGCTAAACTCAGCGCCAAGTACACTGAGTTTTTCAGTAAACTGTGCGCATGGGAACACGGCGGTAGAGATCTGCGTTTTATGGTTTGACAGCAGGAAAATGGC
CAAGGTTTCCTTGCAGCATCTGATTCTCCCACAGGCACAATTTAGTTTTAAAAGCCCTCACTGCAGCGTACATGTCTGTGATGATGCGCCCCCGCCCCTGAAGCTGCAGGTTCAGCGCATCGAGATGGCTCGAGATGTCACACAGAAAGGCCAGCTC
ACACAGAAACTTTTGCTTCCCGGAGCTCTGATGTGTCCTTCCCTTTGCTTTCCAGAAACTGACATATTTCCTCACGCAGCTCGAAACATCTGTTCAGTACTTTTCCTCGGCTTAACCATCTCACCTCTGTGTGATACGGCACGTCTGCGTATTCCGAACCA
CACTCCTCCAGAAAAGACTTAAACTGGCGGTGATTCAGACCTTTGGCTCTTATAAAGTTAACTACTTGTGTTACTGTGGTCATAACATGTTCCATCTTTAGGGCTTTGCCACACAGTGATTCCTGATGTAGGATGCAGTGATAAACAGTTAGCTCACCTGC
ACAGTTCTCTTCCCGCATCTTCTCCCGAACCCTGCCCACCAGTCCACTCTTTTTACCGCACATCGCTGGCGCACCATCTGTCGTTAATCCCACGAGTTTATCCCACGGCAGCTTTATTTCAGTTACACATTTGGAACCTCCTCAAAGATTTCCTTTCCT
GTGGTTGTGCCATTCATTGATTTTAATCCCAATAGCTCCTCCGTAACACACAGATTTGAGTCCACTCCACGGATGAAGACTGACAGCTGAGCAGTATCAGATGCGTCGCTGCTCTCATCCACAGCAAGGAGAATGCAACGAAATCTTTTCCTTTTCC
ATCAGCTGGTCATACAGATTGGTGGCAAGATCACATGTGCGATCAGCTACTGTGTTTCTGCTCAGGCTCACGTTTGAAAATGCTTGCTTTTTCTCTGGGCACACGAGGTCACAAACTTTCATCATGCACTTTTTCACAAACTCTCCCTCATTAAAGGGCC
GGGCTGATTTTGCGATCTCTGCTGCCACAATATAACTAGCCTTTACAGCAGCCTCACTTTGTGATGTGGCTTTTTTGAACATATTCTGTTTCATCTCCTCTACTTTCTGTCTCCTTTGAGTTATGTCCAGGTCCTTGTACTTGT
CATGGTGTTTCGTTTCATAGTGTCGTCTAATGTTGTACTCCTTAGTTATAGCCACGTGACTCCACAAACAAGACAAACAGGTCTGTCTTTTACATATGTAAACAGATATTCTTCCTCCCACCTGTCCAGAAAGCTCCGGTTTTCTGCCTTTCTTTTCGCCA
TTTTTGGGAAGGGGAAGCACGGTGAAAGTTGTAGCGTGAAGTCGCTATGACTACTGTCCGTTTTTGTTGAACGTTTGGACGTCCTACTGCGTTTCTGCGTCCTGTCCTGATTGACGCGCCAAAACAACAGCAGAGCATTCATCAACCTGAACGAAC
GATTGCAGAACGAACTTGAGGCTAGCTGACTGTCTGCCGTTATGTTTTAAAATATATGATCAAATGTTTACGGAGTTTAATTTCCATCCGTGACTGTACTGTGTCATGTAATCACGTTATATGTATGACATTAATAATACAAGTATGTTCAGTTATCTTCAATTT
ATGTTTCTCGGCAAAACGAATATGCTTAGCTAGCATATCAGCTTGCCAGTGAGCTAGGTAAGCTATCCGTGGTTAGCTCACGATTAGCAATAGGAACTACAGGGGAGAACATCGACTGCACTGGTGGTCAATCAATCAGAGATGTGTAGACTACTGG
TGATTTGACTAGGCTAATTTCATATAACTGTCTGGTAGACCTGCTGTTAACCAAGCAAGTTATCGTCGTAGGTTTTCGCCACAGTCAGTTAATGAGCCATTAACTGCTACTACTCCATCGCCGTTTGTGGTGTTTACTTGCTGGGTGACGCTAGCTGACC
GATCGTTCGCAAGTCACTTTTTACCGAATAAAATTAACACTGTCAGCGGTGATTAACAAATCTACCAAGTATTTTAATAACTGATCTACAGGCGTGAAATATGACAACGCACCTTTGAAGAGCAAGTTTTCCACCTGTTGCATAAAGACAAAAATAATGTAC
ATTGAATTTCTAATTATTATTATTTTCTTGCTAGCTTCTAGCTTTGTCACATTCGTGCAGCATAACCCAGGTAGACATTTACGGAATGTACACGACTAAACAAGCCGTCAAACACGTTTGACAGATGGAATATTTGCCGGTTAGGGTTAGCTAGCTAGTCAAAT
GGCTTGGTAGCCGAAGTTGTGAACTGTTTTAGCATAGGAGCTAGTGGACTACCACATATACTAGCAAATAAGCGTTAGCTGATTACGCTTTCTGCCAACTAATTATTTGGTTAAGTGGGCTAAAGGAAATAGTAAAAATGACTCGGCTACCGCAAAACTTCAG
ATGAGGATTATTTTATGGTCGTCTAGTGCAGCTGTAAATAGCACACGCTATAATGAAACCACTACAAAATGGGAAGCCTGCATTTTCTGACTTCGCACAGGTGGACCGTGGTCGGAGCCGCAATGTCAAGGCCAAGAGACGCCACCTCCCACCCCAGT
GACCACAGATTGTAGCCCCTACTGTAGCT

>R_NC_041352.1:3376807-3381622_Perca_flavescens_isolate_YP-PL-M2_chromosome_22,_PFLA_1.0,_whole_genome_shotgun_sequence

ATATATACCACATGTGTCAAACTCAAGGCCCGCGGGCCACATCCGGCCCGCGAATTAATTATCTTTGGCCCGCATGACAAAATGTATTTACTATCACCACCATAATAGTACAAATCCCACAATGCACTTGCCGTGCGTCGGCACGTCAATCACAGGCTCG
CGAAAGCATGCCCCAATCGTCAACTAACCCTCTCCCCAAAAAATGGCTAAACGAAAGGTGGACTTTGAAAACAGGTGTTTTCAGACAGGTGGGAGGCACAGTATATGTTTGCGGATGTAAAGGGCAAAGCTGTGTGTCTCCTGTGCGGAGACAGTG
TGTCTGTAATGAAAGAATACAACATAAGAAGACACTATGAAACGAAGCACCACGACAAATACAAGCATCTGGACACGACACAAAGGCTACAGAAGGTAGAGAGTTAAAAAGAAGTCTGGTGTCACAGCAGGCTATGTTCACTAAAGCCAAATCACAAA
GCGAGGCTGTTGTTAATGCCAGTTTTATTGTGGCAGCAGAGGTTGCAAAATCAGCCCGGCCCTTTACCGAGGGGGAATTTGTCAAAACTGCATGATTAAAGTTTGCGACGCCGTGTGCCCAGAAAAATGGCAAGCATTTTAAATGTGAGCCTCAGC
CGAAACACTGTTCCTGACCGTGTGCGTCACCTTGCTGCCAATCTCCAACAACAGCTTGTGAGAAAGGGAAACAATTTCATCGCATACTCCCTTGCTGTGGATGAGAGCACCGACACTTCTGATACTGCCCAGCTGTCAATTTTCATCCGTGGAGTGGA
CTCAAGCCTGTGCGTAACAGAAGAGTTTTTGGGATTACAATCAATGCATGGCAACACTATGGGGAAAGATATCTTTGAAGAGGTGTCCAGATGTGATAGATAAAATGGGGTTGCCGTGGGAAACTTGTGGGACTGACAACAGATGGAGCGCCTGCGA
TGTGCGGTCAAAAAAGTGGATTGGTGGGGAGGATCTGAGAAAAGACGCAGGAGGAAAACGTCACAGGTGAGCTGACAGCTTATCACTGCATCATCACCAGGAATCTCTTGTGTGGCAAAGCCTTGAAAATGGAACATGTGATGAGCATCATAACACG
AGCGGTTAACTTTATCAGAGCCAAAGGTTTAAATCACCGCCAGTTCAAGTCTTTTCTGGCGGAGTTAAGTTCAGAATATGGTGACTTGCCCTATCACACAGAGGTGCGATGGCTAAGCCAGGGAAAGGTGCTGGAAAGATTTTTCGAGTTGCGTAAGG
AGATCTGTGAGTTCAAGGAAAGCAAAGGGAAAGACACAACAGAGCTCCGAACTAAAAAGTTTCTGTGTGAAGTGGCGTTTCTGTGTGACATCACGAGCCACCTGAATGCGCTCAACCTGCAGCTTCAGGGACGGGGCCGTGTCATCACTGACATGT
ACGCTACAGTGAGGGCTTTTAAAACCAAGCTGCGCCTGTGGGAGACGCAGATGCTGCAAGAAAACTTGAGCCATTTCCGCACTGCCAAACATGAAGAGCAGGTCACTGCCGCCGTTTTCCAACTGCACAGTTTGCTGAAAAATTCGGCATACT
TGGTGCTGACTTCACACGGCGATTTGCCGATTTTGAAGCCCGAAAAGCAGGTTTGAACTGCTCAGTAATCATTTACAGCCGACATGGAAAGCGCACCATCAAACCTCCAAATGGAGCTGATTGAGCTCCAATGTAGTGACACACTGAAGGCAAAAT
ATGAGTCTGTGGGCGCTGCAAAGTTTCCACATTTCATCCCCGATACAATGCCCCAGAAGCGCTCCCAGGCTGCTCAAACGCTCTGTATGTTGGGCAGCACATACTTGTGTGAACAACTCTTCTCTTTGATGAAGATAAACAAAACTTCACACAGGAGT
CGTCTTACTGATGAACACCTTCACTCAATCCTTAGGATTTCTCAGGTCAGAGCCTCACCCCAGACATTGATGAACTTGATCCAAGATGAGACACCAAGCATCTGGCTCATACCAGCAGTGAGCATTCGAGCAGCAGCAAACGGTGCTGTGCAGCATTTTCA
GTCTTTAATGCAGTTTCATTTTTGCATTTTTTTTCTCTGGCTACTACAGGACATTTGATTTTCCTTTTGAAGTAATAACATTTGGCTGTTGTTTGCCTGTAGAAAATGTTTTCACTTGAAATTACTCTGGAAAAACTGCAGATAGAGCCATAAGAAATTAGTG
AAACTGATTTTATTTATTTATTTATTTATTTTTCTTTATTTCCTAATTAATGTTGTTTATAGGCAATAATATAGGCCTTGTTGGTTTCAGGTTCATAATAATAATAATAATAAGGTTGTTTCAATAAGTTTTCAATAAACGTTGAACCCGTCCGGCCCTTGACTTATA
GCAATTTTTTAATCTCGGCCCACTGTGTATTTGAGTTTGACACCCTTGATATAGAC

>NC_041043.1:272912-277486_Parambassis_ranga_chromosome_24,_fParRan2.1,_whole_genome_shotgun_sequence

CTGTAGTGCAGGGGTGGGCAATTATTTTTTCCATGGGGCCACATGAGAAACAGAAAATATTGTAGAGGGCCGGGCCAAAAGGCTGAACTCAATTCCGCATAATATTAATTTATTTCTTTTTAAAAACCAGTAAATAGCATAGTTTTAACAAAATGAGTAGG
CTATGTGTTATAATTAAGCAATGAAAAAGTGAGGTTGCCTTACAAAAAAATCCTTTATTCAATCAAATTTCCCAAACCAATGGCAAACAAAATATGAACTTTTTTTTTTACTATTCATGTTATATTAATGTTATATTAATGAACATTTTTAGTCACATTCACTCCAA
AATGAGTTTCAAAAACTGCTGGCAAGTGGTTTCTTAGCATTCTAAGGACATCACAATATTGTCTTTGTGCTCCAAACATTAAGCAAGACACATATTATAAGATACTGTCATTGAACATCACTGAACTGTGATTTTGAAAAAAAGGCTTCAAAGAAAGTGTCC
CAGTTCACTGCTAGTTGGTGCTTTTACATGCCTACATTTGTAGCCTAACCACCCGATTTTGTGTTTTTAAGTTGTTTTTTCTTGTTCAGTGAGTAAAATCTAGTCTCTGCTGGGCTTTAACGAGTGCATCAAAGTCAGGTTGAATGTTGAGGTGGAGATG
CGAAGCACAGCTGACAGATGATCATCAGTGAGAGAGCTTCTATGGGTAGATTTTGCAAACTTCATCATTGAGAATGTTGTTCAGATATGTAGGTTGAGCCAAAGAGAACTAACATCTTCTGAGCATGTCTCCTCATGTTTGGAAAGTTCTCTCCTCCTAAG
AGTAGAGTAGTAGAAATCCAGCAGAGATCCTGACTTAAAGTGCTCTGCCAGTACTGCATCAGACTGCAGGTCAATGAGCTCCAGCTGAACATCAGTGGGTGCATTATCCACACTGTAGGTAAAGGGAGAGGATATCATGTGCATTTCATCCTCGATTGTTCT
GAGATCTTCAAATCGCCTTGAAAACTCACCATGCAATGCTCCTAACATGGATGAGTACCTGCGGAGGTGATCAGCTGATGGCGTGACTTCCTTCAGGGTTTGCATGTGAGTAAGAGTGTTGCTCTCCAGTTGGCGTGAAAGAAGCTGCAGCTTGTCA
TGAAAGCCTTCACCAGGCTGTGCATTTGATGAACAAAAAGGCCTTTGCCTTGCAGTTTGGTGTTCAGTTCATTCATCAGTGCAGTCACATCGACAGCAAATGCAAAATCTGCCATCCAGTTCTCATCTGAGAGCTCTGGGATGTCTTTGCCTTTCTTCT
CACAGAACTCTCGAATCTCTGCGTTCAGGTCCCAAACTCTTTCTTTCAGCACTTTGCCCAGGCTGAGCCATCTGACAGCTGTGTGGTAGCCGATGTCACTATGTCAGCTCAGACTCATGCTCCTCTAAAAGTGCGACAAACTGTCTGTGATTCAATGCCCATGCC
CTGATGAAGTTTACTATTTACTAACAACATCAATAACATGGGTAATTTTTAGCACTGACTTGCACAGCACATGTTGGTGAATAATACAATGCAAAAATACCAACTTTTGATCTGCATCAATTCAGTTCATTTATCTTGCATAAGTTTCAAAGTCCAACATTT
TTACCTGTAAGATTGGGCAACCGTCCGTTGACGCCTGCTAGTCTGTCTCCCACTTCAACCCCAGGGTGTCTATGCAAGCATTTACCTCCATAAACAGATCGCTCCCTGTTGTTGTCCCTTTCATCGACCGCATTGCTGCAAGCTCCTCCGTAATCTTG
AAGTCTGTTATTCCACGGACAAATATGAGTAGCTGGGCTGTGTCGCGGACATCACAGCTCTCGTCCAAAGCCAAGGAGAAGAAGTCAAAACCTGCCACTTCGCGATGCAGCTGAAGCTCCAAATTTCCTGCAATTTGCTCGATCCTTCTAGTCACGGT
TCGCCTGGACAGCGGGACTTGTTCAAATGCTCCTTTTTGTCAGGGCATATTAGTGCTGCAGAATCCACCAAACACTCTTTGACAAATTCCCCTCCGAGAATGGGCTTACTGTTTTTAGCGATTTTGTGGGATATCAGAAAACTGGTCGTTTCTTGGTTGCAGC
ATCCCTGGATGTGTGAAGCTTGGTAAAAAAGCCTTGCTGCGTTTGCAGCTTAGCTAGCAACATTTCGATGTCCGTGCCCTTTCAGCATCACTCAAGTTTTTGTATTTCTCCGCGTGTTTCGTCTCGTAATGCCGGTTCAAGTTAGTCCTTAAACACA
GCGATCTGCTCCCCGCAAACTAAACACACGGCTTTACCTCCGACTTCAGTAAATAAGTACTTAGCAGTCCAATTTTGTTTGAAATGCCTGCATTCGGCATCGACCTTTCTTTTTTTTGCAGAAACTGACATTTTTGAGGGCTAAAGTCATCTTGTCACCTG
CTTGTAACAAGGATCTAAACATCTTGATATACGTATGTCATTACGTCATTACGTATGTGAATAAAAACAACTTCAAAATAAAAGCCATGCAGTTTTAGTCCATGCATGAGGCAAAATGCGAAAACATGTTTATTTTATAATTCATTACGTGGGCCAGTCAGAG
TCAGCCAAAGGGCCGTATGCGGCCCGCGGGCCGTACAATGCCCAGGTCTGCTGTAGTG

>NC_053389.1:550631646-550636274_Bufo_bufo_chromosome_1,_aBufBuf1.1,_whole_genome_shotgun_sequence

CTCTAAAGCAGGGGTGTCAAACTCAAATACACAGTGGGCCAAAATGTAAAACTTGAACAAAGTCGCGGGCCAGCATTGAACAAATGAACCTTTTAACATGGACCCAAACAAAATTTGCTTTAACATTGAATATGGAACAAGCATATTCATTTATTAAATAAG
ATTTAAATAATAATAAATAATAATAAAAAAAAAAAAAAAAAACAATAATAAAATCAATCAACCATTCAAGCCCATGTCTTGGAGTAGCAAGAAAAAGTGCATAAAGAAAACATTAATTATTGCTCAGTTTGCTGCACACTGATCTAATCTGATGTGCCCAAGCCA
GATACCTGGCATCTCTTCTTGGATGATAGTTCATCAATGTCTGGGCTCAGGCTTTGAGCTGAGGAAATCCTCAGTATCGAGCGAAGGTGTTCATCAGTCAGACGTCTCCTGTGAGATGTTTTGGTCATCTTCATCGAGGAGAAAAGTTGCTCACATAGA
TAAGTGCTGCCGAACATGGAGAGCATCTGAGCAGCTTGGGTGCGGAGCTGAGGCATTGTGTCAGGGATGAACTGTGGAAACTGTGCGGCGCCCACAGCATCATACTTTGACTTCAGTGTGTCATTACACTGGAGTTCAATTAGCTCCATTTGGAGGT
TGGTTGGTGCGTTTTCCACATTAACTGCGAAGGGATTACTGAGCAGTTCAAACCTACATTTCTGGACATCAAAGTCGGCAAATCGCCGGCTAAACTCAGCGCCGAGTACACTGAGTTTTTCAGCAAACTGTGCGCATGGGAACAAGTTGGTAAAGAAC
TGCGTTTTCATGGTTTGGCAGCAGGGAAAATGGCCAAGGTTTCCTTGCAGCATCTGATTCTCCCACAGGCACAGTTTAGTTTTAAAAGCCCTCACTGCAGCGTACATGTCTGTGATGATGCGCCTGCGCCCCTGAAGCTGCAGGTTCAGCGCATCGA
GATGGCTCGAGATGTCACACAGAAAGGCCAGCTCACACAGAACCTTTTTCTCCCGAAGCTCTGCTGTGTCCTTCCCTTTGCTTTCCAGAAACTGACATATTTCCTCATGCAGCTCAAAACATCTATTCAGTACTTTTCCTCGGCTTAACCATCTCACCTC
TGTTTGATTCGGCACGTCTGAATACGCCGAACCACACTCCTCCAGAAAAGACTTAAACTGGCGGTGATTCAGACCTTTGGCTCTTATAAAGTTAACTACTTGTGTTACTGTGGTCATAACATGTTCCATCTTTAGGGCTTTGCCACACAGTGATGCCTGA
TGTATGATGCAGTGATAAACAGTTAGTTCACCTGCACAGTTCTCTTCCTGCATCTTCTCCCGGACCCTGCCCACCAGTCCACTTGTTTTACCGCACATCGCTGGCGCACCATCTGTCGTTAATCCCACGAGTTTATCCCACGACAGCTTTATTTCAGTTA
CGCATTTGGAAACCTCCTCAAAGATTTCCTTTCCTGTGGTTGTGCCATGCATTGATTTGAATCCCAATAGCTCCTCCGTAACACACATATTTGAGTCCACTCCACGGATGAAGACTGACAGCTGAGCAGTATCAGATGCGTCGCTGCTCTCATCCACAG
CAAGGGAGAATGCAACGAAATCTTTTCCCGTTTCCAAAAGCTGGTCATACAGATTGGTGGCAAGATCACATGTGCGATCAGCTACTGTGTTTCTGCTCAGGCTCACGTTTGAAAAGGCTTGCTTTTTCTCTGGGCACACGATGTCACAAACTTTCAACA
TGCACTTTTTCACAAACTCTCCCTCATTAAAGGGCCGGGCTGATTTTGCGATCTCTGCTGCTACAATATAACTAGCTTTTACAGCAGCCTCACTTTGTGATGTGGCTTTTTTGAACATATTCTGTTGTGAAACCAAACTTCTTTTCATCTCCTCCACTTTCT
GGCTCTTTTGAGTCATGTCCAGGTCCTTGCACTTGTCATGGTGTTTTGTTTTTTATAGTGTCGTCGAATGTTGTACTCCTTAGTTATAGCCACGTTGACTCCACAAATCAGACAAACAGGTCTGTCTTTTACATATGTAAACAGATATTCTGTCTCCCACCT
GTCCAGAAAGTTCCTGTTGTCTGCCTTTCTTTTTGCCATTTTTGGGAAGGGGTTCCACAGTGACAGTTGTAGCTTGAGATCGCTATGACTTCTGTCACACAGGAGAGGGCGTTTCTGGGACCAGTCCTTATTGACGCGCCAAAACATCAGCAAGGCAT
TATGGGATTTGTAGTATTAGCGATGAATGAATGCGCTGTATAATACCGGCGGGCTAGCTCTAGTAGTCATTTGGTATAGCCTCGCGGGCCAAATATAATTACACAGCGGGCCAAATTTGGCCCGCGGGCCAGAGTTTGACACCTATGCTCTAAAG

>NC_014776.1:120642620-120647768_Anolis_carolinensis_chromosome_1,_AnoCar2.0,_whole_genome_shotgun_sequence

TCAGGGGTGCTTTGATTGTGTTTTGGTGAGGTTTAGGCAAAAGCAGAGTTTTACCAATATGGGCACTTGTGAGGTTGAAATGATGTGGACTGAGGAGGCTGCATTGATGGACACTGATCAGACTGCATTGGTGGGCAGTGCAGTCTGTATGTCTCTGT
ATGTGCAGTTATTGTTGGTATATATTAGTATTTACTAATAGCTATTTCCCTAGGAAACAATGATGTCAAGAAAGAGAAAAATTGACTCGGAGTGTAGGATATTCAAAGAACAGTGGACTTATGATTACTTTTTCGTGAATTACAAGGAAAGAGCTGTGTGTTT
GATATGCCAGAATATAGTGTCTGTGTTCAAAGAATACAATATGCGCCGACACTATCAAACCCAACATAAAGACAAATATGATTGTTTGGTTGGAGAAGTGAGAAAAGATAAAATATTAAAACTGAAAAATATATTGACAACTCAGCAAAATACTTTTGTGAAG
CAGAAGCAGCTAAATATTTCATCACTGCGAGCAAGTTTTCAAGTTGCCAAGCTAATAGCGTGCACTGGTAGACCATTTGTGGAAGGAGAGTTTGTTAAAGAATGCCTTCTTTCTGTTGCCAAAGAGATGTGTCCAGAGAAGGCTGATTTATTTAGTACAG
TGAGTCTATCAGGATCTACAATTACACGAAGGATTGAAGAAATGGGAGACAATTTGCATCAGCATTTGCAAAACTCTGCAAAAAAACTTTCCTATTTTTCCTTGGCACTTGATGAAAGCAATGATGTTCGTGATTCTGCACAACTTCTAATTTTTATTCGTG
GGACAAATGACTATTTCGAAGTCACAGAAGAGCTTGCTGCACTGCAAAGCATCAAAGGAACAACTACAGGAGAGGATATCTATGAAAAGCTTTGCCAAACTGTGAATGATTTGGAGCTGGACTGGGCTAAACTAGCCAGTGTGACAACTGATGGTGCT
CCTAGCATGGTGGGGTCTAAGAAAGGAGTAATTGCTCGCATTAACCAAGAGATGGACAAACATAACCATTCTCATCCAATAGCCATACACTGCCTCATCCACCAACAAGCGCTGTGTAGTAAGTCACTGAAGTGGGACTCTGTCATGAAAATTGTGGTAT
CTTGTGTTAACTTCATTAGAGCTAATGCACTAAACCACAGACAATTTCAGGAATTTCTGTCTGAGCTAAATGCTGCCTATGAAGATGTTCTGTACCACACAGAAGTCCGTTGGCTGAGTCGAGGGAGAGTTTTGAAACATTTCTATGACTTACTTCCACAG
ATTACAGCTTTTCTGCTTTCAAAAAACAAAGAAGTACCAGAGCTCAATGATGCAGAATGGAAATGGCACCTCGCCTTTCTGACAGATATAACAGAGCTACTCAACAGTTTCAATGTGCAACTTCAAGGAAAGGGGAAGCTCATCTGTGATATGCAATCAC
ATGTGAAAGCATTTGAAGTAAAATTAGGCCTCCTCATCAAACAAGTAAAGGAGGAAAATTTCTGCCATCTCCCCACAACTCAAAGTCTGTCAGCGGAAAAACCATTGATTGCATTCCCAAACAAAACATGTGTGGATTCACTGGAAAGGTTGCAAAAGG
AGTTCCAATTTAGATTTAAAGAGCTTCATCTCCATGAACAGGACATACAGCTTTTCCGTAACCCATTTTCTATTGACATTGAAAATGTGGATACAATTTACCAAATGGAACTGGCTGAACTTCAGAATTGTGACTCTCTGAAAGACGCATTCAAGCCAAGAA
GCCTTCCTAATTTCTATGCATCTCTCCCCTCTGAAACATATCCTAATCTCAGGAACCATGCACTCAAAATGGCAACCATCTTTGGCAGTACTTATGTCTGTGAACAGACTTTTTCCAGAATGAAACATCTGAAATCTCCAACCAGATCTAGATTAACTGACG
CACACTTGCATCACTTATTACGGCTAGCAGTGACAAATATGGAACCGGACATTGACTATCTCATTAGTCAAAAGCAGGCTCATAGTTCCCATTGACATACTGGTGGATTTGTTGGTTTAACTTTACTTGTTCTTTAAATTTTAAATATTGTATTTGTTCCCATT
TTGGTTTTTTTTTTTACCTCAAAATAAGATATGTGCAGTGTGCATAGGAATTTGTTCATAGTTTGTTTGTTTTTTAAAAAAAAATCTATAGTCCGGCCCTCCCAAGTTCTGAGGGACAGTGAACTGGCCCCTTGTTTAAAAAGTTTGGGGACCCCTG

>NW_017711359.1:254870-262837_Alligator_mississippiensis_isolate_KSC_2009_1_unplaced_genomic_scaffold,_ASM28112v4,_whole_genome_shotgun_sequence

CCATGTAAAGCAGGGTGGGCAAAATACAGTCCGCGGCCCACTAGGCACTTTCATCTGGCTTGCGGAGCCCCTCCAAAATGCAGGTAATGGCAGCAGCCTGTGTGAAAGCCTCCAGCGGCTGCAGGAGCATCTGGCTGTTTGCTTGGGCTCTGGTT
GTGAAGCAACAAACCCTGACCCCAACCTGAGCCAGCTGGCCTTGCTGGGCCCCTAGCTCAGGCTAGGTCTGCAGCTTGAGGGGGGCAGGATGGGCTGGGCCAGATCGTGTGGCACCAGCGAGAGGAACAGGTGCCTGGGCTGGCTTCTGCATT
GCTGCGCAGGACTGGACAGGATCGGTCAGCTCCTGCATCACAGGTGAAGGGAAGCAGAGGTTGTGCCAGCTTCTGTTGCACTCCCAGAACAGGACAGGCTGGGCTGGCCACGCGGGACAGTCTTGGCTGGGCTGGCGCAGGTGAGGGGAAGT
GGCAGCTTGGCTGGCTTCTACTGCACCATGCCATGCTGTGTGGAATGGGACAGGCTGGCACCGGTGAGGGGAAGAGGGGGCTGGGCTGGCTTCTCCTGTGCTGTGCCAGGTCCTGCAGCACTGAATGAGCTGAGCTGGTGCCGGTGAGGAGAA
CCTGGGGCTGCGCTGGCTTCTGCCTTGCTGTGCGGGGCGGGACGGGCTAGGCTGGGCTAGTCTCAGCTGGCTTTTGCCACCACATGGGGTGGGCTGGGCTGGGCTGAGCTGAGTCGTGCCATGTGAGACGGGCCCAGCCAGTGCAGGTGAGG
GGAACAGGGGCTTAGCCAGCTTTTGCTGCCATGCAGGACGGGCCTGGCTAGGCCAGTGCAGGTGAGGGGAACAAGGGGTTTGGCCAGCTTTTCCCGCCACGTGTGGCAGGATGGGCTGGGCTGGATCCTGCCATGCGGGACAGGCCTGGTATG
TGGCACTGGTGAGGTGAAGGGAAGGCTGGGCTGGCTTCTGCTGCGCTGTGCAGGTCCCGGCCCTTGCCTTGCAGCTGAGACCCCTGTGCTCCTGGGAGGAGATGGGCCTGGCCATGAGATCCCAACTTTGTAGCTGGGAACCAGGTGCTGCTT
GCTCCATGTGAAGGTCCAGCCTTGCAGCTGGCAACCACTTGCTGCAGCCAGGGCCTGACCGGCAGGGTTAACTCCTTGCTGTGCTGTGCAGGTCCTGGCCTTGCAGTCAGGAACTGTTTGTCATTGGAGCCATGGGCAAACTGGGGTGGGCTGG
GCCCTGGTAGGTACATAAGCAACAGCCATGCTGTGTCCTCACAGGAGGCCCAGTACTCCTACTGCAGTTACTGGCCCACCTACCCTTGGTGGCAAGTTGCCTGTGACTTCCAGGCCTGTTGCTATGTGCTGGATTGCAGGTCCTACATGGTTACCTG
GCCAAGGTCCCTCAATCACCTGGGCATGGCTGTTATAACAAGGACATCTATGTGACTGGTAAGGGCAGAGGCCAGAGCAAGGGCAATGCCCTGAGATGTTATGGTTAAGTGGCAAACTTAGAAAAATGTAAATGGATATTTTTAAATGATATTTATATCCC
TAATATCACCCCTCCACAGAAAAATGATTTGGCTATGAGCACATCAAAGAAGAGGAAGGTAGACACTGAACGCCGAGTATTCAACAAAAAATGGACCTCTAAATATTTTTTTACCGAAATGGGCAAAAAAGCTTTTTGCTTAATTTGCAAAGAAAGTCTGG
CTGTGTTTAAAGAATACAATTTGAATCGGCATTTTGAAACCAAGCATGCATCCAAATATGATAAACTGTCTGCCCAAGAAAAAACAAAGAAAGCAGAGGAGATGGTTGCTGCAATGCAGAAAGAGCAAAGTTTTTCCCCAAAAGCATTGGAAGTACGGG
ATGGAATTATGAGAACGAGTTATGAGATGGCACACATCATTGCCAAGAAGAGCAAACCATTCTCTGAAGGAGAATTTGTAAAGGAATGTATGCTTAAGTCTGCTGCGATTTTGTGCCCAGACAAGAAGGAGCAGTTTGAAAACGTTAGGTTGTCAAGGC
GGACTATTGTGAGACGTGTTGAAGATATCTCTGAGAATCTGCACCAGCAGTTGAAAAGTAAGGTAAATGATTTCTCATATTTTGCACTGGCACTCGATGACAGTTGTGATGTCAAAGGCACAGACCAGTTGTTGATTTTCATTCGAGGAATCACTGAGAA
TTTTGAGCTCACAGAAGAATTGGCATTGATACAGTCAATGAAAGACACAACAACTGGAAAAGATTTATTGGAGGCAGTAGATCAATGCGTGTCAAAACTGGGAACGGATTGGAAAAAATTGGTGAGTGTGACCACCTATGGAAGCCCAAACCTGGCAG
GCAAAGACGTTGGATTGCTCAAAATAATTCAAGACCAAGTCAAAGAAACCGATCCAGAACAGAAAATTATTTTATTACATTGTATTATTCATCAAGAGGTCCTCTGTAAAAGTGTTCTAAAACTAAGCACTGTTGTGGACACAGTTACAAAAGTGGTCAGCT
ACATTCGAGCAAGAGGATTAAACCACAGGCAATTTGCAACTCTGCTTGAGGGGTCTGAATCTGAGCACACAGATGTTCTGCATGATACCAGTGTAAGTTGGCTTAGTTTGGGTAACATTTTGAAAAGAGTGTGGGAACTGAGAGCCGAGATTGGTTTAT
TTTTGAACATGAAAGAAGATTTGGATTTCCCACAGCTGAATGATGCAGAATGGCTCTCTGATTTTGCATTTGCCATTGATGTCACGGGCTACATGAATGATCTGAATTCACGACTGCAAGAGAAGGGGCTTTTTGCTCATGAATTGCATTCCAACATCAA
GTCATTTATGATGAAGTTACTCCTTTTCTCCCGTCAGATAGGAAACAAAGATTTCAGCCATTTTCCTACATTGCAGCAGGTTACTGTTTCTGATCAAAACCTAAAAAAATACGTGTCATTACTAGTAGACTTGCATGCTGAATTTTCTTGCCGAGTTGAGGA
TTTTCAAATGATAGAAAAAGACTTGTCTTTGGTCTCCTCACCTTTCACATTTAATGTTGACAGTGCTCCATGTGACCTCCAACTTGAGCTTATTGATCTGCAGTATGATGCGTTGCTTGCAGAGCAATTCCAAGCAGTGCCACTTCCTAGCTTTTATGCCT
CTCTCAATGAACAGAAGTTTCCAAAGATCAAGACTCATGCCCGGAAGATGCTTGTGCTGTTTGGATCGACATACGTGTGCGAGCAGACACGTTCTGTAATGAAAGTCAACAAATCGAATCTTAGATCTTCGATGAGTGGGGACCACCTTGCAGCAGTG
CTCCGCATTGCAACAACAGAGATGACGCCTGACTTTGATTTTCTGGTTAACGCACATCAGAGGCTTCATTCTTCACCTTGAGTTCCTTTGTCTTTGTTAATCACCTTTACATATGCTACTCAAATTATTTTTTAAATAAAATTAAAATCTGTTATAGTAGATAT
TTAATTTTTTCGTACACAATACTTGATCTATACTTGGTTAAAATGTTTTGTTTTGAAAGATTTTGTGTCTGCAGTCCTTGACTCAATTGGTTTGGTTCTGTTCTCGGGGAGGTCTGATGCCCCTGAATCCGCACCGATTGGTTGAGGGTGATGGGGCAGGA
TGTTAGAGGGTCACAACCCCTTCCTACCTGATAAATTGTGCCTGTGCCTCATGGCCTATAGGGCAGAGCTCTTGGGGCCCCGGGAGCAGGCCATGCACTGGGAGGGTGGGCCCTGTGCAGGGGCAGTGTTTCCCAGTCAAGGGGTAGAACTTCTG
ATGTCTGGGATCATGCAATGGGCAAGGGATTCTGGGACAGATGTGAGTAGGGCAAGGTTTGGTTTTGGATGGGTGGATCTTATGACATGGCCCATGTCACAGGGTGTAATTTCCGGCCCCAAGGCCACCCCCAGTAAAAGGAGCACTTCTGGGGGC
AAGAAGAGACACTCCCAGCGACAAAGATCAGGGGTTAGGGGAGGGATTTCTGGTCCCTAGAGGGCAACCAGGGGGTGGGGCAACTGTTAAGGGGCGGGGCTACCCATGTGGCCCAAGAAGGCTCACCAAAACTCGTGAAGCGACCCTCCAGCT
GAAACAATTGCCCACCCCTGCCATAAAA

>Malaclemys_terrapin_terrapin_MDXI01005171.1:64662-69038(+)

CCCTAGAGCACATGTGTCAAACTCAAGGCCCGCGGGCCACGTCCGGCCCGCCATACAATTATATCCGGCCCGCGAAATCGTTTTATATATCTGTTTTTAATGGCCCTGTGATATGACGCCCCAATAACACACACTACAAATCCCATGATGCAGTGCAATT
GCCGCCAACGGCAAGCCGCGGTTCAAGTTCGCGGTCTGTTGATGTATACAACGCTAATCTCAAATCAAAGCTAGACCCCAACAATGGCCAAGAGAAAAATTGATTCTGAAAACCGAGGCTTTCAAAGCCGGTGGGAGAATGAGTATATGTTTACTGAA
ATTGCAGGTAAACCAGTGTGTCTCCTTTGCGGGAGTAATATCGCTGTAATGAAGGAGTATAACCTAAGACGGCACTACGAGACGAAACATGAGAACAAATTCAAAAACCTGAGCGCAGGACAGAAGCTACAAAAGGTAGAGGAGTTGAAGAAGAATTT
GACATCCCAGCAGACGTTTTTCACCAAAGCAAATCACAAAGTGAAGCTGCTGTGAAAGCAAGTTTCATTGTGGCCGAAGAGATCGCCAAATCAGGACGGCCGTTTACCGAGGGGGAATTCGTAAAGAATTGTATGATGAAAGTGTGCGACGTCCTTT
GTCCAGATAAAACGCGAGCGTTTGCAAATGTAAGCCTCAGCAGAAACACTGTTGCTAATCGGGTTTGTGAGATGGCGACTGATTTGAAAACACAGTTGATTGAAAGAGCAAAAGATTTTGTTGCATACTCCCTTGCCGTGGATGAAACTACTGACGCG
ACTGACACTGCACAGCTGGCGATATTTATCCGTGGTGTGGATTCCAATTTGTGCGTAACACAGGAAATACTGGACATTAAATCGATGCACGGGACAACGAAAGGAGAAGACATCTTTGGAAATGTATTTCAAAGTGTAACCGACATGAAACTGCCGTGG
GAAAAACTCATTGGACTTACAACAGATGGCGCACCTGCTATGTGTGGTGAAAAAAATGGACTGGTGGGAAGAATGCGCTCAAAGATGCGGGAGGAGAACTGTGCCGGTGAGTTGACAGTGTATCACTGCATCATACACCAGGAATCGCTGAGTGCTA
AAGTCCTAAAAATGGATCATGTGATGAACACTGTAACACAAACCGTCAACTTTATCAGAGCCACGTTTAAATCACCGCCAATTCCAGTCTTTTCTGCGGGAAATAGATAGCGAGTTTGGCGATATGCCATATCATACGGAGGTCCGGTGGCTAAGTCGGG
GAAAAGTTCTCAAAAGACACTTTGAGCTGCGAGAGGAAATCTGCCAGTTCATGGACAGTAAGGGGAAAGACTGCACAGTTCTGCGGGATGAAAAGTGGAAATGTGAGTTGGTGTTCCTGGCTGACATAACGTCGCATCTTAGCGCTTTAAACCTTCA
ACTCCAGGGACGGGAGCACATAATAACCGATATGCATGATGCAGTGAAGGCATTTCAAGTGAAGCTGCGCTTATGGGAGACACACATGCACCAATGCAACTTGTCTCACTTTCCCTGTTGCCAAGTAATACGGAACCAAGAAAGTGCCACAGTTTTCC
CAAATGCCACCTTTGCTGAAAAACTCAGCGCGCTGCGCACTGAGTTCGCACGGCGCTTCAGTGACTTTGAGGCACAGAAAAGTAACTTCGAGCTGCTTCGCAACCCATTTGCAGTCGATGTGGAAACCGCACCTGTAGAAATGCAGATGGAGCTGA
TAGAACTGCAATGTAACGGGACACTGAAGGCAAAGTACGACACTGCGGGGCCAGCACAGTTCACTCGCTTCATTCCTGAAGCGATGCAACAGCTCCGCCAACATGCGGCTCGAATCCTGTCCATGTTTGGCAGCACATATCTGTGCGAGCAGCTGTT
CTCTGTGATGAAAATTAACAAAACGTCACACAGGAGTCGCCTCACTGATGAACACCTGCAATCGATCCTGAGAATCTTCACAACACAGAACCTAACCCCAAACATAAACGAACTTGTTGCAAAGAAAAGACTCCAAGTATCAGGCTCTGACTAAATAGG
ACAAGAAAATGGAATGTTATGATTTGTTATGATTATACTTTTGCTTTAAATTCAATTTTTTATTTATATTTTCAGATTTTTTAATATTCCAGCATGTACAGATTTTGAATGTATTGTATTGACAGGATATTTTTTTATGAAGAGCAAAATATTTTAAGTTGAAATGTAT
TTATTTTGGAATGATATCCTGTATTTTGGTTCATATTAATGGTTAAAAAACATAAATATTTAGTCTGTTAAATAAATGGTTATACTGTTCGGCCCGCGAGTTTTGAGTTTTGGCCCCCTGTGCAATTGAGTTTGACACCCCTGCCCTAGAG

>NC_048299.1:253020000-253034478_Trachemys_scripta_elegans_isolate_TJP31775_chromosome_2,_CAS_Tse_1.0,_whole_genome_shotgun_sequence

TGCTAGATCACATGTGTCAAACTCAAGGCCCGCGGGCCACATCCGGCCCGCCATACAATTATATCCGGCCCGCGAAATCGTTTTATATATCTGTTTTTAATGGCCCTGTGATATGACGCCCCAATAACACACACTACAAATCCCATGATGCAGTGCAATTG
CCGCCAACGGCAAGCCGCGGTTCAAGTTCGCGGTCTGTTGATGTATACAACGCTAATATCAAATCAAAGCTAGACCCCAACAATGGCCAAGAGAAAAATTGATTCTGAAAACCGAGGCTTTCAAAGCCGGTGGGAGAATGAGTATATGTTTACTGAAAT
TGCAGGTAAACCAGTGTGTCTCCTTTGCGGGAGTAATATCGCTGTAATGAAGGAGTATAACCTAAGACGGCACTACGAGACGAAACATGAGAACAAATTCAAAAACCTGAGCGCAGGACAGAAGCTACAAAAGGTAGAGGAGTTGAAGAAGAATTTGA
CATCCCAGCAGACGTTTTTCACCAAAGCAAAATCACAAAGTGAAGCTGCTGTGAAAGCAAGTTTCATTGTGGCCGAAGAGATCGCCAAATCAGGACGGCCGTTTACCGAGGGGGAATTCGTAAAGAATTGTATGATGAAAGTGTGCGACGTCCTTTGT
CCAGATAAAACGCGAGCGTTTGCAAATGTAAGCCTCAGCAGAAACACTGTTGCTAATCGGGTTTGTGAGATGGCGACTGATTTGAAAACACAGTTGATTGAAAGAGCAAAAGATTTTGTTGCATACTCCCTTGCCGTGGATGAAACTACTGACGCGACT
GACACTGCACAGCTGGCGATATTTATCCGTGGTGTGGATTCCAATTTGTGCGTAACAGAGGAAATACTGGACATTAAATCGATGCACGGGACAACGAAAGGAGAAGACATCTTTGGAAATGTATTTCAAAGTGTAACCGACATGAAACTGCCGTGGGAA
AAACTCGTTGGACTTACAACAGATGGCGCACCTGCTATGTGTGGTGAAAAAAATGGACTGGTGGGAAGGATGCGCTCAAAGATGCGGGAGGAGAACTGTGCCGGTGAGTTGACAGTGTATCACTGCATCATACACCAGGAATCGCTGAGTGCTAAA
GTCCTAAAAATGGATCATGTGATGAACACTGTAACACAAACCGTCAACTTTATCAGAGCCCACGGTTTAAATCACCGCCAATTCCAGTCTTTTCTGCGGGAAATAGATAGCGAGTTTGGCGATATGCCATATCATACGGAGGTCCGGTGGCTAAGTCGG
GGAAAAGTTCTCAAAAGACACTTTGAGCTGCGAGAGGAAATCTGCCAGTTCATGGACAGTAAGGGGAAAGACTGCACAGTTCTGCGGGATGAAAAGTGGAAATGTGAGTTGGCGTTCCTGGCTGACATAACGTCGCATCTTAGCGCTTTAAACCTTC
AACTCCAGGGACGGGAGCACATAATAACCGATATGCATGATGCAGTGAAGGCATTTCAAGTGAAGCTGCGCTTATGGGAGACACAAATGCACCAATGCAACCTGTCTCACTTTCCCTGTTGCCAAGTAATACGGAACCAAGAAAGTGCCACAGTTTTC
CCAAATGCCACCTTTGCTGAAAAACTCAGCGCGCTGCGCACTGAGTTCGCACGGCGCTTCAGTGACTTTGAGGCACAGAAAAGTAACTTCGAGCTGCTTCGCAACCCATTTGCAGTCGATGTGGAAACCGCACCTGTAGAAATGCAGATGGAGCTG
ATAGAACTGCAATGTAACGGGACACTGAAGGCAAAGTACGACACTGCGGGGCCAGCACAGTTCACTCGCTTCATTCCTGAAGCGATGCCGCAGCTCCGCCAACATGCGGCTCGAATCCTGTCCATGTTTGGCAGCACATATCTGTGCGAGCAGCTG
TTCTCTGTGATGAAAATTAACAAAACGTCACACAGGAGTCGCCTCACTGATGAACACCTGCAATCGATCCTGAGAATCTTCACAACACAGAACCTAACCCCAAACATAAACGAACTTGTTGCAAAGAAAAGACTCCAAGTATCAGGCTCTGACTAAATAG
GACAAGAAAATGGAATGTTATGATTTGTTATGATTATACTTTTGCTTTAAATTCAATTTTTTATTTATATTTTCAGATTTTTTAATATTCCAGCATGTACAGATTTTGAATGTATTGTATTGACAGGATATTTTTTTATGAAGAGCAAAATATTTTAAGTTGAAATGTA
TTTATTTTGGAATGATATCCTGTATTTTGGTTCATATTAATGGTTAAAAAACATAAATATTTAGTCTGTTAAATAAATGGTTATACTGTTCGGCCCGCGAGTTTTGAGTTTTGGCCCCCTGTGCAATTGAGTTTGACACCCCTGTGCTAGAT

>NC_030681.2:91452362-91466350_Xenopus_tropicalis_strain_Nigerian_chromosome_5,_UCB_Xtro_10.0,_whole_genome_shotgun_sequence

CTCTAAACCATATGTGTCAAACTCAAGGCCTGCAGGCCACATCCGGCCCGGTGTGTAATTATATCCGGCCTGCAAGATCATTTTATTTATTATTGTTATTAATGGCCCGGCGATATGAAGCACTGGTAACACAATAAACTACAGGTCCCATAATGCAGCGC
TTCAGCTGCCTTGCCGAACACTTCCCGCATTAATCAAGTCTAGCTTATGATGCTGCAAGTTATTGCGAAGCTAGCCCTCACGATGCCGAAGAGAAAAGTTGATTCTAAAAACAGAGCCTTTAAAAACCGATGGGAGGCTGAGTATATGTTTACTGACATT
GCCGGTAAACCCCTGTGTCTCATTTGTGGAGCTAATGTGGCTGTAATTAAAGAATTTAATTTAAGACGGCACTATGAGACAAAACATCAGGATAACCTGAAAGACCTGAATGCAGAGCAGAAGATACAGAAAGTAGAAGAGTTAAAGAAGAATCTGACAC
TTCAGCAAACGATTTTTACCCGTGCAAAATCAGAAAGTGAAGCTGCTGTGAAAGCAAGTTTTATCGTGGCAGAGGAGATTGCCAAATCAGCCAGGCCATTTACCAAGGGAGAATTTCTGAAGAGCTGCATGATCAAGGTGTTTGACGTCTTATGTCCAG
ACAAAAAGCAGATGCTGGCAAATGTAAGCCTGAGTAGAAATTCGATTTTTTGATCGAGTTTGTGAGATGGCCACTGATTTAAGAACACAGTTGAGTGAAAGAAGCAAAGACTTTATTGCATACTCTCTTGCTGTGGATGAAAGTACTGACATGACTGATA
CTGCACAGCTGGCCATCTTAATCCGTGGAGTGGACTCCAATTTGCGCATTACAGAGGAAATAATGGACATTAAATCGATGCACGGGACAACGAGTGGAAAAGACATTTTTGAAAATGTATGTCAAAGTATAACTGACATGAAACTGCCCTGGGACAAATT
TATTGCACTTACAACTGATGGAGCACCATCTATATGTAGTGAAAAAAGTGGACTAGTGGGAAGGATGCGAGTAAAGATGCAGGAGGAGAACTGTACTGGTGAGTTAACGGCATATCACTGCATCATACACCAGGAAGCACTATGTGGCAAAGTCCTGAA
GATGGACAATGTAATGAGCACTTTAACACAAACTGTAAACTCCGAGCTAAAGGTTTAAAACACCGGCAATTTCAGTCCTTTATGCGGGAGATAGATTCAGAGTTTGCTGACATTCATTATCATACAGAGGTGCATTGGCTAAGTCGGGGAAAAGTTCTCA
ACAGAGTTTTTGAGCTCAGCAACGAAATCTGTCAGTTGATGGACAGTAAAGGAAAAGACTCCACCAATTTTAGGGATAAAAAGTGGAAATGCGAGTTGGCATTACAACTGACATAACAGCTCATCTCAACACCTTAAACCTCCAGCTCCAGGGATGTGA
CCGCATGATCACTGACATGTATGACGCAGTGAAGGCATTTCAAGTGAAGCTGCTTTTATGGGAGACACAAATGTACCAGTACAACTTGCCCCACTTTCCCTGTTGCCAAGTAATGTTGAACCAAGTCGGCACAACGGTGTTCCCAAATATGCACTTTGC
TGATAAACTGAGCGCACTGCGCACGGCGCTTTGGCGACTTTGAAGAACAAAAAAAGAATTTTGAGTTGCTTCGCAACCCATTTGCCGTCGATGTGGAAACTGCACCTGTGCAGATTCAGATGGAGCTAATTGAGCTGCAGTGTAATGGTACACTGAAG
GCAAAGTACGACACTGCAGGGCCTGCACAGTTTATTCACTCCATTCCCGCAGAAATGCCCCAGCTCCGTCTACATGCAGCTCGAACCTTGTGCATGTTTGGTAGCACATATCTGTGTGAGAAGCTCCTCTCAGTGATGAAGACTAACAAAACAGCACA
CAGGAGACATCTCACTGATGAGCACCTGCAGTCCATCCTGAGAATCTCCACACAGAACCTCACACCAAACATAAACAACTTGTTCGTTTGCCAGGCGTCCAGCTCTGATAAAATGACATAAGAGCAAAGACAACTGAATTTTAGTGTGTTCAATAAATGT
TTATCCTGTTCGGCCCGCGACCTAAAGTGTGTTTTGGATTTTGGCCCCTGTGCAATTGACACCCTTGCTCTAAAC

>NW_022603349.1:42820-57984_Etheostoma_spectabile_isolate_EspeVRDwgs_2016_unplaced_genomic_scaffold,_UIUC_Espe_1.0_scaffold00006261,_whole_genome_shotgun_sequence

TTCTAGACCACATGTGTCAAACTCAAGGCCTGCGGGCCACATCCGGCCCGCCATACAATTATATCCGGCCCGCGTGATCATTGTATATATCTGTTGTTAATGGCCCTGTGATATGACGCCCCAAACTACAAATCCCATGATGCAGTGCAATTGCCGCCAA
CGGCAAGCCGCGGTTCAAGTTCGCGGTCTACAACGCTAATATCAAATCAAAGCTAGACCACAACAATGGCCAAGAGAAAAATGTATTCTGAAAACCGAATCTTTCAAAGCCGGTGGGAGAATGAGTATATGTTTACTGAAATTGCAGGTAAATTAGTGTG
TCTCCTTTGCGGGAGTAACGTCGCTGTAATGAAGGAGTATAACCTAAGACGGCACTACGAGACAAAACATGAGGACAAGTTAAAAAACCTGAGCGCAGGACAGAAGCTACAAAAGGTAGAGGAGTTGAAGAAGAATTTGACATCCCAGCAGACGTTTT
TCACCAAAGCGAAATCACAAAGTGAAGCTGCTGTGAAAGCAAGTTTCATTGTGGCCGAAGAGATCGCCAAATCAGGACGGCCGTTTACCGAGGGGGAATTCGTGAAGAATTGTATGATGAAAGTGTGCGACGTCGTTTGTCCAGATAAAACGCGAGC
ATTCGCAAATGTAAGCCTCAGCAGAAACACTGTTGCTAATCGGGTTTGTGAGATGGCGACTGATTTGAAAACACAGTTGATTGAAAGAGCAAAGGATTTTGTTGCATACTCCCTGGCCGTGGATGAAACTACTGACTCGACTGACACTGCACAGCTGG
CGATATTCATCCGTGGAGTGGATTCCAATTTGTGCGTAACAGAGGAAATACTGGACATTAAATCGATGCACGGGACAACGAAAGGAGAAGACATCTTTGGAAATGTATTTCAAAGTGTAACCGACATGAGACTGCCATGGGAAAAACTTGTTGGACTTA
CAACAGATGGCGCACCTGCGATGTGTGGTGAAAAAAAATGGACTGGTGGGAAGGATGCGCTCAAAGATGCGGGAGGAGAACTGTGCCGGTGAGTTGACAGTATATCACTGCATCATACACCAGGAATCGCTGAGTGCTAAAGTCCTAAAAATGGATC
ATGTGATGAAACCTGTAACACAAACCGTCAACTTTATCAGAGCCCACGGTTTAAATCACCGCCAATTCCAGTCTTTTCTGCGGGAAATAGATTGCGAGTTTGGCGATATGCCGTATCATACGGAGGTCCGGTGGCTGAGTCGGGGAAAAGTTCTCAAAA
GACACTTTGAGCTGCGAGAGGAAATCTGCCAGTTCATGGACAGTAAGGGGAAAGACTGCACAGTTTTGCGGGATGAAAAGTGGAAATGTGAGTTGGCGTTCCTGGCTGACATTACGTCGCATCTTAGCGCTTTAAACCTTCAACTCCAGGGACGGGA
GCACATAATAACTGATATGCATGATGCAGTGAAGGCATTTCAAGTGAAGCTGCGCTTATGGGAGACCCAAATGCACCAATGCAACTTGTCTCACTTTCCCTGTTGCCAAGTAATACTGAACCAAGAAAGTGACACAGTTTTCCCAAATGCCACCTTTGCT
GAAAAACTCAGCGCGCTGCGCACTGAGTTCGCCCGGCGCTTCAGTGACTTTGAGGCACAGAAAAGTAATTTCGAGCTGCTTCGCAACCCATTTGCAGTCGACGTGGAAACCGCACCAGTAGAAATGCAGATGGAGCTGATAGAACTGCAGTGTAAC
GGGACACTGAAGGCAAAGTACGACACTGAGGGGCCAGCACAGTTCACCCGCTTCCTTCCTGAAGCGATGCCGCAGCTCCGCCAACATGCGGCTCGAATCCTGTCCATGTTTGGCAGCACATACAGTGGTGCTCAAAAGTTTACATACACATGCTTAA
GTTGACTAAAAAGAGGAATAAAAAATCATGTTTTGGAAATTTATTTTAATACCTAAATTAAAAAATGAGGTAAAATCCAACCTTTAAGGACACCAATTTTCTTTGTGAATGAATAACGTATTGTAAATAAATAAATGTTCTTATTTAAAATACAGGGGTCATAAGT
ATACATACCCCTATGTTAAATTCCCATAGAGGCAGGCAGATTTTTATTATTAAAGGCCAGTTATTTCCTGGATTCAGGATATTATGCATCCTGATAAAGTTCCCTTGGCCTTTAGAATTAAAATAGCCCCACATCCTCACATACTCTTCACCATGCTTAGAGAT
AGGCATGGTTTTATTTCAGTTAGACTAATACCTGGTTTGATTTGCATTGAGAGATGATTTTATAGAAAGTATCCCATGCCTATCTCTAAGCATGGTGAAGAGTATGTGAGGATGTGGGGCTATTTTAATTCTAAAGGCCAAGGGAACTTTATCAGGATGCATA
ATATCCTGAATCCAGGAAATAACTGGCCTTTAATAATAAAAATCTGCCTGCCTCTATGGGAATTTAACATAGGGGTATGTATACTTATGACCCCTGTATTTTAAATAAGAACATTTATTTATTTACAATACGTTATTCATTCACAAAGAAAATTGGTGTCCTTAAA
GGTTGGATTTTACCTCATTTTTTAATTTAGGTATTAAAATAAATTTCCAAAACATGATTTTTTATTCCTCTTTTTAGTCAACTTAAGCATGTGTATGTAAACTTTTGAGCACCACTGTATGTGTGCGAGCAGCTGTTCTCTGTGATGAAAATTAACAAAACGTCA
CACAGGAGCCGCCTCACTGATGAACACCTGCAATCCATCCTGAGAATCTCCACAACACAGAACCTGACCCCAAACATAAACCAACTTGTTGCAAAGAAAAGACTCCAAGTCTCAGGCTCTGACTAAATAGGACAAGAAAATTGAATGTTATGTTTTGTT
ATGATTTAACTTTTACTTTAAATTACATTTTTCATTTACATTTTCAGATTTGTTATATTCCAGCATGTACAGATTTTGAATGTATTGCATTGACAGGATATTGTTTTATGAAGAGCAAAATATTTTAAGTTGAATTTATTTATTTTGGAATGATATCCTGTATTTTGAT
TCATATTAATCGTTAAAAAACACCAATATTTAGTCTGTTAAATAAATGTTAATACTGTTCGGCCCTCGACATTAAGTGTGTTTTGAGTTTTGGCCCCCTGTGCAATTGAGTTTGACACCCCTGTTCTAGAC

>Scleropages_formosus_LR584089.1:6216100-6227904

AATTAAATCAGGGGTGTCAAACTCAATTGCACAGGGGGCCAAAATCCAAAGCACACTTTAGGTCGCGGGCCGAACAGGATAACCATTTATTGAACACACTAAAACTAAACTTTTAAAACTTTAAAACTTAACTTTTTTGAACATAAAAATGAATAAAAACAG
ACAGGAATATTATTCCGGAATAAATCAACTCAAACCTTAAATAACTTATAATATTTTGCTCTCCATAAAAATATATGCTGTCTAAATTATACAAATTCAAAACATGAACATGCTGCATAACAAAACCTGGAAATATAAATAAAATTTGTGCTTTTCAGCAATAACAA
ATCAAATCATTCGGTTGTCTTTGCTCTTATGTCATTTTATCAGAGCTGGACGCCTGGCATCTTTTTTTGGCAACAAGTTCGTTTATGTTTGGTGTGAGGTTCTGTGTTGTGGAGATTCTCAGGATGGACTGCAGGTGCTCATCAGTGAGACGACTCCCGT
GTGCTGTTTTGTTAGTCTTCATCACTGAGAAGAGCTTCTCACACAGATATGTGCTACCAAACATGCACAAGGTTCGAGCCGCATGTAGACGGAGCTGGGGCATTTCTGCGGGAATGGAGTGAATAAACTGTGCGGGCCCTGCAGTGTCGTACTTTGC
CTTCAGTGTGCCATTACACTGCAGCTCAATCAGCTCCATCTGAATCTGCACAGGTGCAGTTTCCACATCGACGGCAAATGGGTTGCGAAGAAACTCAAAATTCTTTTTTTGTTCTTCAAAGTCACCAAAGCGCCGTGCGAACTCAGTGCGCGGTGCGC
TCAGTTTATCAGCAAAGTGCGTATTTGGGAACACCGTTGTGCCGACTTGGTTCAACATTACTTGGCAACAGGGAAAGTGGGGCAAGTTGCACTGGCGCATTTGTGTCTCCCATAAAAGCAGCTTCACTTGAAATGCCTTCACTGCGTCATACATGTCA
GTGATCATGCGGTCACGTCCCTGGAGCTGGAGGTTTAAGGCGTTGAGATGAGCTGTTATGTCAGCCAGAAATGCCAACTCGCATTTCCACTTTTCATCCCTAAAATTGGTGGAGTCTTTTCCTTTACTGTCTATGAACTGACAGATTTCGTTGCTGAGC
TCAAAAACTCTGTCGAGAACTTTTCCCCGACTTAGCCAACGCACCTCTGTATGATAAGGAATGTCAGCAAACTCTGAATCTATCTCCCGCATAAAGGACTGAAATTGCCGGTGATTTAAACCTTTAGCTCGGATAAAGTTTACGGTTTGTGTTAAAGTGCT
CATTACATTGTCCATCTTCAGGACTTTGCCACATAGTGCTTCCTGGTGTATGATGCAGTGATATGCCGTTAACTCACCAGTACAGTTCTCCTCCTGCATCTTTACTCGCATCCTTCCCACTAGTCCACTTTTTTCACTACACATAGATGGTGCTCCATCAGT
TGTAAGTGCAATAAGTTTGTCCCAGGGCAGTTTCATGTCGGTTATACTTTGACATACATTTTCAAAAATGTCTTTTCCAGTCGTTGTCCCGTGCATCGATTTAATGTCCATTATTTCCTCTGTGACGCGCAAATTGGAGTCCACTCCACGGATGAAGATGG
CCAGCTGTGCAGTATCAGTCATGTCAGTACTTTCATCCACAGCAAGAGAGTATGCAATAAAGTCTTTGCTTCTTTCACTCAACTGTGTTCTTAAATCAGTGGCCATCTCACGAACTCGATCAGCAATCGTATTTCTACTCAGGCTTACATTTGCCAGCATC
TGCTTTTTGTCTGGACATAAGACGTCAAACACCTTGATCATGCAGCTCTTCAGAAATTCTCCCTCGGTAAATGGCCTGGCTGATTTGGCGACCTCCTCTGCCACGATAAAACTTGCTTTCACAGCAGCTTCACTTTGTGATTTTGCACGGGTAAAAAACA
TCTGCTGAAGTGTCAGATTCTTCTTTAACTCTTCTGCTTTCTGTATCTTCTGCTCTGCATTCAGGTCTTTCAGGTTATCCTGATGTTTTGTCTCATAGTGCCGTCTTAGATCAAATTCTTTAATTACAGCCACATTAGCTCCACAAATGAGACACACGGGTTT
ACCGGCAATGTCAGTAAACATATACTCAGCCTCCCATCGGTTTTTAAAGGCTCTGTTTTCAGAATCAACTTTTCTCTTCGGCATCGTGAGGGCTAGCTTCGCAATAACTTGCAGCATCATAAGCTAGACTTGATTAACGCGGGAAATGTTCGGCAAGGCA
GCTGAAGCGCTGCATTATGGGATCTGTAGTTTATTGTGTTACCAGCGCTTCATATCGCCGGGCCATTAATAACAATAATATATAAAATGATCTCGCGGGCCGGATATAATTACACGCCGGGCCGGATGTGGCCCGCGGGCCTTGAGTTTGACACATATGA
ATTAAAT

>Thalassophryne_amazonica_LR722981.1:85676192-85687982

CCCTAGAACATATGTGTCAAACTCGAGGCATGCGGGCCATATCCGGCCCGGCGTGTGATTTTATCCAGCTTGCGAGATCATTTTATATATTATTGTTATTAATGGCCCGGCAATATGAAGCGCTGGCAATAAAGACAATAAACTACAGATCCCATAATGCAG
CGCTTCAGCTGCCTTGCCAAATGCTTCACGTTCATTTAGCCTAGCTTATGATGCTGCAAGTTATTGCGCCCGAGTTATTGAGCCCTCACGATGCCGAAGAGAAAAGTTGATTCTGAAAACAGAGCCTTTAAAAGTCGATGGGAGGCTGAGTATATGTTTA
CTGACATTGCCGGTAAACCCGTGTGTCTCATTTGTGGAGATAATGTGGCTGTAATTAAAGAATTTAATCTAAGACGGCACTGTGAGACAAAACATCAGGATAACCTGAAAGACCTGAATGCAGAGCAGAAGATACAGAAAGCAGAAGACTTAAAGAAGA
ATTTGACACTTCAGCAGACATTTTTTACCCGTGCAAAATCACAAAGTGAAGCTGCTGTGAAATCAAGTTTTATCGCGGCAGAGGAGATCGCCAAATCAGCCAGGCCATTTACAGAGGGAGAATTTCTGAAGAGCTGCATGATCAAGGTGTTTGACGTCT
TATGTCCAGACAAAAAGCAGATTCTGGCAAATGTAAGCCTGAGTAGAAAGATGATTGCTGATCGAGTTTGTGAGATGGCCACTGATTTAAGAACACAGTTGAGTGAAAGAAGCAAAGACTTTATTGCATACTCTCTTGCTGTGGATGAAAGTACTGACAT
GACTGATACTGCACAGCTGGCCATCTTCATCCATGGAGTGGACTCCAATTTGCGTGTTACAGAGGAAATAATGGACATTAAATTGATGCACGGGACAACGACTGGAAAAGACATTTTTGAAAATGTATGTCAAAGTATAACCGACATGAAACTGCCCTGG
GACAAACGTATTGCACTTACTACTGATGGAGCGCCATCTATGTGTAGTGAAAAAAGAGGACTAGTGGGAAGGATGCGAGTAAAGATGCAGGAGAACTGTACTGGTGAGTTAACTGCATATCACTGCATTATACGCCAGGAAGCACTATGTGGCAAAGTC
CTGAAGATGGACCATGTAATGAGCACTGTAACACAAACCCTAAACTTTATCCGAGCTAAAGGTTTAAATCACCGGCAATTTGCAGGAGATAGATTCAGAGTTTGCTGACATTCCTTATCATACAGAGGTGCGTTGGCTAAGTTGGGGAAAAGTTCTCAGC
AGAGTTTTTGAGCTCAGCAACAAAATCTGTCAGTTCATGGACAGTAAAGGAAAAGACTCCACCAATTTTAGGGATGAAAAGTGGAAATGCAAGTTGGCATTTCTGGCTGACATAACAGCTCATCTCAACGCCTTAAACCTCCAGCTCCAGGGACGTGA
CCGCATGATCACTGACATGTATGACGCAGTAAAGGCATTTCAAGTGAAGCTGCTTTTATGGGAGTCACAAATGCACCAGTGCAACATGTCCCACTCTCCCTGTTGCCAAGTAATGTTGAACCAAGCGGGCACAACGGTGTTCCCAAATATGCACTTTGC
TGATAAACTGAGTGCACTGCGCACTGAGTTTGCACGGCGCTTTGGTGACTTTGAAGAACAAAAAAAGAATTTGGAGTTGCTTCGCAACCCATTTGCCGTCGATGTGGAAACTGCACCTGTGCAGATTCAGATGAAGCTGATTGAGCTGCAGTGTAATG
GCACACTGAAGGCAAAATATGACACTGCAGGGCCCCCACAGTTTATTCACGCCATTCCTGCAGAAATGCCCCAGCTCCGTCTACATGTGGCTCGAACTTTGTGCATGTTTGGTAGCACATATCTGTGTGAGAAGCTCTTCTCAGTGATGAAGACTAACA
AAACAGCACACAGGAGTCATCTCACTGATGAGCACCTGCAGTCCATCCTGAGAATCTCCACAACACAGAAACTCACACCAAACATAAAGGAACTTGTTGCCAAAAAAAGATGCCAGGCATCCAGCTTTGATAAAACGACATAAGAGCAAAAAAAACTG
AATGATTTGATTTGTTATTGCTGAAATTTTATTTATATTTCCAGGTTTTGTTATGCAGCATGTTCATATTTTGAATTTGTATAATTTTGACAGGATATATTTTTATGGAGAGCAAAATCTTTTGGGATATTTTAGTAAGTTTAAATTTAAGTTTATTTTTTATATAAAAT
AATGTAAGAGTAAAGACATTTGAACGTTTACTTTTTTCTAACTTGTATAATTTAGACAGGATATATTTTTATGGAGAGCAAAATATTAGAAGTTATTTAAGGTTTAAGTTGATTTATTCCAGAATAATAGTCCTGTCTGTTTTTATTCATATTCATGTTCAAAAAAG
TTAAGTTTTAAAAGTTTTAAAGTTTAATGTATTCAATAAATGTTTATCCTGTTCGGCCCGTGACCGAAAGTGTGTTTTGGATTTTGGCCCCCTGTGCAATTGAGTTTGACACCCCTGCCCTAGAA

>Syngnathus_acus_LR594601.1:130790-143594

GTCTGGAGCAGGAGTGTCAAACTCAATTGGACAGGGGGCCGAAATTCAAAACACACTTCAGGTCGCGGGCCGAACAGAACAAACATTTATTGAACACACTAAAACTAACTTTTAACATAAATATAAATACAAACGGACAGGAATGTTATTCTGGAATATAT
AAACTTAAACTTTAAATAACTTAAAATATCTTGATCTCCATAAAAATATACCCAGTTAAAATTATACAAGTTAGAAAAATATCTCCTGTCAAAATTATACAAGATAGAAATATGAACATGCTGCAAAACAAAACAAAACCTGGAAATATAAAAACAATTTGTGCTTT
TCAGCAATAACAAATAATGAATCAAAACATTCAGTTTTCTTTGCTCTTATGCCATTTTATCAGAGCCGGATGATTGGCATCTTTTTTTGGCAACAAGTTGGTTTATGTTTGGTGTGAGGTCCCGTGTTGTGGAGACTCTCAGGATGGACTGAAGGTGCTCA
TCAGTGAGACGACTCCTGTGTGCTGTTTTGTTTGTCTTCATCACTGAGAACAGCTTCTCACACAGATATGTGCTACCAAACATGCACAAGGTTCGAGCCACATGTAGACGGAGTTGGGACATTGCTGCGGGAATGGAGTGAATAAACTCTGTGGGCCC
TGCAGTGTCGTACTTTGACTTTAGCGTCCCATTACACTGCAGCTCAATCAGCTCCATCTGAATCTGAACAGGTGCAGATTCCACGTCGACGGCAAATGGGTTGCGAAACAACTCGAAATTCTTCTTTTGTTCCTCAAAATCACCAAAGCGCCGTGCGAA
CTCCGTGCGCAGTGCGCTCAGTTTAACAGCAAAGTGCGTATTTGGGAACACCGTTGTGCCGACTTGGTTCAGCATTACTTGGCAACAGGGAAAGTGGGACAAGTTGCACTGAAGCATTTGTGTCTCCCATAAAATCAGCTTCACTTGAAATGCCTTCA
CTGCGTCATACATGTCAGTGATCATGCGGTCACGTCCCTGGAGCTGGAGGTTTAAGGCATTGAGATGTGCGGTTATGTCAGCTAGAAACGCCAACTCACATTTCCACTTTTCATCCCGTAAAACGGTGGAGTCTTTGCCTTTACTGTCCATGAACTGAC
AAATTTCGTTGCTGAGCTCAAAAACTCTGTTGAGAACTTTCCCCCGACTTAGCCAACGCACCTCTGTATGATAAGGAATGTCGGCAAACTCTGAATCTATAGTCCGCATAAAGGACTGAAATTGCCGGTGATTTAAACCTCTACTTCGGATAAAGTTTACG
GTTTGCGTTACAGTGCTCATTACATGGTCCATCTTCAGGACTTTGCCACACAGTGCTTCCTGGTGTATGATGCAGTGGTATGTTGTTAACTCACCAGTACAGTTCTCCTCCTGCATCTTTTCTCGCATCCTTCCTACTAGTCCACTTTTCTCACCGCACAT
AGCCGGTGCTCCATCAGTTGTAAGACCAATAAGTTTGTCCCAGGGCAGTTTCATGTCGGTTACACTTTGACATACGTTTTCAAAAATGTCCTTTCCGGTCGTTGTCCCGTGCATCGGTTTAATGTCCAATATTTCCTCAGTAACACGCAAATCGGAGTCC
ACCCCACGGATGAAGATGGCTAGCTCTGCAGTGTCAGTCATGTCAGTACTTTCATCCATAGCAAGAGAGTATGCAATAAAGTCTTTGCTTCTTTTACTCAACTGTGTTCTTAAATCAGTGGCCATCTCACAAACTCGATCAGCAACCGTATTTCTACTCAG
GCTTACATTTGCCAGCATCTGCTTTTTGTCTGGACATATGATGTCGCACACCTTGACCATGCAGCTCTTCAGAAATTCTCCCTCTGTGAATGGCCGGCCTGCTTTGGCGATCTCCTCTGCCACGATAAAACTCGCTTTCACAGCAGCTTCACTTTGTGAT
TTTGCACGGGTGAAAAACGTCTGCTGGAATGTCAGCTTCTTCTTTAACTCTTCTACCTTCTGTATCTTCTGCTCTGCATTCAGGTCTTTCAGGTTATCCAGATGTTTTGTTTCATAGTGCCGTCTTAGATTAAACTCTTTCAATACAGCCACATTAGCGCCA
CAAATGAGACAAACGGGCTTACCGGCAATGTCAGTAAACATATACTCAACCTCCCATCGGTTTTTAAAGGCTCTGTTTTCAGAATCAACTTTTCTCTTCGGCATCATGAGGGGCTAGCTTCGCAATAACTTGCAGCAACAAGCACTTGACCTGATTGCCG
CGGAAAAACGCTCGGCAAGGGAGCGAAAGCACTGCATTATGGGATCTGTAGTGTTTTTTCTTTTTTTTTTTTTTTTCATATCGTCGGGCCATTAGTAACAATAATATAAAATGATCTCGCGGGCCGGATATAATTGCATGCCGGGCCGGATGTGGCCCGCG
GGCCTTGAGTTTGACACATATGGTTTAGGG

>NC_049698.1:506477-509261_Scophthalmus_maximus_isolate_AOZZmale01_ecotype_atlantic_turbot_chromosome_11,_ASM1334776v1,_whole_genome_shotgun_sequence

TAGAGCAGGGGTGTCAAACTCAATTGCACATGGGGCCAAAACTCAAAGCACACCTCAGGTCGCGGGCCGAACAGGATAAACATTTATTGAACACCCTTAAACTACACGTTTGAACATGAATATGAATAAAACAGACAGGAATATTATTTCAGAATAAATAA
ACTTGCACTTTAAATAAAATAAAATGTATTTTGCTCTCCATAAAAATATATCCTGTCAATAATTATACAAGTTCGAAATATGAACATGCTGCAAAAAACCCCTGAAAAAAATAAATAAAAATAGTGCTTTTAAGTAAACGTTAAAATAAATGAAAACATGTTTCTTT
GTTCTTATGCCATTTTGTCAGAGCAGGATGTTTGGCATCTTTTTTTGGCAGTAAGATCGTTTAGGTTTGGTGTGAGTTCCTGTGCTGTGGAGATCCTCAGGATGGACTGCAGGTGTCCGTCAGTGAGACGACTCCTGTGTGCTGTTTTGTTCATCTTCA
TCACTGAGAACAGCTTCTCACACAGATATGTGCTACCAAACATGCACAAGGTTTGAGCAGCATGCAGACGGAGCTGGGGCATTGTTTCAGGAATTGAGCGAATAAATTGTGCGGGCCCTGCAGTGTCGTACTTCGCCTTTAGTGTCCCATTACACTGC
AGCTCAATCAGCTCCATCTGAATCTGTACAGGAGCAGTTTCCACGTCGACGGCAAACGGGTTGCGAAAGTCACCAAAGCGCCGCGCGAACTCAGTGCGTAGTGCGCTCAGTTTGTCAGCAAAGTGCGTATTTGGGAACACCGTTGCGCCGACTTG
GTTAAACATTACTTGGCAACAGGGGAAGTGAGGCAAGTTGGACTGGCGCATTTGTGTCTCCCATAAGAGCAGCTTCACTTTAAATGCCTTCACCGTATCGTACATGTCAGTGATTATGCGGTCACGTCCCTGGAGATGAAGGTTCAAGACGTTGAGAT
GAGCTGTTACGTCAGCCAGAAACGCCAGCTCACATTTCCACTTTTCATCCCGCAAAACTGTGGTGTCTTTTCCTTTACTGTCCATGAACTGACAGATTTCCTTGCTGAGCTCAAAAACTCTATTGAGAACTTTTCCCCGACTCAGCCAGCGCACCTCTG
TATGATAAGGAATGTCGGCAAACTCTGAATCTATTTCCCGCATAAAAGACTGAAATTGCCAGTGATTTAAACCTTTAGCTCGGATAAAGTTTACGGTTTGCGTTACAGTGTTCATTACATGCTCCATTTTTAGGACTTTACAACACAGCATTTCCTGGTGTAT
GATGCAGTGATACGCTGTTAGCTCACCAGTACAGTTCTCCTCCTGCATCTTTGCTCGCATCCTTCCCACCAGTCCAACTTTTTCACTGCACATCGCCGGTGCTCCGTCTGTTGTAAGTCCAATAAGTTTGTCCCAGGGCAGTTTCATGTCAGTTATGCTT
TGACATACGTTTTCAAAAATGTCTTTTCCTGTCGTTGTTCCATGCATCGATTTAATGTCCAATATTTCCTCTGTAACACACAAACTGGAGTCCACTTCACGGATGAAAATGGCCAGCTGTGCAGTGTCCGTCATGTCGGTACTTTCATCAACAGCAAGAGA
AAATGCAGTAAAGTCTTTGCTTCTCTCACTCAACTGTGTTTTTAAATCAGTGGCCATCTCACAAATCCGATCAGCAACCGTATTTCTACTCAGGCTTACATTTGCAAACATCTGCTTATTTTCTGGACACAAGACATCGCACACCTTCATCATGCAGCTCTT
CAGAAACTCTCCCTCGGTAAATGGACGGGCTGATTTGGCTATCTCCTGTGCCACGATAAAACTTGCTTTCACAGCAGCTTCACTTTGGGATTTCGCTCTGGTGAAAAACGTCTGCTGAAATCTCAGATTCTTCTTTAACTCTTTTACTCTCTGTATCTTCT
CCTCTGCATTCAGGTTTTGCAGCTCCTGATGTTTTGTCTCGTAGTGCCGCCGTATATTAAATTCCTTAATTACAGCCACATTAGCTCCACAAACGAGACACACAGGTTTACCAGCAATGTCAGTAAACATGTACTCAGCCTCCCATCGGTTTTGAAATGCT
CTGTTTTCAGAATCAACTTTTCTCTTCGCCATTGTGAGGGGCTAGCTTCGCAATAACTTTTCAACGGGGTTGTAGCAGCCGGTTGTAGCACAAAAGAAGCTGACTTGATTGACGCGGAAAAGTTCCCGGGTATCCGAGTGTCCGTTACTTGGTTACTG
AAGCACTGCATTATGGGATCTGTAGTTTGTGTATTGTCAGCGCTTCATGTCACCGGGCCATAATAATAGATCTATATAAAATTATCTTGCAGGCCGGATATAATTGCACGCCGGGCCGGATGTGGCCCGCGGGCCTTGAGCTTGACATATATGACGTAGAG

Table S7: Sample of misannotation events

Species Name/GenBank assembly ID	Gene ID	Chromosome	Gene Coordinates	Gene description	TE overlap coordinates	TE match	100% overlapped TEs (excluding simple repeats):Total protein-coding gene count
**Garter Snake/GCA_001077635.2**							0.0010
OldhAT1 (hAT-6_DR)	XM_014072166.1_cds_2_0_NW_013660042v1_65137_f	Scaffold2362	65136-68622	Thamnophis sirtalis zinc finger, BED-type containing 4 (ZBED4), transcript variant X2, mRNA	67656-67826	hAT-6_DR	
OldhAT1 (hAT-6_DR)	XM_014072165.1_cds_2_0_NW_013660042v1_65137_f	Scaffold2362	65136-68622	Thamnophis sirtalis zinc finger, BED-type containing 4 (ZBED4), transcript variant X1, mRNA	67656-67826	hAT-6_DR	
hAT-16_Crp	XM_014064415.1_cds_1_0_NW_013658662v1_52202_r	Scaffold982	52201-54022	Thamnophis sirtalis general transcription factor II-I repeat domain-containing protein 2-like (LOC106547299), mRNA	52765-53985	HAT3_MD	
hAT-16_Crp	XM_014074848.1_cds_6_0_NW_013661234v1_33330_r	Scaffold3554	33329-35276, 35102-35206	Thamnophis sirtalis uncharacterized LOC106555913 (LOC106555913), mRNA	34045-34308	HAT3_MD	
hAT-16_Crp	XM_014065111.1_cds_5_0_NW_013658737v1_257155_r	Scaffold1057	258093-258145	Thamnophis sirtalis general transcription factor II-I repeat domain-containing protein 2-like (LOC106547845), mRNA	257154-258998	hAT-11_CPB	
Gypsy-5_AMi-I	XM_014074749.1_cds_6_0_NW_013661182v1_21107_f	Scaffold3502	21106-21704	Thamnophis sirtalis uncharacterized LOC106555836 (LOC106555836), mRNA	21126-21687	Gypsy-3_LaAg-I	
Gypsy-5_AMi-I	XM_014055008.1_cds_1_0_NW_013657942v1_659303_r	Scaffold262	659302-661390	Thamnophis sirtalis uncharacterized protein K02A2.6-like (LOC106540031), mRNA	659985-660347	Gypsy-3_LaAg-I	
Gypsy-5_AMi-I	XM_014058689.1_cds_1_0_NW_013658158v1_231451_f	Scaffold478	231702-231894, 233747-233793	Thamnophis sirtalis uncharacterized protein K02A2.6-like (LOC106542846), mRNA	231450-231911, 233271-234768	Gypsy-3_LaAg-I	
Gypsy-5_AMi-I	XM_014056157.1_cds_2_0_NW_013658001v1_791275_f	Scaffold321	792851-792982	Thamnophis sirtalis uncharacterized protein K02A2.6-like (LOC106540872), mRNA	791274-793709	Gypsy-3_LaAg-I	
Gypsy-5_AMi-I	XM_014057610.1_cds_4_0_NW_013658085v1_131307_r	Scaffold405	131861-131941	Thamnophis sirtalis uncharacterized LOC106542012 (LOC106542012), mRNA	131306-132086	Gypsy-3_LaAg-I	
**Lizard/GCA_000090745.2**							0.0022
Neptune1_Ac	XM_016991679.1_cds_1_0_chr2_184740785_r	2	184740784-184741767	Anolis carolinensis uncharacterized LOC107982479 (LOC107982479), mRNA	184741259-184741363	Neptune-3_ACar	
Neptune1_Ac	XM_008108518.2_cds_0_0_chr4_29871353_f	4	29871375-29871876	Anolis carolinensis uncharacterized LOC103278512 (LOC103278512), mRNA	29871352-29872654	Neptune-3_ACar	
Neptune1_Ac	XM_016996278.1_cds_1_0_chrUn_GL343299_678979_r	Uncharacterised	679309-679413	Anolis carolinensis uncharacterized LOC107983282 (LOC107983282), mRNA	678978-680031	Neptune-3_ACar	
Neptune1_Ac	XM_016996337.1_cds_1_0_chrUn_GL343309_1013021_f	Uncharacterised	1013262-1013323	Anolis carolinensis uncharacterized LOC107983290 (LOC107983290), mRNA	1013020-1013351	Neptune-3_ACar	
**Western clawed frog/GCF_001663975.1**							0.0086
BEL-4-I_XT	XM_031899359.1_cds_1_0_chr3_135678301_f	3	135678300-135684231	Xenopus tropicalis uncharacterized LOC116409808 (LOC116409808), transcript variant X3, mRNA	135678378-135678542, 135679094-135684200	BEL-10_XT-I	
BEL-2-I_XT	XM_004912313.3_cds_1_0_chr4_79669121_f	4	79669120-79673593	Xenopus tropicalis uncharacterized LOC101731929 (LOC101731929), transcript variant X1, mRNA	79669291-79673549	BEL-13_XT-LTR	
Polinton-2_XT	XM_031898444.1_cds_1_0_chr3_2131755_f	3	2131754-2136857	Xenopus tropicalis uncharacterized LOC116409665 (LOC116409665), mRNA	2133200-2136850-	Polinton-2_XT	
BEL-4-I_XT	XM_031897261.1_cds_1_0_chr2_156755135_r	2	156755134-156759487	Xenopus tropicalis uncharacterized LOC101730439 (LOC101730439), transcript variant X1, mRNA	156755218-156758693, 156759245-156759409	BEL-10_XT-I	
BEL-4-I_XT	XM_031893363.1_cds_1_0_chr9_7020578_f	9	7020577-7024930	Xenopus tropicalis uncharacterized LOC101730954 (LOC101730954), mRNA	7020655-7020819, 7021371-7024846	BEL-10_XT-I	
BEL-4-I_XT	XM_031891904.1_cds_1_0_chr8_123361984_r	8	123361983-123365880	Xenopus tropicalis uncharacterized LOC116406890 (LOC116406890), transcript variant X3, mRNA	123362173-123365086, 123365638-123365802	BEL-10_XT-I	
OldhAT1	XM_004919553.4_cds_1_0_chr6_6446974_r	6	6446973-6450126	Xenopus tropicalis ZBED6 C-terminal like (zbed6cl), mRNA	6446974-6448769, 6449084-6449229	hAT-4_LCh	
BEL-4-I_XT	XM_031903497.1_cds_0_0_chr6_60126639_f	6	60126638-60129632	Xenopus tropicalis uncharacterized LOC116411460 (LOC116411460), mRNA	60127403-60129036, 60129050-60129469	BEL-10_XT-I	
BEL-4-I_XT	XM_031902948.1_cds_0_0_chr5_45814946_f	5	45814945-45818155	Xenopus tropicalis uncharacterized LOC116411075 (LOC116411075), mRNA	45815034-45816476, 45817112-45817431, 45817803-45817875	BEL-12_XT-I	
hAT-N13_XT	XM_031904473.1_cds_0_0_chr1_18003320_f	1	18003319-18004792	Xenopus tropicalis fibroin heavy chain-like (LOC116411690), mRNA	18003321-18004761	Helitron-N4_AmRa	
BEL-4-I_XT	XM_031903613.1_cds_1_0_chr6_151119739_r	6	151119738-151125843	Xenopus tropicalis uncharacterized LOC116411480 (LOC116411480), mRNA	151120018-151120090, 151120462-151120781, 151121425-151122859	BEL-12_XT-I	
Kolobok-1_XT	XM_002939351.5_cds_1_0_chr4_143641621_f	4	143641620-143643918	Xenopus tropicalis uncharacterized LOC100487738 (LOC100487738), mRNA	143642514-143643824	Kolobok-2_XT	
Kolobok-2_XT	XM_012960629.3_cds_2_0_chr4_139436205_r	4	139436204-139438373	Xenopus tropicalis coiled-coil domain containing 51 (ccdc51), transcript variant X2, mRNA	139436439-139437619, 139437864-139438373	Kolobok-2_XT	
Kolobok-2_XT	XM_012960632.3_cds_3_0_chr4_139436205_r	4	139436204-139438373	Xenopus tropicalis coiled-coil domain containing 51 (ccdc51), transcript variant X4, mRNA	139436439-139437619, 139437864-139438373	Kolobok-2_XT	
Kolobok-2_XT	XM_031899861.1_cds_1_0_chr4_139445785_r	4	139445784-139447953	Xenopus tropicalis coiled-coil domain containing 51 (ccdc51), transcript variant X1, mRNA	139446059-139447189, 139447448-139447953	Kolobok-2_XT	
Kolobok-2_XT	XM_012960631.3_cds_2_0_chr4_139445785_r	4	139445784-139447953	Xenopus tropicalis coiled-coil domain containing 51 (ccdc51), transcript variant X5, mRNA	139446059-139447189, 139447448-139447953	Kolobok-2_XT	
Kolobok-2_XT	XM_012960630.3_cds_2_0_chr4_139445785_r	4	139445784-139447953	Xenopus tropicalis coiled-coil domain containing 51 (ccdc51), transcript variant X3, mRNA	139446059-139447189, 139447448-139447953	Kolobok-2_XT	
ERV1-4-I_XT	XM_031893987.1_cds_1_0_chr1_186374829_f	1	186374828-186376040	Xenopus tropicalis uncharacterized LOC116407911 (LOC116407911), transcript variant X1, mRNA	186374940-186376032	ERV1-4-LTR_XT	
Kolobok-1_XT	XM_031899558.1_cds_1_0_chr3_146989328_r	3	146989327-146991580	Xenopus tropicalis uncharacterized LOC100489710 (LOC100489710), mRNA	146989633-146990593, 146991298-146991580	Kolobok-1_XT	
BEL-4-I_XT	XM_031906411.1_cds_1_0_chr7_19665528_f	7	19665527-19671491	Xenopus tropicalis uncharacterized LOC101733728 (LOC101733728), mRNA	19668370-19669238, 19669512-19669812, 19670448-19670767, 19671139-19671211	BEL-12_XT-I	
hAT-10_XT	XM_031893344.1_cds_1_0_chr9_5116818_r	9	5116817-5118084	Xenopus tropicalis repetitive proline-rich cell wall protein 2-like (LOC116407633), mRNA	5116862-5117198, 5117203-5118068	hAT-10_XT	
**Zebrafish/GCA_000002035.4**							0.0123
Helitron-2_DR	XM_021477356.1_cds_1_0_chr6_43353271_f	6	43353270-43361226	Danio rerio uncharacterized LOC101882029 (LOC101882029), mRNA	43355995-43361219, 43354165-43354347, 43353381-43353545	Helitron-3_DR	
Helitron-2_DR	XM_021477671.1_cds_1_0_chr7_6016929_r	7	6016928-6025151	Danio rerio uncharacterized LOC101884770 (LOC101884770), mRNA	6016935-6022159, 6024074-6024256, 6024876,6025040	Helitron-3_DR	
Helitron-1_DR	XM_021475425.1_cds_3_0_chr4_31860679_f	4	31860678-31869702	Danio rerio uncharacterized LOC110439423 (LOC110439423), transcript variant X1, mRNA	31864431-31869631, 31863128-31863434	Helitron-4_DR	
Helitron-1_DR	XM_021475426.1_cds_2_0_chr4_31860679_f	4	31860678-31869702	Danio rerio uncharacterized LOC110439423 (LOC110439423), transcript variant X2, mRNA	31864431-31869631, 31863128-31863434	Helitron-4_DR	
Helitron-1_DR	XM_021479014.1_cds_2_0_chr9_44149090_r	9	44149089-44156601	Danio rerio uncharacterized LOC110440049 (LOC110440049), transcript variant X1, mRNA	44149812-44154310, 44155600-44156247	Helitron-4_DR	
Helitron-1_DR	XM_021479015.1_cds_3_0_chr9_44149090_r	9	44149089-44156601	Danio rerio uncharacterized LOC110440049 (LOC110440049), transcript variant X2, mRNA	44149812-44154310, 44155600-44156247	Helitron-4_DR	
Gypsy158-I_DR	XM_009298353.3_cds_0_0_chrUn_KN150252v1_38179_f	Uncharacterised	38178-41307	Danio rerio uncharacterized protein K02A2.6-like (LOC103909980), mRNA	38229-41088	Gypsy-192_DR-I	
IS4EU-2_DR	XM_021473382.1_cds_1_0_chr20_KZ115614v1_alt_42763_f	20	42762-43796	Danio rerio uncharacterized LOC110437851 (LOC110437851), mRNA	42899-43759	ISL2EU-1_DR	
IS4EU-2_DR	XM_021468951.1_cds_1_0_chr21_2212889_r	21	2212888-2213916	Danio rerio uncharacterized LOC110437787 (LOC110437787), mRNA	2212925-2213779	ISL2EU-1_DR	
hAT-4_DR	XM_005155824.1_cds_0_0_chr11_17895941_f	11	17895940-17897068	Danio rerio zinc finger BED domain-containing protein 1-like (LOC101886897), mRNA	17895949-17896644	hAT-30_DR	
Helitron-1_DR	XM_021479014.1_cds_2_0_chr9_44149090_r	9	44149089-44156601	Danio rerio uncharacterized LOC110440049 (LOC110440049), transcript variant X1, mRNA	44149812-44154310	Helitron-4_DR	
Helitron-1_DR	XM_021479015.1_cds_3_0_chr9_44149090_r	9	44149089-44156601	Danio rerio uncharacterized LOC110440049 (LOC110440049), transcript variant X2, mRNA	44149812-44154310, 44155600-44156247	Helitron-4_DR	
IS4EU-2_DR	XM_009299506.3_cds_5_0_chr4_72375200_f	4	72375334-72375975	Danio rerio si:cabz01071907.1 (si:cabz01071907.1), transcript variant X1, mRNA	72375199-72376179	ISL2EU-1_DR	
IS4EU-2_DR	XM_021475290.1_cds_3_0_chr4_72375200_f	4	72375199-72376179	Danio rerio si:cabz01071907.1 (si:cabz01071907.1), transcript variant X3, mRNA	72375334-72375975	ISL2EU-1_DR	
IS4EU-2_DR	XM_021475289.1_cds_3_0_chr4_72375200_f	4	72375199-72376179	Danio rerio si:cabz01071907.1 (si:cabz01071907.1), transcript variant X2, mRNA	72375334-72375975	ISL2EU-1_DR	
Gypsy95-I_DR	XM_005173163.1_cds_0_0_chr14_21020570_r	14	21020569-21023734	Danio rerio uncharacterized protein K02A2.6-like (LOC101884733), mRNA	21022614-21023167, 21022028-21022217, 21021253-21021439	Gypsy-207_DR-I	
HATN16_DR	XM_009301735.3_cds_0_0_chr5_42776614_r	5	42776613-42778491	Danio rerio general transcription factor II-I repeat domain-containing protein 2-like (LOC103911107), mRNA	42777969-42778441	hAT-22_DR	
Gypsy158-I_DR	XM_017354380.1_cds_0_0_chrUn_KN150252v1_36860_f	Uncharacterised	36859-37786	Danio rerio uncharacterized LOC108182213 (LOC108182213), partial mRNA	36912-37375	Gypsy-192_DR-I	
MSAT-3_DR	XM_021466788.1_cds_1_0_chr16_55084565_r	16	55084564-55086621	Danio rerio ribosome-binding protein 1-like (LOC110437958), mRNA	36912-37375, 55086294-55086594, 55084659-55084824	MSAT-3_DR	
IS4EU-2_DR	XM_021473383.1_cds_3_0_chr20_KZ115614v1_alt_44110_r	20	44109-45050	Danio rerio uncharacterized LOC110439056 (LOC110439056), mRNA	44236-44674	ISL2EU-1_DR	
IS4EU-2_DR	XM_002665730.6_cds_2_0_chr20_26894821_f	20	26894820-26895573	Danio rerio uncharacterized LOC100332023 (LOC100332023), mRNA	26894917-26895296	ISL2EU-2_DR	
**Chicken/GCF_000002315.5**							0.0035
GGERV28_I-int	XM_025144565.1_cds_0_0_chrW_1607037_f	W	1607036-1610930	Gallus gallus uncharacterized LOC112530482 (LOC112530482), mRNA	1607130-1607822, 1608104-1608480, 1608792-1610786	ERV2-10_GG-I	
GGLTR11-int	NM_001165913.1_cds_1_0_chr4_85934195_f	4	85934194-85936816	Gallus gallus uncharacterized LOC422926 (LOC422926), mRNA	85934448-85934643, 85935603-85935761, 85935829-85936719	ERV3-2_UCy-I	

Table S8: Conserved domains of GTF2IRD2/2A compared to TEs

#Batch CD-search tool	NIH/NLM/NCBI									
**Query**	**Hit type**	**PSSM-ID**	**From**	**To**	**E-Value**	**Bitscore**	**Accession**	**Short name**	**Incomplete**	**Superfamily**
Q#1 - >Haliaeetus_leucocephalus	specific	427072	565	639	4.14E-35	127.735	pfam02946	GTF2I	-	cl08383
Q#1 - >Haliaeetus_leucocephalus	specific	427072	125	199	4.27E-34	125.039	pfam02946	GTF2I	-	cl08383
Q#1 - >Haliaeetus_leucocephalus	specific	427072	803	875	2.02E-32	120.031	pfam02946	GTF2I	-	cl08383
Q#1 - >Haliaeetus_leucocephalus	specific	427072	348	422	9.58E-29	109.631	pfam02946	GTF2I	-	cl08383
Q#1 - >Haliaeetus_leucocephalus	specific	427072	706	780	1.46E-28	109.246	pfam02946	GTF2I	-	cl08383
Q#2 - >Pterocles_gutturalis	specific	427072	545	619	4.68E-35	127.735	pfam02946	GTF2I	-	cl08383
Q#2 - >Pterocles_gutturalis	specific	427072	108	182	4.86E-34	124.654	pfam02946	GTF2I	-	cl08383
Q#2 - >Pterocles_gutturalis	specific	427072	789	861	2.15E-32	120.031	pfam02946	GTF2I	-	cl08383
Q#2 - >Pterocles_gutturalis	specific	427072	323	397	1.04E-28	109.631	pfam02946	GTF2I	-	cl08383
Q#2 - >Pterocles_gutturalis	specific	427072	676	750	1.53E-28	109.246	pfam02946	GTF2I	-	cl08383
Q#3 - >Aptenodytes_forsteri	specific	427072	573	647	2.38E-35	128.506	pfam02946	GTF2I	-	cl08383
Q#3 - >Aptenodytes_forsteri	specific	427072	135	209	4.94E-33	121.957	pfam02946	GTF2I	-	cl08383
Q#3 - >Aptenodytes_forsteri	specific	427072	795	867	8.27E-32	118.491	pfam02946	GTF2I	-	cl08383
Q#3 - >Aptenodytes_forsteri	specific	427072	356	430	9.23E-29	109.631	pfam02946	GTF2I	-	cl08383
Q#3 - >Aptenodytes_forsteri	specific	427072	698	772	1.40E-28	109.246	pfam02946	GTF2I	-	cl08383
Q#4 - >Antrostomus_carolinensis	specific	427072	578	652	2.00E-35	128.506	pfam02946	GTF2I	-	cl08383
Q#4 - >Antrostomus_carolinensis	specific	427072	125	199	1.83E-34	125.809	pfam02946	GTF2I	-	cl08383
Q#4 - >Antrostomus_carolinensis	specific	427072	356	430	7.63E-29	110.016	pfam02946	GTF2I	-	cl08383
Q#4 - >Antrostomus_carolinensis	specific	427072	709	783	1.18E-28	109.246	pfam02946	GTF2I	-	cl08383
Q#5 - >Phasianus_colchicus	specific	427072	570	644	1.87E-35	128.891	pfam02946	GTF2I	-	cl08383
Q#5 - >Phasianus_colchicus	specific	427072	125	199	9.86E-34	123.883	pfam02946	GTF2I	-	cl08383
Q#5 - >Phasianus_colchicus	specific	427072	796	868	6.49E-32	118.876	pfam02946	GTF2I	-	cl08383
Q#5 - >Phasianus_colchicus	specific	427072	348	422	6.83E-29	110.016	pfam02946	GTF2I	-	cl08383
Q#5 - >Phasianus_colchicus	specific	427072	699	773	1.06E-28	109.631	pfam02946	GTF2I	-	cl08383
Q#6 - >Camarhynchus_parvulus	specific	427072	544	618	1.48E-35	129.276	pfam02946	GTF2I	-	cl08383
Q#6 - >Camarhynchus_parvulus	specific	427072	125	199	1.53E-34	126.195	pfam02946	GTF2I	-	cl08383
Q#6 - >Camarhynchus_parvulus	specific	427072	788	860	1.33E-32	120.802	pfam02946	GTF2I	-	cl08383
Q#6 - >Camarhynchus_parvulus	specific	427072	349	423	8.39E-29	110.016	pfam02946	GTF2I	-	cl08383
Q#6 - >Camarhynchus_parvulus	specific	427072	675	749	1.30E-28	109.246	pfam02946	GTF2I	-	cl08383
Q#7 - >Calypte_anna	specific	427072	571	645	1.82E-35	128.891	pfam02946	GTF2I	-	cl08383
Q#7 - >Calypte_anna	specific	427072	125	199	1.35E-34	126.195	pfam02946	GTF2I	-	cl08383
Q#7 - >Calypte_anna	specific	427072	799	871	1.42E-32	120.417	pfam02946	GTF2I	-	cl08383
Q#7 - >Calypte_anna	specific	427072	349	423	6.66E-29	110.016	pfam02946	GTF2I	-	cl08383
Q#7 - >Calypte_anna	specific	427072	702	776	1.03E-28	109.631	pfam02946	GTF2I	-	cl08383
Q#8 - >Mus_musculus	specific	427072	104	178	6.36E-38	135.825	pfam02946	GTF2I	-	cl08383
Q#8 - >Mus_musculus	superfamily	427072	328	402	1.28E-19	83.8227	cl08383	GTF2I superfamily	-	-
Q#8 - >Mus_musculus	superfamily	405048	489	624	0.00000381518	49.151	cl16778	DUF4371 superfamily	N	-
Q#8 - >Mus_musculus	superfamily	436652	425	484	0.000241678	39.9418	cl39976	zf-C2H2_12 superfamily	-	-
Q#9 - >Camelus_dromedarius	superfamily	405048	95	172	0.00000103996	49.9214	cl16778	DUF4371 superfamily	N	-
Q#10 - >Bos_taurus	specific	427072	109	183	1.12E-40	143.914	pfam02946	GTF2I	-	cl08383
Q#10 - >Bos_taurus	superfamily	427072	333	407	2.16E-19	83.0523	cl08383	GTF2I superfamily	-	-
Q#10 - >Bos_taurus	superfamily	405048	548	625	0.00000619285	48.3806	cl16778	DUF4371 superfamily	N	-
Q#10 - >Bos_taurus	superfamily	436652	430	492	0.000100329	41.0974	cl39976	zf-C2H2_12 superfamily	-	-
Q#11 - >Mus_caroli	specific	427072	104	178	1.37E-38	137.751	pfam02946	GTF2I	-	cl08383
Q#11 - >Mus_caroli	superfamily	427072	324	385	1.14E-12	63.7923	cl08383	GTF2I superfamily	-	-
Q#11 - >Mus_caroli	superfamily	405048	472	607	0.000000742071	51.077	cl16778	DUF4371 superfamily	N	-
Q#11 - >Mus_caroli	superfamily	436652	408	467	0.00117836	38.0158	cl39976	zf-C2H2_12 superfamily	-	-
Q#12 - >Monodon_monoceros	specific	427072	107	181	1.13E-41	146.61	pfam02946	GTF2I	-	cl08383
Q#12 - >Monodon_monoceros	superfamily	427072	331	405	2.60E-19	82.6671	cl08383	GTF2I superfamily	-	-
Q#12 - >Monodon_monoceros	superfamily	405048	482	623	0.00000420518	49.151	cl16778	DUF4371 superfamily	N	-
Q#12 - >Monodon_monoceros	superfamily	436652	428	490	0.000116011	40.7122	cl39976	zf-C2H2_12 superfamily	-	-
Q#13 - >Rhinolophus_ferrumequinum	specific	427072	107	181	5.20E-42	147.381	pfam02946	GTF2I	-	cl08383
Q#13 - >Rhinolophus_ferrumequinum	superfamily	427072	331	405	1.86E-19	83.0523	cl08383	GTF2I superfamily	-	-
Q#13 - >Rhinolophus_ferrumequinum	superfamily	405048	492	623	0.000000813283	51.077	cl16778	DUF4371 superfamily	N	-
Q#13 - >Rhinolophus_ferrumequinum	superfamily	436652	428	487	0.000492084	39.1714	cl39976	zf-C2H2_12 superfamily	-	-
Q#14 - >Leptonychotes_weddellii	superfamily	427072	61	135	1.39E-19	83.0523	cl08383	GTF2I superfamily	-	-
Q#14 - >Leptonychotes_weddellii	superfamily	405048	212	353	0.000000769223	50.6918	cl16778	DUF4371 superfamily	N	-
Q#14 - >Leptonychotes_weddellii	superfamily	436652	158	220	0.000252388	39.5566	cl39976	zf-C2H2_12 superfamily	-	-
Q#15 - >Protobothrops_mucrosquamatu	superfamily	405048	82	268	0.0000274819	46.0694	cl16778	DUF4371 superfamily	-	-
Q#15 - >Protobothrops_mucrosquamatu	superfamily	436652	31	87	0.000495407	38.7862	cl39976	zf-C2H2_12 superfamily	-	-
Q#16 - >Balaenoptera_acutorostrata_sc	specific	427072	107	181	6.32E-41	144.299	pfam02946	GTF2I	-	cl08383
Q#16 - >Balaenoptera_acutorostrata_sc	superfamily	427072	331	405	2.50E-19	83.0523	cl08383	GTF2I superfamily	-	-
Q#16 - >Balaenoptera_acutorostrata_sc	superfamily	405048	482	623	0.00000373295	49.151	cl16778	DUF4371 superfamily	N	-
Q#16 - >Balaenoptera_acutorostrata_sc	superfamily	436652	428	490	0.000125499	40.7122	cl39976	zf-C2H2_12 superfamily	-	-
Q#17 - >Neophocaena_asiaeorientalis_	specific	427072	107	181	1.02E-41	146.61	pfam02946	GTF2I	-	cl08383
Q#17 - >Neophocaena_asiaeorientalis_	superfamily	427072	331	405	2.57E-19	82.6671	cl08383	GTF2I superfamily	-	-
Q#17 - >Neophocaena_asiaeorientalis_	superfamily	405048	490	623	0.00000623448	48.3806	cl16778	DUF4371 superfamily	N	-
Q#17 - >Neophocaena_asiaeorientalis_	superfamily	436652	428	490	0.000037859	42.253	cl39976	zf-C2H2_12 superfamily	-	-
Q#18 - >Neomonachus_schauinslandi	specific	427072	106	180	2.60E-42	148.536	pfam02946	GTF2I	-	cl08383
Q#18 - >Neomonachus_schauinslandi	superfamily	427072	330	404	3.00E-19	82.6671	cl08383	GTF2I superfamily	-	-
Q#18 - >Neomonachus_schauinslandi	superfamily	405048	481	622	0.000000374952	52.2326	cl16778	DUF4371 superfamily	N	-
Q#18 - >Neomonachus_schauinslandi	superfamily	436652	427	489	0.000232845	39.9418	cl39976	zf-C2H2_12 superfamily	-	-
Q#19 - >Meriones_unguiculatus	specific	427072	106	180	9.57E-38	135.439	pfam02946	GTF2I	-	cl08383
Q#19 - >Meriones_unguiculatus	superfamily	427072	329	403	1.28E-19	83.8227	cl08383	GTF2I superfamily	-	-
Q#19 - >Meriones_unguiculatus	superfamily	405048	542	625	0.000408904	42.9878	cl16778	DUF4371 superfamily	N	-
Q#19 - >Meriones_unguiculatus	superfamily	436652	426	485	0.00116569	38.0158	cl39976	zf-C2H2_12 superfamily	-	-
Q#21 - >Bos_mutus	specific	427072	109	183	1.17E-40	143.529	pfam02946	GTF2I	-	cl08383
Q#21 - >Bos_mutus	superfamily	427072	333	407	2.25E-19	83.0523	cl08383	GTF2I superfamily	-	-
Q#21 - >Bos_mutus	superfamily	405048	548	625	0.00000613642	48.3806	cl16778	DUF4371 superfamily	N	-
Q#21 - >Bos_mutus	superfamily	436652	430	492	0.0000974151	41.0974	cl39976	zf-C2H2_12 superfamily	-	-
Q#22 - >Pteropus_alecto	specific	427072	112	186	4.94E-39	138.906	pfam02946	GTF2I	-	cl08383
Q#22 - >Pteropus_alecto	superfamily	427072	336	410	3.56E-19	82.2819	cl08383	GTF2I superfamily	-	-
Q#22 - >Pteropus_alecto	superfamily	405048	487	628	0.000002801	49.5362	cl16778	DUF4371 superfamily	N	-
Q#22 - >Pteropus_alecto	superfamily	436652	433	495	0.0000501098	41.8678	cl39976	zf-C2H2_12 superfamily	-	-
Q#23 - >Ursus_maritimus	specific	427072	107	181	1.24E-41	146.61	pfam02946	GTF2I	-	cl08383
Q#23 - >Ursus_maritimus	superfamily	427072	331	405	2.81E-19	82.6671	cl08383	GTF2I superfamily	-	-
Q#23 - >Ursus_maritimus	superfamily	405048	482	623	0.000000117466	53.7734	cl16778	DUF4371 superfamily	N	-
Q#23 - >Ursus_maritimus	superfamily	436652	428	490	0.000226323	39.9418	cl39976	zf-C2H2_12 superfamily	-	-
Q#24 - >Manis_pentadactyla	specific	427072	107	181	1.49E-41	146.225	pfam02946	GTF2I	-	cl08383
Q#24 - >Manis_pentadactyla	superfamily	427072	328	402	2.80E-19	82.6671	cl08383	GTF2I superfamily	-	-
Q#24 - >Manis_pentadactyla	superfamily	405048	479	626	0.00000863483	47.9954	cl16778	DUF4371 superfamily	N	-
Q#24 - >Manis_pentadactyla	superfamily	436652	425	487	0.000153765	40.7122	cl39976	zf-C2H2_12 superfamily	-	-
Q#25 - >Varanus_komodoensis	superfamily	405048	43	131	0.0000000800711	53.003	cl16778	DUF4371 superfamily	N	-
Q#26 - >Cricetulus_griseus	specific	427072	100	174	9.96E-37	132.358	pfam02946	GTF2I	-	cl08383
Q#26 - >Cricetulus_griseus	superfamily	427072	322	396	1.65E-18	80.3559	cl08383	GTF2I superfamily	-	-
Q#26 - >Cricetulus_griseus	superfamily	405048	535	618	0.00000731205	48.3806	cl16778	DUF4371 superfamily	N	-

Q#26 - >Cricetulus_griseus	superfamily	436652	419	478	0.000110339	41.0974	cl39976	zf-C2H2_12 superfamily	-	-
Q#27 - >Ailuropoda_melanoleuca	specific	427072	138	212	7.09E-42	147.381	pfam02946	GTF2I	-	cl08383
Q#27 - >Ailuropoda_melanoleuca	superfamily	427072	362	436	2.87E-19	82.6671	cl08383	GTF2I superfamily	-	-
Q#27 - >Ailuropoda_melanoleuca	superfamily	405048	513	654	0.0000000493225	54.929	cl16778	DUF4371 superfamily	N	-
Q#27 - >Ailuropoda_melanoleuca	superfamily	436652	459	521	0.000293202	39.9418	cl39976	zf-C2H2_12 superfamily	-	-
Q#28 - >Rattus_rattus	specific	427072	105	179	2.63E-38	136.98	pfam02946	GTF2I	-	cl08383
Q#28 - >Rattus_rattus	superfamily	427072	329	403	1.28E-19	83.8227	cl08383	GTF2I superfamily	-	-
Q#28 - >Rattus_rattus	superfamily	405048	490	625	0.000000302373	52.6178	cl16778	DUF4371 superfamily	N	-
Q#28 - >Rattus_rattus	superfamily	436652	426	485	0.000128985	40.7122	cl39976	zf-C2H2_12 superfamily	-	-
Q#28 - >Rattus_rattus	superfamily	425860	260	336	0.00190389	41.0782	cl38012	ERM superfamily	NC	-
Q#29 - >Larimichthys_crocea	superfamily	405048	10	93	6.12E-10	59.1662	cl16778	DUF4371 superfamily	N	-
Q#30 - >Collichthys_lucidus	superfamily	405048	134	238	0.000000426624	51.077	cl16778	DUF4371 superfamily	N	-
Q#30 - >Collichthys_lucidus	superfamily	399013	500	559	0.0000012153	46.4857	cl05324	Dimer_Tnp_hAT superfamily	N	-
Q#30 - >Collichthys_lucidus	superfamily	436652	14	51	0.000960566	37.6306	cl39976	zf-C2H2_12 superfamily	N	-
Q#31 - >Ursus_arctos_horribilis	specific	427072	107	181	5.73E-42	147.381	pfam02946	GTF2I	-	cl08383
Q#31 - >Ursus_arctos_horribilis	superfamily	427072	331	405	3.03E-19	82.6671	cl08383	GTF2I superfamily	-	-
Q#31 - >Ursus_arctos_horribilis	superfamily	405048	482	623	0.000000245715	52.6178	cl16778	DUF4371 superfamily	N	-
Q#31 - >Ursus_arctos_horribilis	superfamily	436652	428	490	0.000240072	39.9418	cl39976	zf-C2H2_12 superfamily	-	-
Q#32 - >Oreochromis_niloticus	superfamily	405048	184	264	0.000234817	42.2174	cl16778	DUF4371 superfamily	N	-
Q#33 - >Oryzias_latipes	superfamily	405048	63	264	0.00000000487782	57.2402	cl16778	DUF4371 superfamily	-	-
Q#33 - >Oryzias_latipes	superfamily	436652	33	57	0.00826629	38.0158	cl39976	zf-C2H2_12 superfamily	NC	-
Q#34 - >Anolis_carolinensis	superfamily	436652	16	75	0.0000121725	43.0234	cl39976	zf-C2H2_12 superfamily	-	-
Q#34 - >Anolis_carolinensis	superfamily	405048	175	258	0.0000255867	46.0694	cl16778	DUF4371 superfamily	N	-
Q#35 - >Odobenus_rosmarus_divergen	superfamily	405048	73	214	0.000000651846	50.6918	cl16778	DUF4371 superfamily	N	-
Q#35 - >Odobenus_rosmarus_divergen	superfamily	436652	19	81	0.000363762	38.7862	cl39976	zf-C2H2_12 superfamily	-	-
Q#36 - >Homo_sapiens	specific	427072	107	181	1.14E-41	146.61	pfam02946	GTF2I	-	cl08383
Q#36 - >Homo_sapiens	superfamily	427072	332	406	2.99E-20	85.3635	cl08383	GTF2I superfamily	-	-
Q#36 - >Homo_sapiens	superfamily	405048	547	630	0.0000111072	47.6102	cl16778	DUF4371 superfamily	N	-
Q#36 - >Homo_sapiens	superfamily	436652	429	491	0.00100014	38.401	cl39976	zf-C2H2_12 superfamily	-	-
Q#37 - >Microtus_ochrogaster	specific	427072	100	174	7.83E-35	126.965	pfam02946	GTF2I	-	cl08383
Q#37 - >Microtus_ochrogaster	superfamily	427072	324	398	2.17E-18	80.3559	cl08383	GTF2I superfamily	-	-
Q#37 - >Microtus_ochrogaster	superfamily	405048	537	620	0.0000365742	46.0694	cl16778	DUF4371 superfamily	N	-
Q#37 - >Microtus_ochrogaster	superfamily	436652	421	480	0.0000963706	41.0974	cl39976	zf-C2H2_12 superfamily	-	-
Q#38 - >Vicugna_pacos	specific	427072	107	181	3.94E-40	141.988	pfam02946	GTF2I	-	cl08383
Q#38 - >Vicugna_pacos	superfamily	427072	331	405	2.92E-19	82.6671	cl08383	GTF2I superfamily	-	-
Q#38 - >Vicugna_pacos	superfamily	405048	547	624	0.000000806833	51.077	cl16778	DUF4371 superfamily	N	-
Q#38 - >Vicugna_pacos	superfamily	436652	428	487	0.0000323893	42.6382	cl39976	zf-C2H2_12 superfamily	-	-
Q#39 - >Globicephala_melas	specific	427072	107	181	1.20E-41	146.61	pfam02946	GTF2I	-	cl08383
Q#39 - >Globicephala_melas	superfamily	427072	331	405	2.65E-19	82.6671	cl08383	GTF2I superfamily	-	-
Q#39 - >Globicephala_melas	superfamily	405048	482	623	0.00000390797	49.151	cl16778	DUF4371 superfamily	N	-
Q#39 - >Globicephala_melas	superfamily	436652	428	490	0.000116011	40.7122	cl39976	zf-C2H2_12 superfamily	-	-
Q#40 - >Delphinapterus_leucas	specific	427072	107	181	9.82E-42	146.61	pfam02946	GTF2I	-	cl08383
Q#40 - >Delphinapterus_leucas	superfamily	427072	305	379	2.33E-19	83.0523	cl08383	GTF2I superfamily	-	-
Q#40 - >Delphinapterus_leucas	superfamily	405048	456	597	0.00000399684	49.151	cl16778	DUF4371 superfamily	N	-
Q#40 - >Delphinapterus_leucas	superfamily	436652	402	464	0.000117287	40.7122	cl39976	zf-C2H2_12 superfamily	-	-
Q#41 - >Suricata_suricatta	specific	427072	107	181	9.88E-42	146.61	pfam02946	GTF2I	-	cl08383
Q#41 - >Suricata_suricatta	superfamily	427072	331	405	1.94E-19	83.0523	cl08383	GTF2I superfamily	-	-
Q#41 - >Suricata_suricatta	superfamily	405048	546	623	0.0000108927	47.6102	cl16778	DUF4371 superfamily	N	-
Q#41 - >Suricata_suricatta	superfamily	436652	428	490	0.000197227	40.327	cl39976	zf-C2H2_12 superfamily	-	-
Q#42 - >Nannospalax_galili	specific	427072	106	180	1.51E-38	137.751	pfam02946	GTF2I	-	cl08383
Q#42 - >Nannospalax_galili	superfamily	427072	342	416	1.30E-20	86.5191	cl08383	GTF2I superfamily	-	-
Q#42 - >Nannospalax_galili	superfamily	405048	557	640	0.00000594479	48.3806	cl16778	DUF4371 superfamily	N	-
Q#42 - >Nannospalax_galili	superfamily	436652	439	498	0.000172983	40.327	cl39976	zf-C2H2_12 superfamily	-	-
Q#43 - >Physeter_catodon	specific	427072	107	181	1.36E-41	146.225	pfam02946	GTF2I	-	cl08383
Q#43 - >Physeter_catodon	superfamily	427072	331	405	2.57E-19	82.6671	cl08383	GTF2I superfamily	-	-
Q#43 - >Physeter_catodon	superfamily	405048	482	629	0.000011015	50.6918	cl16778	DUF4371 superfamily	N	-
Q#43 - >Physeter_catodon	superfamily	436652	428	490	0.000559191	38.7862	cl39976	zf-C2H2_12 superfamily	-	-
Q#44 - >Bos_indicus	specific	427072	109	183	7.12E-41	144.299	pfam02946	GTF2I	-	cl08383
Q#44 - >Bos_indicus	superfamily	427072	333	407	7.78E-20	84.2079	cl08383	GTF2I superfamily	-	-
Q#44 - >Bos_indicus	superfamily	405048	548	625	0.00000613642	48.3806	cl16778	DUF4371 superfamily	N	-
Q#44 - >Bos_indicus	superfamily	436652	430	492	0.00010333	41.0974	cl39976	zf-C2H2_12 superfamily	-	-
Q#45 - >Bison_bison_bison	specific	427072	109	183	1.07E-40	143.914	pfam02946	GTF2I	-	cl08383
Q#45 - >Bison_bison_bison	superfamily	427072	333	407	2.12E-19	83.0523	cl08383	GTF2I superfamily	-	-
Q#45 - >Bison_bison_bison	superfamily	405048	548	625	0.00000591579	48.3806	cl16778	DUF4371 superfamily	N	-
Q#45 - >Bison_bison_bison	superfamily	436652	430	492	0.00010232	41.0974	cl39976	zf-C2H2_12 superfamily	-	-
Q#47 - >Arvicola_amphibius	specific	427072	100	174	1.30E-36	131.973	pfam02946	GTF2I	-	cl08383
Q#47 - >Arvicola_amphibius	superfamily	427072	336	410	4.44E-18	79.2003	cl08383	GTF2I superfamily	-	-
Q#47 - >Arvicola_amphibius	superfamily	405048	549	632	0.0000897746	44.9138	cl16778	DUF4371 superfamily	N	-
Q#47 - >Arvicola_amphibius	superfamily	436652	433	492	0.000116527	40.7122	cl39976	zf-C2H2_12 superfamily	-	-
Q#48 - >Lynx_canadensis	specific	427072	107	181	1.62E-42	148.921	pfam02946	GTF2I	-	cl08383
Q#48 - >Lynx_canadensis	superfamily	427072	331	405	2.35E-19	83.0523	cl08383	GTF2I superfamily	-	-
Q#48 - >Lynx_canadensis	superfamily	405048	546	623	0.000146216	44.1434	cl16778	DUF4371 superfamily	N	-
Q#48 - >Lynx_canadensis	superfamily	436652	428	490	0.000335351	39.5566	cl39976	zf-C2H2_12 superfamily	-	-
Q#49 - >Zalophus_californianus	specific	427072	106	180	1.93E-41	145.84	pfam02946	GTF2I	-	cl08383
Q#49 - >Zalophus_californianus	superfamily	427072	330	404	3.09E-19	82.6671	cl08383	GTF2I superfamily	-	-
Q#49 - >Zalophus_californianus	superfamily	405048	481	622	0.000000177715	53.003	cl16778	DUF4371 superfamily	N	-
Q#49 - >Zalophus_californianus	superfamily	436652	427	489	0.000235145	39.9418	cl39976	zf-C2H2_12 superfamily	-	-
Q#50 - >Canis_lupus_dingo	specific	427072	107	181	9.32E-42	146.995	pfam02946	GTF2I	-	cl08383
Q#50 - >Canis_lupus_dingo	superfamily	427072	331	405	2.81E-19	82.6671	cl08383	GTF2I superfamily	-	-
Q#50 - >Canis_lupus_dingo	superfamily	405048	482	623	0.00000409117	49.151	cl16778	DUF4371 superfamily	N	-
Q#50 - >Canis_lupus_dingo	superfamily	436652	428	490	0.000283738	39.9418	cl39976	zf-C2H2_12 superfamily	-	-
Q#51 - >Orcinus_orca	specific	427072	107	181	1.14E-41	146.61	pfam02946	GTF2I	-	cl08383
Q#51 - >Orcinus_orca	superfamily	427072	331	405	2.50E-19	83.0523	cl08383	GTF2I superfamily	-	-
Q#51 - >Orcinus_orca	superfamily	405048	482	623	0.00000424389	49.151	cl16778	DUF4371 superfamily	N	-
Q#51 - >Orcinus_orca	superfamily	436652	428	490	0.000116011	40.7122	cl39976	zf-C2H2_12 superfamily	-	-
Q#52 - >Eumetopias_jubatus	specific	427072	106	180	1.93E-41	145.84	pfam02946	GTF2I	-	cl08383
Q#52 - >Eumetopias_jubatus	superfamily	427072	330	404	3.09E-19	82.6671	cl08383	GTF2I superfamily	-	-
Q#52 - >Eumetopias_jubatus	superfamily	405048	481	622	0.000000177715	53.003	cl16778	DUF4371 superfamily	N	-
Q#52 - >Eumetopias_jubatus	superfamily	436652	427	489	0.000235145	39.9418	cl39976	zf-C2H2_12 superfamily	-	-
Q#53 - >Ovis_aries	specific	427072	109	183	1.12E-40	143.914	pfam02946	GTF2I	-	cl08383
Q#53 - >Ovis_aries	superfamily	427072	333	407	1.66E-19	83.4375	cl08383	GTF2I superfamily	-	-
Q#53 - >Ovis_aries	superfamily	405048	548	625	0.00000534902	48.7658	cl16778	DUF4371 superfamily	N	-
Q#53 - >Ovis_aries	superfamily	436652	430	492	0.0000200415	43.0234	cl39976	zf-C2H2_12 superfamily	-	-
Q#54 - >Lagenorhynchus_obliquidens	specific	427072	107	181	1.11E-41	146.61	pfam02946	GTF2I	-	cl08383
Q#54 - >Lagenorhynchus_obliquidens	superfamily	427072	331	405	2.60E-19	82.6671	cl08383	GTF2I superfamily	-	-
Q#54 - >Lagenorhynchus_obliquidens	superfamily	405048	482	623	0.00000416683	49.151	cl16778	DUF4371 superfamily	N	-
Q#54 - >Lagenorhynchus_obliquidens	superfamily	436652	428	490	0.000116011	40.7122	cl39976	zf-C2H2_12 superfamily	-	-
Q#55 - >Acinonyx_jubatus	specific	427072	59	133	1.54E-42	148.921	pfam02946	GTF2I	-	cl08383
Q#55 - >Acinonyx_jubatus	superfamily	427072	283	357	2.28E-19	83.0523	cl08383	GTF2I superfamily	-	-

Q#55 - >Acinonyx_jubatus	superfamily	405048	498	575	0.000149402	44.1434	cl16778	DUF4371 superfamily	N	-
Q#55 - >Acinonyx_jubatus	superfamily	436652	380	442	0.000263846	39.9418	cl39976	zf-C2H2_12 superfamily	-	-
Q#56 - >Vulpes_vulpes	specific	427072	107	181	8.71E-42	146.995	pfam02946	GTF2I	-	cl08383
Q#56 - >Vulpes_vulpes	superfamily	427072	331	405	2.65E-19	82.6671	cl08383	GTF2I superfamily	-	-
Q#56 - >Vulpes_vulpes	superfamily	405048	482	623	0.00000405386	49.151	cl16778	DUF4371 superfamily	N	-
Q#56 - >Vulpes_vulpes	superfamily	436652	428	490	0.000295118	39.9418	cl39976	zf-C2H2_12 superfamily	-	-
Q#57 - >Callorhinus_ursinus	specific	427072	106	180	1.82E-41	145.84	pfam02946	GTF2I	-	cl08383
Q#57 - >Callorhinus_ursinus	superfamily	427072	330	404	3.03E-19	82.6671	cl08383	GTF2I superfamily	-	-
Q#57 - >Callorhinus_ursinus	superfamily	405048	481	622	0.000000189568	53.003	cl16778	DUF4371 superfamily	N	-
Q#57 - >Callorhinus_ursinus	superfamily	436652	427	489	0.000237468	39.9418	cl39976	zf-C2H2_12 superfamily	-	-
Q#58 - >Bubalus_bubalis	specific	427072	109	183	1.28E-40	143.529	pfam02946	GTF2I	-	cl08383
Q#58 - >Bubalus_bubalis	superfamily	427072	333	407	2.08E-19	83.0523	cl08383	GTF2I superfamily	-	-
Q#58 - >Bubalus_bubalis	superfamily	405048	548	625	0.0000062498	48.3806	cl16778	DUF4371 superfamily	N	-
Q#58 - >Bubalus_bubalis	superfamily	436652	430	492	0.0000909408	41.0974	cl39976	zf-C2H2_12 superfamily	-	-
Q#59 - >Myotis_lucifugus	specific	427072	107	181	9.17E-41	143.914	pfam02946	GTF2I	-	cl08383
Q#59 - >Myotis_lucifugus	superfamily	427072	310	384	5.83E-20	84.5931	cl08383	GTF2I superfamily	-	-
Q#59 - >Myotis_lucifugus	superfamily	405048	525	608	0.0000110074	47.6102	cl16778	DUF4371 superfamily	N	-
Q#59 - >Myotis_lucifugus	superfamily	436652	407	469	0.000621096	38.7862	cl39976	zf-C2H2_12 superfamily	-	-
Q#60 - >Equus_caballus	specific	427072	117	191	4.43E-40	141.988	pfam02946	GTF2I	-	cl08383
Q#60 - >Equus_caballus	superfamily	427072	341	415	2.95E-19	82.6671	cl08383	GTF2I superfamily	-	-
Q#60 - >Equus_caballus	superfamily	405048	556	633	0.0000120816	47.6102	cl16778	DUF4371 superfamily	N	-
Q#60 - >Equus_caballus	superfamily	436652	438	500	0.0000479637	41.8678	cl39976	zf-C2H2_12 superfamily	-	-
Q#61 - >Enhydra_lutris_kenyoni	specific	427072	107	181	2.72E-40	142.758	pfam02946	GTF2I	-	cl08383
Q#61 - >Enhydra_lutris_kenyoni	superfamily	427072	331	405	1.54E-19	83.4375	cl08383	GTF2I superfamily	-	-
Q#61 - >Enhydra_lutris_kenyoni	superfamily	405048	482	624	0.00000344162	49.151	cl16778	DUF4371 superfamily	N	-
Q#61 - >Enhydra_lutris_kenyoni	superfamily	436652	428	490	0.00023798	39.9418	cl39976	zf-C2H2_12 superfamily	-	-
Q#62 - >Sus_scrofa	specific	427072	107	181	1.36E-41	146.225	pfam02946	GTF2I	-	cl08383
Q#62 - >Sus_scrofa	superfamily	427072	331	405	2.81E-19	82.6671	cl08383	GTF2I superfamily	-	-
Q#62 - >Sus_scrofa	superfamily	405048	479	623	0.00000137322	50.3066	cl16778	DUF4371 superfamily	N	-
Q#62 - >Sus_scrofa	superfamily	436652	428	490	0.000120662	40.7122	cl39976	zf-C2H2_12 superfamily	-	-
Q#63 - >Odocoileus_virginianus_texanu	specific	427072	1	54	1.52E-26	103.083	pfam02946	GTF2I	N	cl08383
Q#63 - >Odocoileus_virginianus_texanu	superfamily	427072	204	278	2.62E-19	82.6671	cl08383	GTF2I superfamily	-	-
Q#63 - >Odocoileus_virginianus_texanu	superfamily	405048	419	496	0.00000620479	48.3806	cl16778	DUF4371 superfamily	N	-
Q#63 - >Odocoileus_virginianus_texanu	superfamily	436652	301	363	0.000283891	39.5566	cl39976	zf-C2H2_12 superfamily	-	-
Q#64 - >Panthera_pardus	specific	427072	59	133	1.25E-42	149.307	pfam02946	GTF2I	-	cl08383
Q#64 - >Panthera_pardus	superfamily	427072	283	357	2.05E-19	83.0523	cl08383	GTF2I superfamily	-	-
Q#64 - >Panthera_pardus	superfamily	405048	498	575	0.000138931	44.1434	cl16778	DUF4371 superfamily	N	-
Q#64 - >Panthera_pardus	superfamily	436652	380	442	0.000261265	39.9418	cl39976	zf-C2H2_12 superfamily	-	-
Q#65 - >Equus_asinus	specific	427072	235	309	7.30E-40	141.603	pfam02946	GTF2I	-	cl08383
Q#65 - >Equus_asinus	superfamily	427072	459	533	4.11E-19	82.2819	cl08383	GTF2I superfamily	-	-
Q#65 - >Equus_asinus	superfamily	405048	674	751	0.0000100281	47.9954	cl16778	DUF4371 superfamily	N	-
Q#65 - >Equus_asinus	superfamily	436652	556	618	0.0000443585	42.253	cl39976	zf-C2H2_12 superfamily	-	-
Q#66 - >Ceratotherium_simum_simum	specific	427072	107	181	4.00E-41	145.069	pfam02946	GTF2I	-	cl08383
Q#66 - >Ceratotherium_simum_simum	superfamily	427072	331	405	3.00E-19	82.6671	cl08383	GTF2I superfamily	-	-
Q#66 - >Ceratotherium_simum_simum	superfamily	405048	482	623	0.00000136067	50.3066	cl16778	DUF4371 superfamily	N	-
Q#66 - >Ceratotherium_simum_simum	superfamily	436652	428	490	0.0000731075	41.4826	cl39976	zf-C2H2_12 superfamily	-	-
Q#67 - >Mustela_putorius_furo	specific	427072	59	133	7.50E-41	144.299	pfam02946	GTF2I	-	cl08383
Q#67 - >Mustela_putorius_furo	superfamily	427072	283	357	1.56E-19	83.4375	cl08383	GTF2I superfamily	-	-
Q#67 - >Mustela_putorius_furo	superfamily	405048	434	576	0.0000251225	46.4546	cl16778	DUF4371 superfamily	N	-
Q#67 - >Mustela_putorius_furo	superfamily	436652	380	442	0.000202573	40.327	cl39976	zf-C2H2_12 superfamily	-	-
Q#68 - >Condylura_cristata	specific	427072	107	181	1.21E-39	140.832	pfam02946	GTF2I	-	cl08383
Q#68 - >Condylura_cristata	superfamily	427072	331	405	2.65E-19	82.6671	cl08383	GTF2I superfamily	-	-
Q#68 - >Condylura_cristata	superfamily	405048	492	623	0.00000376732	49.151	cl16778	DUF4371 superfamily	N	-
Q#68 - >Condylura_cristata	superfamily	436652	428	487	0.000254657	39.9418	cl39976	zf-C2H2_12 superfamily	-	-
Q#69 - >Vulpes_lagopus	specific	427072	107	181	8.21E-42	146.995	pfam02946	GTF2I	-	cl08383
Q#69 - >Vulpes_lagopus	superfamily	427072	331	405	2.60E-19	82.6671	cl08383	GTF2I superfamily	-	-
Q#69 - >Vulpes_lagopus	superfamily	405048	482	623	0.00000394394	49.151	cl16778	DUF4371 superfamily	N	-
Q#69 - >Vulpes_lagopus	superfamily	436652	428	490	0.000286541	39.9418	cl39976	zf-C2H2_12 superfamily	-	-
Q#70 - >Mesocricetus_auratus	specific	427072	100	174	8.45E-37	132.743	pfam02946	GTF2I	-	cl08383
Q#70 - >Mesocricetus_auratus	superfamily	427072	322	396	1.65E-18	80.3559	cl08383	GTF2I superfamily	-	-
Q#70 - >Mesocricetus_auratus	superfamily	405048	535	618	0.00000426025	49.151	cl16778	DUF4371 superfamily	N	-
Q#70 - >Mesocricetus_auratus	superfamily	436652	419	478	0.000112529	41.0974	cl39976	zf-C2H2_12 superfamily	-	-
Q#71 - >Puma_yagouaroundi	specific	427072	107	181	1.28E-42	149.307	pfam02946	GTF2I	-	cl08383
Q#71 - >Puma_yagouaroundi	superfamily	427072	331	405	2.07E-19	83.0523	cl08383	GTF2I superfamily	-	-
Q#71 - >Puma_yagouaroundi	superfamily	405048	546	623	0.000151625	44.1434	cl16778	DUF4371 superfamily	N	-
Q#71 - >Puma_yagouaroundi	superfamily	436652	428	490	0.000342011	39.5566	cl39976	zf-C2H2_12 superfamily	-	-
Q#72 - >Oryx_dammah	specific	427072	109	183	1.43E-40	143.529	pfam02946	GTF2I	-	cl08383
Q#72 - >Oryx_dammah	superfamily	427072	333	407	2.12E-19	83.0523	cl08383	GTF2I superfamily	-	-
Q#72 - >Oryx_dammah	superfamily	405048	548	625	0.00000575553	48.7658	cl16778	DUF4371 superfamily	N	-
Q#72 - >Oryx_dammah	superfamily	436652	430	492	0.00001799	43.0234	cl39976	zf-C2H2_12 superfamily	-	-
Q#73 - >Canis_lupus_familiaris	specific	427072	107	181	9.32E-42	146.995	pfam02946	GTF2I	-	cl08383
Q#73 - >Canis_lupus_familiaris	superfamily	427072	331	405	2.81E-19	82.6671	cl08383	GTF2I superfamily	-	-
Q#73 - >Canis_lupus_familiaris	superfamily	405048	482	623	0.00000409117	49.151	cl16778	DUF4371 superfamily	N	-
Q#73 - >Canis_lupus_familiaris	superfamily	436652	428	490	0.000283738	39.9418	cl39976	zf-C2H2_12 superfamily	-	-
Q#74 - >Peromyscus_leucopus	specific	427072	105	179	1.94E-38	137.365	pfam02946	GTF2I	-	cl08383
Q#74 - >Peromyscus_leucopus	superfamily	427072	341	415	1.05E-18	81.1263	cl08383	GTF2I superfamily	-	-
Q#74 - >Peromyscus_leucopus	superfamily	405048	554	631	0.0000108068	47.6102	cl16778	DUF4371 superfamily	N	-
Q#74 - >Peromyscus_leucopus	superfamily	436652	438	495	0.000195508	40.327	cl39976	zf-C2H2_12 superfamily	-	-
Q#75 - >Onychomys_torridus	specific	427072	131	205	1.21E-37	135.054	pfam02946	GTF2I	-	cl08383
Q#75 - >Onychomys_torridus	superfamily	427072	355	429	2.60E-18	79.9707	cl08383	GTF2I superfamily	-	-
Q#75 - >Onychomys_torridus	superfamily	405048	516	651	0.00000172751	50.3066	cl16778	DUF4371 superfamily	N	-
Q#75 - >Onychomys_torridus	superfamily	436652	452	511	0.00266304	37.2454	cl39976	zf-C2H2_12 superfamily	-	-
Q#76 - >Halichoerus_grypus	specific	427072	106	180	2.10E-42	148.536	pfam02946	GTF2I	-	cl08383
Q#76 - >Halichoerus_grypus	superfamily	427072	330	404	3.21E-19	82.6671	cl08383	GTF2I superfamily	-	-
Q#76 - >Halichoerus_grypus	superfamily	405048	481	622	0.000000165072	53.3882	cl16778	DUF4371 superfamily	N	-
Q#76 - >Halichoerus_grypus	superfamily	436652	427	489	0.000237468	39.9418	cl39976	zf-C2H2_12 superfamily	-	-
Q#77 - >Mirounga_leonina	specific	427072	106	180	2.02E-42	148.536	pfam02946	GTF2I	-	cl08383
Q#77 - >Mirounga_leonina	superfamily	427072	330	404	3.09E-19	82.6671	cl08383	GTF2I superfamily	-	-
Q#77 - >Mirounga_leonina	superfamily	405048	481	622	0.000000403621	52.2326	cl16778	DUF4371 superfamily	N	-
Q#77 - >Mirounga_leonina	superfamily	436652	427	489	0.000200926	40.327	cl39976	zf-C2H2_12 superfamily	-	-
Q#78 - >Tursiops_truncatus	specific	427072	107	181	1.10E-41	146.61	pfam02946	GTF2I	-	cl08383
Q#78 - >Tursiops_truncatus	superfamily	427072	331	405	2.57E-19	82.6671	cl08383	GTF2I superfamily	-	-
Q#78 - >Tursiops_truncatus	superfamily	405048	482	623	0.00000398025	49.151	cl16778	DUF4371 superfamily	N	-
Q#78 - >Tursiops_truncatus	superfamily	436652	428	490	0.000120662	40.7122	cl39976	zf-C2H2_12 superfamily	-	-
Q#79 - >Lontra_canadensis	specific	427072	107	181	6.10E-41	144.299	pfam02946	GTF2I	-	cl08383
Q#79 - >Lontra_canadensis	superfamily	427072	331	405	1.39E-19	83.4375	cl08383	GTF2I superfamily	-	-
Q#79 - >Lontra_canadensis	superfamily	405048	482	625	0.0000226736	46.8398	cl16778	DUF4371 superfamily	N	-
Q#79 - >Lontra_canadensis	superfamily	436652	428	490	0.000250237	39.9418	cl39976	zf-C2H2_12 superfamily	-	-
Q#80 - >Phocoena_sinus	specific	427072	107	181	1.21E-41	146.61	pfam02946	GTF2I	-	cl08383

Q#80 - >Phocoena_sinus	superfamily	427072	331	405	2.65E-19	82.6671	cl08383	GTF2I superfamily	-	-
Q#80 - >Phocoena_sinus	superfamily	405048	482	623	0.00000424389	49.151	cl16778	DUF4371 superfamily	N	-
Q#80 - >Phocoena_sinus	superfamily	436652	428	490	0.000117157	40.7122	cl39976	zf-C2H2_12 superfamily	-	-
Q#81 - >Phoca_vitulina	specific	427072	106	180	2.10E-42	148.536	pfam02946	GTF2I	-	cl08383
Q#81 - >Phoca_vitulina	superfamily	427072	330	404	3.21E-19	82.6671	cl08383	GTF2I superfamily	-	-
Q#81 - >Phoca_vitulina	superfamily	405048	481	622	0.000000165072	53.3882	cl16778	DUF4371 superfamily	N	-
Q#81 - >Phoca_vitulina	superfamily	436652	427	489	0.000237468	39.9418	cl39976	zf-C2H2_12 superfamily	-	-
Q#82 - >Mustela_erminea	specific	427072	107	181	6.84E-41	144.299	pfam02946	GTF2I	-	cl08383
Q#82 - >Mustela_erminea	superfamily	427072	331	405	1.51E-19	83.4375	cl08383	GTF2I superfamily	-	-
Q#82 - >Mustela_erminea	superfamily	405048	482	624	0.0000314487	46.4546	cl16778	DUF4371 superfamily	N	-
Q#82 - >Mustela_erminea	superfamily	436652	428	490	0.00023798	39.9418	cl39976	zf-C2H2_12 superfamily	-	-
Q#83 - >Capra_hircus	specific	427072	262	336	4.07E-41	145.069	pfam02946	GTF2I	-	cl08383
Q#83 - >Capra_hircus	superfamily	427072	486	560	1.39E-19	83.8227	cl08383	GTF2I superfamily	-	-
Q#83 - >Capra_hircus	superfamily	405048	647	778	0.00000112237	51.077	cl16778	DUF4371 superfamily	N	-
Q#83 - >Capra_hircus	superfamily	436652	583	645	0.0000209434	43.0234	cl39976	zf-C2H2_12 superfamily	-	-
Q#83 - >Capra_hircus	superfamily	223029	99	148	0.003399	41.1438	cl42984	PHA03264 superfamily	NC	-
Q#84 - >Nicrophorus_vespilloides	superfamily	405048	165	265	0.000676043	41.447	cl16778	DUF4371 superfamily	N	-
Q#85 - >Labeo_rohita	superfamily	405048	157	240	0.0000426093	45.299	cl16778	DUF4371 superfamily	N	-
Q#86 - >Gadus_morhua	superfamily	436652	16	70	0.000000384566	47.2606	cl39976	zf-C2H2_12 superfamily	-	-
Q#86 - >Gadus_morhua	superfamily	405048	118	255	0.00000128339	49.9214	cl16778	DUF4371 superfamily	N	-
Q#87 - >Erpetoichthys_calabaricus	superfamily	405048	131	255	0.00000000113178	59.1662	cl16778	DUF4371 superfamily	N	-
Q#87 - >Erpetoichthys_calabaricus	superfamily	399013	562	609	0.00306618	36.8557	cl05324	Dimer_Tnp_hAT superfamily	N	-
Q#88 - >Thamnophis_sirtalis	specific	426216	628	669	2.37E-20	84.7915	pfam01352	KRAB	-	cl02581
Q#88 - >Thamnophis_sirtalis	specific	227381	774	975	0.00000626933	49.6942	COG5048	COG5048	N	cl34881
Q#88 - >Thamnophis_sirtalis	superfamily	405048	169	252	0.00000977972	47.9954	cl16778	DUF4371 superfamily	N	-
Q#88 - >Thamnophis_sirtalis	superfamily	436652	14	55	0.000258296	39.9418	cl39976	zf-C2H2_12 superfamily	C	-
Q#89 - >Maylandia_zebra	superfamily	436652	13	73	5.55E-10	55.3498	cl39976	zf-C2H2_12 superfamily	-	-
Q#89 - >Maylandia_zebra	superfamily	405048	115	250	0.00000000492899	57.2402	cl16778	DUF4371 superfamily	N	-
Q#90 - >Orycteropus_afer_afer	specific	427072	107	181	3.17E-37	133.899	pfam02946	GTF2I	-	cl08383
Q#90 - >Orycteropus_afer_afer	superfamily	427072	331	405	7.69E-22	89.9859	cl08383	GTF2I superfamily	-	-
Q#90 - >Orycteropus_afer_afer	superfamily	405048	546	623	0.000010598	47.6102	cl16778	DUF4371 superfamily	N	-
Q#90 - >Orycteropus_afer_afer	superfamily	436652	428	490	0.000022945	43.0234	cl39976	zf-C2H2_12 superfamily	-	-
Q#91 - >Lipotes_vexillifer	specific	427072	107	181	1.22E-41	146.61	pfam02946	GTF2I	-	cl08383
Q#91 - >Lipotes_vexillifer	superfamily	427072	331	405	2.47E-19	83.0523	cl08383	GTF2I superfamily	-	-
Q#91 - >Lipotes_vexillifer	superfamily	405048	482	623	0.00000275846	49.5362	cl16778	DUF4371 superfamily	N	-
Q#91 - >Lipotes_vexillifer	superfamily	436652	428	490	0.00011045	41.0974	cl39976	zf-C2H2_12 superfamily	-	-
Q#92 - >Chrysochloris_asiatica	specific	427072	107	181	3.17E-40	142.373	pfam02946	GTF2I	-	cl08383
Q#92 - >Chrysochloris_asiatica	superfamily	427072	331	405	3.42E-20	85.3635	cl08383	GTF2I superfamily	-	-
Q#92 - >Chrysochloris_asiatica	superfamily	405048	545	622	0.0000869922	44.9138	cl16778	DUF4371 superfamily	N	-
Q#92 - >Chrysochloris_asiatica	superfamily	436652	428	490	0.00146478	37.6306	cl39976	zf-C2H2_12 superfamily	-	-
Q#93 - >Elephantulus_edwardii	specific	427072	244	318	3.48E-38	136.595	pfam02946	GTF2I	-	cl08383
Q#93 - >Elephantulus_edwardii	superfamily	427072	468	542	1.85E-21	89.2155	cl08383	GTF2I superfamily	-	-
Q#93 - >Elephantulus_edwardii	superfamily	405048	673	750	0.0000126159	47.6102	cl16778	DUF4371 superfamily	N	-
Q#93 - >Elephantulus_edwardii	superfamily	436652	565	627	0.000154602	40.7122	cl39976	zf-C2H2_12 superfamily	-	-
Q#94 - >Rana_temporaria	superfamily	436652	16	69	0.0000209857	42.253	cl39976	zf-C2H2_12 superfamily	-	-
Q#94 - >Rana_temporaria	superfamily	405048	173	254	0.000130038	43.373	cl16778	DUF4371 superfamily	N	-
Q#95 - >Anguilla_anguilla	superfamily	436652	29	88	0.000000114464	48.8014	cl39976	zf-C2H2_12 superfamily	-	-
Q#95 - >Anguilla_anguilla	superfamily	405048	75	271	0.000000691634	50.6918	cl16778	DUF4371 superfamily	-	-
Q#96 - >Periophthalmus_magnuspinnat	superfamily	405048	60	261	0.0000055875	47.9954	cl16778	DUF4371 superfamily	-	-
Q#96 - >Periophthalmus_magnuspinnat	superfamily	436652	14	56	0.000395208	38.7862	cl39976	zf-C2H2_12 superfamily	C	-
Q#97 - >Geotrypetes_seraphini	superfamily	405048	8	115	0.00000255322	48.3806	cl16778	DUF4371 superfamily	N	-
Q#98 - >Xiphophorus_hellerii	superfamily	405048	31	114	0.0000000672087	53.3882	cl16778	DUF4371 superfamily	N	-
Q#98 - >Xiphophorus_hellerii	superfamily	399013	357	436	0.000000120188	49.1821	cl05324	Dimer_Tnp_hAT superfamily	-	-
Q#99 - >Thamnophis_elegans	specific	426216	628	669	2.09E-20	84.7915	pfam01352	KRAB	-	cl02581
Q#99 - >Thamnophis_elegans	specific	227381	923	980	0.0000094856	49.309	COG5048	COG5048	C	cl34881
Q#99 - >Thamnophis_elegans	superfamily	405048	169	252	0.00000977972	47.9954	cl16778	DUF4371 superfamily	N	-
Q#99 - >Thamnophis_elegans	specific	433230	883	907	0.0000994995	40.0319	pfam13465	zf-H2C2_2	-	cl22375
Q#99 - >Thamnophis_elegans	specific	227381	867	920	0.000222268	44.6866	COG5048	COG5048	C	cl34881
Q#99 - >Thamnophis_elegans	superfamily	436652	14	55	0.000245908	39.9418	cl39976	zf-C2H2_12 superfamily	C	-
Q#101 - >Xiphophorus_couchianus	superfamily	436652	35	61	0.00009701	40.7122	cl39976	zf-C2H2_12 superfamily	NC	-
Q#101 - >Xiphophorus_couchianus	superfamily	405048	177	260	0.000143779	43.7582	cl16778	DUF4371 superfamily	N	-
Q#102 - >Chelmon_rostratus	superfamily	405048	62	256	8.01E-10	59.5514	cl16778	DUF4371 superfamily	-	-
Q#102 - >Chelmon_rostratus	superfamily	436652	12	73	0.0000000053451	52.6534	cl39976	zf-C2H2_12 superfamily	-	-
Q#102 - >Chelmon_rostratus	superfamily	446965	265	395	0.00834019	37.7776	cl06336	Commd superfamily	C	-
Q#103 - >Bufo_bufo	superfamily	405048	33	254	1.78E-11	64.1738	cl16778	DUF4371 superfamily	-	-
Q#104 - >Salvelinus_namaycush	superfamily	405048	33	254	0.00000000309565	57.6254	cl16778	DUF4371 superfamily	-	-
Q#105 - >Kryptolebias_marmoratus	superfamily	405048	61	257	0.00000000113029	59.1662	cl16778	DUF4371 superfamily	-	-
Q#105 - >Kryptolebias_marmoratus	superfamily	436652	15	74	0.00000000126472	54.1942	cl39976	zf-C2H2_12 superfamily	-	-
Q#106 - >Fundulus_heteroclitus	superfamily	405048	254	335	0.00000279848	48.7658	cl16778	DUF4371 superfamily	N	-
Q#106 - >Fundulus_heteroclitus	superfamily	436652	99	151	0.0000160508	42.6382	cl39976	zf-C2H2_12 superfamily	-	-
Q#107 - >Oncorhynchus_kisutch	superfamily	405048	128	211	0.0000000165219	55.3142	cl16778	DUF4371 superfamily	N	-
Q#108 - >Archocentrus_centrarchus	superfamily	405048	95	261	0.000000200689	52.2326	cl16778	DUF4371 superfamily	N	-
Q#108 - >Archocentrus_centrarchus	superfamily	399013	567	621	0.000106556	41.0929	cl05324	Dimer_Tnp_hAT superfamily	N	-
Q#108 - >Archocentrus_centrarchus	superfamily	436652	14	55	0.000155574	39.9418	cl39976	zf-C2H2_12 superfamily	C	-
Q#109 - >Sphaeramia_orbicularis	superfamily	405048	180	263	0.000395708	42.2174	cl16778	DUF4371 superfamily	N	-
Q#109 - >Sphaeramia_orbicularis	superfamily	399013	542	599	0.00735565	35.7001	cl05324	Dimer_Tnp_hAT superfamily	N	-
Q#110 - >Myripristis_murdjan	superfamily	405048	106	191	0.0000801967	44.1434	cl16778	DUF4371 superfamily	N	-
Q#111 - >Takifugu_rubripes	superfamily	405048	64	291	0.00000000350976	57.6254	cl16778	DUF4371 superfamily	-	-
Q#112 - >Salmo_trutta	superfamily	405048	18	204	0.00000000165135	58.0106	cl16778	DUF4371 superfamily	-	-
Q#113 - >Cottoperca_gobio	superfamily	399013	399	452	0.00159404	37.2409	cl05324	Dimer_Tnp_hAT superfamily	N	-
Q#114 - >Labrus_bergylta	superfamily	405048	157	240	0.0000736176	44.5286	cl16778	DUF4371 superfamily	N	-
Q#114 - >Labrus_bergylta	superfamily	436652	5	40	0.00610783	35.3194	cl39976	zf-C2H2_12 superfamily	NC	-
Q#115 - >Erpetoichthys_calabaricus	superfamily	405048	74	267	3.90E-10	60.3218	cl16778	DUF4371 superfamily	-	-
Q#116 - >Podarcis_muralis	superfamily	436652	237	299	0.0000000938011	49.5718	cl39976	zf-C2H2_12 superfamily	-	-
Q#116 - >Podarcis_muralis	superfamily	405048	312	484	0.0000188289	46.8398	cl16778	DUF4371 superfamily	N	-
Q#116 - >Podarcis_muralis	superfamily	445849	1	29	0.000057234	42.4863	cl02605	SCAN superfamily	N	-
Q#116 - >Podarcis_muralis	superfamily	445843	108	146	0.000245834	39.0698	cl02581	KRAB_A-box superfamily	-	-
Q#116 - >Podarcis_muralis	superfamily	399013	749	806	0.00257027	37.6261	cl05324	Dimer_Tnp_hAT superfamily	N	-
Q#118 - >Parambassis_ranga	superfamily	405048	99	180	0.00937634	37.595	cl16778	DUF4371 superfamily	N	-
Q#119 - >Paralichthys_olivaceus	superfamily	436652	17	59	0.000000300894	46.8754	cl39976	zf-C2H2_12 superfamily	C	-
Q#119 - >Paralichthys_olivaceus	superfamily	405048	175	258	0.000293363	41.8322	cl16778	DUF4371 superfamily	N	-
Q#120 - >Hippocampus_comes	superfamily	436652	21	80	0.0000000027717	53.4238	cl39976	zf-C2H2_12 superfamily	-	-
Q#120 - >Hippocampus_comes	superfamily	405048	175	260	0.000000279335	51.8474	cl16778	DUF4371 superfamily	N	-
Q#123 - >Cyprinus_carpio	superfamily	405048	153	267	0.00000327307	48.3806	cl16778	DUF4371 superfamily	N	-
Q#123 - >Cyprinus_carpio	superfamily	436652	20	62	0.000198062	39.5566	cl39976	zf-C2H2_12 superfamily	C	-
Q#124 - >Lates_calcarifer	superfamily	405048	147	230	0.000866292	40.6766	cl16778	DUF4371 superfamily	N	-
Q#127 - >malaclemys_terrapin_terrapin	superfamily	405048	19	102	0.000000606357	50.3066	cl16778	DUF4371 superfamily	N	-
Q#128 - >Trachemys_scripta_elegans	superfamily	405048	35	256	0.000000021094	55.3142	cl16778	DUF4371 superfamily	-	-
Q#129 - >Xenopus_tropicalis	superfamily	436652	14	76	0.0000000251332	49.1866	cl39976	zf-C2H2_12 superfamily	-	-

Q#130 - >Etheostoma_spectabile	superfamily	436652	14	76	0.00000000228116	53.4238	cl39976	zf-C2H2_12 superfamily	-	-
Q#130 - >Etheostoma_spectabile	superfamily	405048	173	254	0.0000118632	46.8398	cl16778	DUF4371 superfamily	N	-
Q#131 - >Scleropages_formosus	superfamily	436652	25	87	0.0000000411232	49.957	cl39976	zf-C2H2_12 superfamily	-	-
Q#131 - >Scleropages_formosus	superfamily	405048	184	267	0.00000132053	49.9214	cl16778	DUF4371 superfamily	N	-
Q#132 - >Thalassophryne_amazonica	superfamily	436652	16	72	0.00000492213	44.179	cl39976	zf-C2H2_12 superfamily	-	-
Q#132 - >Thalassophryne_amazonica	superfamily	405048	173	256	0.000414221	42.2174	cl16778	DUF4371 superfamily	N	-
Q#133 - >Syngnathus_acus	superfamily	436652	14	76	0.0000000505667	49.957	cl39976	zf-C2H2_12 superfamily	-	-
Q#133 - >Syngnathus_acus	superfamily	405048	173	256	0.000000200809	52.2326	cl16778	DUF4371 superfamily	N	-
Q#134 - >Scophthalmus_maximus	superfamily	436652	14	75	1.01E-10	57.2758	cl39976	zf-C2H2_12 superfamily	-	-
Q#134 - >Scophthalmus_maximus	superfamily	405048	95	255	0.00000122574	49.9214	cl16778	DUF4371 superfamily	N	-
