# Supplementary material for: Fake IDs? Widespread misannotation of DNA transposons as a general transcription factor
Source: Genome Biol. 2023 Nov 13;24:260. doi: 10.1186/s13059-023-03102-9 (PMC10641963; doi:10.1186/s13059-023-03102-9)
Supplement: Supplementary file 2 — Additional file 2. DDE/RW positions in new hAT TEs. [file 13059_2023_3102_MOESM2_ESM.pdf]

DDE/RW positions in new hAT TEs

```
CLUSTAL format alignment by MAFFT (v7.505)

DDE/RW residues are marked by '*'


Sphaeramia_orbi M-----------------------------------------------------------
Labrus_bergylta ------------------------------------------------------------
Labeo_rohita    ------------------------------------------------------------
Xiphophorus     ------------------------------------------------------------
Xiphophorus_hel ------------------------------------------------------------
Gouania_willden ------------------------------------------------------------
Lates_calcarife M-----------------------------------------------------------
Anolis_caroline M-----------------------------------------------------------
Kryptolebias_ma ------------------------------------------------------------
Anguilla_anguil ML-QVK---------------------------------------------------LRI
Larimichthys_cr ------------------------------------------------------------
Chelmon_rostrat ------------------------------------------------------------
Maylandia_zebra ------------------------------------------------------------
Salvelinus_nama ------------------------------------------------------------
Salmo_trutta    ------------------------------------------------------------
Oncorhynchus_ki ------------------------------------------------------------
Bufo_bufo       ------------------------------------------------------------
malaclemys_terr ------------------------------------------------------------
Trachemys_scrip ------------------------------------------------------------
Etheostoma_spec ------------------------------------------------------------
Scleropages_for ML-QV------------------------------------------------------I
Syngnathus_acus ------------------------------------------------------------
Thalassophryne_ ------------------------------------------------------------
Scophthalmus_ma ------------------------------------------------------------
Xenopus_tropica ------------------------------------------------------------
Collichthys_luc ------------------------------------------------------------
Paralichthys_ol M-----------------------------------------------------------
Cottoperca_gobi ------------------------------------------------------------
Alligator_missi MN-RAEEETP-MEGPANLEPPWTSPGSLGEMDSLKPEKEQWPKNQGRPRKQMKNVAVNQK
Parambassis_ran ------------------------------------------------------------
Perca_flavescen ------------------------------------------------------------
Gadus_morhua    M-----------------------------------------------------------
Archocentrus    ------------------------------------------------------------
Takifugu        MSHQCQQPSPSLAGGTTAGTP---------------------------------------
Oryzias_latipes ------------------------------------------------------------
Erpetoichthys   -----------MAS----------------------------------------------


Sphaeramia_orbi ------AEGKR---AKTYHFHPEWEQDYFFV-YSHSKPVCLI-CNTTVALAKKGNLEWHF
Labrus_bergylta --------------------------------MSFSKCVCLI-CQSTIAIPKKGNVERHF
Labeo_rohita    --------------------------------MSFSKCICLI-CQSVIAIPKKGNVERHF
Xiphophorus     ------------------------------------------------------------
Xiphophorus_hel ------------------------------------------------------------
Gouania_willden ------------------------------------------------------------
Lates_calcarife -----------------------------------QRKGCLSHLSQAVAVFEEYNLRRHY
```

```
Anolis_caroline ------MSRKRKIDSECRIFKEQWTYDYFFV-NYKERAVCLI-CQNIVSVFKEYNMRRHY
Kryptolebias_ma ------MSRKRKVDADGRLFQERWEGEYLFV-LQGERPVCLL-CYEAVSVVKEYNLRRHF
Anguilla_anguil SAIDPEMSRKRKIDADGRQFQERWEGEYMFV-LQGEKPVCLL-CYEAVSVVKEYNLRRHF
Larimichthys_cr ------------------------------------------------------------
Chelmon_rostrat ------MA-KRK--IDNRTFQDRWEANYLFT-TIKDKPVCLV-CGAGVAVIKEYNIRRHY
Maylandia_zebra ------MA-KRK--NENRSFLDRWETEYLFT-YVKDRPVCLV-CGAHVALTKEYNIRRHY
Salvelinus_nama ------MA-KRK--AENRSFLDKWEAEYLFT-YVKDKPVCLV-CGVNVAVSKEYNIRRHY
Salmo_trutta    ------------------------------------------------------------
Oncorhynchus_ki -------------------------------------------------------MKLHF
Bufo_bufo       ------MA-KRK--ADNRNFLDRWETEYLFT-YVKDRPVCLI-CGVNVAITKEYNIRRHY
malaclemys_terr ------------------------------------------------------------
Trachemys_scrip ------MA-KRKIDSENRGFQSRWENEYMFT-EIAGKPVCLL-CGSNIAVMKEYNLRRHY
Etheostoma_spec ------MA-KRKMYSENRIFQSRWENEYMFT-EIAGKLVCLL-CGSNVAVMKEYNLRRHY
Scleropages_for AKLALTMP-KRKVDSENRAFKNRWEAEYMFT-DIAGKPVCLI-CGANVAVIKEFDLRRHY
Syngnathus_acus ------MP-KRKVDSENRAFKNRWEVEYMFT-DIAGKPVCLI-CGANVAVLKEFNLRRHY
Thalassophryne_ ------MP-KRKVDSENRAFKSRWEAEYMFT-DIAGKPVCLI-CGDNVAVIKEFNLRRHC
Scophthalmus_ma ------MA-KRKVDSENRAFQNRWEAEYMFT-DIAGKPVCLV-CGANVAVIKEFNIRRHY
Xenopus_tropica ------MP-KRKVDSKNRAFKNRWEAEYMFT-DIAGKPLCLI-CGANVAVIKEFNLRRHY
Collichthys_luc -------------------------MQYFFV-EHRGTPTCLI-CTEKVAVHKEYNLKRHY
Paralichthys_ol -----ATVKKRKVDGECRVFQEKWTNDFFFV-EVKGKPVCLV-CGEALAVMKKANVERHY
Cottoperca_gobi ------------------------------------------------------------
Alligator_missi NDLAMSTSKKRKVDTERRVFNKKWTSKYFFT-EMGKKAFCLI-CKESLAVFKEYNLNRHF
Parambassis_ran ------------------------------------------------------------
Perca_flavescen ---MSAHARKRKVDAECRIFNKNWTAKYLFT-EVGGKAVCLV-CGERIAVFKDYNLSRHY
Gadus_morhua    ---------ERQWHTDKRKFKKCWEHDYLFT-EVDSNAICLV-CKQKVAVLKEYNIRRHY
Archocentrus    -------MAKRKKDEEYRTFQQEWTDEFAFV-ERAGSAVCLI-CSDKIASMKRSNIKRHF
Takifugu        TTVSMKRAQKRKNSEENREFNAAWTSAFAFTANDAGLPACLI-CGEKLSNNKKSNVERHF
Oryzias_latipes ------MMDKRKISEDNRTFNATWADSFAFTADETGLPVCLI-CGEILANDKKSNVARHF
Erpetoichthys   ------TKKKRRTEEEHREFNRDWTELFAFICNSDGLPTCLI-CHEKLAHNKKSNLERHF


Sphaeramia_orbi KTVH--RSYERDFPAKTLLRATKVRDLKAQLAARQSIFTTPKTQSKAATIASYRVSHVLA
Labrus_bergylta RTAH--KNYNTNFPPKSELRKRKVKELKCQLSGQQSFFSQPTLKAKAATEASFRVSHLIV
Labeo_rohita    RTVH--KNYDTDFPPKSELRKRRVKELKSQLSGQQSFFSQLTSKGKAATEASFRVSHLIV
Xiphophorus     ------------------------------------------------------------
Xiphophorus_hel ------------------------------------------------------------
Gouania_willden ------------------MRADKLAKLKSGLLTQQNTFVRQAQLNQASIRAGFWVAQLIA
Lates_calcarife ESRH--KDKYDSL--QGQMRADKLSKLKSGLLAQQNTFVRQAQLNQSSVRASFRVAQLIA
Anolis_caroline QTQH--KDKYDCL--VGEVRKDKILKLKNILTTQQNTFVKQKQLNISSLRASFQVAKLIA
Kryptolebias_ma DTKH--GAKYAQA--SLQEKQQIAQELKGKLRSQQSLFTKSTAKNEAAVKASFIVAKEIA
Anguilla_anguil DTKH--GAKYAKV--SLHEKQQIVKELKGKLRSQQNLFTKATTKNDAAVKASFMVAEEIA
Larimichthys_cr ------------------------------------------------------------
Chelmon_rostrat ETKH--YEKYKDL--DLKQKLKKVEEMKRSLVSRQTLFTKAKSKSEAAVKASFIVAAEIA
Maylandia_zebra ETKH--QEKYKDL--DMTQRRRKAEEMKRSFVSQQTMFKKAISQSEAAVKASFVVAAEIA
Salvelinus_nama ETKH--HDKYKDL--DMTQRSQKIEEMKRSLVSQQNMFKKATSQSEAAVKASYIVAAEIA
Salmo_trutta    -MKH--HDKYKDL--DMTQRSQKVEEMKRSLVSQQNMFKKATSQSEAAVKASYIVAAEIA
Oncorhynchus_ki -LGHGCQTLARDL--DMTHRSQKVEEMKRSLVSQQNMFKKATSQSEAAVKASYIVAAEIA
Bufo_bufo       ETKH--HDKCKDL--DMTQKSQKVEEMKRSLVSQQNMFKKATSQSEAAVKASYIVAVEIA
malaclemys_terr ------------------------------------------------------------
Trachemys_scrip ETKH--ENKFKNL--SAGQKLQKVEELKKNLTSQQTFFTKAKSQSEAAVKASFIVAEEIA
Etheostoma_spec ETKH--EDKLKNL--SAGQKLQKVEELKKNLTSQQTFFTKAKSQSEAAVKASFIVAEEIA
Scleropages_for ETKH--QDNLKDL--NAEQKIQKAEELKKNLTLQQMFFTRAKSQSEAAVKASFIVAEEVA
```

```
Syngnathus_acus ETKH--LDNLKDL--NAEQKIQKVEELKKKLTFQQTFFTRAKSQSEAAVKASFIVAEEIA
Thalassophryne_ ETKH--QDNLKDL--NAEQKIQKAEDLKKNLTLQQTFFTRAKSQSEAAVKSSFIAAEEIA
Scophthalmus_ma ETKH--QE-LQNL--NAEEKIQRVKELKKNLRFQQTFFTRAKSQSEAAVKASFIVAQEIA
Xenopus_tropica ETKH--QDNLKDL--NAEQKIQKVEELKKNLTLQQTIFTRAKSESESEAAVKASFIVAEEIA
Collichthys_luc TTRH--AEEYEKY--QGDERANRIANLKTCLLRQQDFFKKATKESNAAVKASYMVSEMIA
Paralichthys_ol SSKH---AKLDEL--KGQMXLDKINALRRSLGAQQAAXTRPQTDRENITRASFVVSELIA
Cottoperca_gobi ------------------------------------------------------------
Alligator_missi ETKH--ASKYDKL--SAQEKTKKAEEMVAAMQKEQSFSPKALEVRDGIMRTSYEMAHIIA
Parambassis_ran ------------------------MLLAKLQTQQGFFTKLHTSRDAATKTSFLISHKIA
Perca_flavescen ETTH--AEKYKNL--SDAERARTSEALLAKLQKQQGFFTKLHTSRDAATKTSFVISHKIA
Gadus_morhua    ETMH--SHQFAKY--TGEDRKVKAANLLIKLETQQQTLLQPSTAQENATLASYRISNIIV
Archocentrus    DTRH--TTFASKYP-AGDSRKKACQELLCKVQASQQQLRVWTQQ-GDWNSASFAGALAIV
Takifugu        QSKH--LAFAEKYP-TEDERQRAISELQRQAEERKLSFKKWISSPQSTTAASFLAAQEIV
Oryzias_latipes ENKH--SAFAKKYP-KGEERKKAVSELMQKADVSRSHFKKWIKTGNSTTCTSFVVAQEIV
Erpetoichthys   TKKH--TQFASKYP-AGEERKKAVDELQKQKQQSSSMLSNWTQSTSNVNLASFAVSLEIA


Sphaeramia_orbi KHNKPFKDGDIVKEAFLEAADSLFDHFKNKTEIVDAIKGVQLSRNTATRWCEGMAVDVEE
Labrus_bergylta KNKKSFQDGEMVKEAFVEAADSLFRDFKNKAEILSSIKALQLSRSTVTRRSEAIAEDLTQ
Labeo_rohita    KNKKSFQDGEMVKEAFVEAADSLFRDFKNKPEILSSIKALQLSRSTVTRRSEAMAEDLTQ
Xiphophorus     -----------------------------------------MSRHTVETRISDINNALES
Xiphophorus_hel -----------------------------------------MSRHTVETRISDINNALES
Gouania_willden SSGKPFTDGEFVKKCLNAVVEEVCPDKKDVF------NAVSLSASTITRRIEEIGGNVYA
Lates_calcarife SSGKPFTDGEFVKKCMNAVT-EVCPEKKNVF------NAVSLSVSTITRHIEEIGGNVYA
Anolis_caroline CTGRPFVEGEFVKECLLSVAKEMCPEKADLF------STVSLSGSTITRRIEEMGDNLHQ
Kryptolebias_ma QASKSFSEGAFLKQCMLKVCEQVCPDQLQTF------KNVSLSRNTIANRVKELAENLTT
Anguilla_anguil RASKSFSEGAFLKQCMLKVCEQVCPDQIQTF------KNVSLSRNTIADRVKELAANLST
Larimichthys_cr ------------------------------------------------------------
Chelmon_rostrat KSARPFNEGEFVKKCMVKVCDIVCPDKKQEFL----SKNVSLSRNTMAERVCELSTNLHE
Maylandia_zebra KSARPFSEGEFVKSCMMK----VCPEKKQVF------SNVSLSRNTVADRTRELANDLNN
Salvelinus_nama KSARPFNEGEFVKKCMMKVCDLVCPEKKQAF------SNVSLSRNTVADRTCDLATNLYD
Salmo_trutta    KSARPFNEGEFVKKCTMKVCDLVCPEKKQAF------SNVSLSRNTVADRTCDLATNLYD
Oncorhynchus_ki KSARPFNEGEFMKKCMMKVCDLVCPEKKQAF------SNVSLSRNTVADRTCDLATNLYD
Bufo_bufo       KSARPFNEGEFVKKCMLKVCDIVCPEKKQAF------SNVSLSRNTVADRTCDLATNLYD
malaclemys_terr -----------------------------------------------------MATDLKT
Trachemys_scrip KSGRPFTEGEFVKNCMMKVCDVLCPDKTRAF------ANVSLSRNTVANRVCEMATDLKT
Etheostoma_spec KSGRPFTEGEFVKNCMMKVCDVVCPDKTRAF------ANVSLSRNTVANRVCEMATDLKT
Scleropages_for KSARPFTEGEFLKSCMIKVFDVLCPDKKQML------ANVSLSRNTIADRVREMATDLRT
Syngnathus_acus KAGRPFTEGEFLKSCMVKVCDIICPDKKQML------ANVSLSRNTVADRVCEMATDLRT
Thalassophryne_ KSARPFTEGEFLKSCMIKVFDVLCPDKKQIL------ANVSLSRKMIADRVCEMATDLRT
Scophthalmus_ma KSARPFTEGEFLKSCMMKVCDVLCPENKQMF------ANVSLSRNTVADRICEMATDLKT
Xenopus_tropica KSARPFTKGEFLKSCMIKVFDVLCPDKKQML------AN---------------------
Collichthys_luc TAGKPFTEGEFVKKCILQAASIICPEKKGQF------SNISLSANTVAERISDLSNDIYD
Paralichthys_ol TKLKPHAEGEFVKECLVAAAELLAPDKVKSF------QSVSLSRRTVSERITDMAQDIEK
Cottoperca_gobi ---------------MVETSELLCPESKGKF------EKISLSRRTVTRRVELIDEDIVR
Alligator_missi KKSKPFSEGEFVKECMLKSAAILCPDKKEQF------ENVRLSRRTIVRRVEDISENLHQ
Parambassis_ran KNSKPFSEGEFVKECLVDSAALICPDKKGAF------EQVPLSRRTVTRRIEQIAGNLEL
Perca_flavescen KNSKPFSEGEFVKECLVDSAALICPEKKGAF------EQVPLSRRTVTRRIEEIAGNLEL
Gadus_morhua    RSGHAFAAGDFVKECLTVAAEAVCPTQKRAF------SQISLSRNTVTRRVEDMAEDVRG
Archocentrus    RNGKPFTDGEYAKTFMLDVASELFDDFSDKDKIMKRIKDMPLSARTVHDRTIMMANQIEE
Takifugu        KRGKPFTDGEYIKETFIQISEHLFSDFKNKNEIVQKIKDMPLSAKTVKDRTIKMAANISS
Oryzias_latipes KHGKPFTDGEYLKETFIKISKHLFSDFKNKDEILQKIKDMPLSAKIVQDRSVNMAENVTR
```

```
Erpetoichthys    KKGKPFTDGEYVKDCFIRASEELFRDFKNKPEILKKIKDLPLSAKTVQDRIAKMSSNVTY


Sphaeramia_orbi QLRRDIDACECFSLQFDESTDMVDVAQLCVFIRMVFEDMSTKEELLTILPLKGHTRGEDI
Labrus_bergylta QLWKDIADCECFSLQLDESTDVSDTAQLCIFIRMVFTDMTAKEELLTLLPMKEHTRGEDI
Labeo_rohita    QLWKDITDCECFSLQLDESTDVSDTAQLCIFIRMVFNDMTAKEELLTVLPMKEHTRGEDI
Xiphophorus     DLHADLNACAYFSVALDESCDIQDKPQLAIFVRMISEDCVVKEELLDIVPLKDRTRGTDV
Xiphophorus_hel DLHADLNACAYFSVALDESCDIQDKPQLAIFVRMISEDCVVKEELLDIVPLKDRTRGTDV
Gouania_willden QLQQKMKEFDFFSLAMDESTDVQDTAQLLIFICGVSANFEMCEELAALQSLKGTTMGEDI
Lates_calcarife QLQQKMKEFEFFSLALDESTDVQDTVQRLIFIRGVSANFEMCKELAALQSLKGTTTGEDI
Anolis_caroline HLQNSAKKLSYFSLALDESNDVRDSAQLLIFIRGTNDYFEVTEELAALQSIKGTTTGEDI
Kryptolebias_ma QLAEETRSYTAFSLAVDESTDNTDTAQLSIFIRGVKSDLSITEELLDVAALHGTTTGQDI
Anguilla_anguil QLAEESRSYTAFSLAVDESTDNTDMAQLSIFIRGVKSDLSVTEELLDVAAMHGTTTGRDI
Larimichthys_cr --MRKGEDFVAYSLAVDESSDTSDTAQLSIFIRGVDSNLCVTEELLGLKSMHGTTTGKDI
Chelmon_rostrat KLIKKGKDFIAYSLAVDESSDTSDTAQLSIFIRGVDSSLCVTEELLGLKSVHGTTTGKDI
Maylandia_zebra QLMEKGNSFVAFSLAMDESSDASDTAQLSVFIRGVDSTLCVTEELLGLKSMHGTTTGKEI
Salvelinus_nama QLMEKGKDFVAFSLAVDESCDASDTAQLSVFIRGVDSNLCVTEELLGFKSMHGTTTGKEI
Salmo_trutta    QLMEKGKDFVAFSLAVDESCDASDTAQLSVFIRGVDSNLCVTEELLGFKSMHGTTTGKEI
Oncorhynchus_ki QLMEKGKDFVAFSLAVDESCDASDTAQLSFFIRGVDSNLCVTEELLGFKSMHGTTTGKEI
Bufo_bufo       QLLEKGKDFVAFSLAVDESSDASDTAQLSVFIRGVDSNMCVTEELLGFKSMHGTTTGKEI
malaclemys_terr QLIERAKDFVAYSLAVDETTDATDTAQLAIFIRGVDSNLCVTQEILDIKSMHGTTKGEDI
Trachemys_scrip QLIERAKDFVAYSLAVDETTDATDTAQLAIFIRGVDSNLCVTEEILDIKSMHGTTKGEDI
Etheostoma_spec QLIERAKDFVAYSLAVDETTDSTDTAQLAIFIRGVDSNLCVTEEILDIKSMHGTTKGEDI
Scleropages_for QLSERSKDFIAYSLAVDESTDMTDTAQLAIFIRGVDSNLRVTEEIMDIKSMHGTTTGKDI
Syngnathus_acus QLSKRSKDFIAYSLAMDESTDMTDTAELAIFIRGVDSDLRVTEEILDIKPMHGTTTGKDI
Thalassophryne_ QLSERSKDFIAYSLAVDESTDMTDTAQLAIFIHGVDSNLRVTEEIMDIKLMHGTTTGKDI
Scophthalmus_ma QLSERSKDFTAFSLAVDESTDMTDTAQLAIFIREVDSSLCVTEEILDIKSMHGTTTGKDI
Xenopus_tropica ------------------------------------------------------------
Collichthys_luc QLCEKAKCFSAYSVALDETTDITDTAQLAMYVRGVNDNFEVMEELLRVIPMHGQTTAQEI
Paralichthys_ol TLKNTARDFEYFSLACDETTDITHTAQLAIFLRGITSEFETKEELLSLQAMHGTTKGEDL
Cottoperca_gobi ELNKKVESFKLYSLALDESNDIKDTAQLLIFIRGINDSFEITEELLSMESLKGKTRGEDL
Alligator_missi QLKSKVNDFSYFALALDDSCDVKGTDQLLIFIRGITENFELTEELALIQSMKDTTTGKDL
Parambassis_ran QLHREVAGFDFFSLALDESCDVRDTAQLLIFVRGITD-FKITEELAAMRSMKGTTTGSDL
Perca_flavescen QLQREVACFDFFSLALDESCDVRDTAQLLPWDYGLQDY----------------------
Gadus_morhua    QIAQRAGRFSAFSIACDESTDISDSAQLLVFLRGVNEDFEVCQELAGLETLKGTTRGIDI
Archocentrus    TQVKDINAAVFFSLALDESTDVSHLSQFSVIARYAVGD-TLHEESLAVLPMKGTTRGEDL
Takifugu        KQIDDINSAQAFSIGCDESSDVNDIEQIALLCRYANAN-GPQEELIELIPLKGQTRGQDI
Oryzias_latipes QQIEDINSALAYAIACNMSKDKNDIEQIALFCRYVSAA-GPQEEMIELIPLKGQTRGEDI
Erpetoichthys   LQVEDIQLSSALSLAIDESCDIKDTAQVALFVRYMSSQ-GPKEELLGLLPLSGQTRGEDI
                                *

Sphaeramia_orbi FNAFMGFVSGTKLPLFKLISITTDGAPSMVGRTSGFIALCK----ESESF-PDILNYHCI
Labrus_bergylta FQSFNNFITKTQLPVYKLVSITTDGAPAMVGRVNGFIAKCR----EDDAF-PDFLNYHCI
Labeo_rohita    FQSFKNFMEKTRLPVCKLVSITTDGAPAMVGRSNGFIAKCR----EDDAF-PDSLNYHCI
Xiphophorus     KEAMMEVFKTANMSLEKLTAIATDGAPSMIGSVNGLVGLCKG----DDLF-PDFWNFHCI
Xiphophorus_hel KEAMMEVFKTANMSLEKLTAIATDGAPSMIGSVNGLVGLCKG----DDLF-PDFWNFHCI
Gouania_willden FGKVCQTMEELDLDWSKLASITTDGAPSLVGAFRGLIGRMNREIEKRGLT--APLQVHFL
Lates_calcarife FGKVYRTMEELDLDWSKLASITTDGAPSMVGMFRGLIGRMNREMEERGLI--APLQVHCL
Anolis_caroline YEKLCQTVNDLELDWAKLASVTTDGAPSMVGSKKGVIARINQEMDKHNHS--HPIAIHCL
Kryptolebias_ma FDAVEKSVSKNALQWENLVGLTTDGAPAMCGEKAGLVGLMRRKMQKMNCH-TPLITYHCI
Anguilla_anguil YDAVEKSVSKTGLPWEKLVGLTTDGAPAMCGGKAGLVGLMKEKMQKSNCH-TPLITYHCI
Larimichthys_cr FEEVSKCVDEMKLPWDKLVGLTTDGAPAMCGQKSGLVGRMREKMREKQ-A-GELTVYHCI
```

```
Chelmon_rostrat FEEVSKCITEMSLPWDKLVGLTTDGAPAMCGQKSGLVGRIQEKMREED-A-GKLTVYHCI
Maylandia_zebra FEEVSKCLTEMKLPWEKLVGLTTDGAPAMCGQRSGLVGRVREKMRAENCA-GELTVYHCI
Salvelinus_nama FEEVSKCVTEIKLPWDKLVGLTTDGAPAMCGKKSGLVGMVREKMREENCA-GELTVYHCI
Salmo_trutta    FEEVSKCVTEIKLPWDKLVGLTTDGAPAMCGKKSGLVGMVREKMREENCA----------
Oncorhynchus_ki FEEVSKCVTEIKLPWDKLVGLTTDGAPAMCGKKSGLVGMVREKMREENCA-GELTVYHCI
Bufo_bufo       FEEVSKCVTEIKLSWDKLVGLTTDGAPAMCGKTSGLVGRVREKMQEENCA-GELTVYHCI
malaclemys_terr FGNVFQSVTDMKLPWEKLIGLTTDGAPAMCGEKNGLVGRMRSKMREENCA----------
Trachemys_scrip FGNVFQSVTDMKLPWEKLVGLTTDGAPAMCGEKNGLVGRMRSKMREENCA-GELTVYHCI
Etheostoma_spec FGNVFQSVTDMRLPWEKLVGLTTDGAPAMCGEKKWTGGKDALK----DAG-GELC-----
Scleropages_for FENVCQSITDMKLPWDKLIALTTDGAPSMCSEKSGLVGRMRVKMQEENCT-GELTAYHCI
Syngnathus_acus FENVCQSVTDMKLPWDKLIGLTTDGAPAMCGEKSGLVGRMREKMQEENCT-GELTTYHCI
Thalassophryne_ FENVCQSITDMKLPWDKRIALTTDGAPSMCSEKRGLVGRMRVKMQ-ENC-----------
Scophthalmus_ma FENVCQSITDMKLPWDKLIGLTTDGAPAMCSEKVGLVGRMRAKMQEENCT-GELTAYHCI
Xenopus_tropica ------------------------------------------------------------
Collichthys_luc FCQLCDAIKNAGLPWKRFVGITTNGALSMTRRKNGLVALVKKKLEEEGVE--EAIALHCI
Paralichthys_ol FNQVIVAMNNFELPFEKLSGIATDGAPAMVGXQKGLTALVKKEMSRLSLDPSDLVVCHCI
Cottoperca_gobi YEQVSAVIERMKLPWSKLANVTTDGSPNLTGKNVGLLKRIQDKVKEENPD-QDVIFLHCI
Alligator_missi LEAVDQCVSKLGTDWKKLVSVTTYGSPNLAGKDVGLLKIIQDQVKETDPE-QKIILLHCI
Parambassis_ran FMEVNACIDTLGLKWDRLAGVTTDGCPNLT------------------------------
Perca_flavescen -----------------------GGAGSNA------------------------------
Gadus_morhua    FKAVEQVMDKNGLKWENLSGITTDGAPAMVGKRAGLTALVSDKVRECG---GSIFKYHCI
Archocentrus    FKSFTEFAKGKNLPMDKLISVCTDGAPCMVGKNRGFVALLREHEK------RPILSFHCI
Takifugu        CDAVVSCLKDKGINTTHLVSVSIDGAPSMRGAQKGFVNLLQKSLG------RDLMTFHCI
Oryzias_latipes CEAVLHCLRTKEIKTTHLVPVASDGAPSMTGAQKGFVALLQKSLD------RKLLTFHCI
Erpetoichthys   ANAVQKCLEDNKIDLNKIVSIATDGARSMTGKNKGATTILQSKIN------HEILTFHCI
                                       *

Sphaeramia_orbi IHQQVLCGKIL--NMKEVMDVAMKIVCSVRARSLQRRLFRAHLEENDAEH-TDLLLHTDV
Labrus_bergylta IHQQALCAKML--NMKEIMDVAMKIACSIRARSLQRRLFRAHLEQADCDH-SELLLHTDV
Labeo_rohita    IHQQALCAKML--NMKEIMDVATKIACSIRARSLQRRLFRAHLENADCDH-SELLLHTDV
Xiphophorus     IHREQLVSKTL--NLDHVMKPMMEIVNYIRTHALTHRQFKNLIADLDGDLPGDLPLHCAV
Xiphophorus_hel IHREQLVSKTL--NLDHVMKPMMEIVNYIRTHALTHRQFKNLIADLDGDLPGDLPLHCAV
Gouania_willden IHQQTLCCKVL--KWDSVMKVVVSCINFIRANGLKHRQFQQFLTELESAH-GDVLYYTEV
Lates_calcarife IHQQALCCKVL--TWDSVMKVVVSCINFIRAKGLKHRQFQEFLSELEPAH-GDVLYYTEV
Anolis_caroline IHQQALCSKSL--KWDSVMKIVVSCVNFIRANALNHRQFQEFLSELNAAY-EDVLYHTEV
Kryptolebias_ma IHQEALCGKVL--GMEDIVTTVMKTVNFIRARGLNHRQFQQFLLEMGSEH-GDVPYHTEV
Anguilla_anguil IHQEALCGKVL--GMDDIMTTVMKTVNFIRARGLNHRQFQLFLQEMGSEY-GDVPYHTEV
Larimichthys_cr IHQEALCGKAL--KMDHIMSTVTQVVNFIKAKGLNHRQFKSFLEELGADH-SDMPYHTEV
Chelmon_rostrat IHQEALCGKAL--QMEHVMSSIKGVVNFIRAKGLNHRQFKSFLEELDSEY-RDVPYHTEV
Maylandia_zebra IHQESLCGKAL--KMEHVMTAVTRVVNFIRAKGLNHRQFKSFLEECGSEY-GDVPYHTEV
Salvelinus_nama IHQESLCAKAL--KMEHVMTTVTQVVNFIRAKGLNHRQFKFFLEECGSEY-ADVPYHTEV
Salmo_trutta    -------------------------------------------EECGSEY-ADVPYHTEV
Oncorhynchus_ki IHQEALCAKAL--KMEHVMTTVTQGVNFIRAKGLNHRQFKSFLEECGSEY-ADVPYHTEV
Bufo_bufo       IHQESLCGKAL--KMEHVMTTVTQVVNFIRAKGLNHRQFKSFLEECGSAY-SDVPNQTEV
malaclemys_terr ------------------------------EPRLNHRQFQSFLREIDSEF-GDMPYHTEV
Trachemys_scrip IHQESLSAKVL--KMDHVMNTVTQTVNFIRAHGLNHRQFQSFLREIDSEF-GDMPYHTEV
Etheostoma_spec -----------------------------RAHGLNHRQFQSFLREIDCEF-GDMPYHTEV
Scleropages_for IHQEALCGKVL--KMDNVMSTLTQTVNFIRAKGLNHRQFQSFMREIDSEF-ADIPYHTEV
Syngnathus_acus IHQEALCGKVL--KMDHVMSTVTQTVNFIRSRGLNHRQFQSFMRTIDSEF-ADIPYHTEV
Thalassophryne_ ---------------------------------------------------------TEV
Scophthalmus_ma IHQEMLCCKVL--KMEHVMNTVTQTVNFIRAKGLNHWQFQSFMREIDSEF-ADIPYHTEV
Xenopus_tropica ------------------------------------------------------------
```

```
Collichthys_luc IHQQALCSKCL--KFDNVMSVVVKCINQIKSRGLKHRRFRAFLEEMESEY-GDVLYFTEV
Paralichthys_ol IHQESLCAHSL--KLNNVMTTVVSTINFIKGRGLNNRQFKELLSEIESEY-GDLVYHCEV
Cottoperca_gobi IHQESLCKSVL--QLNHVVDPVVKLVNFIRARGLNHRQFITFLEETDADH-QDLLYHSRV
Alligator_missi IHQEVLCKSVL--KLSTVVDTVTKVVSYIRARGLNHRQFATLLEGSESEH-TDVLHDTSV
Parambassis_ran -------------------------------------------EEHESEH-SDIGYHTAV
Perca_flavescen -----------------------------------------VDERNND-----------
Gadus_morhua    LHQEQLCAKNI--GLKNVMQDVVGIVNNIRSKALSHRQFKAVVDEMDAQY-GDVLYHQEV
Archocentrus    LHQEALCAQMCGEQLGEVMSLVIRVVNFIVARALNDRQFKTLLDEVGNNY-PGLLLHSNV
Takifugu        IHQEALCAQTFPPECVEVMNLVIKIVNKIIANGLSHRQFCSLLEEVENTY-SDLLLHNRV
Oryzias_latipes LHQEALCAQTFPPECTQVMDLVIQIVNKIMANGLNHRQFRSLLDELDSAY-SDLLLHNKV
Erpetoichthys   IHQEALCAQTFPAEIVEVMNLVIKIVNSILSKALHHRQFKEFLNEMETQY-SDLLLHNKV


Sphaeramia_orbi RWLSRGKFLDRFMELLPEIKDFLRLSKHM--DYHTKLEDHQWLLDLSFLTDLTGELSELN
Labrus_bergylta RWLSRGKFLQRFRELCPEIKEFFRVAGHA--EY-KQLNDGQWLLDLAFLTDLTNLLNDLN
Labeo_rohita    RWLSRGKFLQRFRELCPEIKEFLRVAKHA--EY-SQLNDNLWLLDLAFLTDLTNMLNDLN
Xiphophorus     RWLSRGKVVSRFLELLEPVKLFM-AEKNK--SY-PQLSDPKWLMDLAFLVDMLSHLDKLN
Xiphophorus_hel RWLSRGKVVSRFLELLEPVKLFM-AEKNK--SY-PQLSDPKWLMDLAFLVDMLSHLDKLN
Gouania_willden RWLSRGRVLRRFYELLPEINAFL-HLKDK--TI-PELIDPEWKWHLAFLTDVTEMLNSLN
Lates_calcarife RWLSRGRVLRRFYELLPEINAFL-HSQNK--TV-PELIEPEWKWHLAFLTDMTEMLNSFN
Anolis_caroline RWLSRGRVLKHFYDLLPQITAFL-LSKNK--EV-PELNDAEWKWHLAFLTDITELLNSFN
Kryptolebias_ma RWLSRSKVLKRFFELREDIAFFM-QSKGK--LM-SELSDPKWLCDFAVLCDITDHLAQLN
Anguilla_anguil RWLSRSKILKRFFELREDIALFM-QSKGK--PL-SELSDPNWLCDFAMLCDITEHLAQLN
Larimichthys_cr RWLSRGKVAKRFFELREEICLFM-ESKGK--DT-TELRDEKFQCELAFLCDIMNHLDALN
Chelmon_rostrat RWLSRGKVLNRCFELREEICQFI-ENKGK--DT-TELRDEKFLCELAFLSDIVSHLDVLN
Maylandia_zebra RWLSRGKVLNRCFELREEIFQFL-ESKGQ--DT-AELREQEFLCELAFMCDITSHLDALN
Salvelinus_nama RWLIRGKVLNRCFELREEICQFL-ETKGK--DT-AELREQKFLCELAFLCDISSHLDALN
Salmo_trutta    RWLSRGKVLNRCFELHEEICQFL-ETKGK--DT-AELREQKFLCELAFLCDISSHLDALN
Oncorhynchus_ki RWLSRGKVLNRCFELSEEICKFL-ETKGK--DT-AELREQKFLCELAFLCDISSHLDALN
Bufo_bufo       RWLSRGKVLNRCFELHEEICQFL-ESKGK--DT-AELREKKVLCELAFLCDISSHLDALN
malaclemys_terr RWLSRGKVLKRHFELREEICQFM-DSKGK--DC-TVLRDEKWKCELVFLADITSHLSALN
Trachemys_scrip RWLSRGKVLKRHFELREEICQFM-DSKGK--DC-TVLRDEKWKCELAFLADITSHLSALN
Etheostoma_spec RWLSRGKVLKRHFELREEICQFM-DSKGK--DC-TVLRDEKWKCELAFLADITSHLSALN
Scleropages_for RWLSRGKVLDRVFELSNEICQFI-DSKGK--DS-TNFRDEKWKCELAFLADITAHLNALN
Syngnathus_acus RWLSRGKVLNRVFELSNEICQFM-DSKGK--DS-TVLRDEKWKCELAFLADITAHLNALN
Thalassophryne_ RWLSWGKVLSRVFELSNKICQFM-DSKGK--DS-TNFRDEKWKCKLAFLADITAHLNALN
Scophthalmus_ma RWLSRGKVLNRVFELSKEICQFM-DSKGK--DT-TVLRDEKWKCELAFLADVTAHLNVLN
Xenopus_tropica ------------------------------------------------------------
Collichthys_luc RWLSRGNVLKRFFELRAEVKAFM-EKDGV--AV-PVLSDPKWLMDLAFLVDITHELNVLN
Paralichthys_ol RWLSRANMLAGFIHC-------------------GKKSDASWR-----------------
Cottoperca_gobi RWLSLGKVCQRVWELKEEIRSFL-EQMGKSDEF-PELSDTDWLCDFAFAVDILSHMNELN
Alligator_missi SWLSLGNILKRVWELRAEIGLFL-NMKED-LDF-PQLNDAEWLSDFAFAIDVTGYMNDLN
Parambassis_ran RWLSLGKVLKRVWDLNAEIREFC-EKKGK--DI-PELSDENWMADFAFAVDVTALMNELN
Perca_flavescen ------------------------------------------------------------
Gadus_morhua    RWLSRGKVLRRFFELREEIRVFQ-ATKEN--NI-QVPSDKHWIADLAFLVDITELLNILN
Archocentrus    RWLSRGKVLSRFAACLSEIRTFL-EMKNI--EH-PELANTEWLLKFYYLVDMTEHLNQLN
Takifugu        RWLSSGEVLKRFAACLEHVKTFL-GNKGL--GY-PELEDPSWLEKFYFMVDMTSYLNMLN
Oryzias_latipes RWLSRGVVLKRFAACLEEVKVFL-SNKGL--TF-PELEQPEWQEKLHFMVDMTAHLNTLN
Erpetoichthys   RWLSKGKVLKRFALCLNEINTFL-NEKGI--NH-PELENDKWLQKFYFMVDITAKLNELN
                **

Sphaeramia_orbi LELQGKGKDVVNMMSSVSTFKSKLKLMSNRLRLGNLCNFPHMQAELQRQGKGVT----QL
```

```
Labrus_bergylta LELQGKDKTVINMISSVNAFKRKMQHLSSKLRRHDLANVQNLASELEMQGKACV----QL
Labeo_rohita    LELQGKDKTVTNMISSVNAFKRKMQHFSSKLQRHDLANFQNLVSELEMQGKSCV----QL
Xiphophorus     LDLQGKLKTLPDLTQSVFSFVNKVKLFKVHIQNGNLSHFPSLRSA---QQKAGI----RM
Xiphophorus_hel LDLQGKLKTLPDLTQSVFSFVNKVKLFKVHIQNGNLSHFPSLRSA---QQKAGI----RM
Gouania_willden LQLQGQGKLICDMYSHIKAFEVKLALLLEQVKKHNFIHLPATQNL--STENPAV----PF
Lates_calcarife LQLQGQGKLICEMYSHIKAFEVKPELLLGQVKKHSFIHLPATQNL--SAENPEV----PF
Anolis_caroline VQLQGKGKLICDMQSHVKAFEVKLGLLIKQVKEENFCHLPTTQSL--SAEKPLI----AF
Kryptolebias_ma QKLQGRKQVITQMSDTITAFQRKLDLWMWQVKQDNLVHFPVCQSM--SASFPET-----F
Anguilla_anguil QKLQGRKQVITQMSDMITCFQRKLDLWKWQVEQDNLAHFSVCQSI--SASVPDA-----F
Larimichthys_cr LQLQGRAHVITDMYYAVRAFRTKLRLWESQMQQGNLAHFLCCQVM--KEQTATA----VM
Chelmon_rostrat LQLQGRGHVITDMYAAVKAFKTKLRLWKTQMLQG---HFPCCQTM--AAQISPD----AL
Maylandia_zebra LQLQGRGRIITDMYTAVRAFKTKLCLWQNQMLQGNLGHFPCCQMM--NMQISTA----VF
Salvelinus_nama LQLQGRGRIITDMYAAVRAFKTKLCLWENQMLQGNPCHFPCCQTI--KAQISTA----VF
Salmo_trutta    LQLQGRGRIITDMYAAVRAFKTKLCLWENQMLQGNPCHFPCCQTI--KAQISTA----VF
Oncorhynchus_ki LQLQGRGRIITDMYAAGRAFKTKLCLWENQMLQGNPCHFPCCQSI--KAQISTA----MF
Bufo_bufo       LQLQGRRRIITDMYAAVRAFKTKLCLWENQMLQGNLGHFPCCQTM--KTQFFTN----LF
malaclemys_terr LQLQGREHIITDMHDAVKAFQVKLRLWETHMHQCNLSHFPCCQVI--RNQESAT----VF
Trachemys_scrip LQLQGREHIITDMHDAVKAFQVKLRLWETQMHQCNLSHFPCCQVI--RNQESAT----VF
Etheostoma_spec LQLQGREHIITDMHDAVKAFQVKLRLWETQMHQCNLSHFPCCQVI--LNQESDT----VF
Scleropages_for LQLQGRDRMITDMYDAVKAFQVKLLLWETQMRQCNLPHFPCCQVM--LNQVGTT----VF
Syngnathus_acus LQLQGRDRMITDMYDAVKAFQVKLILWETQMLQCNLSHFPCCQVM--LNQVGTT----VF
Thalassophryne_ LQLQGRDRMITDMYDAVKAFQVKLLLWESQMHQCNMSHSPCCQVM--LNQAGTT----VF
Scophthalmus_ma LHLQGRDRIITDMYDTVKAFKVKLLLWETQMRQSNLPHFPCCQVM--FNQVGAT----VF
Xenopus_tropica ------------------------------------------------------------
Collichthys_luc KKLQGQGQLVSAAYDNVRAFSTKLVLWKAQLSQTNLCHFPACKAL--AD--AGT----PF
Paralichthys_ol ------------------------------------------------------------
Cottoperca_gobi VKLQGKDQFVHDMYTNVRAFKSKLILFSRQMANKSFAHFPTLAMQ--KEAT--------R
Alligator_missi SRLQEKGLFAHELHSNIKSFMMKLLLFSRQIGNKDFSHFPTLQQV--TVSD--------Q
Parambassis_ran TKLQGKGLFVHQMHSLVKAFMTKLQLLSRQLESNTLTHMQTLKEV--TPSA--------D
Perca_flavescen ------------------------------------------------------------
Gadus_morhua    LQLQGRDQIITQLYDHVRAFKQKLQLLNRHLLTGNLAHFPSLREV--GLME--------E
Archocentrus    VKMQGIGNTVLSLQQAVFAFENKLELFIADIETGRLLHFEKLGEF--KDACTASDPAQHL
Takifugu        KNLQGQGSTALHMLEEILAFERKMTVFARDVQNGTLSHFPSLREF--KEANNH------I
Oryzias_latipes TSLQGKGGTALHMLEEVLGFERKLTVFATDLKRGTLYHFPALREF--QQAGNA------I
Erpetoichthys   LKLQGKGNPAYVLVEELVCFEEKLILFAEDIQSGKLLHFQFLKQY--RDKTSAT-----V


Sphaeramia_orbi DSARYEEHVQSISSEFERRFTDFASIEPIASYMCYPFGASIDVGDIAAKVKSLFDFESST
Labrus_bergylta DSARYTEQIDKCLSEFDKHFQDFFLLEPIATFMCYPFREDAEVDSLASKISTLFHLNSSG
Labeo_rohita    DNARYKEQIDNCLSEFDKRFQDFALLEPIATFMCYPFQEDAEVVSLASKIATLFHLNSSG
Xiphophorus     NMGQYVVKMLAQLYGSITSRFEDLQQKRPQIALLIDPFNAEADCLKSPLVT------DEAA
Xiphophorus_hel NMGQYVVKMLAQLYGSITSRFEDLQQKRPQIALLIDPFNAEADCLKSPLVT------DEAA
Gouania_willden PAEKCVEALEMLKAEFGVRFRELHVNAKEIRLFQNPFVADIDEAQ-------------PS
Lates_calcarife PAEKCVEALEMLKAEFGVRFRELRVYAKEIRLFQNPFVADID------------------
Anolis_caroline PNKTCVDSLERLQKEFQFRFKELHLHEQDIQLFRNPFSIDIENVD-------------TI
Kryptolebias_ma SCAQLATKLSWLKTEFDRRFSDFRAQQSGFDIFANPFTTDVCTAP-------------QH
Anguilla_anguil SCARLATKLSRLMNEFDRRFSDFKAQHSGFAIFASPFTTDVSSAP-------------HH
Larimichthys_cr PFAQFAEKLILLGAEFTRRFADFEAQKCRFELLSNPFAFDVNNAP-------------SN
Chelmon_rostrat PSAQFAEKINALSVEFSRRFADFEAQKGRFELLSNLFAIDVESAP-------------TS
Maylandia_zebra PCAQFAEKLCVLSTEFTWRFADFDAQKCRFELLSNPFAVDVAKAP-------------TN
Salvelinus_nama PCAQFAEKLSVLAAEFSRRFADFDVQKCRFELLSNPFAVDVENAP-------------TN
Salmo_trutta    PCAQFAEKLSVLAAEFSRRFADFDVQKCRFELLSNPFAVDVENAP-------------TN
```

```
Oncorhynchus_ki PCAQFAEKLSVLAAEFSRRFADFDAQKCKFELLSNPFAVDVENAP-------------TN
Bufo_bufo       PCAQFAEKLSVLGAEFSRRFADFDVQKCRFELLSNPFAVNVENAP-------------TN
malaclemys_terr PNATFAEKLSALRTEFARRFSDFEAQKSNFELLRNPFAVDVETAP-------------VE
Trachemys_scrip PNATFAEKLSALRTEFARRFSDFEAQKSNFELLRNPFAVDVETAP-------------VE
Etheostoma_spec PNATFAEKLSALRTEFARRFSDFEAQKSNFELLRNPFAVDVETAP-------------VE
Scleropages_for PNTHFADKLSAPRTEFARRFGDFEEQKKNFEFLRNPFAVDVETAP-------------VQ
Syngnathus_acus PNTHFAVKLSALRTEFARRFGDFEEQKKNFELFRNPFAVDVESAP-------------VQ
Thalassophryne_ PNMHFADKLSALRTEFARRFGDFEEQKKNLELLRNPFAVDVETAP-------------VQ
Scophthalmus_ma PNTHFADKLSALRTEFARRFGDF----------RNPFAVDVETAP-------------VQ
Xenopus_tropica ------------------------------------------------------------
Collichthys_luc SGEKYVDVILKLQEEFDHRFADFKTHRATFQIFADPFSFDVQDAP-------------PV
Paralichthys_ol ------------------------------------------------------------
Cottoperca_gobi NVKKYCTSLDDMHREFCRRFSDFEKIDKSLQLVSCPLSQDPETAP-------------HE
Alligator_missi NLKKYVSLLVDLHAEFSCRVEDFQMIEKDLSLVSSPFTFNVDSAP-------------CD
Parambassis_ran HLRRYSSMLGALHGEFSRRFEDLRTIEDEMHMISSPFTYSVDNAP-------------TD
Perca_flavescen ------------------------------------------------------------
Gadus_morhua    DTPKYCNILSDLVVEFDHRFEDFHGNEAAFELFAQPFAVNVDAVS-------------EE
Archocentrus    DLQQLAGFTSNLLQSFKARFGEFRERSRLFKFITHPHECAVDSADLSYIPG----VSVRD
Takifugu        NCDYFHRAITAMQAAFEKRFSEFRKEKQTLSFPVAPLNSDPSLLNTSAFTG----VSKPD
Oryzias_latipes NSQYLKSVITAMQTSFGKRFCEFRQEKHTLSFPVTPLTIDPSQLNMTAFAGV---VSQPD
Erpetoichthys   DTNYFSTVIKKIKDEFADRFEQFKTNKTTLAFIVNPLNTNSNEIHLEPFG-----IDTGS


Sphaeramia_orbi LEDEILTLQNDIEMKARSTSAQR-------------GEH---------------VVFWKL
Labrus_bergylta VEDEILTLQADIQLKSRA--------------------H---------------GEFWNL
Labeo_rohita    VEDEILTLQADIQLKSRA--------------------H---------------GQFWNL
Xiphophorus     SELEMVELREDDRLKSLLKEGPV--------------------------------AFWRV
Xiphophorus_hel SELEMVELREDDRLKSLLKEGPV--------------------------------AFWRV
Gouania_willden YQFELAELQNCDVLKDAFKPNSL-------------------------------INFYAA
Lates_calcarife ------------------------------------------------------------
Anolis_caroline YQMELAELQNCDSLKDAFKPRSL-------------------------------PNFYAS
Kryptolebias_ma LQMELIELQSDSGLKAKFQDAAI-------------------------------QDFYRL
Anguilla_anguil LQLELIELQSDSGLRAKFQDAAI-------------------------------EDFYRL
Larimichthys_cr LQMELIELQCNDTLKSKYDSVGA-------------------------------AQFPPY
Chelmon_rostrat LQMELIELQCNDMLKSKYDSVGA-------------------------------AQFPCF
Maylandia_zebra LQMELIELQYSDTLKSKYNAVGA-------------------------------SQFPHF
Salvelinus_nama IQMELIELQCNDTLKSKYDAVGA-------------------------------AQFPRF
Salmo_trutta    IQMELIELQCNDTLKSKYDAVGA-------------------------------AQFPRF
Oncorhynchus_ki IQMELIELQCNDTLKSKYDAVGA-------------------------------AQFPRF
Bufo_bufo       LQMELIELQCNDTLKSKYDAVGA-------------------------------AQFPQF
malaclemys_terr MQMELIELQCNGTLKAKYDTAGP-------------------------------AQFTRF
Trachemys_scrip MQMELIELQCNGTLKAKYDTAGP-------------------------------AQFTRF
Etheostoma_spec MQMELIELQCNGTLKAKYDTEGP-------------------------------AQFTRF
Scleropages_for IQMELIELQCNGTLKAKYDTAGP-------------------------------AQFIHS
Syngnathus_acus IQMELIELQCNGTLKSKYDTAGP-------------------------------TEFIHS
Thalassophryne_ IQMKLIELQCNGTLKAKYDTAGP-------------------------------PQFIHA
Scophthalmus_ma IQMELIELQCNGTLKAKYDTAGP-------------------------------AQFIRS
Xenopus_tropica IQMELIELQCNGTLKAKYDTAGP-------------------------------AQFIHS
Collichthys_luc LQMELIDLQCNSELKAKFREVSGKVDK--------------------------LGQFLRE
Paralichthys_ol ------------------------------------------------------------
Cottoperca_gobi LQLELIDLQSDSVSQEKFKSLKL-------------------------------NDFYAS
Alligator_missi LQLELIDLQYDALLAEQFQAVPL-------------------------------PSFYAS
```

```
Parambassis_ran VQLELIDLQSDAVLAEHFKSGSL-------------------------------LDFYST
Perca_flavescen ------------------------------------------------------------
Gadus_morhua    LQMELLELQSDSDLCCRFRELPL-------------------------------QDFYKS
Archocentrus    FELQVADLKASDMWVNKFKSLNEDLERLAQQQAELASKHKWGEMKKLQPADQLIVKTWKA
Takifugu        LEIELADIADKVLWVNKFKSLSADLEEVVRQRATLAKEHKWSDMEQLPQPDKLIFATWNA
Oryzias_latipes LEMELADI-----WVFKFKSLTAYLENVSRQKAVLAQNHKWSDIANLPKQDKLVFETWNA
Erpetoichthys   LEMQLIDLKSKALWSGKFTELKSKLEELEVQKYMYVTQQKWTALKEMPRVEALIFDAWNS


Sphaeramia_orbi LVAEKYPNLRRCALNLTALFGSTYLCESAFSHMNIIKSKYRSTMTDDHLAACLRLATSS-
Labrus_bergylta LTEEKYPNMRKCATSLTALFGSTYLCESAFSHMKIIKSKYRSTITDGHLEVCLRLAVSS-
Labeo_rohita    LTEEKYPNMRKCATSLTALFGSTYLCESAFSHMKIIKSKYRSTMTDDHLEVCLRLAISS-
Xiphophorus     VPTEKYPCVKRAALKLLSMFSSTYVCESLFSTLKHVKSKHRSVLTDTHVKELLRVATTE-
Xiphophorus_hel VPTEKYPCVKRAALKLLSMFSSTYVCESLFSTLKHVKSKHRSVLTDTHVKELLRVATTE-
Gouania_willden LPNDTYPNIKKHAMKMSTLFGSTYICEQTFSHMKHLKTPTRSRLTDEHLHQCLRLAVTR-
Lates_calcarife ------------------------------------------------------------
Anolis_caroline LPSETYPNLRNHALKMATIFGSTYVCEQTFSRMKHLKSPTRSRLTDAHLHHLLRLAVTN-
Kryptolebias_ma MPPGLMPQLRLHAARVLSMFGSTYLCEQFFSMMNLNKNQHRSRLTDDNLHAVLRIASAQD
Anguilla_anguil LPPGVMPQLRLHAARVLSMFGSTYLCEQMFSIMNLNKTKHRSRITDDNLHAVLRIATAQE
Larimichthys_cr L-PDTLPELRAQAAQMLSMFGSTYLCEQLFSLMKMNKTPHRSRLTDEHLHSVLRISSAQS
Chelmon_rostrat L-PDTMPQLRTQAAQMLSMFGSTYLCEQLFSMMKVNKTPHRSRLTDEHLHSVLRLSSAQS
Maylandia_zebra I-PDTMPGLRTHAAQMLSMFGSTYLCEQLFSSMKMAKTSHRRRLTDEHLHSILRISSAQS
Salvelinus_nama I-SDTMPQLRTQAAQMLSMFGSTYLCEQLFSSMKMTKTTHRRRLTDEHLRSILRISSAQR
Salmo_trutta    I-PDTMPQLRTQAAQMLSMFGSTYLCEQLFSSMKMTKTTHRRRLTDEHLRSILRISSAQS
Oncorhynchus_ki I-PDTMPQLRTQAAQMLSMFGSTYLCEQLFSSMKMTKTTHRRRLTDEHLRSILRISSAQS
Bufo_bufo       I-PDTMPQLRTQAAQMLSMFGSTYLCEQLFSSMKMTKTSHRRRLTDEHLRSILRISSAQS
malaclemys_terr I-PEAMQQLRQHAARILSMFGSTYLCEQLFSVMKINKTSHRSRLTDEHLQSILRIFTTQN
Trachemys_scrip I-PEAMPQLRQHAARILSMFGSTYLCEQLFSVMKINKTSHRSRLTDEHLQSILRIFTTQN
Etheostoma_spec L-PEAMPQLRQHAARILSMFGSTYSGAQKFTYTCLS------------------------
Scleropages_for I-PAEMPQLRLHAARTLCMFGSTYLCEKLFSVMKTNKTAHGSRLTDEHLQSILRISTTQN
Syngnathus_acus I-PAAMSQLRLHVARTLCMFGSTYLCEKLFSVMKTNKTAHRSRLTDEHLQSILRVSTTRD
Thalassophryne_ I-PAEMPQLRLHVARTLCMFGSTYLCEKLFSVMKTNKTAHRSHLTDEHLQSILRISTTQK
Scophthalmus_ma I-PETMPQLRLHAAQTLCMFGSTYLCEKLFSVMKMNKTAHRSRLTDGHLQSILRISTAQE
Xenopus_tropica I-PAEMPQLRLHAARTLCMFGSTYLCEKLLSVMKTNKTAHRRHLTDEHLQSILRIST-QN
Collichthys_luc L-TPSFPELSRMFKRTMCLFGSTYLCEKLFSTLNFNKSKYRSRLTDDHLQAILRVSTASS
Paralichthys_ol ------------------------------------------------------------
Cottoperca_gobi LNEATFPNLRRTAQKMLALFGSTYVCEQAFSVMNINKARHRSRLTDQHLRSILRIATTK-
Alligator_missi LNEQKFPKIKTHARKMLVLFGSTYVCEQTRSVMKVNKSNLRSSMSGDHLAAVLRIATTE-
Parambassis_ran LKEENFPNMRRHAQKMLVLFGSTYICEQTFSMMKFAKSTHRSSLTDDHLSAVLRISTSN-
Perca_flavescen ------------------------------------------------------------
Gadus_morhua    VPAHRYGKIRKHAQVMFSLFGSTYVCEQAFSLMNLNKSKLRNALSDSHLHDILTLSVSQ-
Archocentrus    L-PVTYHTLQRVSIAVLTMFGSTYACEQSFSHLKNIKTNLRSRLTDGSLNSCMKLNLTT-
Takifugu        I-PDTYINMKRCAFGVLSIFGSTYLCEQVFSSMNIIKSKYRSRFTSETLQSCVKMKVTS-
Oryzias_latipes I-PDSYINMKRYAFGVLEIFGSTYICEQVFSNVNFIKNKHRSRLTHISLRSCLKMKVTS-
Erpetoichthys   L-PDCYSEVKKLAFGVLTIFGSTYS-----------------------------------
                                          *

Sphaeramia_orbi YTPNYEKLASSS-QCQVSH--------
Labrus_bergylta YCPDYASLADSI-QCKSSE--------
Labeo_rohita    YCPDYASLADSI-QCKSSE--------
Xiphophorus     YQPDLQRITRNK-RCQKSH--------
Xiphophorus_hel YQPDLQRITRNK-RCQKSH--------
```

```
Gouania_willden MEPDINLLTSQM-QAHSSH--------
Lates_calcarife ---------------------------
Anolis_caroline MEPDIDYLISQK-QAHSSH--------
Kryptolebias_ma LKPDIDTLATGK-RCQTSGQKNTG---
Anguilla_anguil LKPDIDTLAKGK-RCQTSGQKTH----
Larimichthys_cr LTPEIDALACKK-RCQVSGLDPCASSE
Chelmon_rostrat LTPDLDELASKK-RCQVSGLDQCAE--
Maylandia_zebra LSPDIEELASKK-RCQVSGLDASE---
Salvelinus_nama LSPDIDELPSKK-RCQVSGLGTSD---
Salmo_trutta    LSPDIDELASKK-RCQVSGLGTSD---
Oncorhynchus_ki LSPDIDELASKK-RCQVSGLGTSD---
Bufo_bufo       LSPDIDELSSKK-RCQVSGLGTSD---
malaclemys_terr LTPNINELVAKK-RLQVSGSD------
Trachemys_scrip LTPNINELVAKK-RLQVSGSD------
Etheostoma_spec ---------------------------
Scleropages_for LTPNINELVAKK-RCQASSSDKMT---
Syngnathus_acus LTPNINQLVAKK-RCQSSGSDKMA---
Thalassophryne_ LTPNIKELVAKK-RCQASSFDKTT---
Scophthalmus_ma LTPNLNDLTAKK-RCQTSCSDKMA---
Xenopus_tropica LTPNINNLFV----CQASSSDKMT---
Collichthys_luc LHPNVARLCERR-RCQVSGSKK-----
Paralichthys_ol ---------------------------
Cottoperca_gobi LTPDFDALAKKGDQQHCSH--------
Alligator_missi MTPDFDFLVNAHQRLHSSP--------
Parambassis_ran IQPDFDALVKAQQRLDFTH--------
Perca_flavescen -------------REQSVYGGHGH---
Gadus_morhua    LEPDINRLVKTKDRLHVSH--------
Archocentrus    YQPDYKAISKTM-QHQKSH--------
Takifugu        YSADIGKICREM-QTQKSH--------
Oryzias_latipes YSPDVKKLCSEV-QEQKSH--------
Erpetoichthys   ---------------------------
```
