# Supplementary figures and images for: Fake IDs? Widespread misannotation of DNA transposons as a general transcription factor
Source: Genome Biol. 2023 Nov 13;24:260. doi: 10.1186/s13059-023-03102-9 (PMC10641963; doi:10.1186/s13059-023-03102-9)

Hermes transposase

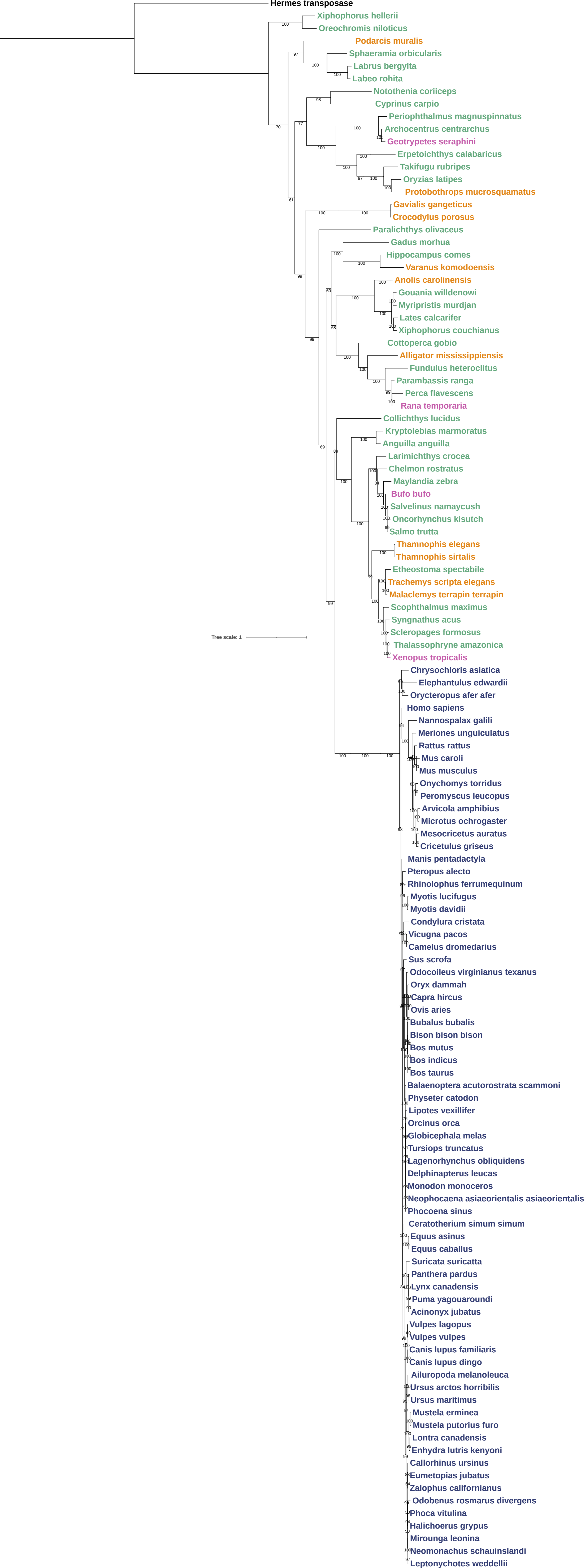

Supplement: Supplementary file 4 — Additional file 4. Full support values for Fig. 1 phylogenetic tree. [file 13059_2023_3102_MOESM4_ESM.pdf]
