# Supplementary material for: Fake IDs? Widespread misannotation of DNA transposons as a general transcription factor
Source: Genome Biol. 2023 Nov 13;24:260. doi: 10.1186/s13059-023-03102-9 (PMC10641963; doi:10.1186/s13059-023-03102-9)
Supplement: Supplementary file 5 — Additional file 5. Peer review history. [file 13059_2023_3102_MOESM5_ESM.docx]

**Review history**

**First round of review**

**Reviewer 1**

Summary: In this paper, Hassan and Adelson identify a family of hAT transposons in reptile genomes that have been misannotated as transcription factors due to their similarity to mammalian GTF2IRD2. They verify this using a combination of manual curation (identifying TIRs, TSDs, catalytic residues, and other hallmarks of transposon) and comparative genomics. The authors also determine that this is not an isolated event, but rather a pervasive, systematic issue with automated gene annotation, such that many autonomous transposons are annotated as genes in species whose genomes are not well curated (ie: non mammals/birds). They conclude that this mis annotation is a problem for any field relying on quality annotations and recommend that an additional TE filtering step during genome annotation could mitigate many of these issues.

Comments: The authors bring to light a pervasive but largely unappreciated flaw in automated genome annotation pipelines, and they do so in a very scientifically rigorous way. This flaw is something I have personally noticed during my research, and although I know what to look for, I think most researchers, especially those unfamiliar with transposons, would not make this connection and design experiments under erroneous assumptions. As such, I think it is an important study that will increase awareness of this problem. Moreover, the study is timely, as more and more high-quality genomes are sequenced and annotated. My only suggestion is that I think the GTF2IRD2 analyses could have one additional component to bolster their claims - namely, whether sequences with similarity to the N-terminus of GTF2IRD2 are located upstream of the transposons in the genome, such that they could be plausibly spliced to the transposons as is the case for GTF2IRD2. My instinct is that they won't be there, which would further support the author's assertion that these sequences are indeed hAT transposons as opposed to fusion genes.

**Reviewer 2**

I have reviewed the manuscript by Hassan & Adelson, which describes misannotation of hAT DNA transposons as transcription factors in the genomes of various non-mammalian and non-avian vertebrate genomes. I commend the authors on doing the detailed detective work that is necessary to uncover a good example of this data analysis issue and working to publish these results as a cautionary tale for how repeat/gene annotations can be erroneous. I anticipate that this paper will help others to avoid mis-annotation of these specific repeats and will encourage methods development that helps researchers to avoid mis-annotation of repeats and genes. Altogether, I think this manuscript is a nice contribution to repeat element annotation and analysis, and only have some relatively minor copy edits , some suggestions for improving readability of the paper, and some suggestions for ways in which the authors can ensure their paper in reproducible, as some important information is currently missing. My critiques should be relatively easy to address.

Comments:
1. Page 1, line 57: What exactly are DDE amino acids? This acronym should be written out the first time it is used and briefly defined for readers who do not have field-specific knowledge of this terminology.
2. Page 1, line 59: What, specifically, is meant by "old". Do you mean the element was identified a long time ago? Or that it was active in mammal genomes longer ago (in which case, I would use "ancient" instead)?
3. Page 2, line 27: Add comma between "transcription factor" and "was the top".
4. Page 3, line 25: "transcription factor" should be written out the first time that TF is used.
5. Page 3, line 31: Change to "…DNA transposons as they appears more closely related to hAT-6 transposons…".
6. Page 3, line 36: The authors specifically mention Salmo salar but no species with that name is present in Table 1, so I'm not sure where this comment comes from. Moreover, as best I can tell from Table 1, hAT transposons are equally abundant in each genome (similar lengths). What is the word "particularly" referring to? It suggests this occurs many times, but there are not data present to confirm that.
7. Page 5, line 13: "at least" and "or more" are redundant, so keep one or the other.
8. Page 5, line 40-42: The authors refer to a specific hAT transposon, hAT-6_TSE. I'm assuming "(in prep)" means that the publication describing this TE is in preparation, but please make this clear in the text. If the genome/coordinates or a consensus sequence is not available at this time, this information should be provided as a supplement to this manuscript for reproducibility.
9. Page 5, line 51: Here, "DD/E" is used, but "DDE" is used in other locations in the manuscript. Please select one and use consistently throughout.
10. Page 6, line 27: Change to "…protein-coding regions (CDS), which were selected for analysis."
11. Methods: Some software has version numbers but that's not the case with all software. Version numbers should be reported for all software for reproducibility. See BLASTP, CLipKit, IQTree, BEDTools, and RepeatMasker, but there may be others. The release version (or search date) for Repbase should also be reported.
12. Methods: To aid in reader understanding, I suggest that the authors report the total number of genomes searched and the number each from Actinopterygii, Amphibia, and Reptilia. To aid in understanding and reproducibility, the authors should also include a supplementary table listing all genomes analyzed, where they were sourced from, and the assembly/annotation versions.
13. Abbreviations: I have several suggestions for additional terms used in the manuscript that can be defined for readers: DDE/RW (separately or together), TSD, TIR, ORF, CDS, TE, and TF.
14. Figure 1. The version of the figure I received for review was low resolution, making the node labels difficult to read. Please ensure a readable, high-quality version of the figure is available for final publication.
15. Figure 2: What do the astericks "*" mean in the alignment? This meaning should be briefly described in the caption.
16. Table 1: Aside from the element length, I also suggest that the authors include the chromosome/contig and coordinates for the elements. This would allow others to specifically evaluate the locus in question when working with these species in the future. Right now, they would need to replicate your analysis to do so, so providing this information makes the utility of this study broader. I encourage the authors to think of ways like this to broaden the impact or usability of their results through actions like this.
17. Supplementary Materials: In general, I encourage the authors to include a manifest or README file documenting the supplementary materials, so the authors can understand what each one is. Currently, for many figures, only the file name includes any information, and it is often not complete enough to understand exactly what the resource is and how it is related to mentions of "Supplementary Materials" in the text. Specifically, regarding the resource "missannotated_gene_predictions.pdf", it appears that some information in the table is being cut-off, which limits its utility. The full Excel file or one where the columns are properly resized to include all information should address this issue.

**Reviewer 3**

In the manuscript titled "Fake IDs? Widespread misannotation of DNA Transposons as a General Transcription Factor" Hassan and Adelson describe the misannotation of a DNA hAT transposon as the GTF2IRD2 gene in the annotation of non-mammal genomes. To conclude, the authors advise caution when relying on gene annotation predictions because TE missanotation as protein-coding genes may be more pervasive than currently appreciated - especially for non-mammal species.

General comments:
Hassan and Adelson present a compelling, well written manuscript, which I think should be accepted with very minor revisions - mostly suggestions.

* Table 1) Not everyone may be accustomed to TE ID notation. Since in the MS they refer to Salmo salar hAT transposons (line36 of page 5 of the final submission) they should also point out either in text or in the table caption that hAT-x_SSa are the hAT elements they are referring to.

* Methods used to identify TE misannotations:
- the authors performed a highly stringent analysis that included only instances where 100% of a TE sequence overlaps the CDS of a gene/gene prediction, and manually analyzed a subset of candidates where the TE and the CDS overlap for more than 1000bp. The methods paragraph should be more clear, because their pipeline and filtering criteria can be misunderstood. I suggest providing the entire bedtools code (i.e. bedtools intersect -a TE.gtf -b gene.gtf -f/F -wo > out.txt). Similarly, they should specify the blast code used to identify the best TE match (if blast it was).

- I am curious; how frequent is the misannotation of TEs? The authors could report a metric, like a percentage, so that the count of misannotated TEs according to their filtering parameters is scaled to the number of unique genes for a species in their intersectBed hits.
I am wondering because TE exonization in CDS is fairly common in mammals - at least in theory. In such instances, 100% of a TE sequence (as annotated in RepeatMasker) is part of a coding exon. Have the authors considered this possibility, since it is not openly discussed?
- This brings me to the considerations the authors made about Zinc Finger binding proteins: at least in mammals, some KRAB/Zinc Finger proteins evolved from TEs to silence the expression of the TEs themselves (i.e., 10.1016/j.devcel.2016.02.024). Same goes for some microRNAs, but that's not the scope of the manuscript. I think the authors should use more care, or be more specific, when discussing the specific case of Zinc Fingers.

* I praise the authors for clarifying how gene annotation pipelines works - this is something that (unfortunately) not many researchers are aware of.

* Figure 1) The main point of the figure is to show the discrepancies between species tree and GTF2IRD2/2A gene tree. Whereas it is easier to see such discrepancies for non-mammal species, if there are any within mammals (there may not be) their identification requires a very close look at the tree (and knowledge of mammalian phylogeny in latin). While not required, may be beneficial to highlight somehow -maybe in bold- species for whom the gene tree does not reflect the species tree.

**Authors’ response to reviewers**

Reviewer reports and RESPONSES IN CAPS

Reviewer #1: Summary: In this paper, Hassan and Adelson identify a family of hAT transposons in reptile genomes that have been misannotated as transcription factors due to their similarity to mammalian GTF2IRD2. They verify this using a combination of manual curation (identifying TIRs, TSDs, catalytic residues, and other hallmarks of transposon) and comparative genomics. The authors also determine that this is not an isolated event, but rather a pervasive, systematic issue with automated gene annotation, such that many autonomous transposons are annotated as genes in species whose genomes are not well curated (ie: non mammals/birds). They conclude that this mis annotation is a problem for any field relying on quality annotations and recommend that an additional TE filtering step during genome annotation could mitigate many of these issues.

Comments: The authors bring to light a pervasive but largely unappreciated flaw in automated genome annotation pipelines, and they do so in a very scientifically rigorous way. This flaw is something I have personally noticed during my research, and although I know what to look for, I think most researchers, especially those unfamiliar with transposons, would not make this connection and design experiments under erroneous assumptions. As such, I think it is an important study that will increase awareness of this problem. Moreover, the study is timely, as more and more high-quality genomes are sequenced and annotated. My only suggestion is that I think the GTF2IRD2 analyses could have one additional component to bolster their claims - namely, whether sequences with similarity to the N-terminus of GTF2IRD2 are located upstream of the transposons in the genome, such that they could be plausibly spliced to the transposons as is the case for GTF2IRD2. My instinct is that they won't be there, which would further support the author's assertion that these sequences are indeed hAT transposons as opposed to fusion genes.

RESPONSE #1: THANK YOU FOR YOUR FEEDBACK AND RECOGNISING THE IMPORTANCE OF OUR STUDY. OUR INITIAL BLAST SEARCHES THAT REVEALED THE MISANNOTATION SHOWED NO EVIDENCE OF UPSTREAM N-TERMINUS LIKE SEQUENCES FOR GTF2IRD2. WE HAVE PROVIDED AN ALIGNMENT OF THE N-TERMINUS OF GTF2IRD2 WITH RESPECT TO MISANNOTATED TRANSPOSABLE ELEMENTS IN FIGURE 1B OF THE REVISED MANUSCRIPT (FORMERLY FIGURE 2 OF THE ORIGINAL MANUSCRIPT). THIS FIGURE HIGHLIGHTS HOW ANY HOMOLOGY IS INDEED LIMITED DOWNSTREAM OF GTF2IRD2 WHICH SUPPORTS OUR CLAIM OF MISANNOTATED TES RATHER THAN INSTANCES OF FUSION GENES. WE ALSO PROVIDE A FULL MULTIPLE ALIGNMENT OF SEVERAL MAMMALIAN GTF2IRD2 WITH OTHER MISANNOTATED TRANSPOSABLE ELEMENTS IN ADDITIONAL FILE 3 SHOWING LIMITED TO NO N-TERMINUS HOMOLOGY.

Reviewer #2: I have reviewed the manuscript by Hassan & Adelson, which describes misannotation of hAT DNA transposons as transcription factors in the genomes of various non-mammalian and non-avian vertebrate genomes. I commend the authors on doing the detailed detective work that is necessary to uncover a good example of this data analysis issue and working to publish these results as a cautionary tale for how repeat/gene annotations can be erroneous. I anticipate that this paper will help others to avoid mis-annotation of these specific repeats and will encourage methods development that helps researchers to avoid mis-annotation of repeats and genes. Altogether, I think this manuscript is a nice contribution to repeat element annotation and analysis, and only have some relatively minor copy edits, some suggestions for improving readability of the paper, and some suggestions for ways in which the authors can ensure their paper in reproducible, as some important information is currently missing. My critiques should be relatively easy to address.

Comments:
1. Page 1, line 57: What exactly are DDE amino acids? This acronym should be written out the first time it is used and briefly defined for readers who do not have field-specific knowledge of this terminology.
2. Page 1, line 59: What, specifically, is meant by "old". Do you mean the element was identified a long time ago? Or that it was active in mammal genomes longer ago (in which case, I would use "ancient" instead)?
3. Page 2, line 27: Add comma between "transcription factor" and "was the top".
4. Page 3, line 25: "transcription factor" should be written out the first time that TF is used.
5. Page 3, line 31: Change to "…DNA transposons as they appears more closely related to hAT-6 transposons…".
6. Page 3, line 36: The authors specifically mention Salmo salar but no species with that name is present in Table 1, so I'm not sure where this comment comes from. Moreover, as best I can tell from Table 1, hAT transposons are equally abundant in each genome (similar lengths). What is the word "particularly" referring to? It suggests this occurs many times, but there are not data present to confirm that.
7. Page 5, line 13: "at least" and "or more" are redundant, so keep one or the other.
8. Page 5, line 40-42: The authors refer to a specific hAT transposon, hAT-6_TSE. I'm assuming "(in prep)" means that the publication describing this TE is in preparation, but please make this clear in the text. If the genome/coordinates or a consensus sequence is not available at this time, this information should be provided as a supplement to this manuscript for reproducibility.
9. Page 5, line 51: Here, "DD/E" is used, but "DDE" is used in other locations in the manuscript. Please select one and use consistently throughout.
10. Page 6, line 27: Change to "…protein-coding regions (CDS), which were selected for analysis."
11. Methods: Some software has version numbers but that's not the case with all software. Version numbers should be reported for all software for reproducibility. See BLASTP, CLipKit, IQTree, BEDTools, and RepeatMasker, but there may be others. The release version (or search date) for Repbase should also be reported.
12. Methods: To aid in reader understanding, I suggest that the authors report the total number of genomes searched and the number each from Actinopterygii, Amphibia, and Reptilia. To aid in understanding and reproducibility, the authors should also include a supplementary table listing all genomes analyzed, where they were sourced from, and the assembly/annotation versions.
13. Abbreviations: I have several suggestions for additional terms used in the manuscript that can be defined for readers: DDE/RW (separately or together), TSD, TIR, ORF, CDS, TE, and TF.
14. Figure 1. The version of the figure I received for review was low resolution, making the node labels difficult to read. Please ensure a readable, high-quality version of the figure is available for final publication.
15. Figure 2: What do the astericks "*" mean in the alignment? This meaning should be briefly described in the caption.
16. Table 1: Aside from the element length, I also suggest that the authors include the chromosome/contig and coordinates for the elements. This would allow others to specifically evaluate the locus in question when working with these species in the future. Right now, they would need to replicate your analysis to do so, so providing this information makes the utility of this study broader. I encourage the authors to think of ways like this to broaden the impact or usability of their results through actions like this.
17. Supplementary Materials: In general, I encourage the authors to include a manifest or README file documenting the supplementary materials, so the authors can understand what each one is. Currently, for many figures, only the file name includes any information, and it is often not complete enough to understand exactly what the resource is and how it is related to mentions of "Supplementary Materials" in the text. Specifically, regarding the resource "missannotated_gene_predictions.pdf", it appears that some information in the table is being cut-off, which limits its utility. The full Excel file or one where the columns are properly resized to include all information should address this issue.

RESPONSE #2: THANK YOU FOR YOUR THOROUGH FEEDBACK TO HELP IMPROVE OUR MANUSCRIPT. WE HAVE INCORPORATED ALL OF THE ABOVE SUGGESTIONS INTO THE REVISED MANUSCRIPT AND ALL CHANGES ARE HIGHLIGHTED. WE HAVE UPDATED ALL SUPPLEMENTARY MATERIALS TO IMPROVE READABILITY AND REPRODUCIBILITY.

Reviewer #3: In the manuscript titled "Fake IDs? Widespread misannotation of DNA Transposons as a General Transcription Factor" Hassan and Adelson describe the misannotation of a DNA hAT transposon as the GTF2IRD2 gene in the annotation of non-mammal genomes. To conclude, the authors advise caution when relying on gene annotation predictions because TE missanotation as protein-coding genes may be more pervasive than currently appreciated - especially for non-mammal species.
General comments:
Hassan and Adelson present a compelling, well written manuscript, which I think should be accepted with very minor revisions - mostly suggestions.

* Table 1) Not everyone may be accustomed to TE ID notation. Since in the MS they refer to Salmo salar hAT transposons (line36 of page 5 of the final submission) they should also point out either in text or in the table caption that hAT-x_SSa are the hAT elements they are referring to.

* Methods used to identify TE misannotations:
- the authors performed a highly stringent analysis that included only instances where 100% of a TE sequence overlaps the CDS of a gene/gene prediction, and manually analyzed a subset of candidates where the TE and the CDS overlap for more than 1000bp. The methods paragraph should be more clear, because their pipeline and filtering criteria can be misunderstood. I suggest providing the entire bedtools code (i.e. bedtools intersect -a TE.gtf -b gene.gtf -f/F -wo > out.txt). Similarly, they should specify the blast code used to identify the best TE match (if blast it was).

- I am curious; how frequent is the misannotation of TEs? The authors could report a metric, like a percentage, so that the count of misannotated TEs according to their filtering parameters is scaled to the number of unique genes for a species in their intersectBed hits.
I am wondering because TE exonization in CDS is fairly common in mammals - at least in theory. In such instances, 100% of a TE sequence (as annotated in RepeatMasker) is part of a coding exon. Have the authors considered this possibility, since it is not openly discussed?
- This brings me to the considerations the authors made about Zinc Finger binding proteins: at least in mammals, some KRAB/Zinc Finger proteins evolved from TEs to silence the expression of the TEs themselves (i.e., 10.1016/j.devcel.2016.02.024). Same goes for some microRNAs, but that's not the scope of the manuscript. I think the authors should use more care, or be more specific, when discussing the specific case of Zinc Fingers.

* I praise the authors for clarifying how gene annotation pipelines works - this is something that (unfortunately) not many researchers are aware of.

* Figure 1) The main point of the figure is to show the discrepancies between species tree and GTF2IRD2/2A gene tree. Whereas it is easier to see such discrepancies for non-mammal species, if there are any within mammals (there may not be) their identification requires a very close look at the tree (and knowledge of mammalian phylogeny in latin). While not required, may be beneficial to highlight somehow -maybe in bold- species for whom the gene tree does not reflect the species tree.

RESPONSE #3: THANK YOU FOR YOUR SUGGESTIONS. WE HAVE NOW INCLUDED A METRIC DESCRIBING THE FREQUENCY OF MISANNOTATION IN ADDITIONAL FILE 1: TABLE S7. WE CALCULATED THE FREQUENCY BY TAKING THE TOTAL COUNT OF TE OVERLAP OF 100% TO THE CDS (EXCLUDING SIMPLE REPEATS) AND DIVIDING IT BY THE TOTAL COUNT OF PROTEIN-CODING GENES IN EACH ASSEMBLY. WE CHOSE TO EXCLUDE SIMPLE REPEATS FOR CONSISTENCY AND TO PROVIDE THE MINIMUM FREQUENCY OF MISANNOTATION EVENTS. WE CHOSE TO USE TOTAL PROTEIN-CODING GENES RATHER THAN UNIQUE GENES AS FOR THE SPECIES INVOLVED THERE IS NO “GROUND TRUTH” FOR THE NUMBER OF UNIQUE GENES.

WHILE EXONISATION IS CERTAINLY POSSIBLE, IT IS BEYOND THE SCOPE OF THIS MANUSCRIPT. HOWEVER, IN THE INSTANCES WE INVESTIGATED WE FOUND FULL OVERLAP OF TE TO THE PREDICTED ZINC FINGER BINDING PROTEIN, WHICH IS MORE INDICATIVE OF MISANNOTATION RATHER THAN EXONISATION. FOR EXONISATION, WE AGREE WITH THE REVIEWER AND WE WOULD EXPECT PARTIAL OVERLAP OF THE TE WITHIN THE ZINC FINGER BINDING PROTEIN, AS WE OBSERVE WITH GTF2IRD2 AND ITS CHARLIE8-LIKE DOMAIN. WITH RESPECT TO THE COMMENT ABOUT THE DISCUSSION OF SPECIFIC ZINC FINGERS, WE ONLY REFER TO THESE TWO TIMES IN THE MANUSCRIPT, ONCE IN A GENERAL FASHION AND SUBSEQUENTLY IN THE CONTEXT OF SPECIFIC ANNOTATIONS OF GENE MODELS WHERE MISANNOTATION WAS EVIDENT.

WE BELIEVE THAT FIGURE 1 HIGHLIGHTS THE DISCREPANCIES AS WE OPTED TO USE COLOUR CODING TO HELP THE READER NOTICE PHYLOGENETICALLY DISCORDANT SPECIES BASED ON GTF2IRD2 AND PREDICTED GTF2IRD2 HOMOLOGY. WE HAVE NOT OBSERVED ANY DISCREPANCIES BETWEEN MAMMALIAN SPECIES BASED ON GTF2IRD2.
